# The feedback loop of METTL14 and USP38 regulates cell migration, invasion and EMT as well as metastasis in bladder cancer

**DOI:** 10.1371/journal.pgen.1010366

**Published:** 2022-10-26

**Authors:** Ji Huang, Weimin Zhou, Chao Hao, Qiuming He, Xinhua Tu

**Affiliations:** Departments of Urology, Jiangxi Cancer Hospital, Nanchang, Jiangxi, China; Dana-Farber Cancer Institute, UNITED STATES

## Abstract

**Background:**

Bladder cancer (BCa) is one of the most prevalent malignancies globally. Previous study has reported the inhibitory effect of methyltransferase-like 14 (METTL14) on BCa tumorigenesis, but its role in the cell migration, invasion and epithelial–mesenchymal transition (EMT) in BCa remains unknown.

**Materials and methods:**

Quantitative real-time PCR (RT-qPCR) and western blot were applied to measure RNA and protein expression respectively. Cell migration, invasion and EMT were evaluated by wound healing, Transwell, and immunofluorescence (IF) assays as well as western blot of EMT-related proteins. *In vivo* experiments were performed to analyze metastasis of BCa. Mechanism investigation was also conducted to study METTL14-mediated regulation of BCa progression.

**Results:**

METTL14 overexpression prohibits BCa cell migration, invasion *in vitro* and tumor metastasis *in vivo*. METTL14 stabilizes USP38 mRNA by inducing N6-methyladenosine (m6A) modification and enhances USP38 mRNA stability in YTHDF2-dependent manner. METTL14 represses BCa cell migration, invasion and EMT via USP38. Additionally, miR-3165 inhibits METTL14 expression to promote BCa progression.

**Conclusions:**

Our study demonstrated that METTL14 suppresses BCa progression and forms a feedback loop with USP38. In addition, miR-3165 down-regulates METTL14 expression to promote BCa progression. The findings may provide novel insight into the underlying mechanism of METTL14 in BCa progression.

## Author summary

Bladder cancer (BCa) is a common type of cancer that begins in the cells of the bladder and poses a significant threat to human health worldwide. In order to improve the diagnosis and treatment of BCa, molecular mechanisms associated with BCa tumorigenesis and tumor progression needs to be clarified. Currently, long non-coding RNAs (lncRNAs) have been suggested to act as regulators of cancer progression. Here, we identified lncRNA methyltransferase-like 14 (METTL14) as a tumor-suppressor gene in BCa, acting to inhibit cell migration, invasion and epithelial–mesenchymal transition (EMT) as well as tumor metastasis. We also found that METTL14 forms a feedback loop with ubiquitin specific peptidase 38 (USP38) in BCa. In addition, microRNA-3165 (miR-3165) was verified as an upstream regulator of METTL14 and was elucidated to downregulate METTL14 expression, contributing to the malignancy of BCa. Given that the therapeutic potential of some miRNAs have been identified in a number of diseases, targeting miR-3165 may be a potential therapeutic strategy in BCa treatment. Our study provides new insights into the understanding of molecular mechanism by which METTL14 regulates BCa progression and offer novel and potential targets for BCa treatment.

## Introduction

Bladder cancer (BCa) is the tenth most commonly diagnosed all over the world according to the GLOBOCAN 2020 Statistics and its incidence displays an upward trend [1]. However, the etiology and pathophysiology of BCa remain unclear. Its incidence may be attributed to smoking, occupational exposure, inflammation, radiation, and chemotherapy [2]. At present, the treatment options include surgery, radiotherapy, chemotherapy and immunotherapy [3]. Nevertheless, advanced BCa presents poor sensitivity to chemotherapy and radiotherapy [4]. Therefore, it is of great significance to investigate the molecular mechanisms in the tumorigenesis of BCa.

N6-methyladenosine (m6A) modification is the most prevalent internal modification in eukaryotic messenger RNA (mRNA) [5]. The mRNA modification exerts a vital function in the regulation of mRNA metabolism, including mRNA stability, translation, and splicing [6]. In addition, emerging evidence supports the roles of m6A modification in human cancers [7–10]. For instance, Zhang et al. elucidated that reduced m6A modification (represented by METTL14 knockdown) promotes malignant phenotypes of gastric cancer cells via activating Wnt/PI3K-Akt signaling pathway [11]. BCa is also no exception. For example, METTL3 enhances tumor proliferation of BCa via promoting pri-miR221/222 maturation in an m6A-dependent manner [12]. In addition, m6A modification of ITGA6 mRNA facilitates the development and progression of BCa [13].

METTL14 (methyltransferase-like 14) is a key methyltransferase of m6A and plays crucial roles in various cancers [14]. For instance, METTL14 inhibits colorectal cancer progression via regulating m6A-dependent primary miR-375 processing [15]. In pancreatic cancer, METTL14 promotes cell proliferation and migration via directly targeting PERP in an m6A-dependent manner [16]. Notably, METTL14 has been reported to be low-expressed in BCa and inhibit the tumorigenesis of BCa [17]. However, the role of METTL14 in the migration, invasion and EMT of BCa cells remains unknown. In addition to biological role, the underlying mechanism of METTL14-mediated m6A modification in BCa progression is much worthwhile to be investigated.

Furthermore, deubiquitination is a posttranslational protein modification common in mammalian cells [18]. Also, deubiquitination is a reverse process of ubiquitination that cleaves ubiquitin chains from target proteins, and consequently regulating the function of target proteins [18]. Deubiquitinating enzymes (DUBs) reverse ubiquitination-mediated protein degradation [19] and are divided into six families, including OTUs (ovarian-tumor proteases), MCPIP (monocyte chemotactic protein-induced protein), UCHs (ubiquitin carboxy-terminal hydrolases), MJDs (Machado–Joseph disease protein domain proteases), USPs (ubiquitin-specific proteases), and JAMMs (JAMM/MPN domain-associated metallopeptidases) [20]. Herein, we aimed to explore the interaction between METTL14 protein and DUB in BCa cells.

Moreover, microRNA (miRNAs) are a group of ~22-nucleotide-long, single-stranded, non-coding RNAs that negatively regulate gene expression post-transcriptionally [21]. Emerging evidence supports that miRNAs can regulate the expression of oncogenes and tumor suppressors, the alterations of which affect cancer progression [22]. For instance, miR-217 accelerates the proliferation and migration of BCa through the inhibition of tumor suppressor KMT2D [23]. Zhao et al. have demonstrated that miR-616 facilitates BCa cells to proliferate and migrate via inhibiting SOX7 [24]. This study also aims to explore the upstream regulator of METTL14 in BCa cells.

In conclusion, the current study intended to explore the effect of METTL14 on the migration, invasion and EMT of BCa cells and probe into the underlying regulation mechanism, which might offer a novel sight into the comprehensive understanding of METTL14 in BCa progression.

## Results

### Overexpression of METTL14 prohibits BCa cell migration, invasion *in vitro* and tumor metastasis *in vivo*

**Fig 1:**
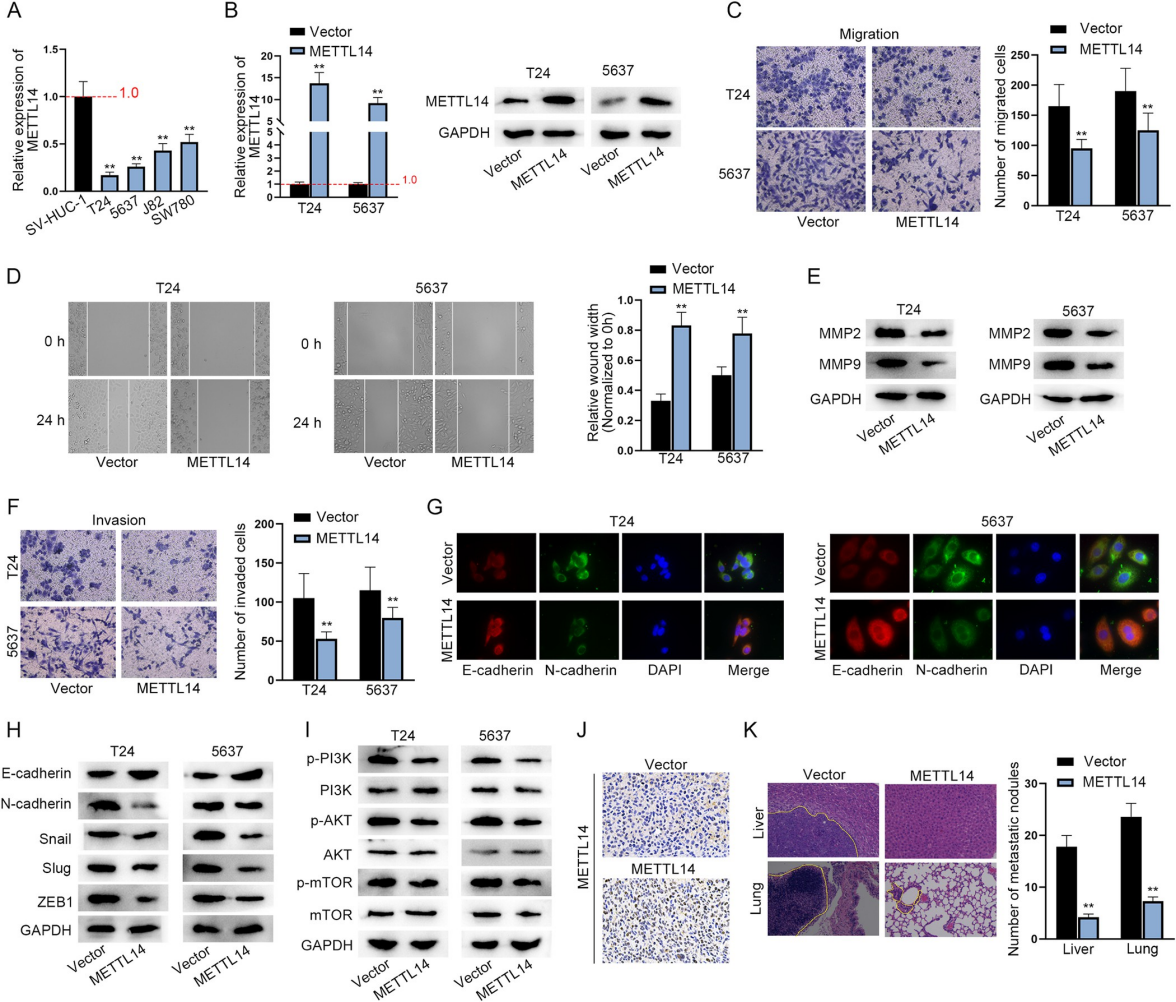
Overexpression of METTL14 prohibits BCa cell migration, invasion *in vitro* and tumor metastasis *in vivo*. A. RT-qPCR was applied to measure the relative expression of METTL14 in human normal uroepithelium cell line (SV-HUC-1) and BCa cell lines (T24, 5637, J82 and SW780). (n = 3) Each bar represents mean (or average); error bars represent SD. P values were determined by one-way ANOVA followed by Tukey’s test. B. RT-qPCR was implemented to measure the relative expression of METTL14 in BCa cells after the transfection of empty pcDNA3.1 or pcDNA3.1-METTL14 to verify the overexpression efficiency of pcDNA3.1-METTL14 (left panel). (n = 3) Each bar represents mean; error bars represent SD. P values were determined by Student’s t test. Western blot was used to examine the protein level of METTL14 in METTL14-overexpressing cells (right panels). C-D. Transwell (migration) and wound healing assays were conducted to determine the migratory ability of BCa cells before and after the overexpression of METTL14. (n = 3) Each bar represents mean; error bars represent SD. P values were determined by Student’s t test. E. Western blot analysis of the expression of migration markers (MMP2 and MMP9) in BCa cells before and after the overexpression of METTL14. F. Transwell (invasion) assay was performed to detect the invasive ability of BCa cells before and after the overexpression of METTL14. (n = 3) Each bar represents mean; error bars represent SD. P values were determined by Student’s t test. G. IF analysis was applied to detect the levels of EMT-related proteins: E-cadherin (an epithelial marker) and N-cadherin (a mesenchymal marker). H. Western blot analysis of the expression of EMT markers and transcriptional factors (Snail, Slug and ZEB1) in BCa cells before and after the overexpression of METTL14. I. Western blot analysis of the expression of PI3K pathway related proteins in BCa cells before and after the overexpression of METTL14. J. Representative images of IHC staining of METTL14 in the xenografts. K. Representative images and quantification results of H&E staining of metastatic nodules in the livers and lungs from the mice injected with METTL14-expressing or vector-transfected T24 cells. (n = 3) Each bar represents mean; error bars represent SD. P values were determined by Student’s t test. **P < 0.01. See also S1 and S4 Data files.

To preliminarily verify whether METTL14 is aberrantly expressed in BCa, RT-qPCR was conducted to measure the relative expression of METTL14 in human normal uroepithelium cell line (SV-HUC-1) and BCa cell lines (T24, 5637, J82 and SW780). It turned out that METTL14 was significantly down-regulated in BCa cell lines in comparison with that in human normal uroepithelium cell line, in particular, T24 and 5637 cells showing a relatively lower expression of METTL14 (Fig 1A). Hence, the following assays were performed to explore the gain-of-function effect of METTL14 on the biological behaviors of BCa cells using T24 and 5637 cells. First, the overexpression efficiency of pcDNA3.1-METTL14 was testified for the subsequent functional assays (Fig 1B). Then, BCa cell migration was determined through Transwell (migration) and wound healing assays, which showed that the migratory ability of BCa cells was inhibited terribly after the overexpression of METTL14 according to the decreased number of migrated cells and increased wound width (Fig 1C and 1D). Likewise, western blot detection of reduced expression of migration markers (MMP2 and MMP9) also indicated that METTL14 hampers cell migration in BCa (Fig 1E). Expectedly, the invasive capacity of BCa cells was significantly impaired by the upregulation of METTL14 (Fig 1F). IF was performed to detect the expression of E-cadherin (an epithelial marker) and mesenchymal markers (N-cadherin, Snail, Slug and ZEB1). An increase in E-cadherin expression and a decrease in N-cadherin expression were caused by the upregulation of METTL14, which suggested that METTL14 overexpression inhibits EMT of BCa cells (Fig 1G). Similarly, western blot analysis of EMT markers and transcriptional factors (Snail, Slug and ZEB1) also verified that METTL14 overexpression suppresses EMT in BCa (Fig 1H). Further, we investigated the impact of METTL14 on the phosphoinositide-3-kinase (PI3K) pathway. Western blot detected decreased levels of phosphorylated proteins (PI3K, AKT and mTOR) in METTL14-overexpressing BCa cells while protein levels of PI3K, AKT and mTOR had no marked change (Fig 1I).

Besides *in vitro* assays, we performed *in vivo* experiments to explore the impact of METTL14 on tumor metastasis. IHC detected a relatively higher expression of METTL14 in the xenografts removed from nude mice injected with METTL14-expressing T24 cells (Fig 1J). The metastasis *in vivo* assay validated the impediment of BCa tumor metastasis caused by the elevation of METTL14 expression based on the reduced number of metastatic nodules (Fig 1K).

In addition to the gain-of-function effects of METTL14 on BCa progression, we investigated loss-of-function effects of METTL14 in SW780 and control SV-HUC-1 cells. SW780 cell line was chosen due to its relatively higher expression of METTL14. Transwell and wound healing assays indicated that ablation of METTL14 promotes the migration and invasion of SW780 and SV-HUC-1 cells (S1A–S1C Fig). The results of IF analysis showed higher expression of N-cadherin and lower expression of E-cadherin in METTL14-deficient SW780 and SV-HUC-1 cells, which revealed that METTL14 knockdown accelerates the EMT of SW780 and SV-HUC-1 cells (S1D Fig).

In short, METTL14 is low-expressed in BCa cell lines and represses BCa cell migration, invasion and EMT *in vitro* and tumor metastasis *in vivo*.

### METTL14 stabilizes USP38 mRNA through m6A modification

**Fig 2:**
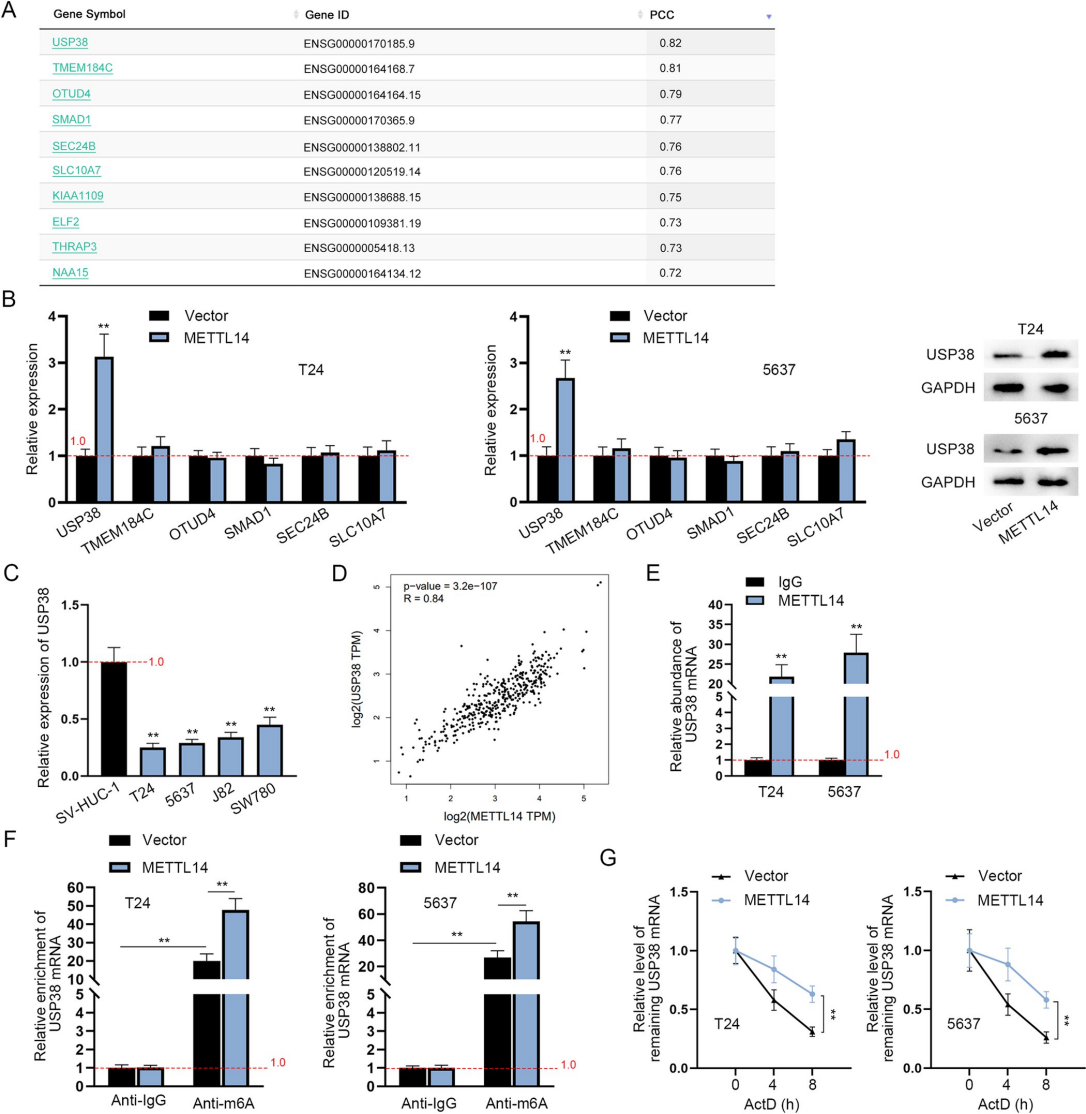
METTL14 stabilizes USP38 mRNA through m6A modification. A. GEPIA analysis of similar genes with METTL14 in BLCA. B. RT-qPCR was carried out to assess the relative expression of 6 similar RNAs with METTL14 (USP38, TMEM184C, OTUD4, SMAD1, SEC24B and SLC10A7) before and after the overexpression of METTL14 in BCa cells (left panels). (n = 3) Each bar represents mean; error bars represent SD. P values were determined by one-way ANOVA followed by Tukey’s test. Western blot of USP38 expression in BCa cells was shown (right panels). C. RT-qPCR was carried out to assess the relative expression of USP38 in SV-HUC-1 and BCa cell lines. (n = 3) Each bar represents mean; error bars represent SD. P values were determined by one-way ANOVA followed by Dunnett’s test. D. GEPIA predicted a positive correlation between METTL14 and USP38 mRNA in BLCA tissues. E. RIP assays were applied to explore the interaction between METTL14 and USP38 in BCa cells. (n = 3) Each bar represents mean; error bars represent SD. P values were determined by Student’s t test. F. Me-RIP assays were conducted to determine the influence of METTL14 on the m6A modification of USP38 mRNA. (n = 3) Each bar represents mean; error bars represent SD. P values were determined by two-way ANOVA followed by Tukey’s test. G. RT-qPCR was used in mRNA stability assays to assess the relative level of USP38 mRNA after the treatment of ActD with or without the upregulation of METTL14 in BCa cells. (n = 3) Each point represents mean; error bars represent SD. P values were determined by Student’s t test. **P < 0.01. See also S4 Data.

Next, we investigated the molecular mechanism by which METTL14 regulates BCa tumor metastasis. With the help of GEPIA (http://gepia.cancer-pku.cn/) analysis with PCC (Pearson correlation coefficient) value > 0.75, USP38, TMEM184C, OTUD4, SMAD1, SEC24B and SLC10A7 were screened out as similar genes with METTL14 in BLCA (bladder urothelial carcinoma; Fig 2A). The relative expression of the candidate genes was detected in METTL14-overexpressed BCa cells through RT-qPCR. Results showed that only USP38 expression was overtly upregulated after the overexpression of METTL14 whereas the expression of the rest exhibited no distinct change (Fig 2B). Moreover, it was revealed by RT-qPCR analysis that USP38 was obviously lower expressed in BCa cell lines than in SV-HUC-1 cell line (Fig 2C). Based on GEPIA analysis, USP38 expression was positively correlated with METTL14 expression in BLCA (Fig 2D). The above results preliminarily verified USP38 as a downstream gene of METTL14 in BCa cells. To attest the interaction of METTL14 with USP38, RIP assays were performed. Results exhibited that USP38 mRNA was highly enriched in METTL14 antibody bound precipitates while being seldom precipitated by IgG antibody (Fig 2E), validating the interaction between METTL14 and USP38 mRNA. Next, Me-RIP assay showed that USP38 was significantly enriched in Anti-m6A groups and METTL14 overexpression caused an increase in the enrichment of USP38 in Anti-m6A bound precipitates (Fig 2F), suggesting the participation of METTL14 in the m6A methylation of USP38. Previous studies have proven that METTL3 [25] and METTL14 [26] could stabilize mRNAs via m6A modification. Therefore, mRNA stability assays were performed with ActD treatment. It was apparently shown that the overexpression of METTL14 effectively prolonged the half-life of USP38 mRNA, indicating that METTL14 could enhance the stability of USP38 mRNA (Fig 2G). Generally, METTL14 promotes USP38 mRNA stability via m6A modification in BCa cells.

### METTL14 enhances the stability of USP38 mRNA via YTHDF2

**Fig 3:**
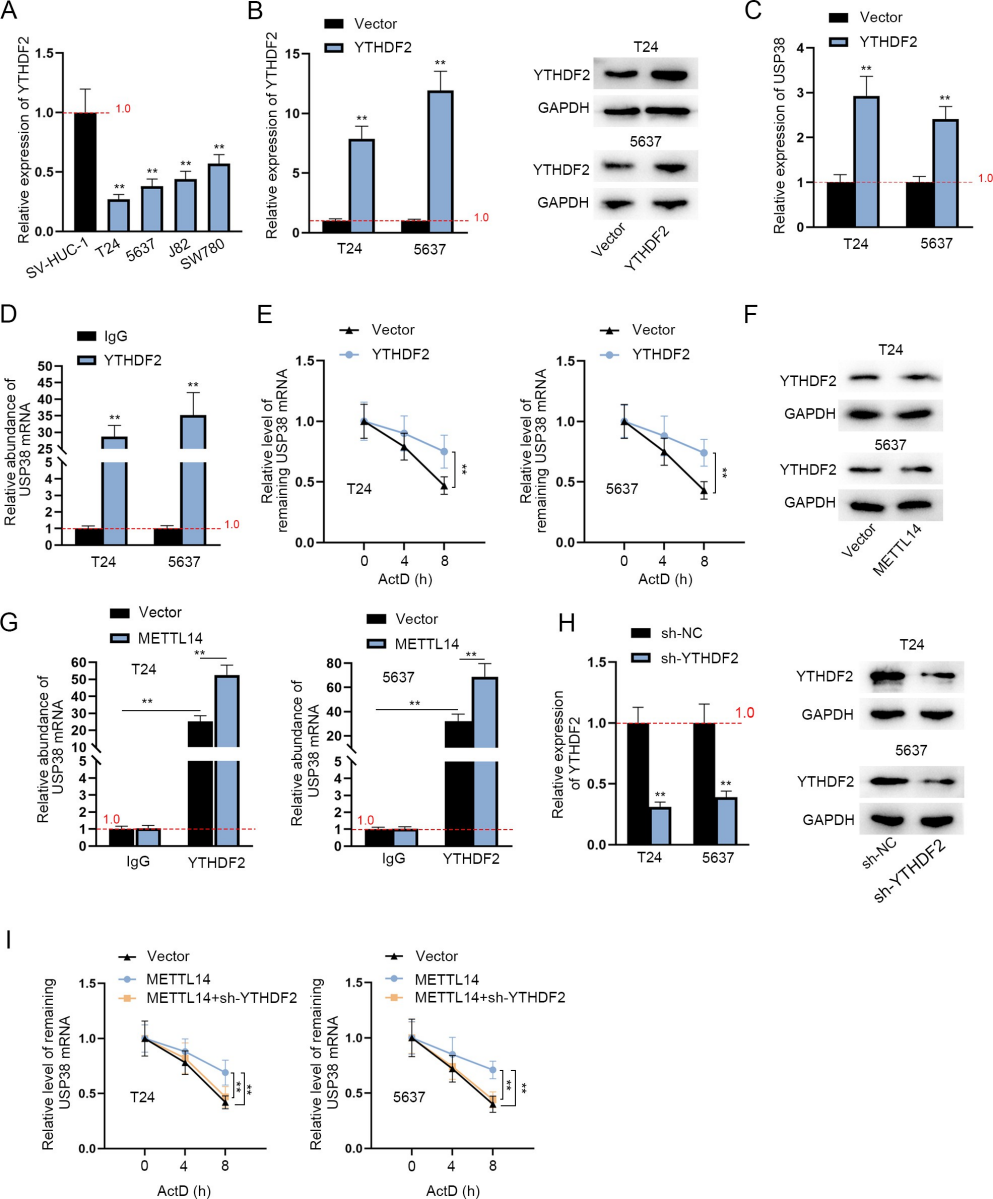
METTL14 enhances the stability of USP38 mRNA via YTHDF2. A. The relative expression of YTHDF2 in human normal uroepithelium cell line (SV-HUC-1) and BCa cell lines (T24, 5637, J82 and SW780) was evaluated by RT-qPCR. (n = 3) Each bar represents mean; error bars represent SD. P values were determined by one-way ANOVA followed by Dunnett’s test. B. The overexpression efficiency of pcDNA3.1-YTHDF2 was verified through RT-qPCR (left panel). (n = 3) Each bar represents mean; error bars represent SD. P values were determined by Student’s t test. Western blot of YTHDF2 protein level was shown (right panels). C. RT-qPCR was conducted to assess the relative expression of USP38 before and after the overexpression of YTHDF2 in BCa cells. (n = 3) Each bar represents mean; error bars represent SD. P values were determined by Student’s t test. D. RIP assays were applied to explore the relation between YTHDF2 and USP38 mRNA in BCa cells. (n = 3) Each bar represents mean; error bars represent SD. P values were determined by Student’s t test. E. RT-qPCR was used to assess the relative level of USP38 mRNA after the treatment of ActD with or without the overexpression of YTHDF2 in BCa cells. (n = 3) Each point represents mean; error bars represent SD. P values were determined by Student’s t test. F. Western blot analysis was applied to measure the protein level of YTHDF2 before and after the overexpression of METTL14 in BCa cells. G. The enrichment of USP38 mRNA in YTHDF2 antibody precipitates was measured in RIP assays before and after METTL14 overexpression. (n = 3) Each bar represents mean; error bars represent SD. P values were determined by two-way ANOVA followed by Tukey’s test. H. The knockdown efficiency of sh-YTHDF2 was tested by RT-qPCR (left panel). (n = 3) Each bar represents mean; error bars represent SD. P values were determined by Student’s t test. Western blot analysis of YTHDF2 protein level in YTHDF2-silenced cells was shown (right panels). I. The relative level of USP38 mRNA was measured via RT-qPCR in the presence of pcDNA3.1-METTL14 (and sh-YTHDF2) in BCa cells. (n = 3) Each point represents mean; error bars represent SD. P values were determined by one-way ANOVA followed by Dunnett’s test. **P < 0.01. See also S4 Data.

Given that the m6A modification consists of three vital components including m6A writers, erasers and readers [27], we next probed into the readers involved in the m6A modification of USP38 mRNA in BCa cells. Previous literature has reported many readers stabilizing mRNAs including YTHDC1, IGF2BP1-3 [28] and YTHDF1-3 [29]. Hence, after the verification of the overexpression efficiency of the overexpression plasmids of the above-mentioned readers, RT-qPCR was performed to measure the expression of USP38 after the overexpression of the seven readers (S2A and S2B Fig). It was clearly shown that only YTHDF2 overexpression led to a significant increase in USP38 mRNA level in both T24 and 5637 cells. Then, GEPIA analysis exhibited a significantly positive correlation between YTHDF2 and USP38 in BLCA tissues (S2C Fig), indicating that YTHDF2 regulates USP38 mRNA level in BCa cells. Nonetheless, it was unclear whether YTHDF2 is mediated by METTL14 to affect USP38 stability. Therefore, we explored the impact of YTHDF2 on USP38 expression. YTHDF2 expression was aberrantly down-regulated in BCa cell lines according to RT-qPCR analysis (Fig 3A). Next, the overexpression efficiency of pcDNA3.1-YTHDF2 was verified (Fig 3B). USP38 level was markedly elevated by YTHDF2 upregulation (Fig 3C). To determine the interaction of USP38 mRNA and YTHDF2, RIP assay was implemented. The results showed that USP38 mRNA was abundantly enriched in the YTHDF2 antibody bound precipitates compared to that in the IgG antibody bound precipitates (Fig 3D). Further, RT-qPCR showed that YTHDF2 overexpression stabilized the expression of USP38 mRNA in BCa cells post ActD treatment (Fig 3E), indicating that YTHDF2 combines with USP38 mRNA to stabilize the expression of USP38 mRNA. Subsequently, we explored whether METTL14 affects the m6A methylation of USP38 mRNA via YTHDF2. Western blot analysis showed that the protein level of YTHDF2 had no marked change after the overexpression of METTL14 (Fig 3F). However, the enrichment of USP38 mRNA in YTHDF2 antibody bound precipitates was largely upregulated by the overexpression of METTL14 (Fig 3G), suggesting the positive effect of METTL14 on the interaction of YTHDF2 and USP38 mRNA. After the attestation of the knockdown efficiency of sh-YTHDF2 via RT-qPCR and western blot analyses (Fig 3H), mRNA stability assay was conducted in BCa cells and the results displayed that the stability of USP38 mRNA enhanced by METTL14 overexpression was blocked by the down-regulation of YTHDF2 (Fig 3I). Taken all above together, METTL14 enhances the stability of USP38 mRNA in the YTHDF2-dependent way.

### METTL14 inhibits BCa cell migration, invasion and EMT by mediating USP38

**Fig 4:**
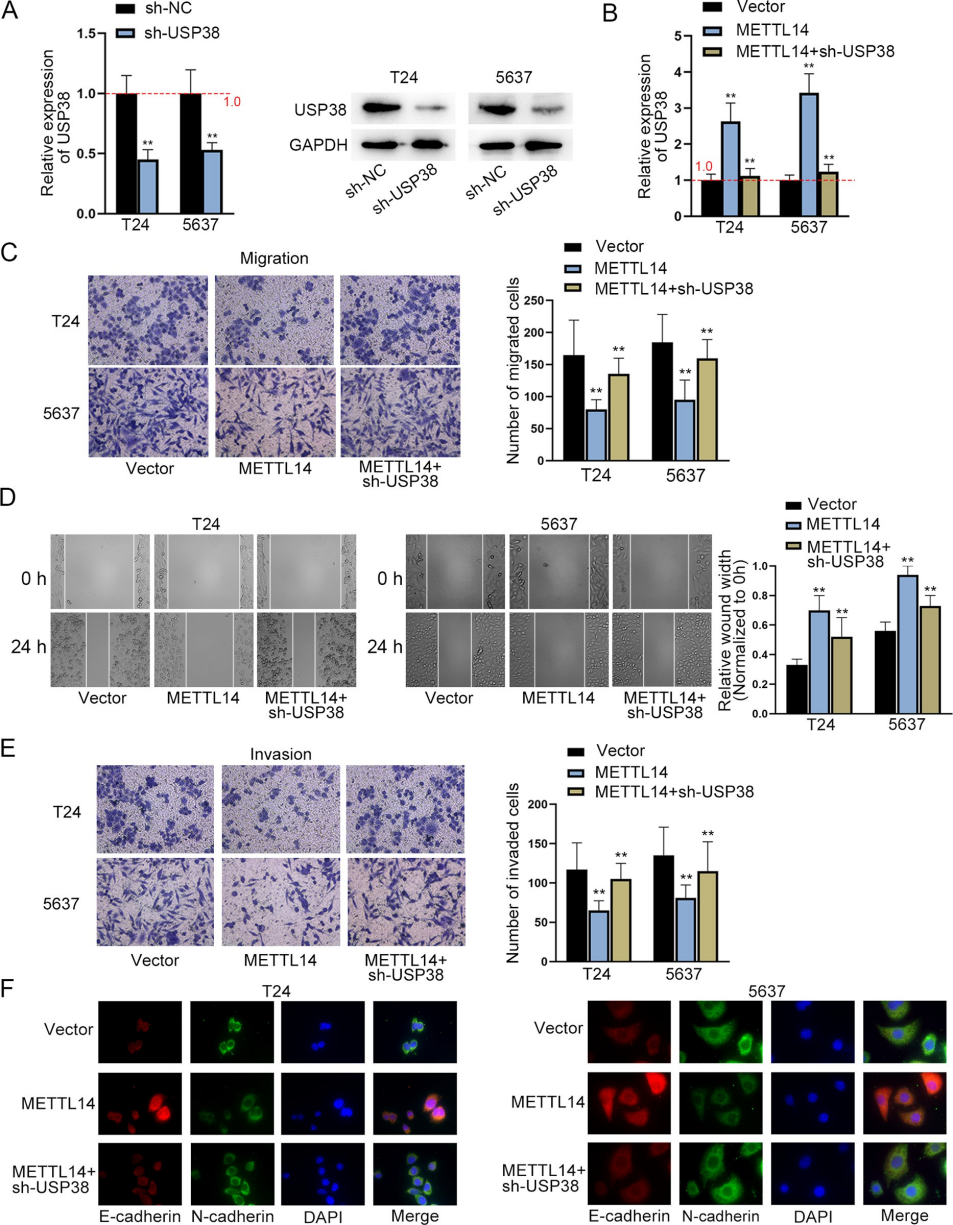
METTL14 promotes BCa cell migration, invasion and EMT by mediating USP38. A. RT-qPCR was used to evaluate the knockdown efficiency of sh-USP38 (left panel). (n = 3) Each bar represents mean; error bars represent SD. P values were determined by Student’s t test. Western blot of USP38 protein level in USP38-silenced cells was shown (right panels). B. RT-qPCR was used to evaluate the relative expression of sh-USP38 after the indicated transfection. (n = 3) Each bar represents mean; error bars represent SD. P values were determined by one-way ANOVA followed by Dunnett’s test. C-D. Transwell (migration) and wound healing assays were conducted to measure the migration of BCa cells after the indicated transfection. (n = 3) Each bar represents mean; error bars represent SD. P values were determined by one-way ANOVA followed by Dunnett’s test. E. Transwell (invasion) assay was conducted to measure the invasion of BCa cells after the indicated transfection. (n = 3) Each bar represents mean; error bars represent SD. P values were determined by one-way ANOVA followed by Dunnett’s test. F. IF assay was applied to detect the levels of EMT-related proteins after the indicated transfection. **P < 0.01. See also S2 and S4 Data files.

Although the *in vitro* assays have proved METTL14 mediates YTHDF2 to enhance the stability of USP38 mRNA in BCa cells, the function of this axis in BCa cells has not been verified. Hence, functional rescue assays were carried out to determine the biological effect of the METTL14/USP38 axis on BC cell migration and invasion. For the following assays, knockdown efficiency of sh-USP38 was attested at first (Fig 4A). RT-qPCR showed the silence of USP38 efficiently countervailed the elevating effect of METTL14 on USP38 mRNA expression (Fig 4B). Then, functional assays were conducted after the overexpression of METTL14 with or without the presence of sh-USP38. Transwell and wound healing assays manifested that the migration and invasion of BCa cells suppressed by METTL14 upregulation was rescued by silenced USP38 (Fig 4C–4E). Consistently, the IF result demonstrated the rescuing effect of sh-USP38 on the EMT of METTL14-overexpressing BCa cells (Fig 4F). CCK-8 assay indicated that METTL14 overexpression inhibits cell growth but this was reversed by USP38 silencing (S3A Fig). In summary, METTL14 suppresses BCa cell migration, invasion and EMT by mediating USP38.

### USP38 mediates the deubiquitination of METTL14 protein

**Fig 5:**
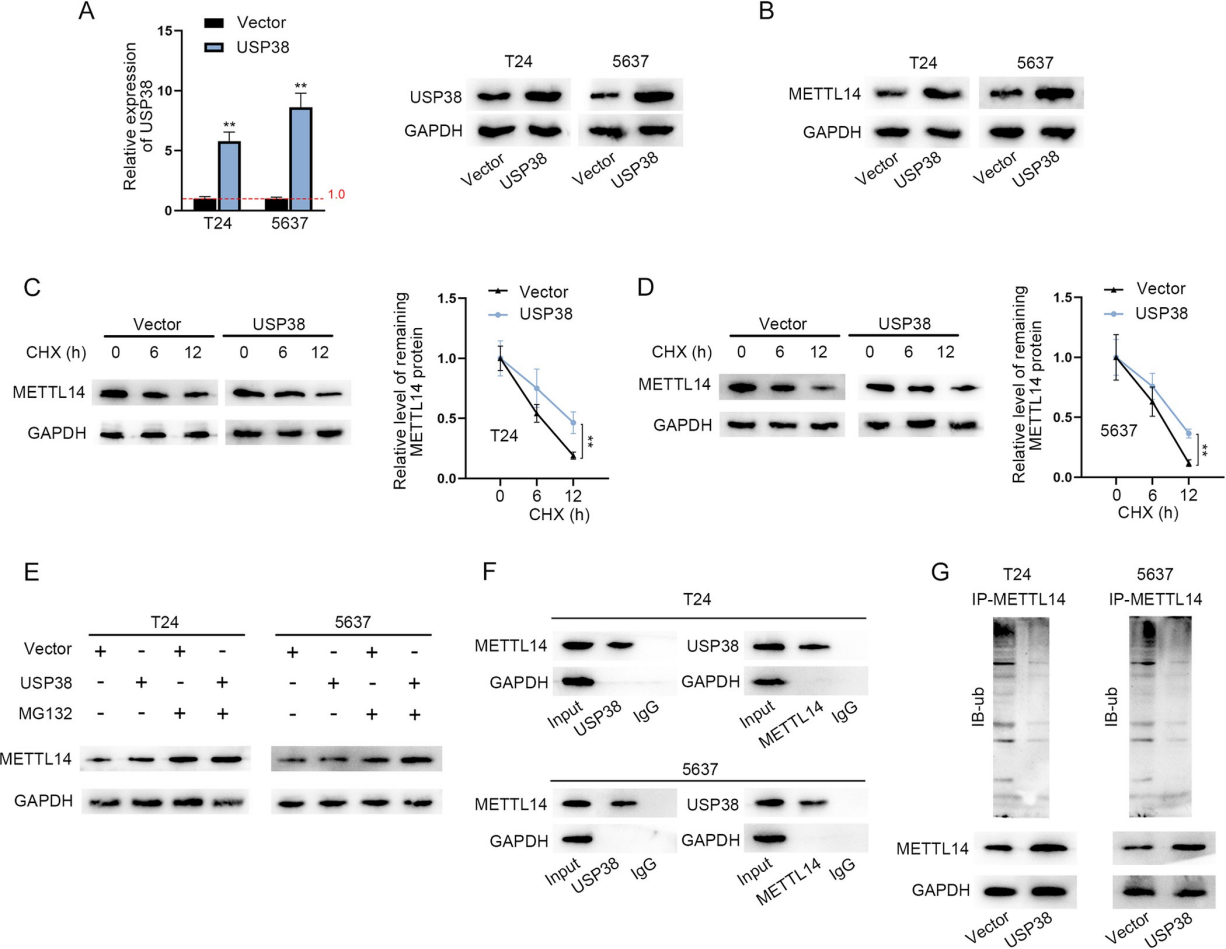
USP38 mediates the deubiquitination of METTL14 protein. A. RT-qPCR was used to evaluate the overexpression efficiency of pcDNA3.1-USP38 in BCa cells (left panel). (n = 3) Each bar represents mean; error bars represent SD. P values were determined by Student’s t test. Western blot of USP38 in USP38-overexpressing cells (right panels). B. Western blot was performed for the measurement of the protein level of METTL14 after the overexpression of USP38 in BCa cells. C-D. Western blot was used to detect the protein level of METL14 after the treatment of CHX for different time points (0, 6 and 12 h) before and after the upregulation of USP38 in BCa cells. (n = 3) Each point represents mean; error bars represent SD. P values were determined by Student’s t test. E. Western blot was conducted to determine the protein level of METTL14 in different groups. F. CoIP assay was implemented to investigate the co-expression of METTL14 and USP38 in BCa cells. G. IP assay followed by western blot analysis was performed to assess the ubiquitination of METTL14 before and after USP38 overexpression. **P < 0.01. See also S4 Data.

USP38 is one of DUBs that could affect the ubiquitination and degradation of proteins [30, 31]. Moreover, it has been reported that the post-translation modification of METTL3 influences the function of METTL3 protein [32] and USP5 (a member of USP family) could deubiquitinate METTL3 protein to stabilize the METTL3-METTL14-WTAP methyltransferase complex [33]. GeneCards (https://www.genecards.org/) also revealed that METTL14 could be modified through the ubiquitination process. Thereby, we wondered whether USP38 regulates the ubiquitination of METTL14 protein to influence its biological function. Through RT-qPCR and western blot analyses, the overexpression efficiency of USP38 overexpression plasmids was determined (Fig 5A). Then, it was discovered that METTL14 protein level was elevated in USP38-overexpressing BCa cells (Fig 5B). Then, the stability of METTL14 protein was assessed utilizing CHX (an inhibitor of protein synthesis) treatment. Results turned out that the accelerated degradation of METTL14 protein was blocked by the overexpression of USP38 in BCa cells (Fig 5C and 5D). Then, western blot analysis of METTL14 expression before and after MG132 (an inhibitor of proteasome) treatment was conducted and the results revealed that the promotive influence of USP38 overexpression on METTL14 protein level was ineffective at the presence of MG132 (Fig 5E). Hence, it was determined that USP38 prohibits the degradation of METTL14 protein in BCa cells. Next, the combination between METTL14 and USP38 was explored through CoIP assays. The co-expression of METTL14 and USP38 proteins were discovered based on the result that the above two proteins could be precipitated by the antibodies of each other (Fig 5F). The subsequent analysis on the ubiquitination of METTL14 through IP assay further validated the suppressive effect of USP38 upregulation on the ubiquitination of METTL14 protein to upregulate METTL14 protein level (Fig 5G). Therefore, it was concluded that USP38 enhances the stability of METTL14 protein through the deubiquitination of METTL14 in BCa cells.

### MiR-3165 regulates METTL14 as an upstream mediator

**Fig 6:**
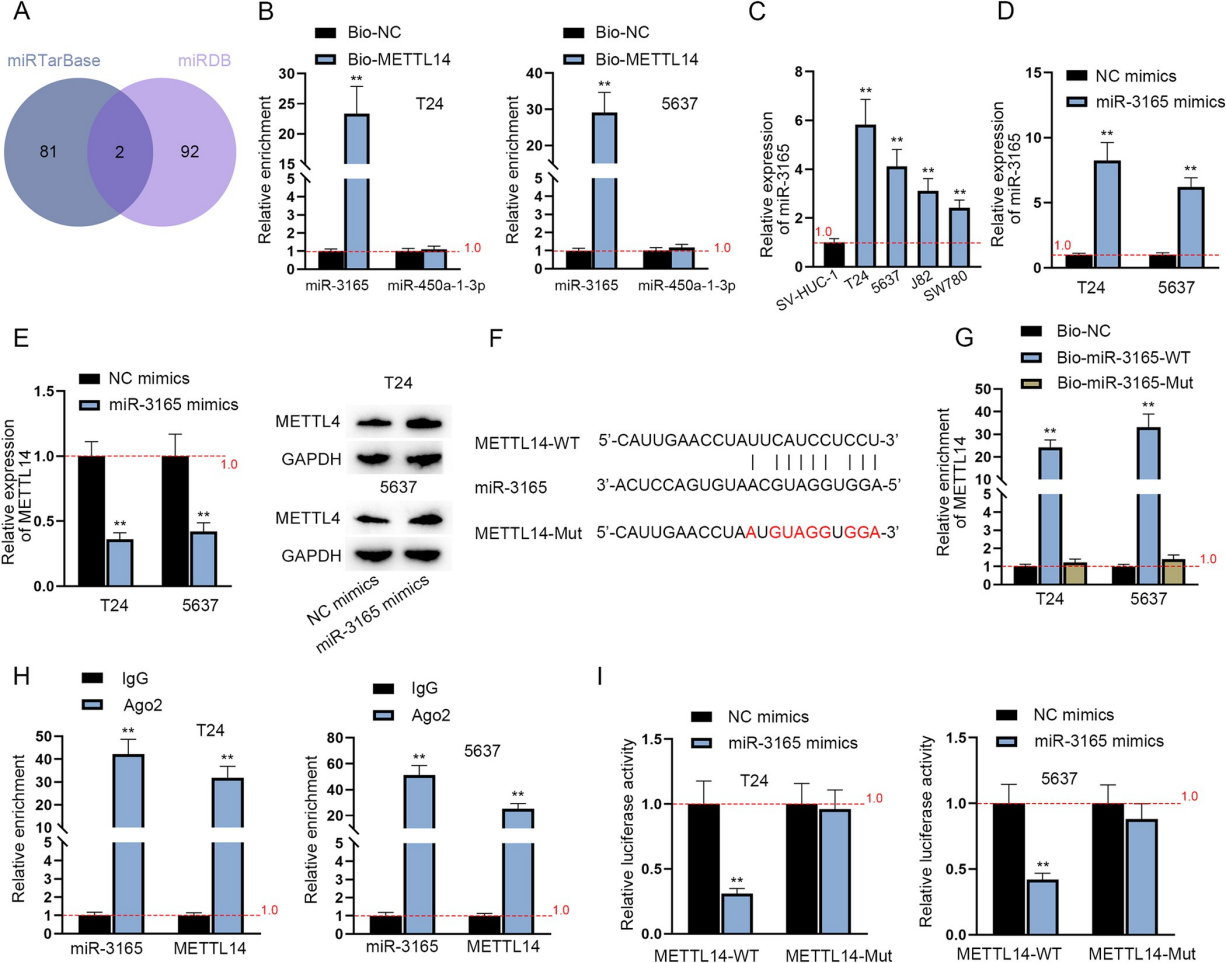
MiR-3165 regulates METTL14 as the upstream gene. A. 2 miRNAs were predicted to bind to METTL14 via miRTarBase (http://mirtarbase.cuhk.edu.cn/php/index.php) and miRDB (http://mirdb.org/). B. RIP assays were conducted to verify the interaction between METTL14 and miR-3165/miR-450a-1-3p in BCa cells. (n = 3) Each bar represents mean; error bars represent SD. P values were determined by two-way ANOVA followed by Tukey’s test. C. RT-qPCR was used to assess the relative expression of miR-3165 in SV-HUC-1 cell line and BCa cell lines. (n = 3) Each bar represents mean; error bars represent SD. P values were determined by one-way ANOVA followed by Dunnett’s test. D. RT-qPCR was used to determine the overexpression efficiency of miR-3165 mimics. (n = 3) Each bar represents mean; error bars represent SD. P values were determined by Student’s t test. E. The relative expression of METTL14 in miR-3165 mimics-transfected BCa cells was assessed through RT-qPCR (left panel). (n = 3) Each bar represents mean; error bars represent SD. P values were determined by Student’s t test. Western blot analysis of METTL14 protein level in miR-3165 mimics-transfected cells (right panels). F. The potential binding site between METTL14 and miR-3165 was predicted through miRBase database. G. RNA pulldown assay was performed to determine the combination between METTL14 and miR-3165 in BCa cells. (n = 3) Each bar represents mean; error bars represent SD. P values were determined by one-way ANOVA followed by Dunnett’s test. H. RIP assay was applied to verify the interaction between METTL14 and miR-3165 in BCa cells. (n = 3) Each bar represents mean; error bars represent SD. P values were determined by Student’s t test. I. Luciferase reporter assays were conducted to validate the combination between METTL14 and miR-3165 in BCa cells. (n = 3) Each bar represents mean; error bars represent SD. P values were determined by two-way ANOVA followed by Tukey’s test. **P < 0.01. See also S4 Data.

The downstream genes of METTL14 and their interactions in BCa cells have been investigated above. Therefore, the upstream gene regulating METTL14 was explored in the following assays. Given the fact that miRNAs are the common regulator of protein-coding genes, the upstream miRNA of METTL14 was next explored. Through the prediction of miRTarBase (http://mirtarbase.cuhk.edu.cn/php/index.php) and miRDB (http://mirdb.org/), two putative miRNAs were screened out as the upstream regulators of METTL14: miR-3165 and miR-450a-1-3p (Fig 6A). Then, RNA pulldown assays were applied to determine the actual regulator of METTL14. The results showed the distinct enrichment of miR-3165 in Bio-METTL14 groups versus Bio-NC groups (Fig 6B). Then, the upregulation of miR-3165 in BCa cell lines compared with SV-HUC-1 cell line was shown by RT-qPCR analysis (Fig 6C). In this way, miR-3165 was preliminarily regarded as the bona fide upstream regulator of METTL14 in BCa cells. Subsequently, the overexpression efficiency of miR-3165 mimics was verified (Fig 6D) and the expression of METTL14 in BCa cells was discovered to be silenced by miR-3165 overexpression according to the results of RT-qPCR and western blot (Fig 6E). Next, the putative biding site between miR-3165 and METTL14 was acquired through miRBase (http://www.mirbase.org/) database and the sequence of METTL14-Mut was also displayed (Fig 6F). The following RNA pulldown assay further proved the combination between miR-3165 and METTL14 since METTL14 was overtly abundant in Bio-miR-3165-WT relative to that in Bio-NC groups while Bio-miR-3165-Mut had no marked abundance of METTL14 (Fig 6G). RIP assay showed the significant enrichment of miR-3165 and METTL14 in Ago2 antibody bound precipitates (Fig 6H), manifesting the co-existence of miR-3165 and METTL14 in RNA-inducing silenced complexes (RISCs) for Ago2 is the essential component of RISCs [34]. Successively, luciferase reporter assays validated this interplay between miR-3165 and METTL14 in BCa cells via the putative binding site since the transfection of miR-3165 mimics obviously impaired the luciferase activity of METTL14-WT instead of METTL14-Mut (Fig 6I). Taken all together, miR-3165 negatively modulates METTL14 expression as an upstream regulator in BCa cells.

### MiR-3165 inhibits METTL14 expression to promote migration, invasion and EMT of BCa cells

**Fig 7:**
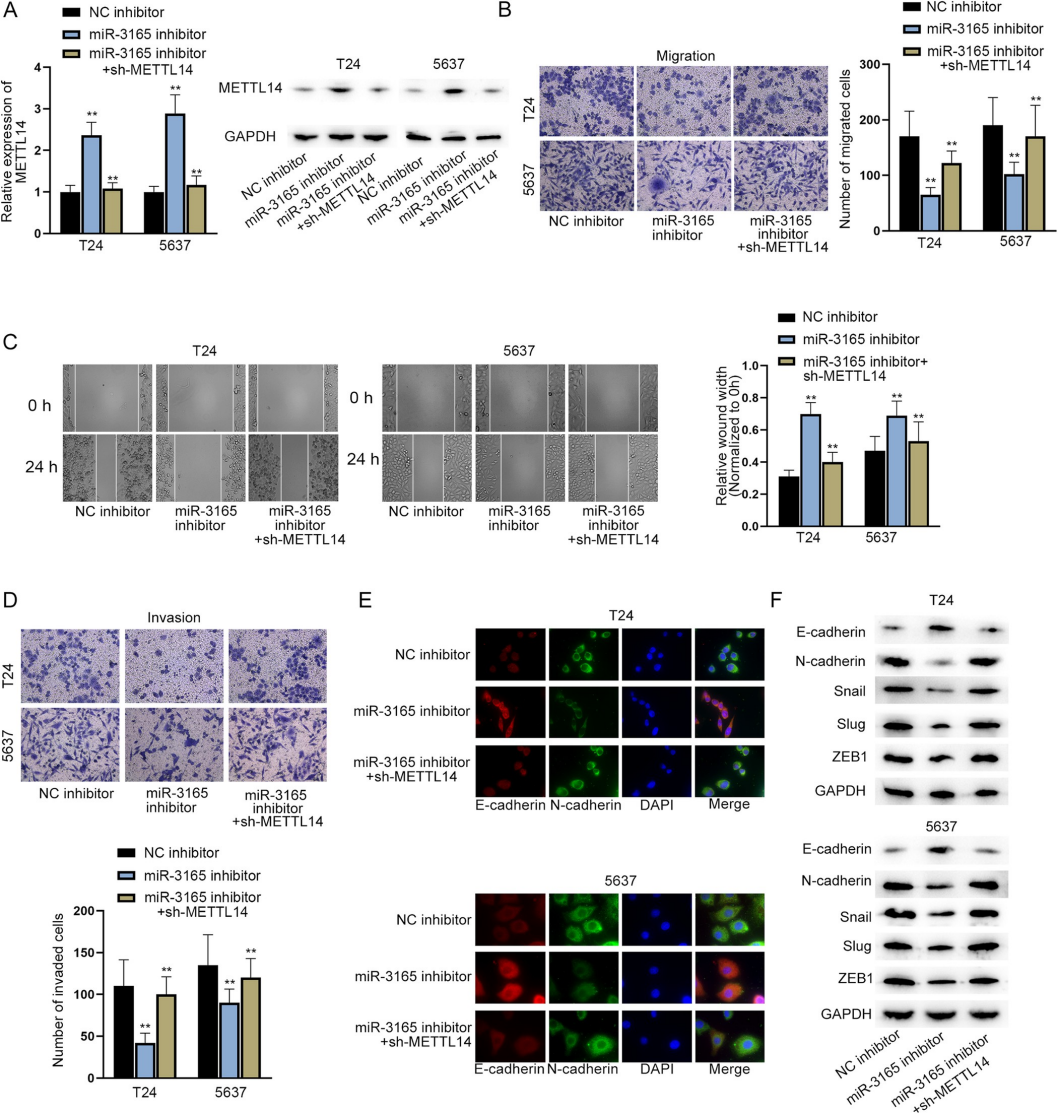
MiR-3165 inhibits METTL14 expression to promote migration, invasion and EMT of BCa cells. A. RT-qPCR was used to measure the relative expression of METTL14 after the down-regulation of miR-3165 with or without the subsequent knockdown of METTL14 in BCa cells (left panel). (n = 3) Each bar represents mean; error bars represent SD. P values were determined by one-way ANOVA followed by Dunnett’s test. Western blot analysis of METTL14 protein levels in BCa cells with different transfections (right panels). B-C. Transwell (migration) and wound healing assays were conducted to measure the migration of BCa cells after the indicated transfection. (n = 3) Each bar represents mean; error bars represent SD. P values were determined by one-way ANOVA followed by Dunnett’s test. D. Transwell (invasion) assay was conducted to measure the invasion of BCa cells after the indicated transfection. (n = 3) Each bar represents mean; error bars represent SD. P values were determined by one-way ANOVA followed by Dunnett’s test. E. IF assay was performed to assess levels of EMT-related proteins in BCa cells after the indicated transfection. F. Western blot analysis of EMT-related proteins in BCa cells transfected with the indicated plasmids. **P < 0.01. See also S3–S7 Data files.

To clarify whether miR-3165 regulates METTL14 expression to function in BCa cells, we designed and performed functional rescue assays. According to the RT-qPCR and western blot analyses, the transfection of sh-METTL14 plasmids successfully reduced the increased expression of METTL14 caused by the knockdown of miR-3165 (Fig 7A). Then, a series of functional assays, including Transwell assays, wound healing assays and IF and western blot analyses of EMT-related proteins, exhibited that knockdown of METTL14 countervailed the suppressive effect of miR-3165 inhibition on BCa cell migration, invasion and EMT (Fig 7B–7F). Additionally, CCK-8 assay indicated that METTL14 deficiency reversed the inhibition of miR-3165 inhibitor on BCa cell growth (S3B Fig). Thus, it was deduced that miR-3165 inhibits METTL14 expression to promote the migration, invasion and EMT of BCa cells.

## Discussion

Being frequently diagnosed in genitourinary tract, BCa is the one of the most common solid tumor around the world [35]. The molecular mechanism for BCa is worth of great efforts to be explored.

This study focuses on the impact of METTL14 and its related genes on BCa cell biological behaviors and tumor metastasis. This study verified that METTL14 expression is down-regulated in BCa cells lines, prohibiting the migration, invasion and EMT of BCa cells, as well as tumor metastasis. In previous study, METTL14 has been discovered to play a role as a tumor suppressor in various cancers, such as colorectal cancer [36] and kidney renal clear cell carcinoma [37]. At the same time, the oncogenic role of METTL14 has been also uncovered in pancreatic cancer [16], breast cancer [38] and so on. Therefore, this study is an important supplement to the research on METTL14 and its suppressive function in BCa. This finding regarding METTL14 role in BCa is consistent with that reported in the previous study [17]. Differently, the previous study focused on the inhibitory role of METTL14 in BCa tumor initiating capacity of bladder tumor initiating cells, sphere formation, invasion and tumor propagation while ours uncovered its suppression on BCa cell migration, invasion and EMT as well as tumor metastasis. As for the regulation mechanism, m6A function was both investigated in two research studies but subsequent investigation was conducted in different ways. The study of Gu et al. probed into the downstream mechanism of METTL14 in terms of target genes of major signaling pathways while ours investigated it from the aspect of METTL14 similar genes and m6A readers.

Moreover, USP38 was discovered as a downstream gene mediated by METTL14 through m6A modification, which was participated by YTHDF2, a well-known m6A reader. Previous study has revealed that METTL14 exerts prohibitive function in cancers through m6A modification. For instance, METTL14 mediates the m6A-dependent processing to slacken the metastatic potential of hepatocellular carcinoma [39]. In this study, METTL14 was elucidated as an upstream regulator positively modulating USP38 and enhancing the stability of USP38 mRNA by the YTHDF2-dependent m6A modification. Interestingly, the YTHDF2-dependent pathway relied on by METTL14-related regulation has been discovered previously in colorectal cancer [40]. In this way, the finding in this current study further validates the cooperation of METTL14 and YTHDF2 in affecting human cancers. Furthermore, we found a feedback loop within METTL14 and USP38 mRNA through the result that the two genes positively altered the expression of each other and they were co-expressed in BCa cells. Furthermore, the stability of the two genes was dependent on each other according to the mRNA stability assay and western blot analysis along with CHX or MG132 treatment. Previous studies have reported that METTL14 forms a negative feedback loop with ALKBH5/TFEB in cardiomyocytes treated with hypoxia/reoxygenation [41] and constitutes a positive one with ALKBH5/HuR in breast cancer [42]. Strikingly, this is the first study on the METTL14-USP38 positive feedback loop in BCa cells. Besides, miR-3165 was verified as the upstream regulator of METTL14 and found to negatively modulate the expression of METTL14, facilitating the progression of BCa cells. Moreover, the inhibition of METTL14 rescued the inhibited BCa cell progression after miR-3165 silencing. In previous study, downstream miRNAs regulated by METTL14 were discovered, such as miR-375 [15], miR-19a [43] and miR-146a-5p [38]. Different from these downstream miRNAs, miR-3165 was determined as the regulator of METTL14, functioning through METTL14. Hence, the vacancy of upstream miRNAs of METTL14 was filled by this study. Given that the potential of miRNAs as therapeutic targets [44], targeting miR-3165 might be a potential therapeutic strategy in BCa treatment since it causes a subsequent decrease in METTL14 expression, further leading to the promotion of cell migration, invasion and EMT as well as tumor metastasis in BCa.

However, there are some limitations to be addressed in the future explorations. The pathological features of patients will be collected and analyzed to provide more penetrative discussion about BCa. Considering the result that miR-3165 and METTL14 co-exist in RISCs, we wonder whether the two genes are involved in ceRNA mechanism. Hence, the ceRNA mechanism in BCa cells would also be explored in the future investigation.

Taken all together, METTL14 forms a feedback loop dependent on YTHDF2 with USP38 in BCa (S4 Fig). In addition, miR-3165 facilitates BC cell progression and tumor metastasis via down-regulating tumor suppressor METTL14. This study provides novel insights into the cellular mechanism of METTL14 in BCa cells for BCa treatment. Nonetheless, the influence of the feedback loop of METTL14 and USP38 and the function of miR-3165 has not been verified through *in vivo* assays, which will be conducted in the future study.

## Materials and methods

### Ethics statement

The animal study in this study was approved by the Ethics Committee of Jiangxi Cancer Hospital (0029-2100D06B6101).

### Cell lines and cell culture

Human normal urothelial cell line SV-HUC-1 from ATCC (Washington, DC, USA) was cultured in DMEM (#11885–076, Gibco, Grand Island, NY, USA) containing 10% FBS (fetal bovine serum; #11885–076, Gibco), 100U/mL penicillin and 100μg/mL streptomycin sulfate at 37°C in 5% CO_2_. BCa cell lines (T24, 5637, J82, and SW780) were procured from the Cell Bank of Type Culture Collection, Chinese Academy of Science (Shanghai, China) and cultured in RPMI-1640 (#11875–093, Gibco) with 10% FBS, 100 U/mL penicillin and 100μg/mL streptomycin at 37°C in 5% CO_2_.

### Vector construction and cell transfection

To knock down YTHDF2, USP38 and METTL14, short hairpin RNAs (shRNAs) targeting YTHDF2 (sh-YTHDF2), USP38 (sh-USP38), and METTL14 (sh-METTL14) were chemically synthesized. Overexpression vectors including pcDNA3.1-YTHDF2, pcDNA3.1-METTL14, and pcDNA3.1-USP38 were generated by inserting full length of YTHDF2 sequence, METTL14 sequence, or USP38 sequence into pcDNA3.1 vector (V79520, Invitrogen, Carlsbad, CA, USA) individually. For luciferase reporter assays, wild-type or mutant sequence of METTL14 (METTL14-WT or METTL14-Mut) was subcloned into pmirGLO luciferase vector (E1330, Promega, Madison, WI, USA) to form luciferase reporter constructs.

### RT-qPCR (quantitative real-time PCR)

Total RNAs were extracted using TRIzol reagent (#15596–018, Thermo Fisher Scientific, Waltham, MA, USA). PrimeScript RT reagent Kit with gDNA Eraser (RR047A, Takara, Dalian, China) was used to conduct reverse transcription and cDNA (complementary DNA) synthesis following the supplier’s protocols. PCR was implemented on the Real-time PCR System (ABI7500, Applied Biosystems, Foster City, CA, USA) utilizing SYBR-Green Master Mix (#4364346, Applied Biosystems). The results were calculated with 2^-ΔΔCt^ method [45].

### Western blot

Total proteins were extracted from BCa cells using RIPA buffer, subjected to SDS-PAGE and transferred onto PVDF membranes. The membranes were blocked in TBST containing 5% skimmed milk for 1 h. Then, the membranes were incubated with primary antibody against METTL14 (anti-METTL14, ab252562, Abcam, Cambridge, MA, USA), MMP2 (anti-MMP2, ab92536, Abcam), MMP9 (anti-MMP9, ab76003, Abcam), E-cadherin (anti-E-cadherin, ab40772, Abcam), N-cadherin (anti-N-cadherin, ab76011, Abcam), Snail (anti-Snail, ab216347, Abcam), Slug (anti-Slug, ab27568, Abcam), ZEB1 (anti-ZEB1, ab203829, Abcam), p-PI3K (anti-p-PI3K, ab278545, Abcam), PI3K (anti-PI3K, ab278545, Abcam), p-AKT (anti-p-AKT, ab8805, Abcam), AKT (anti-AKT, ab38449, Abcam), p-mTOR (anti-p-mTOR, ab109268, Abcam), mTOR (anti-mTOR, ab134903, Abcam), USP38 (anti-USP38, ab72244, Abcam) or YTHDF2 (anti-YTHDF2, ab220163, Abcam) at 4°C overnight. After TBST washing (4 times, 5 min per time), the membranes were incubated with secondary antibodies on a shaker for 1 h, followed by TBST washing (5 times, 5 min per time). The protein levels were detected using enhanced chemiluminescence (ECL) reagent. GAPDH (anti-GAPDH, ab9485/ab8245, Abcam) was used as an internal reference.

### Analysis of RNA stability

Actinomycin D (ActD; SBR00013, Sigma-Aldrich, St. Louis, MI, USA) was added to interfere with RNA transcription. RNA level was detected via RT-qPCR at different time points (0, 4, and 8 h).

### CHX (cycloheximide) and MG132 treatment

CHX (AC357420010; ACROS Organics, Shanghai, China) was used to inhibit protein synthesis. The indicated BCa cells were treated with CHX for 0, 6, and 12 h. The proteasomal inhibitor MG132 (M7449, Sigma-Aldrich) was used to inhibit protein degradation. The level of METTL14 was analyzed via western blot.

### IF (immunofluorescence) staining

Briefly, the transfected cells were incubated with primary antibodies against E-cadherin (anti-E-cadherin, #3195, Cell signaling Technology, Boston, MA, USA), N-cadherin (anti-N-cadherin, #13116, Cell signaling Technology) at 4°C overnight, followed by PBS washing and incubation with secondary antibodies. The nuclei were counterstained by DAPI. Lastly, images were photographed under a fluorescence microscope. Each experiment was repeated three times and three replicates for each bio-repeat.

### Wound healing assay

Transfected BCa cells were seeded in 6-well plates. The wound was scratched using a pipette tip. Subsequently, cells were incubated at 37°C in 5% CO_2_. 24 h later, images were acquired under an optical microscope. Results were analyzed via ImageJ software. Each experiment was repeated three times and three replicates for each bio-repeat.

### Transwell migration and invasion assays

Transfected BCa cells were seeded in 96-well chamber (CLS3374; Corning, Cambridge, MA, USA). The upper chambers were supplemented with serum-free medium and the lower ones were with complete medium. For invasion assays, the upper chambers were precoated with Matrigel (#356234; BD Biosciences, San Jose, CA, USA) particularly. After 24 h of incubation, the chambers were fixed in methanol and then stained by crystal violet. The migrated or invaded cells were photographed under a light microscope. Each experiment was repeated three times and three replicates for each bio-repeat.

### CCK-8 (cell counting kit-8)

CCK-8 assay was utilized to determine the proliferative ability of BCa cells as per the supplier’s instructions. The transfected cells were seeded into 96-well plates and tested at 450 nm with a microplate reader.

### *In vivo* metastatic experiment

BALB/c nude mice were purchased from the Slac Laboratories (Shanghai, China) and randomly divided into two groups. The xenograft mouse model was generated via tail-vein injection of METTL14-expressing T24 cells (5 × 10^5^ cells per mouse) into experimental mice while those injected with pcDNA3.1 empty vector transfected cells as control. Four weeks later, the mice were sacrificed and tumors were excised to be weighed.

To produce experimental lung metastasis, the stably transfected T24 cells were injected into the lateral tail veins of mice (five mice per group). 3 weeks later, all the mice were killed under anesthesia. The lungs and livers were collected for H&E (hematoxylin and eosin) staining. For H&E staining, tissue samples from mice were fixed in 10% formalin and paraffin embedded. 5μm sections were made and stained with H&E Staining Kit (C0105S; Beyotime, Beijing, China). The stained sections were imaged and liver/lung metastatic nodules were counted under an optical microscope.

### IHC (immunohistochemistry)

Paraffin sections of xenograft tumor tissue were dewaxed with xylene and dehydrated with gradient alcohol. Antigen repair was performed with 0.01 mol/L citrate buffer solution. 3% hydrogen peroxide was used to block endogenous peroxidase. After cooling naturally at room temperature, the paraffin sections were washed with 0.1 mol/L PBS and blocked with 5% BSA. Then, the paraffin sections were incubated overnight with rabbit anti-MELLT14 (1:100). Subsequently, the paraffin sections were incubated with HRP-labeled goat anti-rabbit secondary antibody. The proteins were detected at room temperature using a DAB immunohistochemistry color development kit (Boshide Biological, Wuhan, China).

### RIP (RNA-binding protein immunoprecipitation) and Me-RIP (methylated RNA immunoprecipitation) assays

For RIP assay, 6 × 10^7^ BCa cells were lysed and the lysates (300 μL) were divided into three group: IgG (100 μL), YTHDF2/Ago2 (100 μL) and Input. 5 μg anti-IgG (CS200621, Millipore, Billerica, MA, USA), anti-YTHDF2 or anti-Ago2 (#2897, Cell signaling Technology, Boston, MA, USA) were incubated with 50μg Protein A/G Agarose for the whole night at 4°C. Then, cell lysates in different groups were cultured with the complexes comprising antibodies and Agarose beads overnight. Finally, when the precipitation was fully finished, RNAs were extracted from the washed RNA-antibody-Agarose beads complexes for RT-qPCR analysis. For Me-RIP assay, anti-m6A (#202003, Synaptic Systems, Goettingen, Germany) was used in the experimental group.

### Co-IP (co-immunoprecipitation) and IP (immunoprecipitation) assays

After being extracted from BCa cells, total proteins were cultivated with anti-ubiquitin (Cat No. 10201-2-AP, Proteintech, Chicago, IL, USA), anti-METTL14, anti-USP38 (ab72244, Abcam) and anti-IgG conjugated with Protein A/G Agarose beads beforehand. When the precipitation of antibody-bead-protein complexes was fully finished, proteins were purified from the precipitates and assessed by western blot analysis.

### Dual luciferase reporter assay

Dual Luciferase Report Assay System (E1910; Promega, Madison, WI, USA) was used under the instruction of the manufacturer. METTL14-WT or METTL14-Mut reporter vector was co-transfected into BCa cells with NC mimics or miR-3165 mimics. After the culture for 36 h, the luciferase activity was observed under a fluorescence microscope and analyzed. Renilla luciferase served as the internal reference.

### RNA pulldown assays

The biotinylated (Bio-) plasmids (Bio-NC, Bio-METTL14, Bio-miR-3165-WT and Bio-miR-3165-Mut) in this study were all chemically synthesized by RiboBio (Guangzhou, China). 1 × 10^7^ BCa cells were lysed and the cell lysate was meanly divided into Input, Bio-NC and Bio-METTL14/Bio-miR-3165-WT/Bio-miR-3165-Mut. Bio-NC and Bio-RNAs conjugated with Streptavidin beads at 4°C for 2 h were mixed with cell lysates respectively. After the culture at 4°C overnight, complexes comprising beads and RNAs were centrifuged shortly and RNAs were extracted from the precipitates via TRIzol for RT-qPCR.

### Statistical analysis

The data were shown as mean ± SD. Statistical analysis was performed using Student’s t test or one-way/two-way ANOVA (analysis of variance) followed by post hoc test (Dunnett or Tukey) to conduct difference comparison between or among groups with the application of SPSS 22.0. P value < 0.05 indicated statistical difference.

## Supporting information

S1 Fig A-B. Transwell (migration) and wound healing assays were conducted to determine the effect of METTL14 deficiency on cell migration. (n = 3) Each bar represents mean; error bars represent SD. P values were determined by Student’s t test. C. Transwell (invasion) assay was carried out to evaluate the invasive capacity of METTL14-deficient cells. (n = 3) Each bar represents mean; error bars represent SD. P values were determined by Student’s t test. D. Representative images of IF staining of EMT markers in METTL14-deficient cells. **P < 0.01. See also S5 Data.
(TIF)

S2 Fig A-B. The overexpression efficiency of overexpression plasmids of different genes (YTHDC1, IGF2BP1, IGF2BP2, IGF2BP3, YTHDF1, YTHDF2 and YTHDF3) was determined and the relative level of USP38 mRNA was measured through RT-qPCR after the overexpression of those genes. (n = 3) Each bar represents mean; error bars represent SD. P values were determined by Student’s t test (for left panels) or one-way ANOVA followed by Tukey’s test (for rights panels). C. GEPIA correlation analysis of USP38 and YTHDF2 in BLCA tissues was shown. **P < 0.01. (TIF)

S3 Fig A-B. CCK-8 assay was performed to assess the proliferative ability of BCa cells after the indicated transfection. **P < 0.01 was determined by one-way ANOVA followed by Dunnett’s test. n = 3. Each point with an error bar represents mean ± SD.
(TIF)

S4 Fig The schema drawn by the author shows that the feedback loop between METTL14 and USP38 regulates cell migration, invasion and EMT as well as metastasis in BCa. (TIF)

S1 Data Original microscopy images in Fig 1. (ZIP)

S2 Data Original microscopy images in Fig 4. (ZIP)

S3 Data Original microscopy images in Fig 7. (ZIP)

S4 Data Original microscopy images in Fig 7. (ZIP)

S5 Data Original microscopy images in Fig 7. (ZIP)

S6 Data Original microscopy images in Fig 7. (ZIP)

S7 Data Original western blots. (ZIP)

S8 Data Original microscopy images in S1 Fig. (ZIP)

S1 Text Abbreviations in the present work. (DOCX)
