# Supplementary figures and images for: The feedback loop of METTL14 and USP38 regulates cell migration, invasion and EMT as well as metastasis in bladder cancer (part 1 of 2)
Source: PLoS Genet. 2022 Oct 26;18(10):e1010366. doi: 10.1371/journal.pgen.1010366 (PMC9605029; doi:10.1371/journal.pgen.1010366)

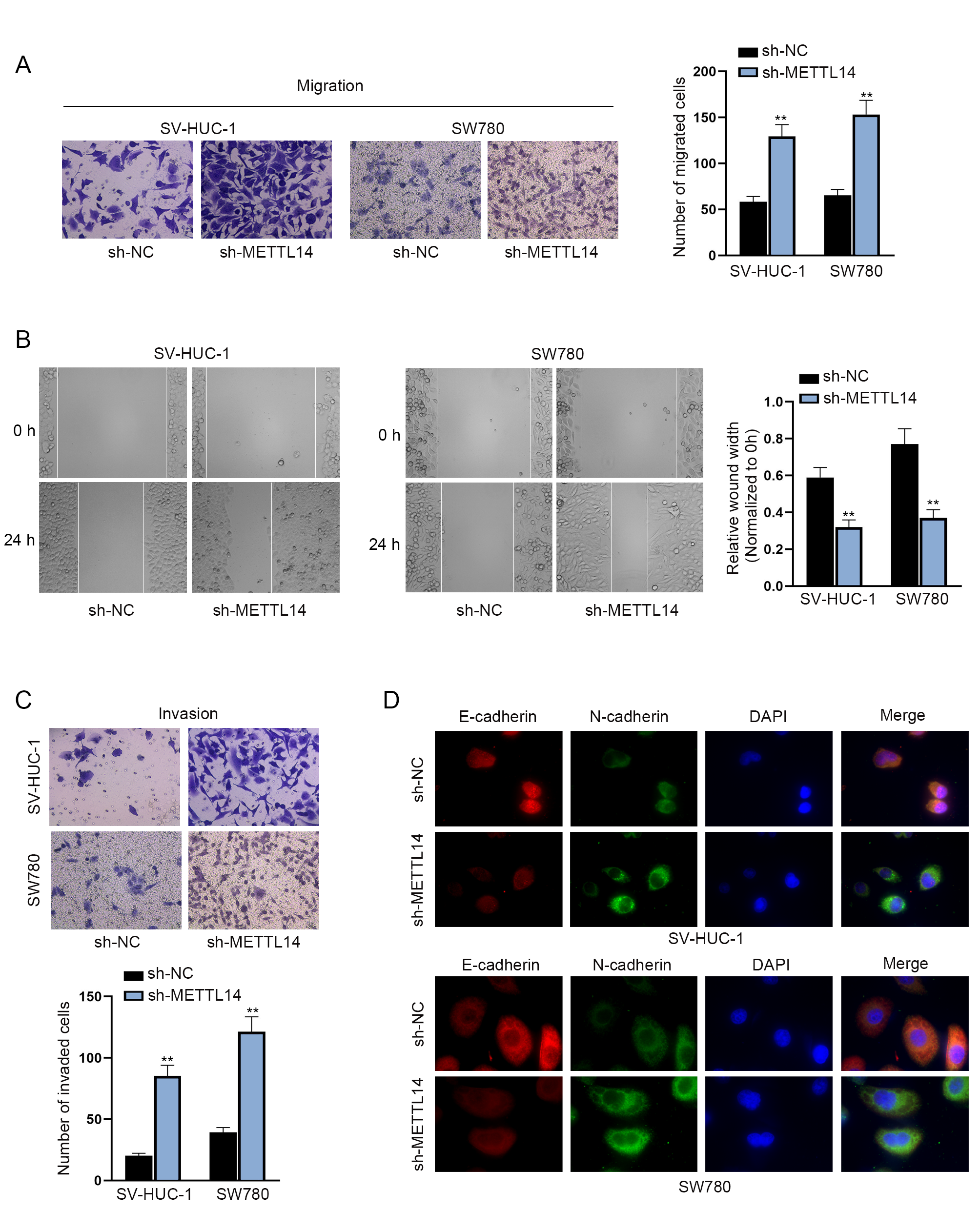

Supplement: S1 Fig — A-B. Transwell (migration) and wound healing assays were conducted to determine the effect of METTL14 deficiency on cell migration. (n = 3) Each bar represents mean; error bars represent SD. P values were determined by Student’s t test. C. Transwell (invasion) assay was carried out to evaluate the invasive capacity of METTL14-deficient cells. (n = 3) Each bar represents mean; error bars represent SD. P values were determined by Student’s t test. D. Representative images of IF staining of EMT markers in METTL14-deficient cells. **P < 0.01. See also S5 Data. (TIF) [file pgen.1010366.s001.tif]

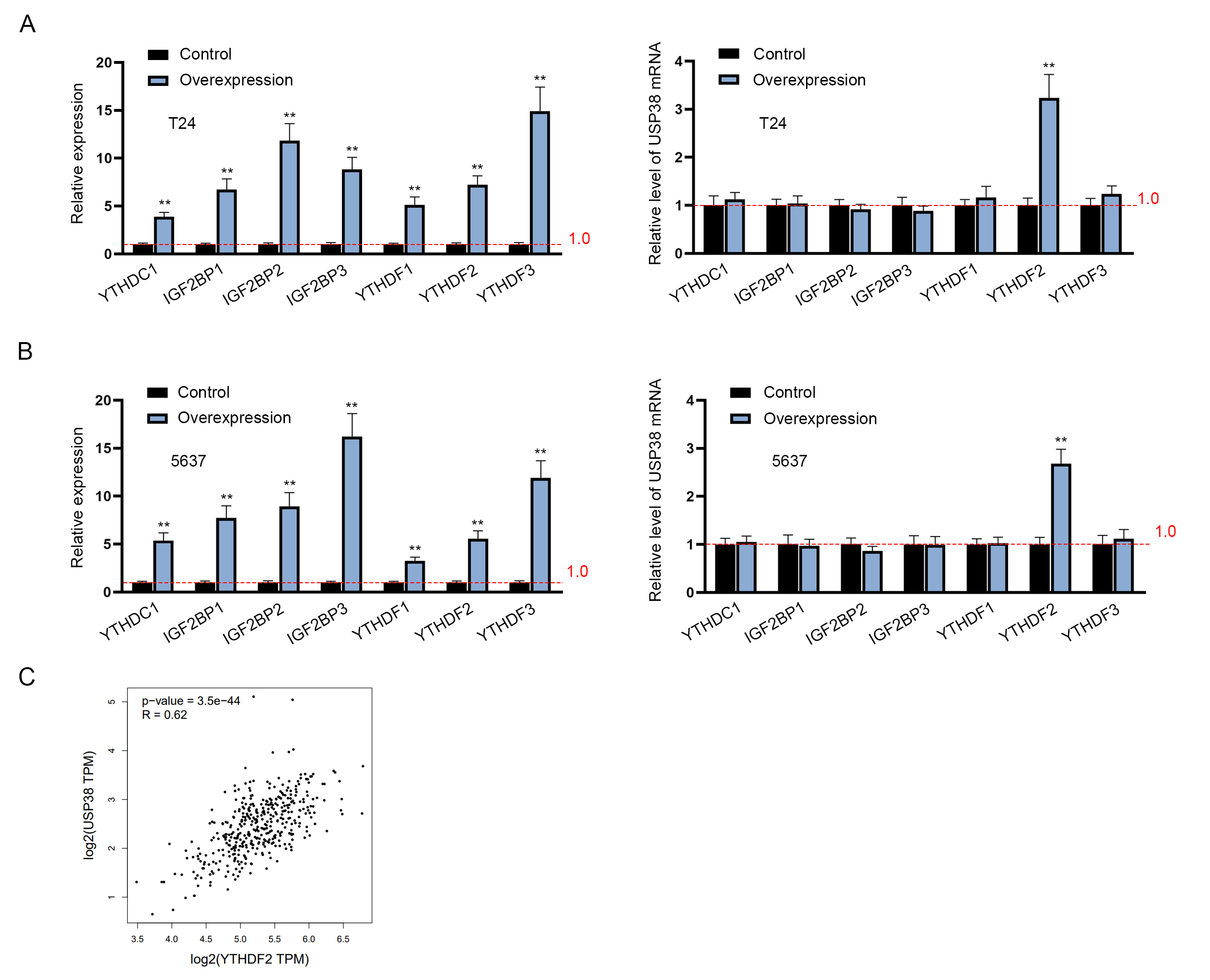

Supplement: S2 Fig — (TIF) [file pgen.1010366.s002.tif]

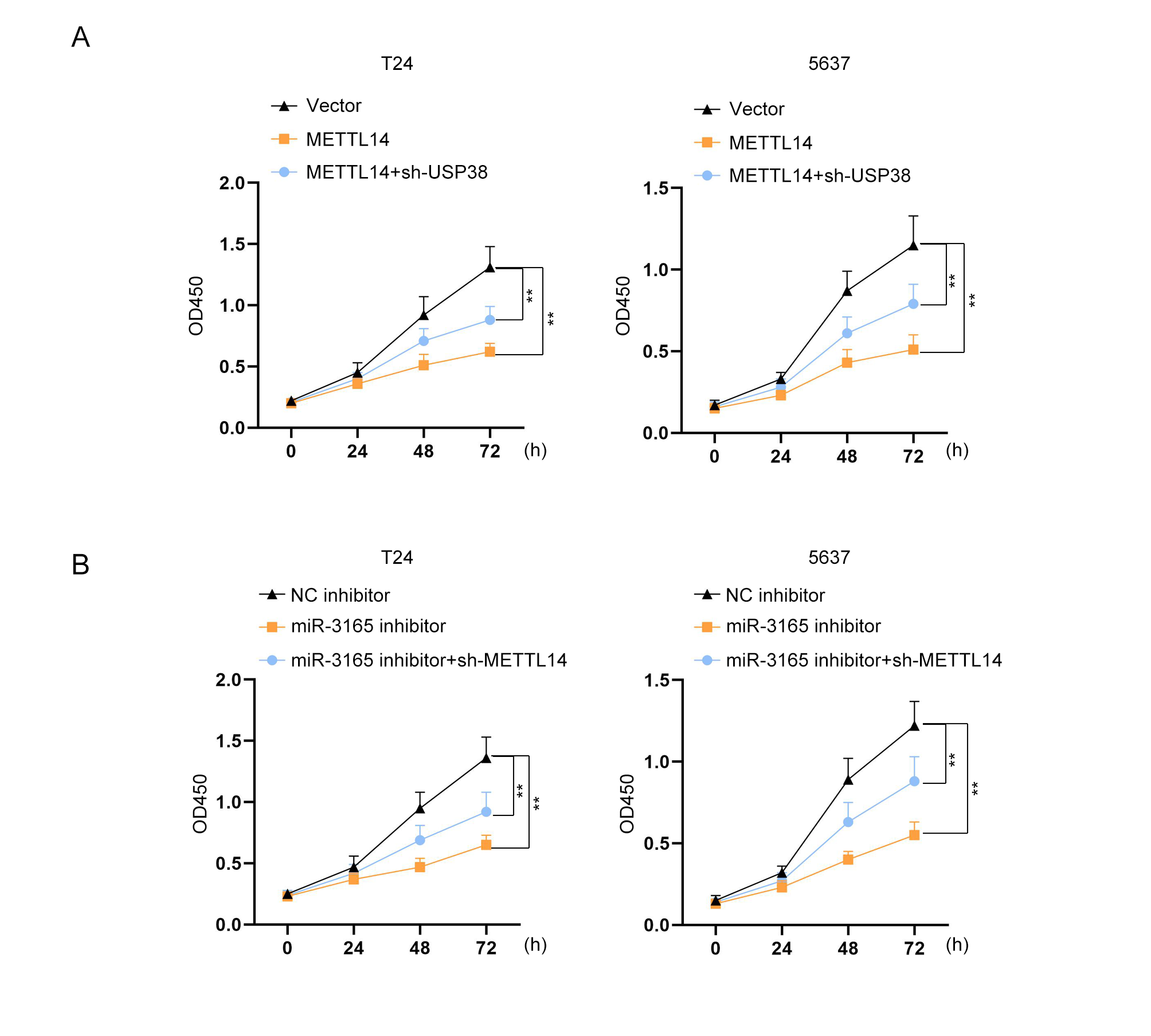

Supplement: S3 Fig — A-B. CCK-8 assay was performed to assess the proliferative ability of BCa cells after the indicated transfection. **P < 0.01 was determined by one-way ANOVA followed by Dunnett’s test. n = 3. Each point with an error bar represents mean ± SD. (TIF) [file pgen.1010366.s003.tif]

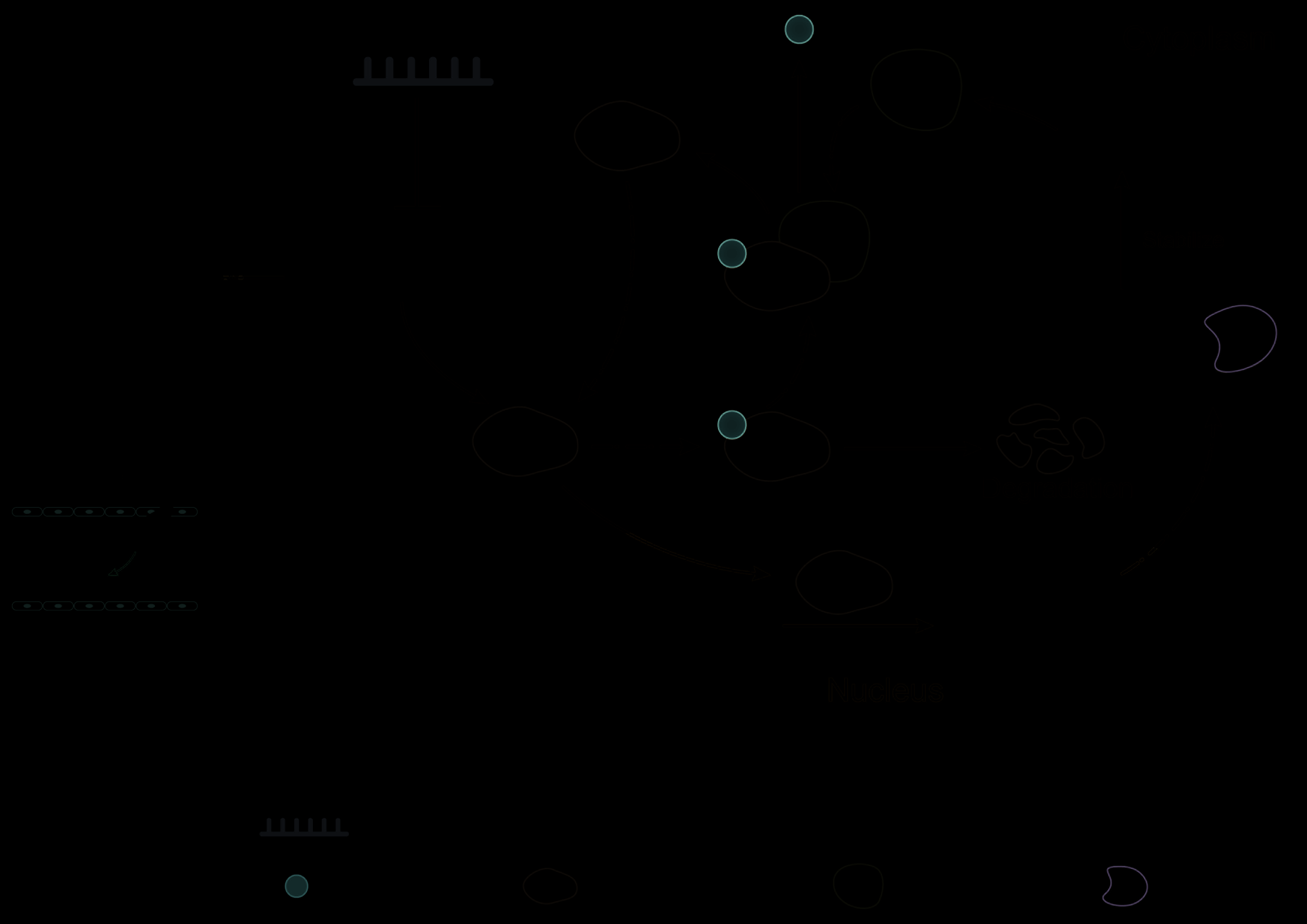

Supplement: S4 Fig — (TIF) [file pgen.1010366.s004.tif]

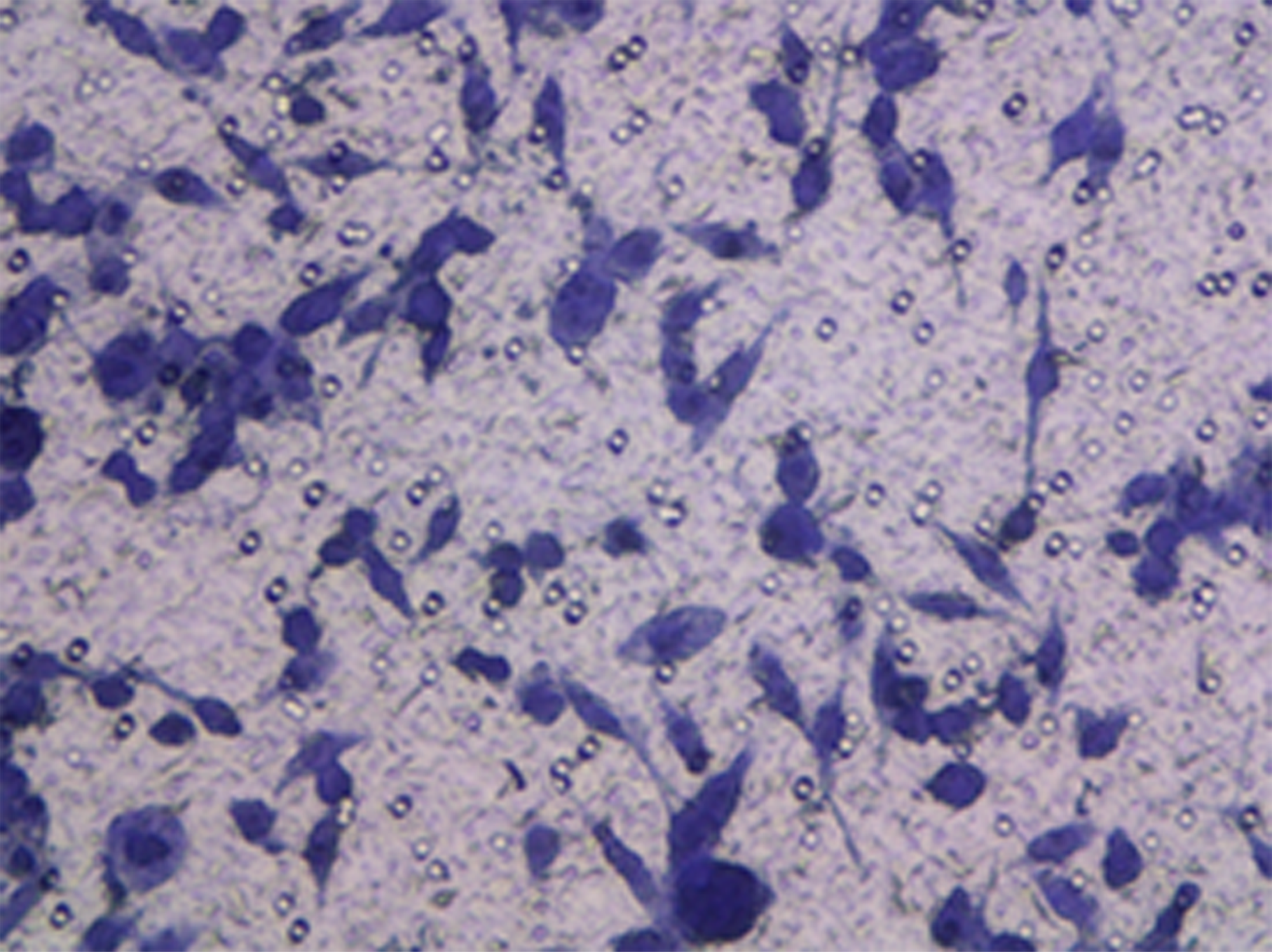

Supplement: S1 Data — (ZIP) [file pgen.1010366.s005.zip › 1C Migration 5637 METTL14.png]

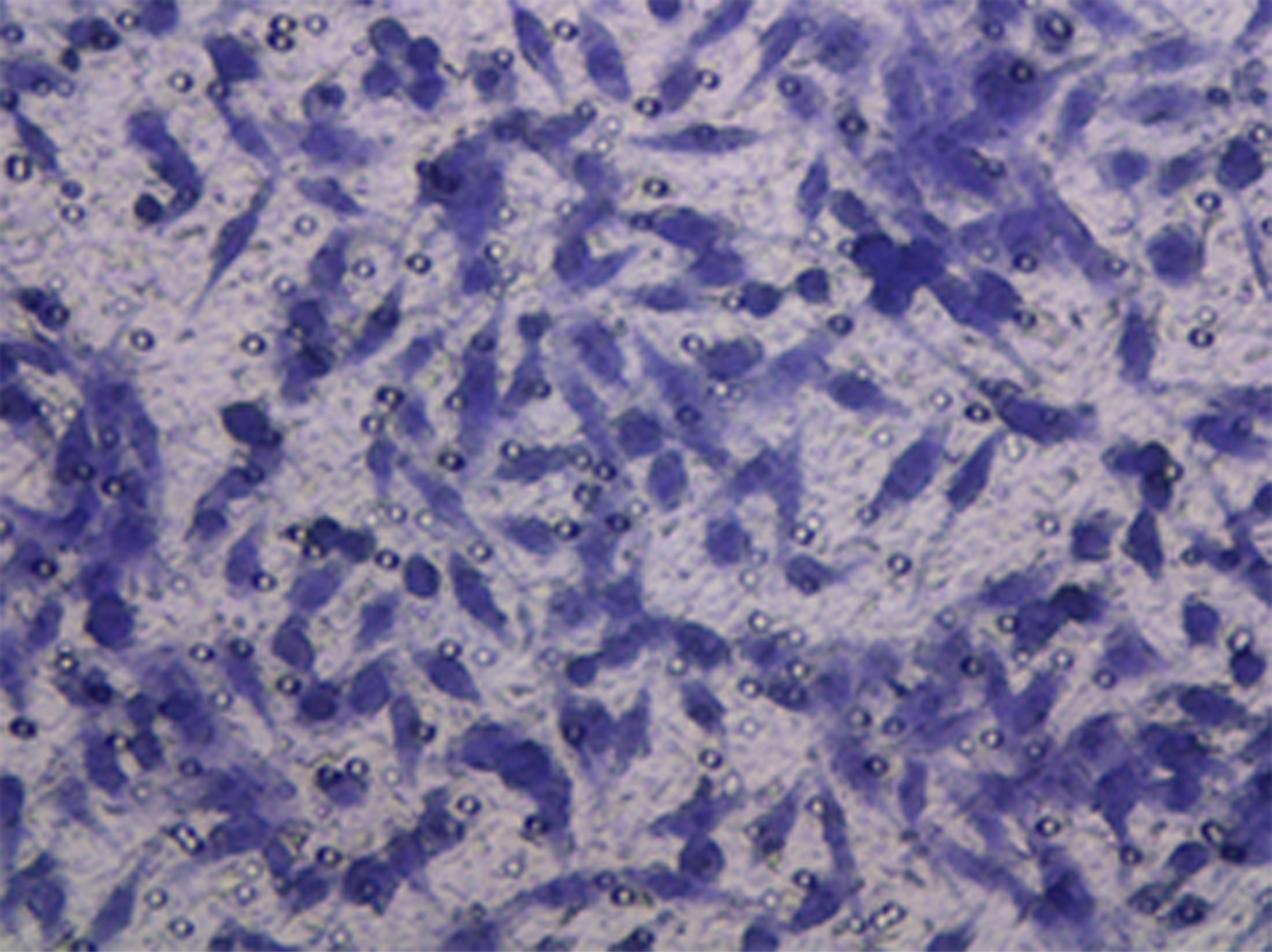

Supplement: S1 Data — (ZIP) [file pgen.1010366.s005.zip › 1C Migration 5637 Vector.png]

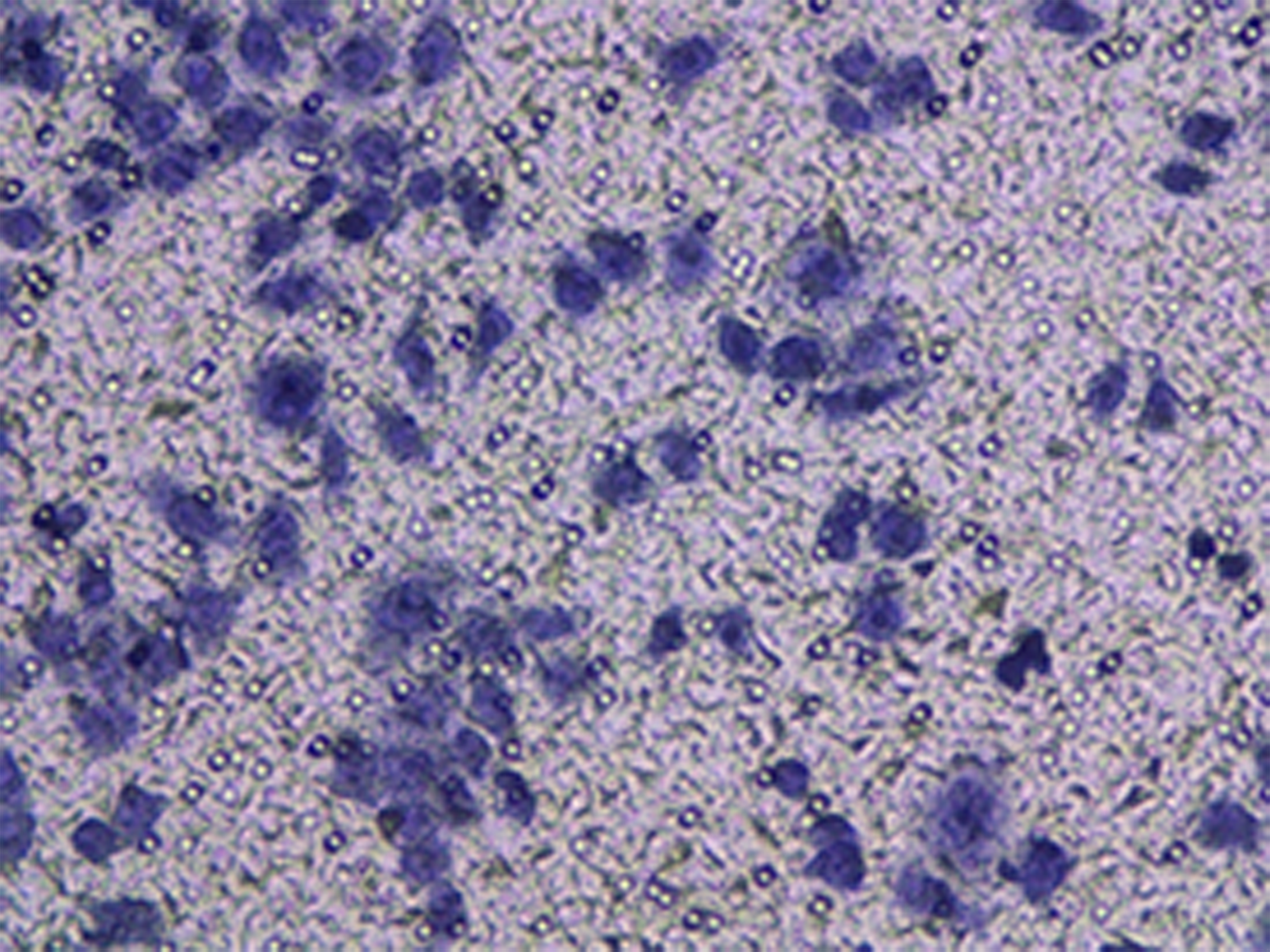

Supplement: S1 Data — (ZIP) [file pgen.1010366.s005.zip › 1C Migration T24 METTL14.png]

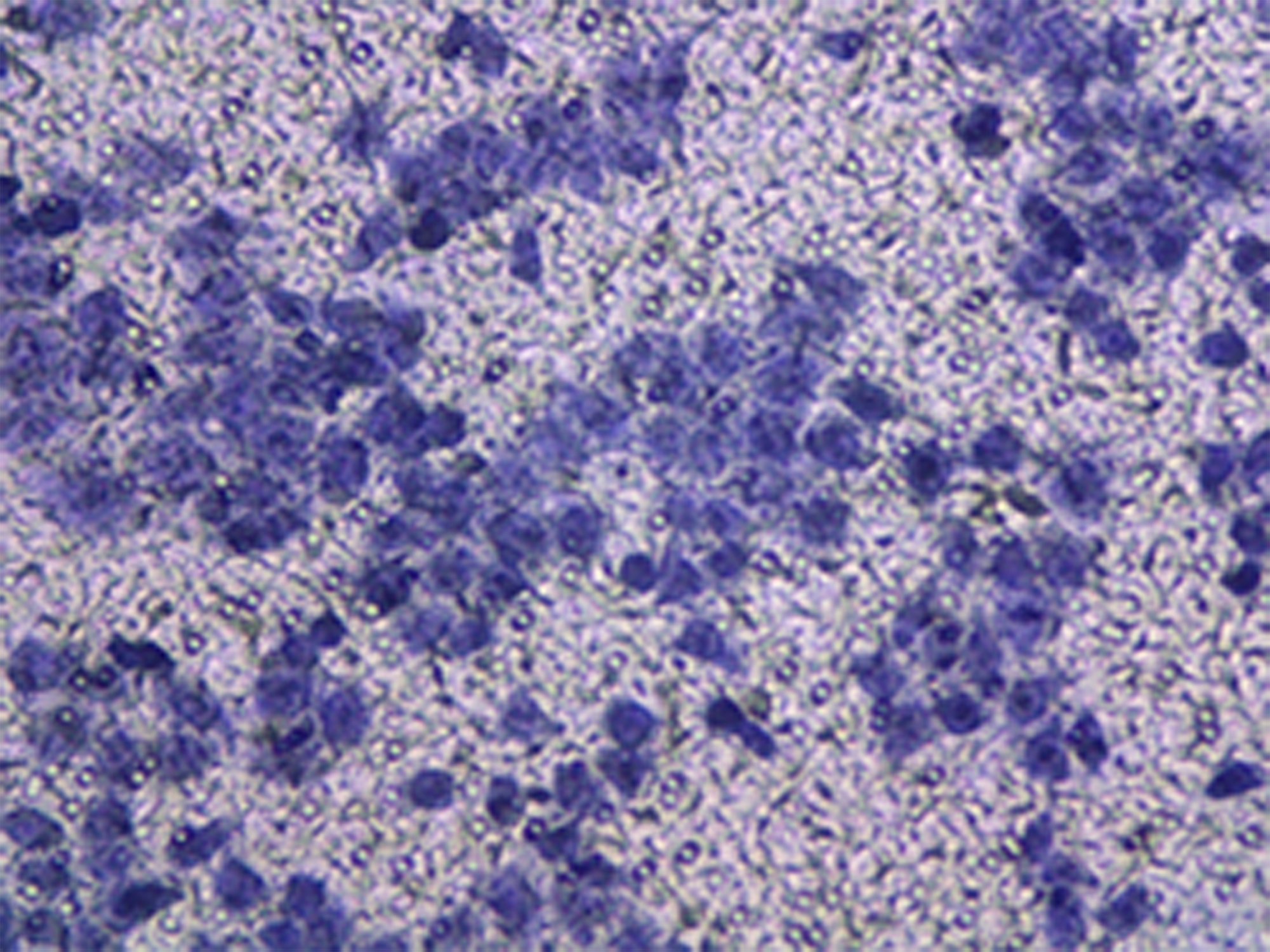

Supplement: S1 Data — (ZIP) [file pgen.1010366.s005.zip › 1C Migration T24 Vector.png]

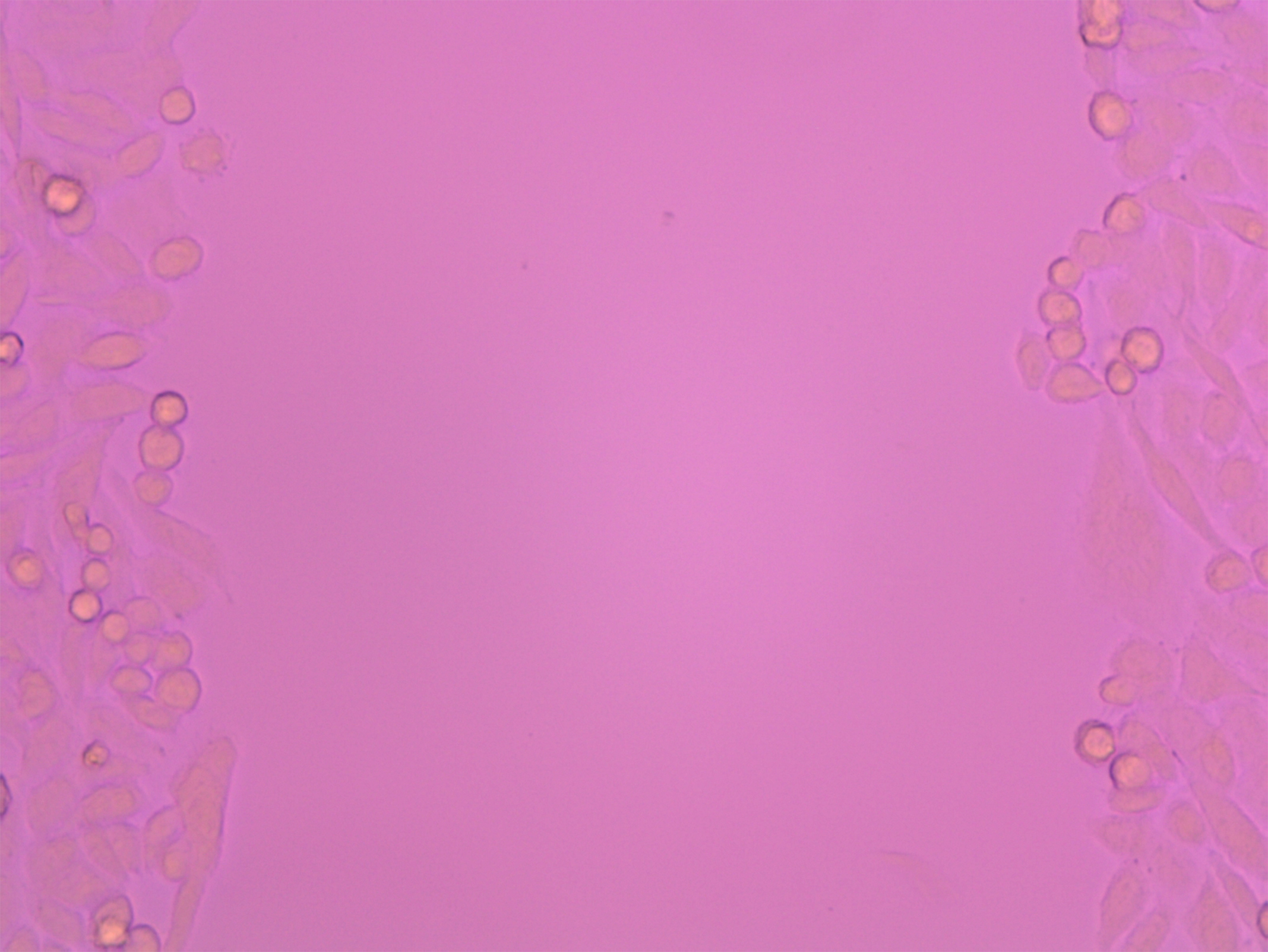

Supplement: S1 Data — (ZIP) [file pgen.1010366.s005.zip › 1D 5637 0h METTL14.png]

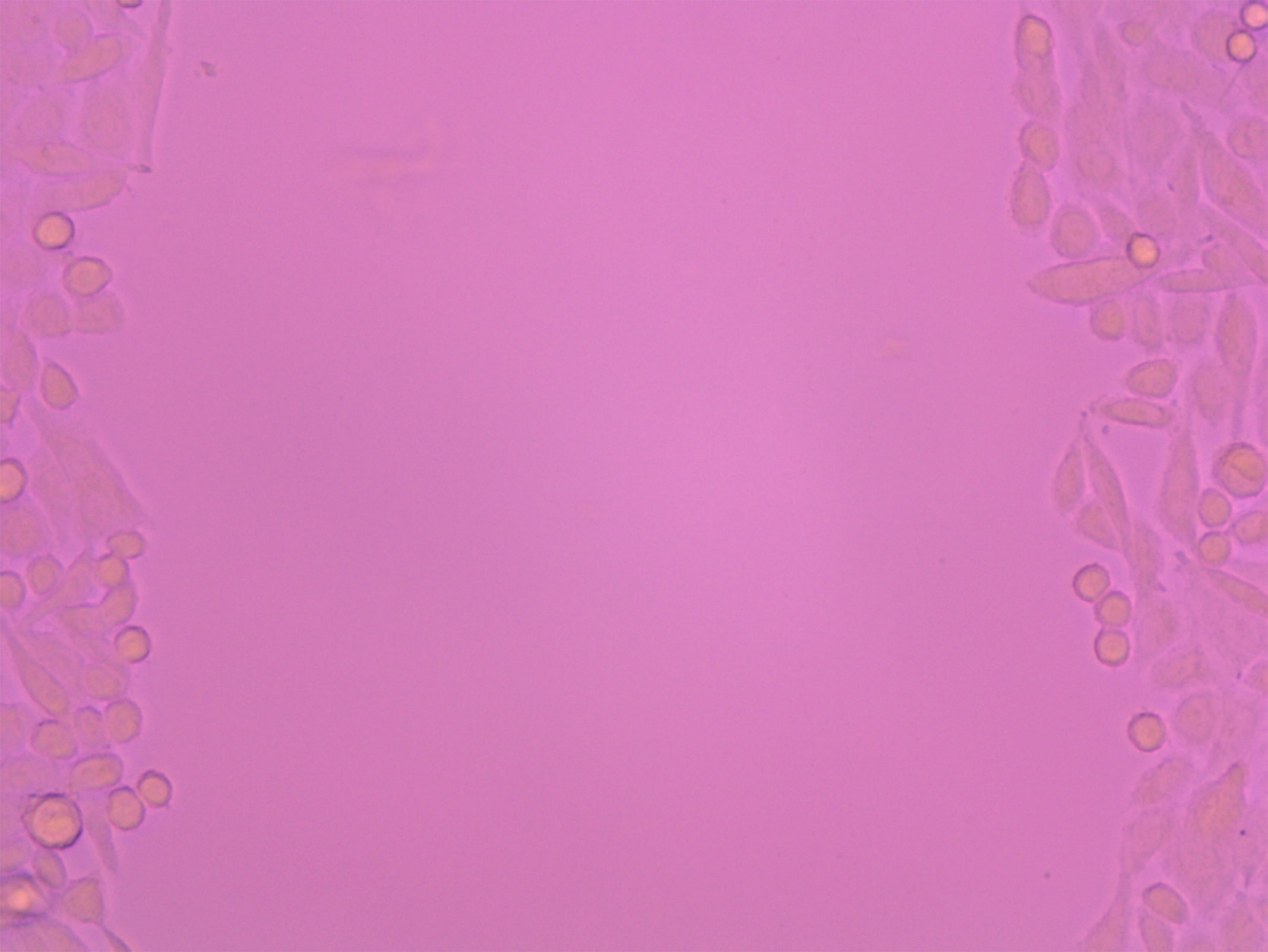

Supplement: S1 Data — (ZIP) [file pgen.1010366.s005.zip › 1D 5637 0h Vector.png]

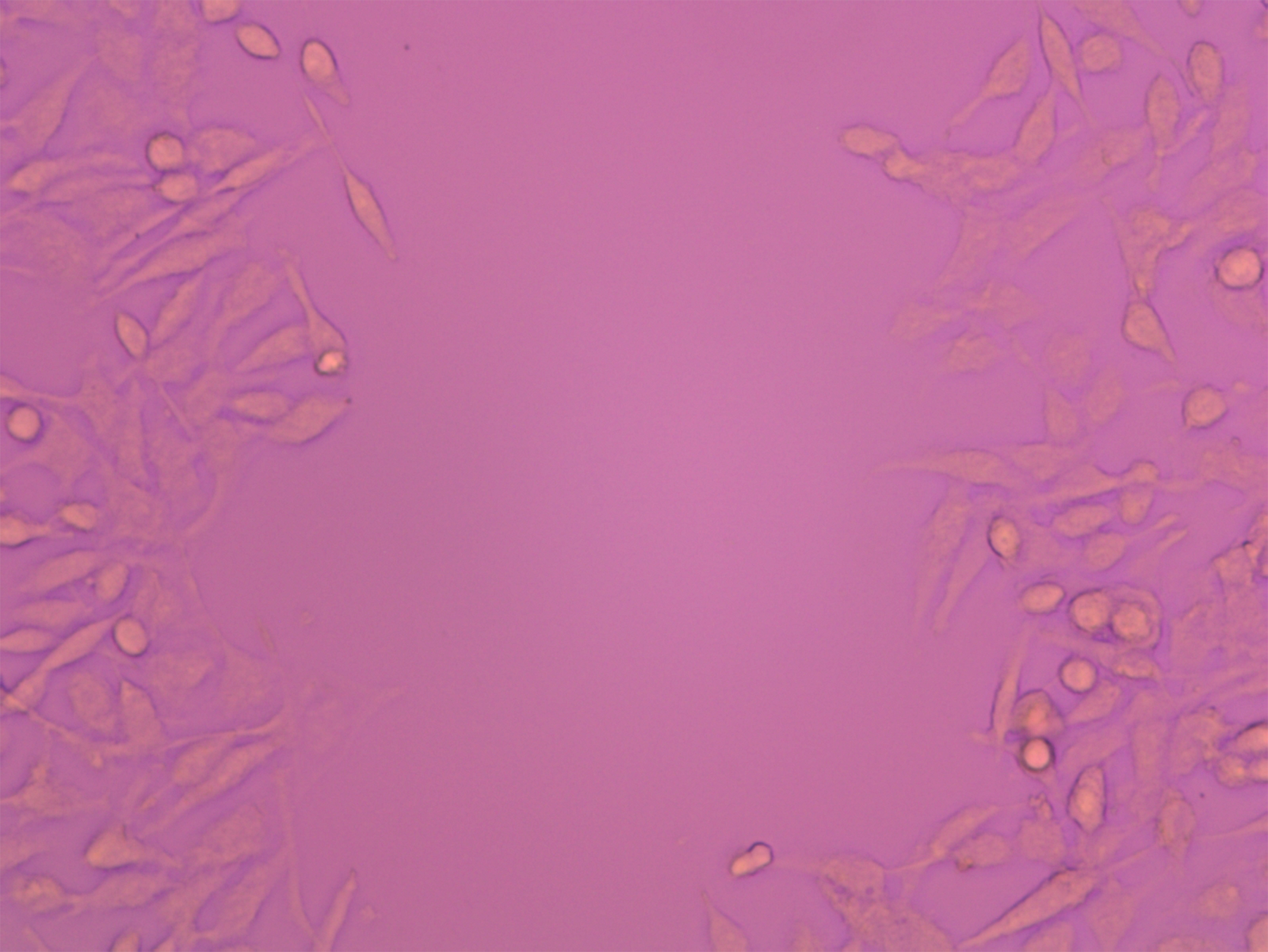

Supplement: S1 Data — (ZIP) [file pgen.1010366.s005.zip › 1D 5637 24h METTL14.png]

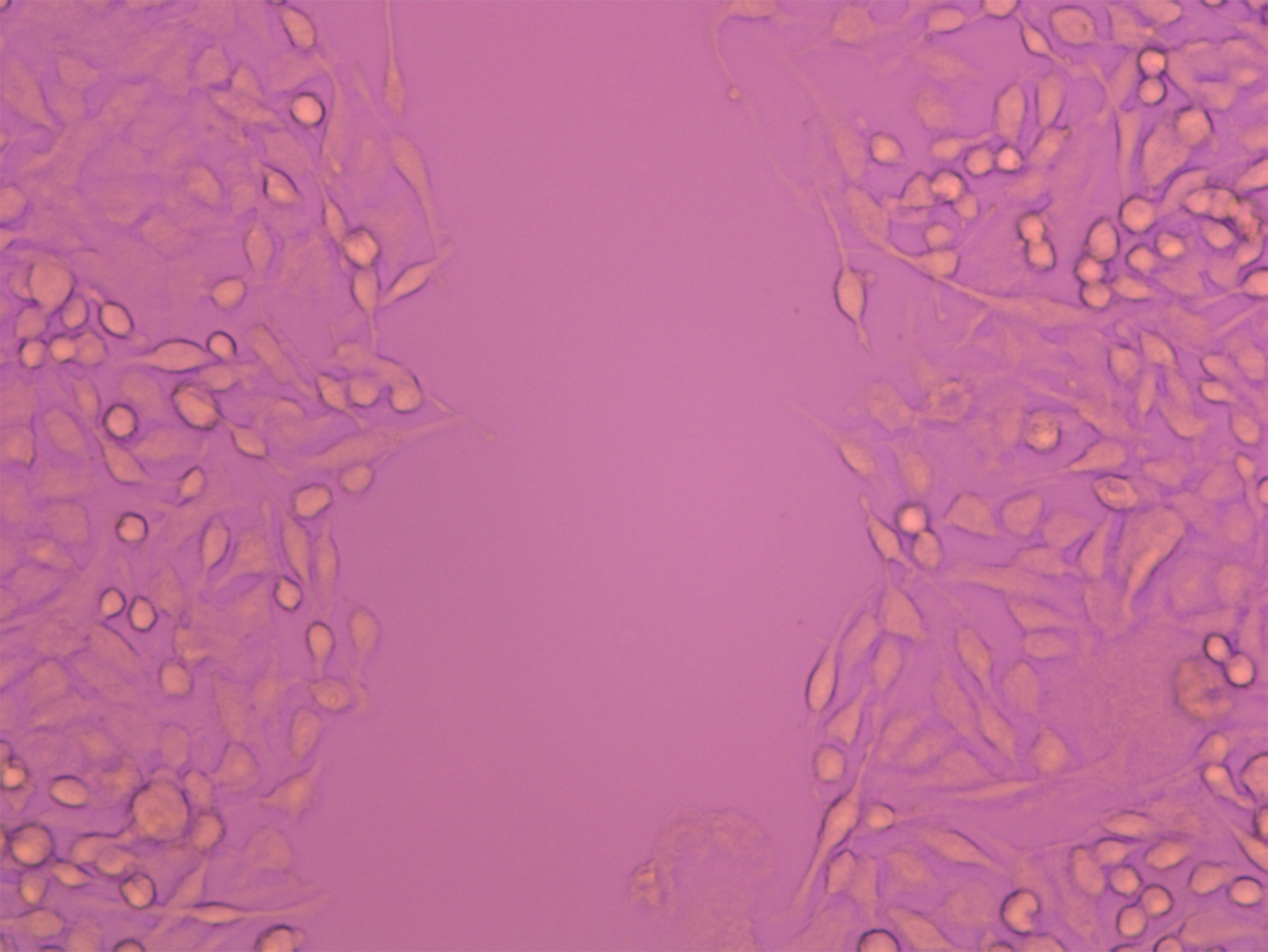

Supplement: S1 Data — (ZIP) [file pgen.1010366.s005.zip › 1D 5637 24h Vector.png]

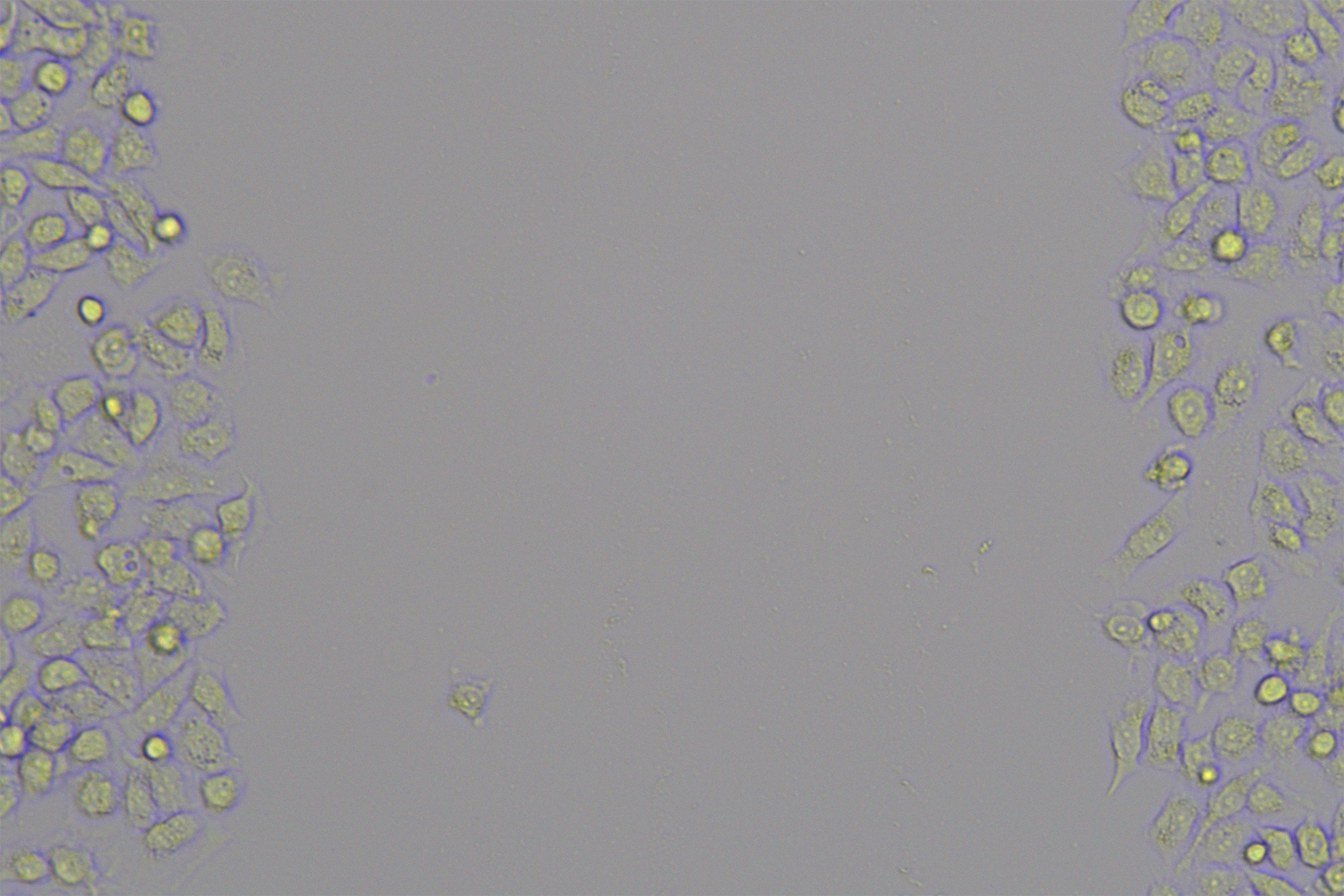

Supplement: S1 Data — (ZIP) [file pgen.1010366.s005.zip › 1D T24 0h METTL14.png]

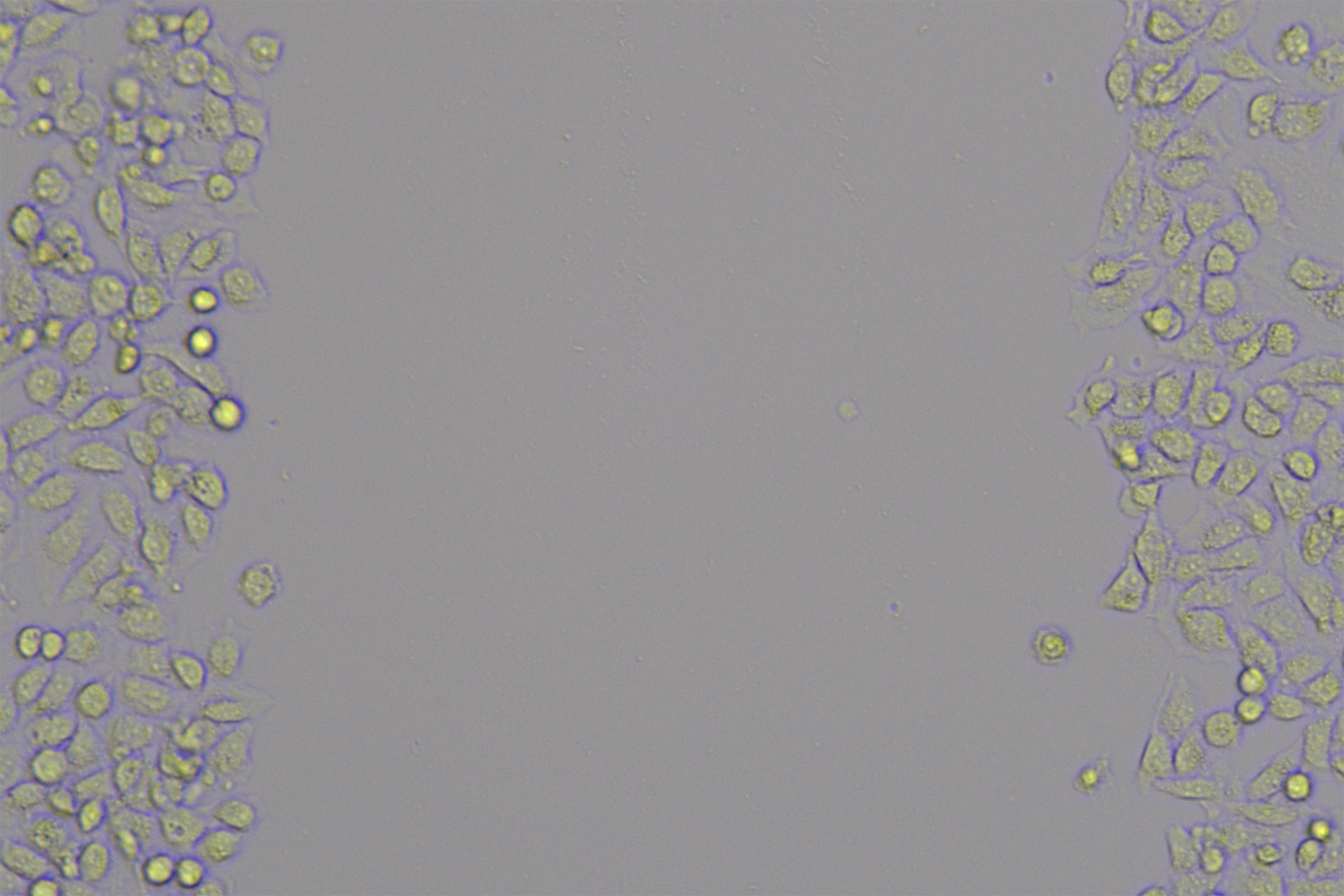

Supplement: S1 Data — (ZIP) [file pgen.1010366.s005.zip › 1D T24 0h Vector.png]

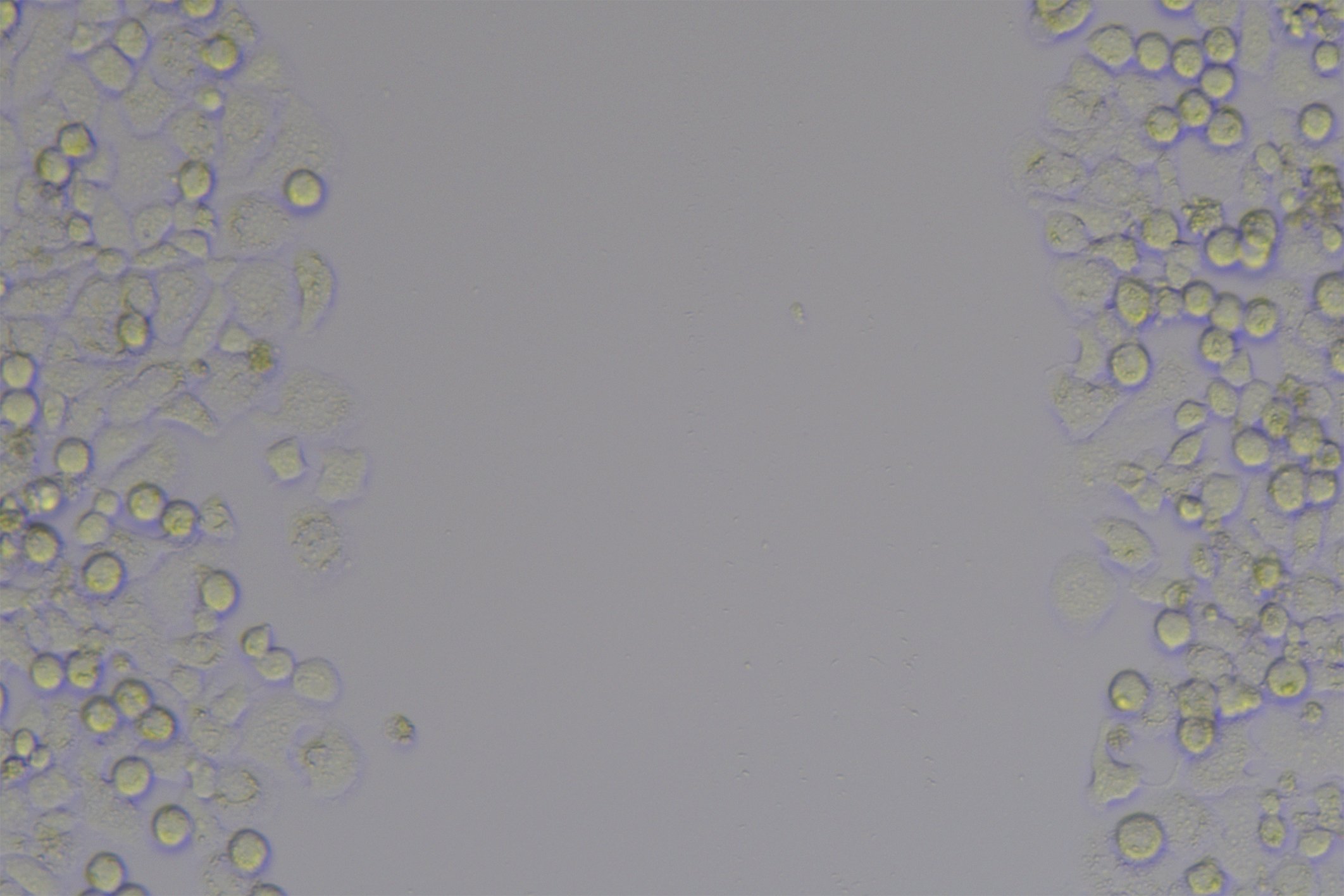

Supplement: S1 Data — (ZIP) [file pgen.1010366.s005.zip › 1D T24 24h METTL14.png]

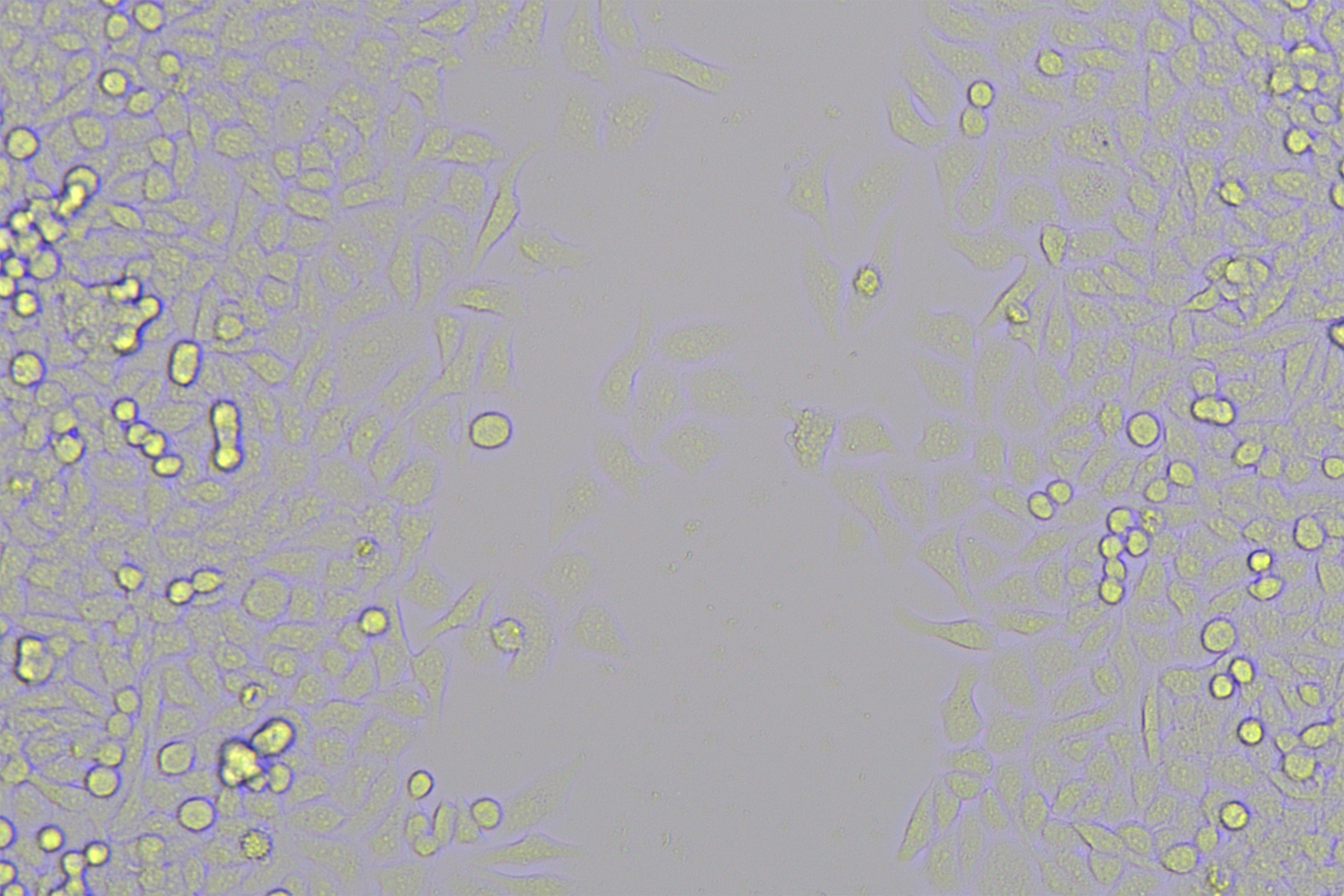

Supplement: S1 Data — (ZIP) [file pgen.1010366.s005.zip › 1D T24 24h Vector.png]

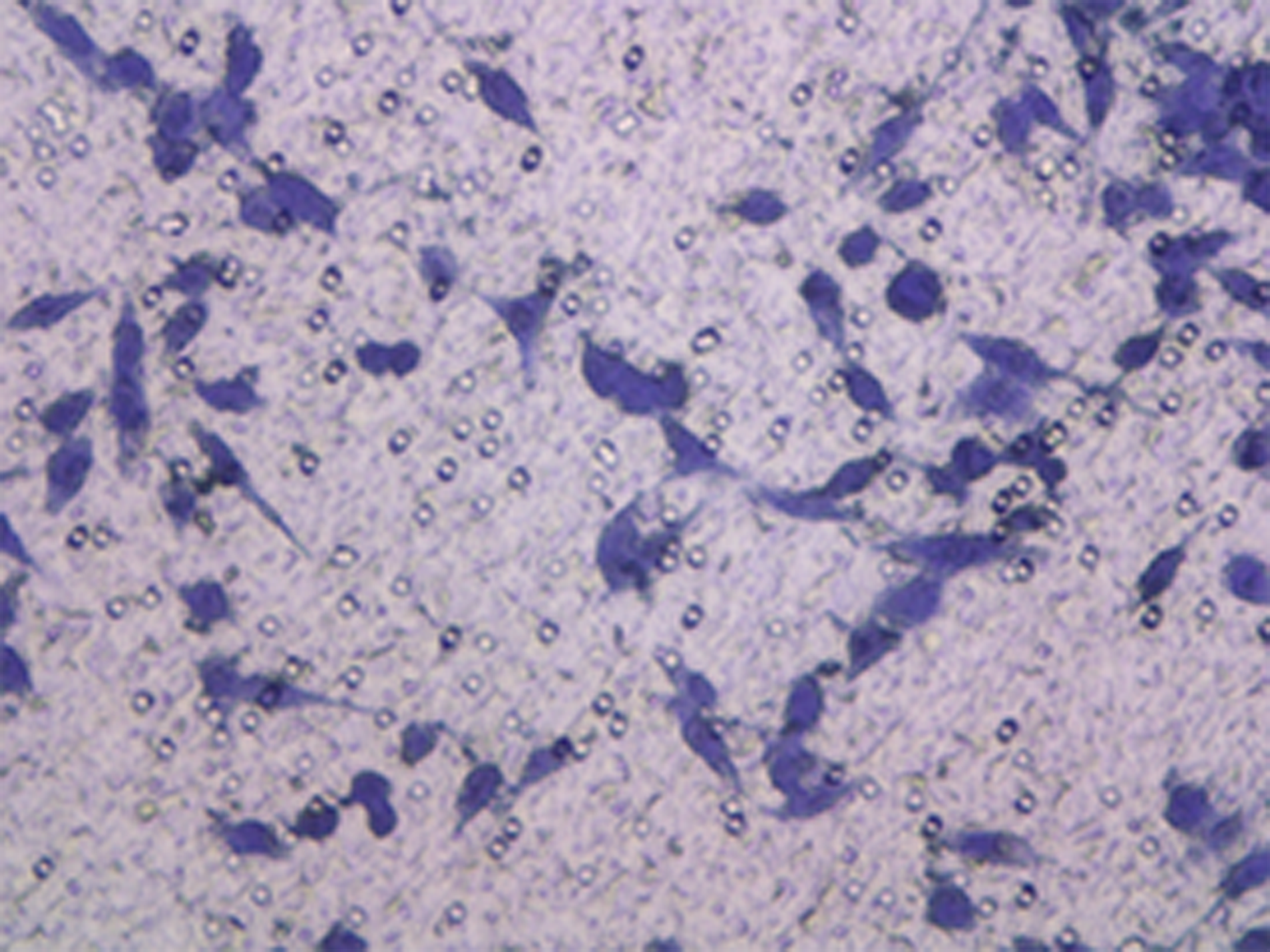

Supplement: S1 Data — (ZIP) [file pgen.1010366.s005.zip › 1F Invasion 5637 METTL14.png]

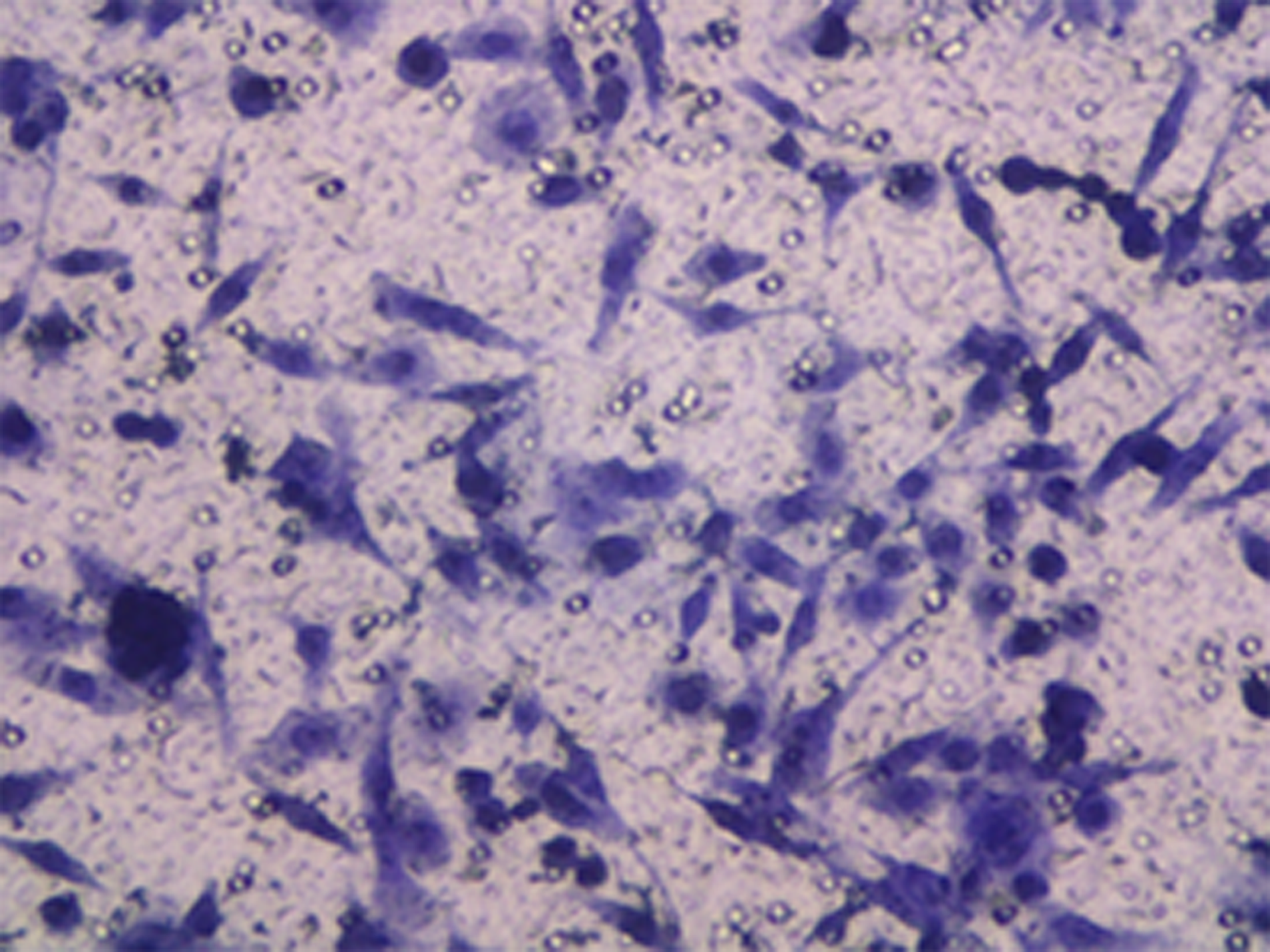

Supplement: S1 Data — (ZIP) [file pgen.1010366.s005.zip › 1F Invasion 5637 Vector.png]

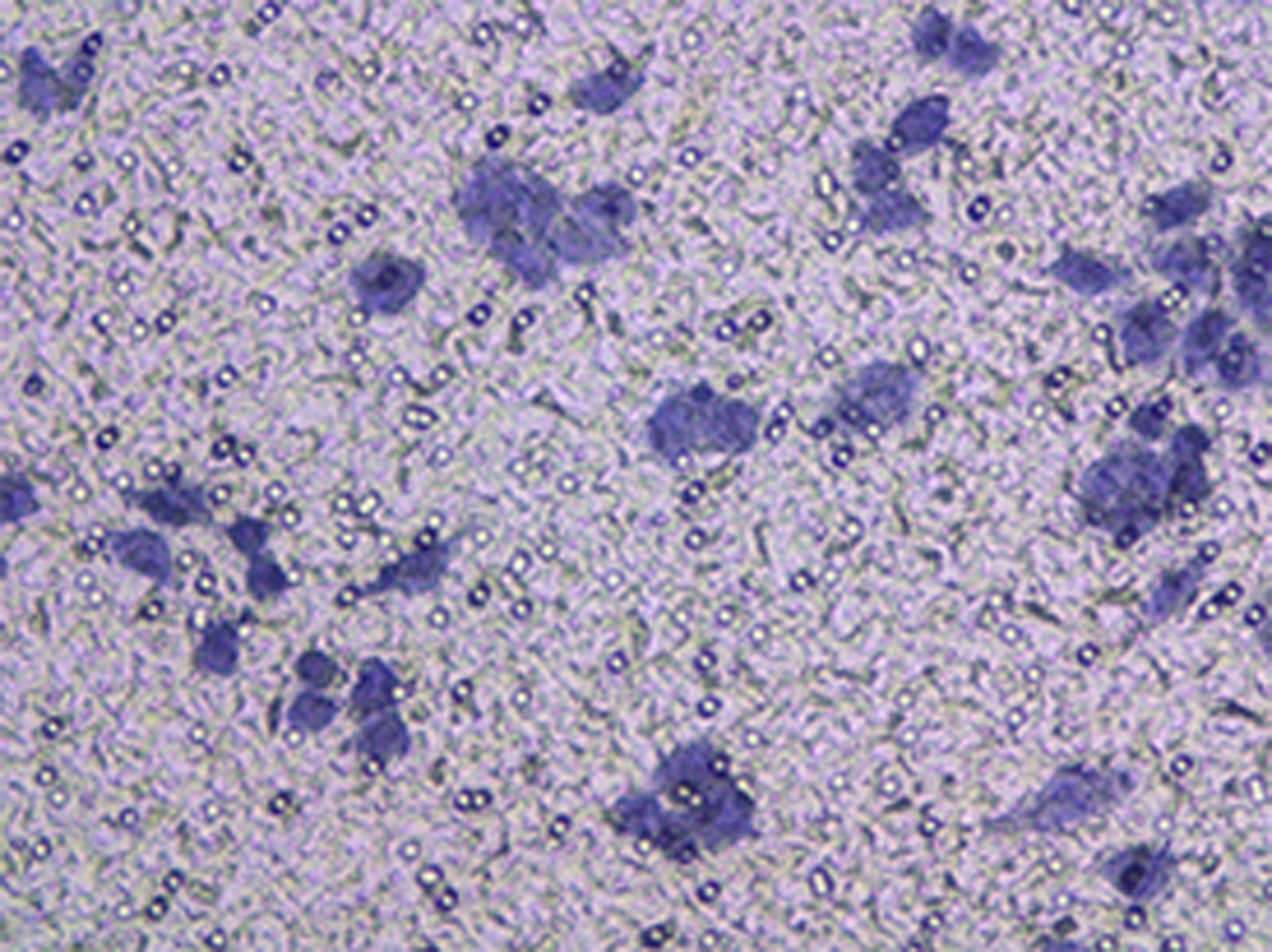

Supplement: S1 Data — (ZIP) [file pgen.1010366.s005.zip › 1F Invasion T24 METTL14.png]

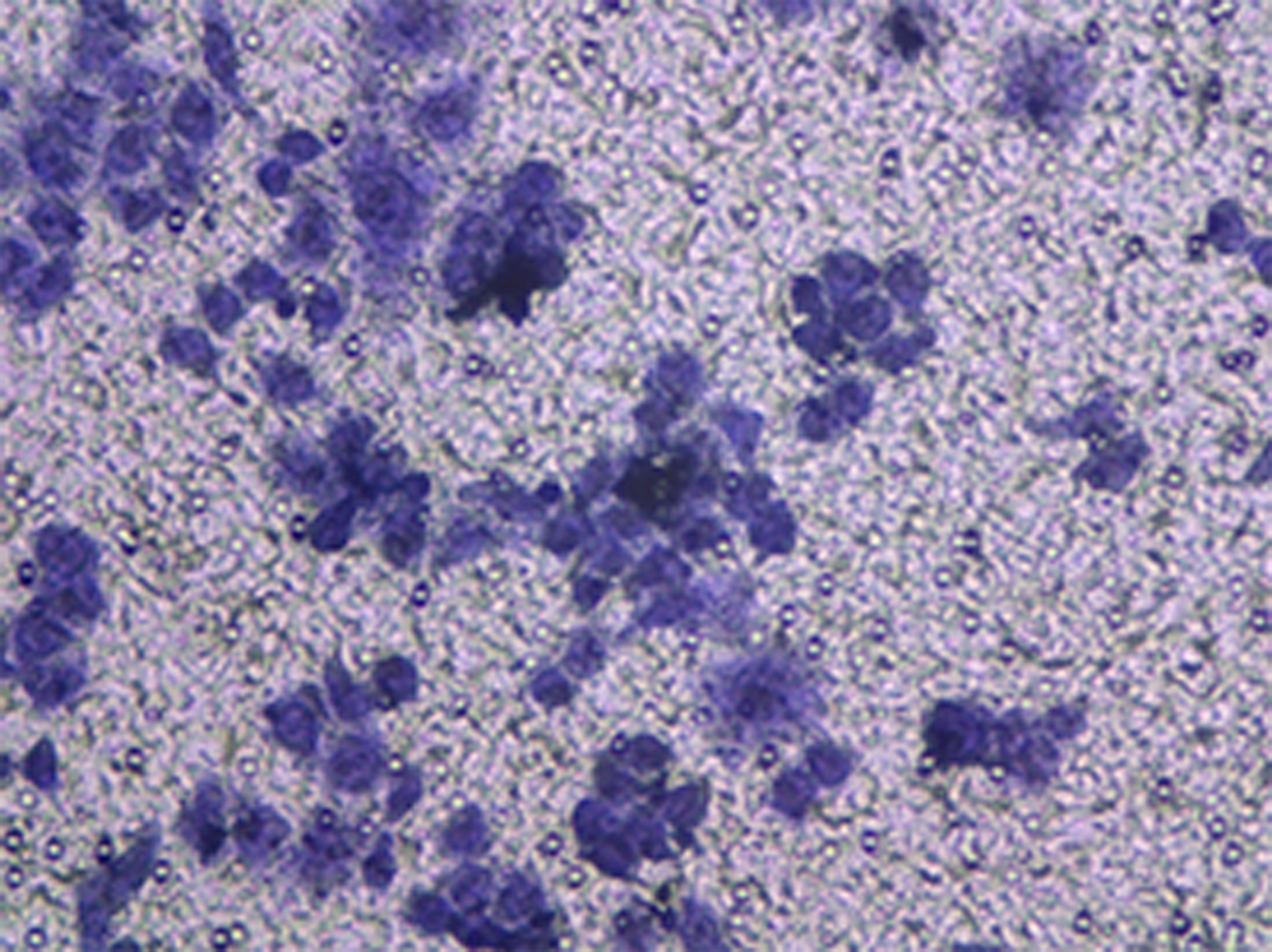

Supplement: S1 Data — (ZIP) [file pgen.1010366.s005.zip › 1F Invasion T24 Vector.png]

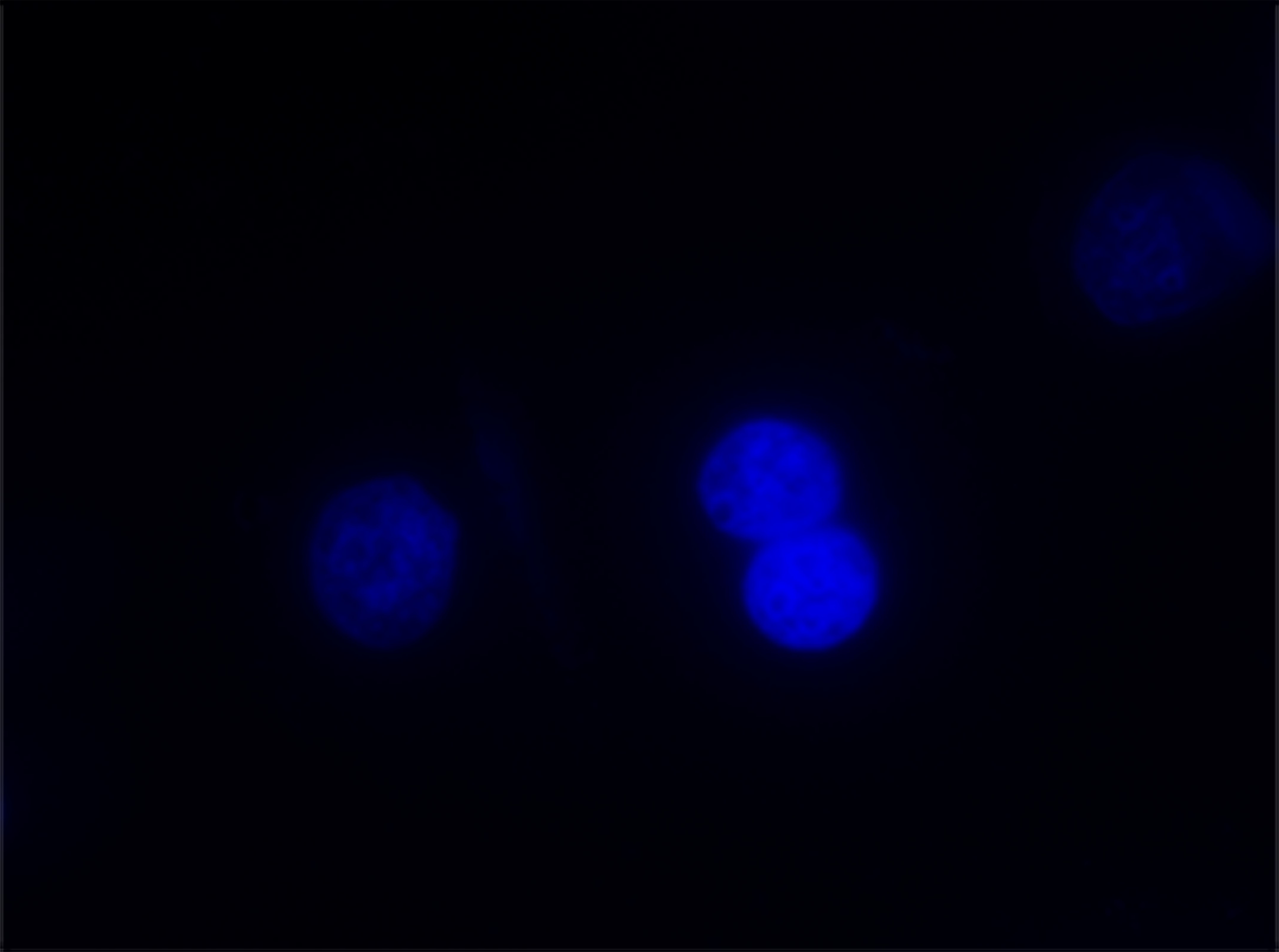

Supplement: S1 Data — (ZIP) [file pgen.1010366.s005.zip › 1G 5637 METTL14 DAPI.png]

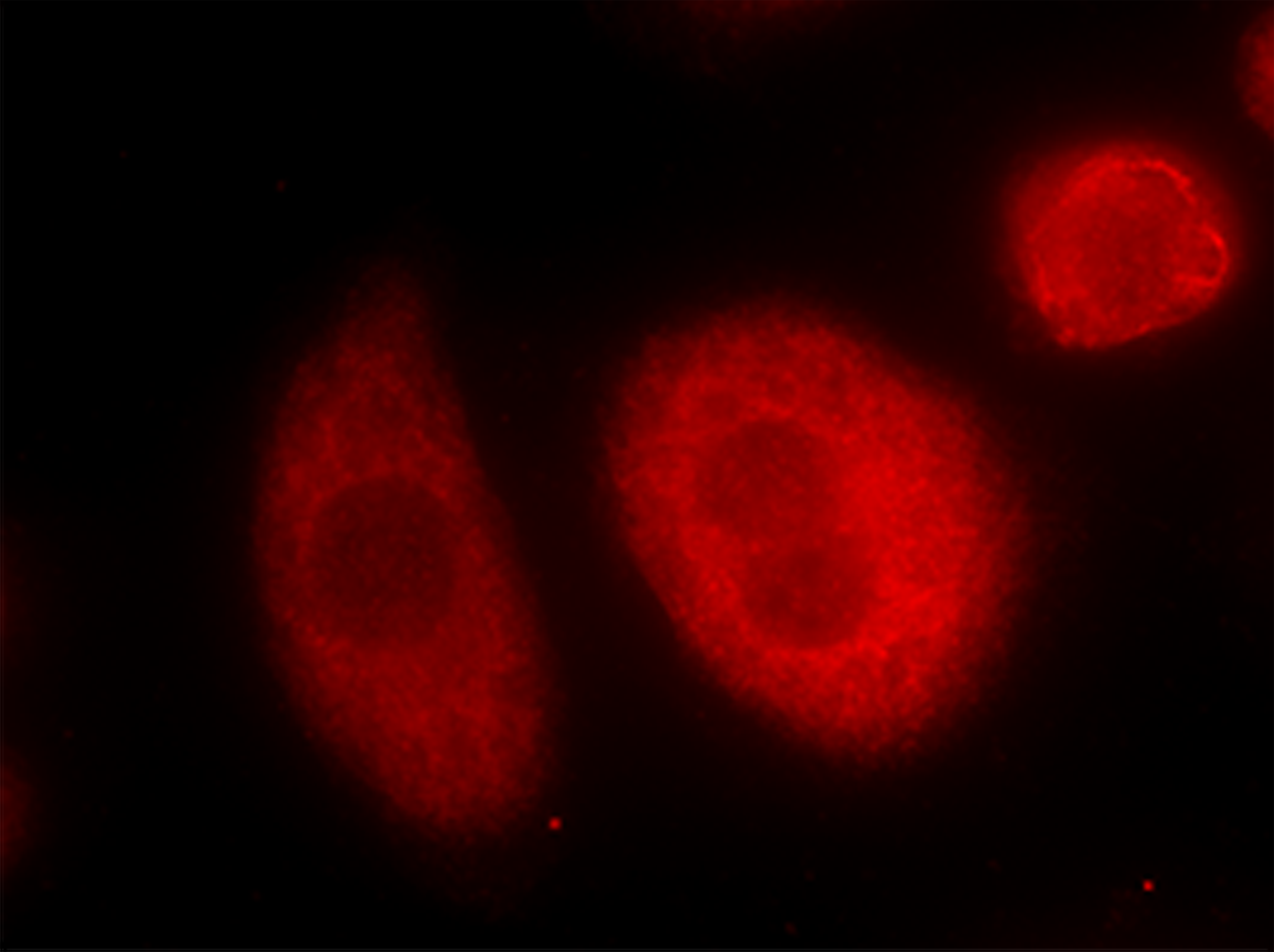

Supplement: S1 Data — (ZIP) [file pgen.1010366.s005.zip › 1G 5637 METTL14 E-cadherin.png]

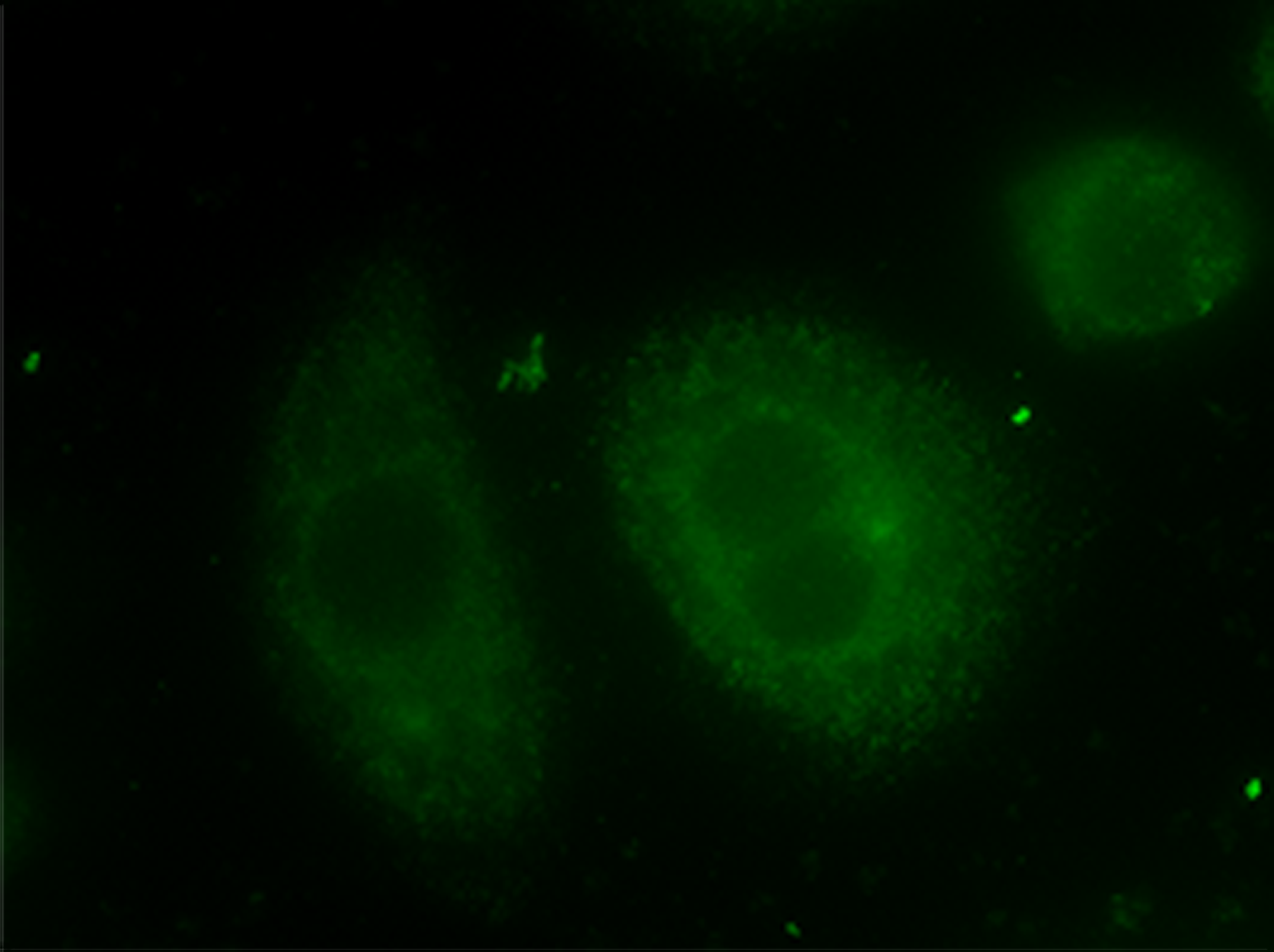

Supplement: S1 Data — (ZIP) [file pgen.1010366.s005.zip › 1G 5637 METTL14 N-cadherin.png]

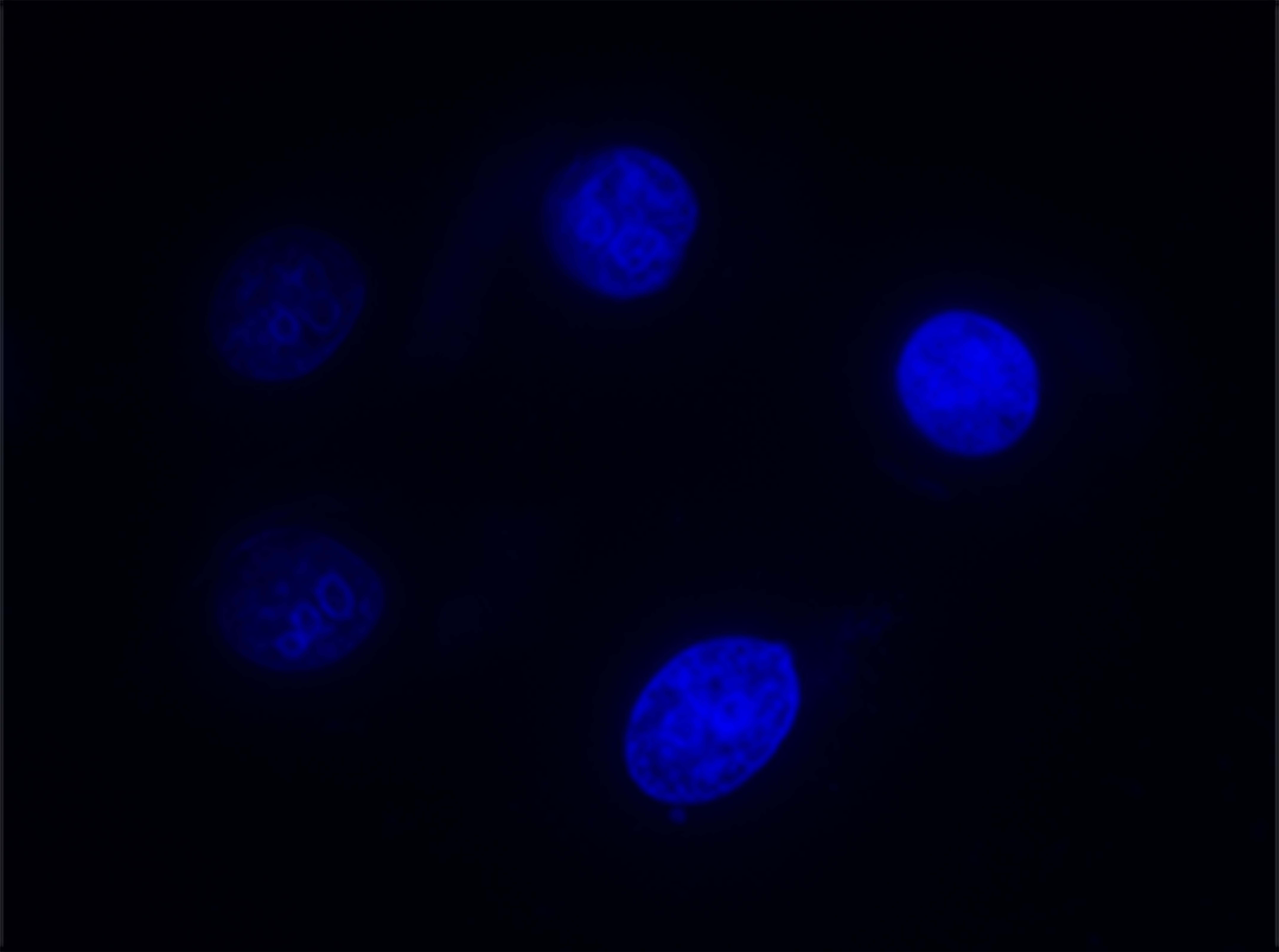

Supplement: S1 Data — (ZIP) [file pgen.1010366.s005.zip › 1G 5637 Vector DAPI.png]

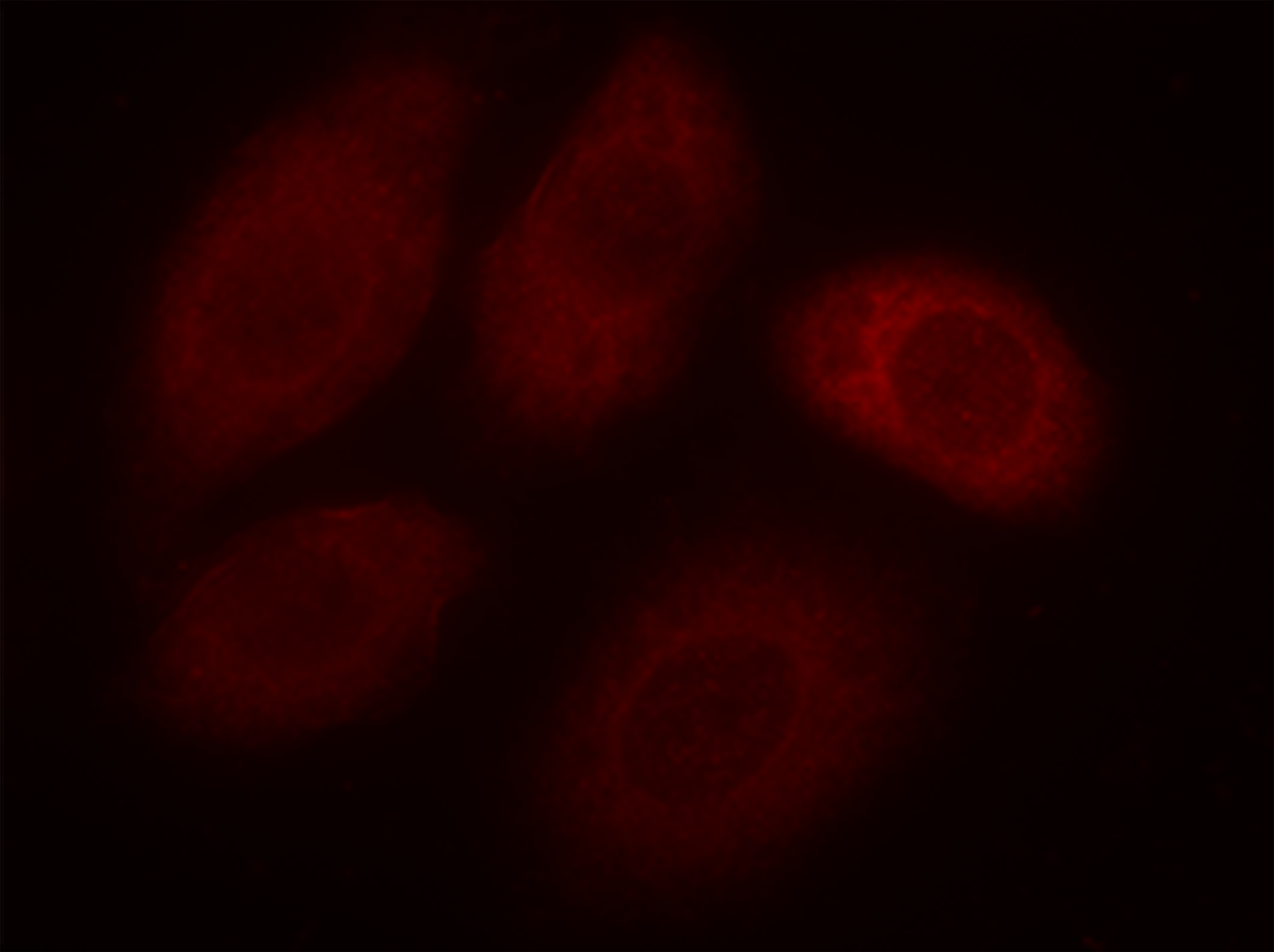

Supplement: S1 Data — (ZIP) [file pgen.1010366.s005.zip › 1G 5637 Vector E-cadherin.png]

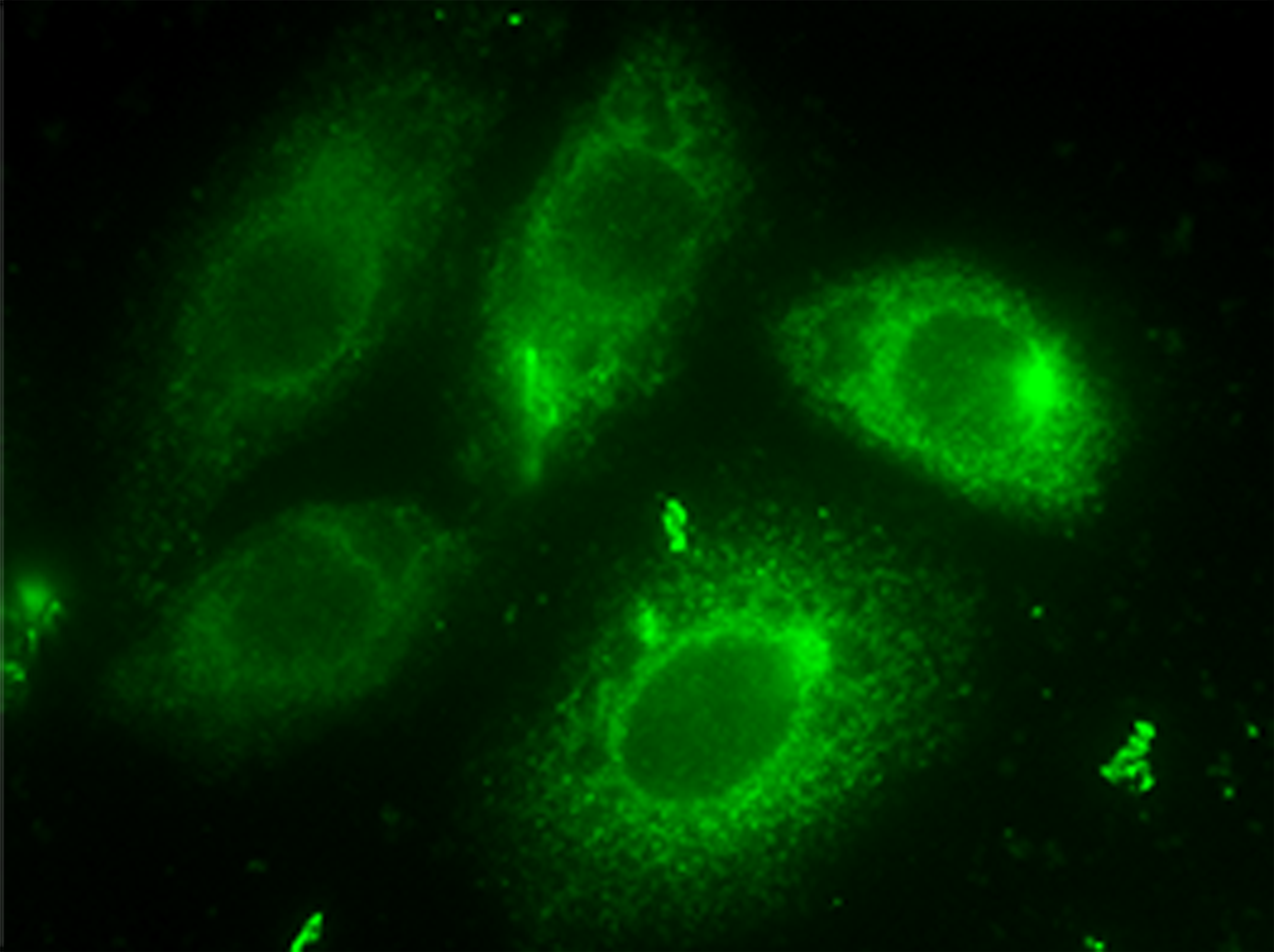

Supplement: S1 Data — (ZIP) [file pgen.1010366.s005.zip › 1G 5637 Vector N-cadherin.png]

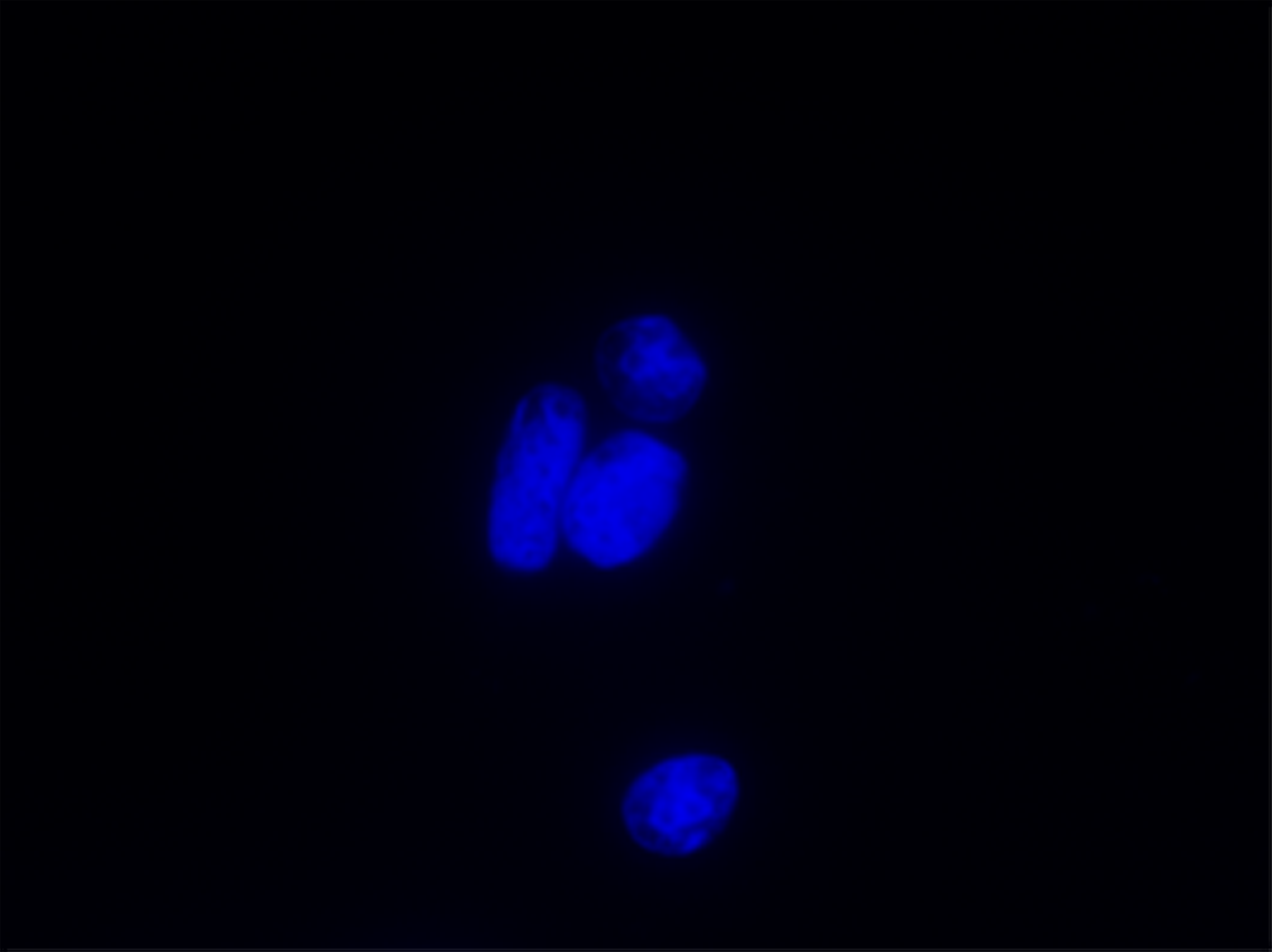

Supplement: S1 Data — (ZIP) [file pgen.1010366.s005.zip › 1G T24 METTL14 DAPI.png]

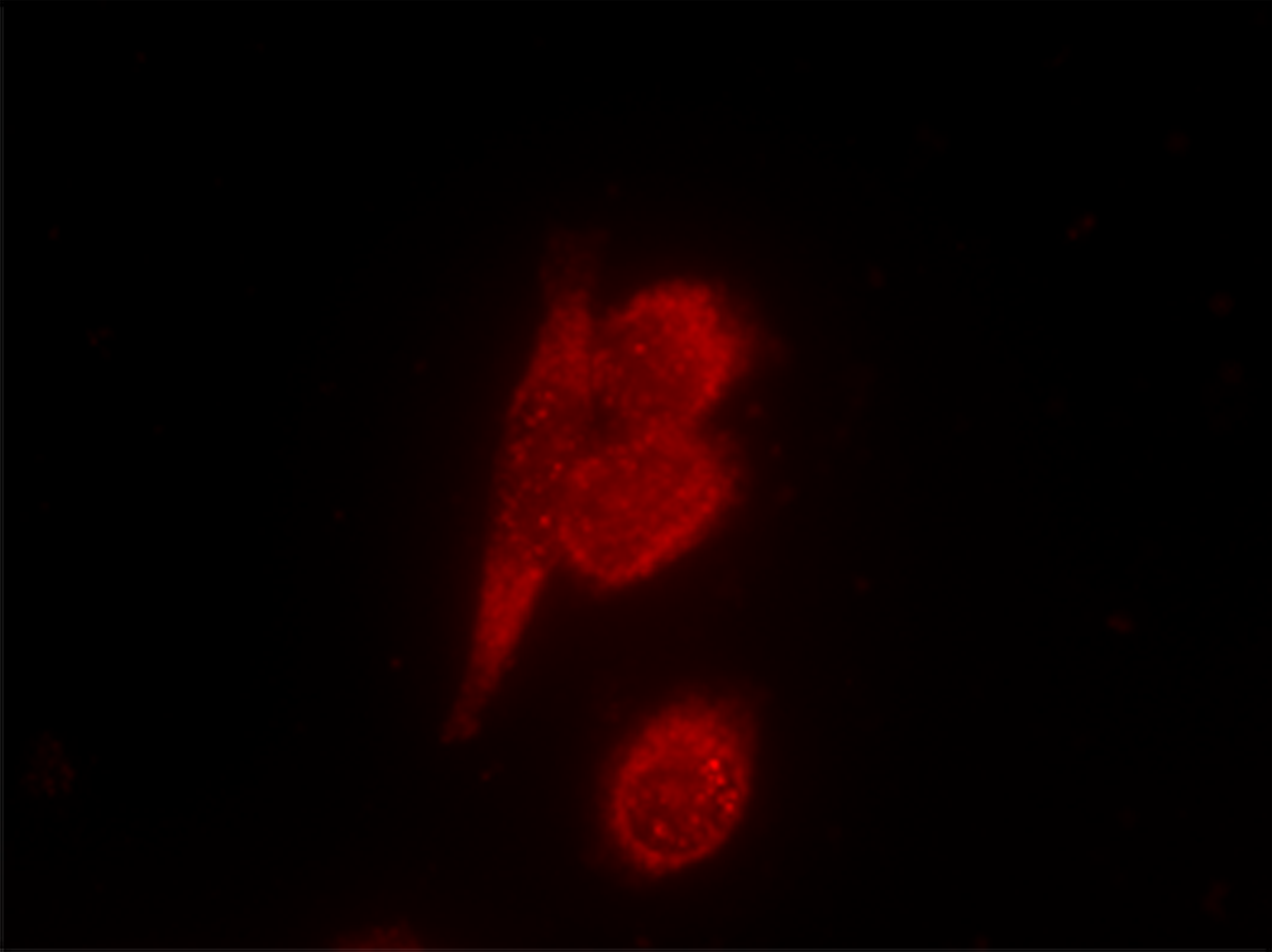

Supplement: S1 Data — (ZIP) [file pgen.1010366.s005.zip › 1G T24 METTL14 E-cadherin.png]

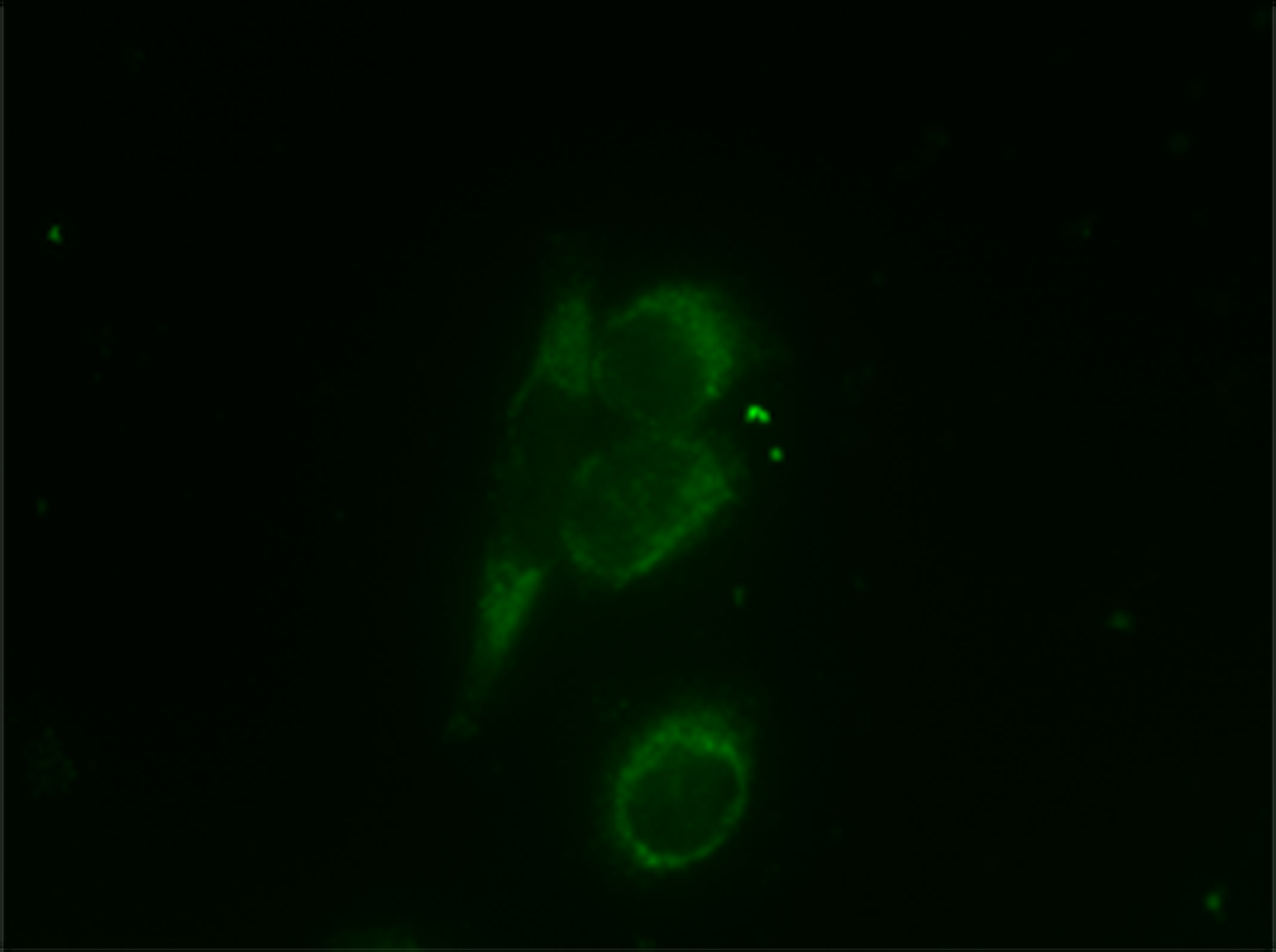

Supplement: S1 Data — (ZIP) [file pgen.1010366.s005.zip › 1G T24 METTL14 N-cadherin.png]

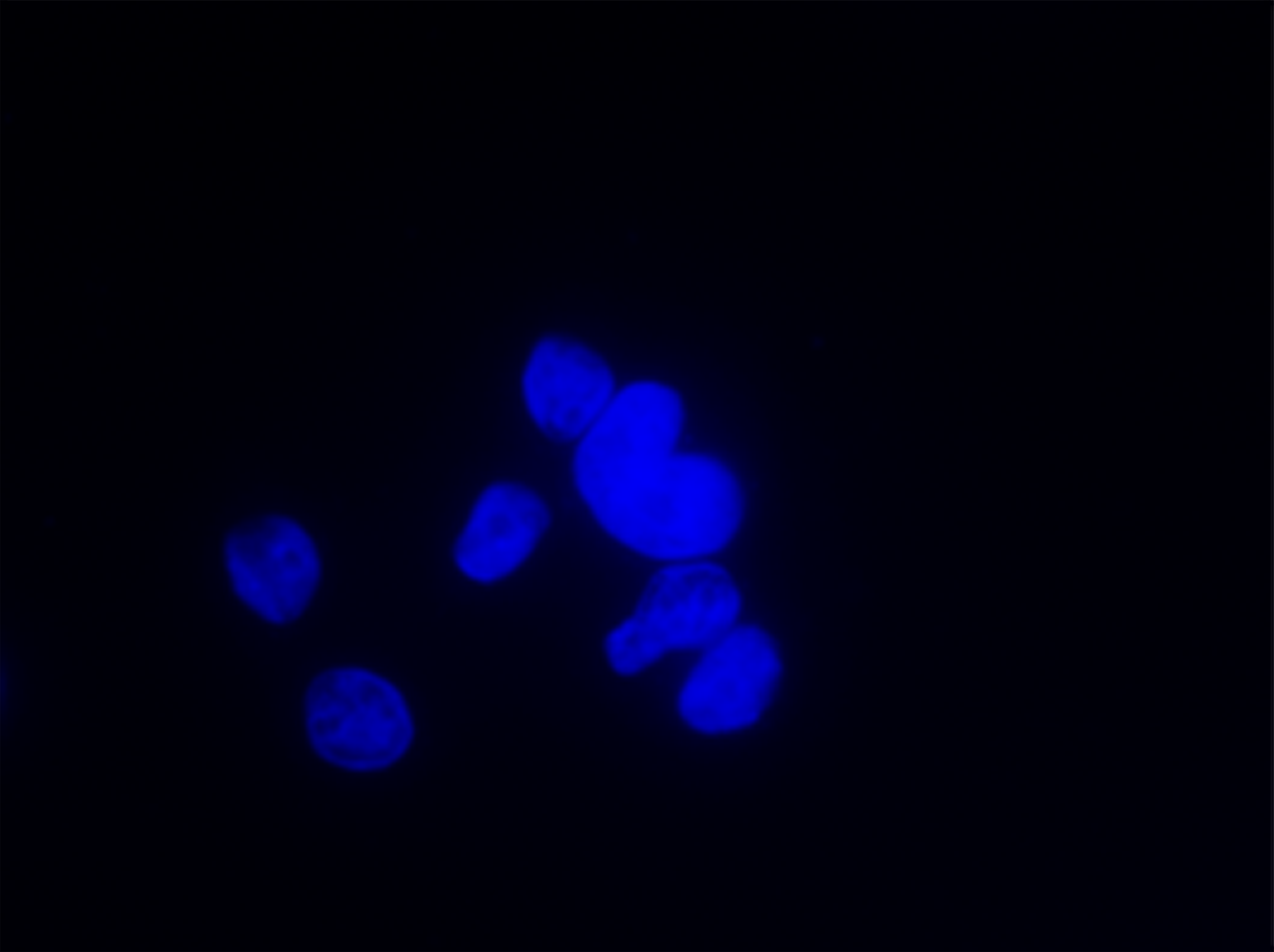

Supplement: S1 Data — (ZIP) [file pgen.1010366.s005.zip › 1G T24 Vector DAPI.png]

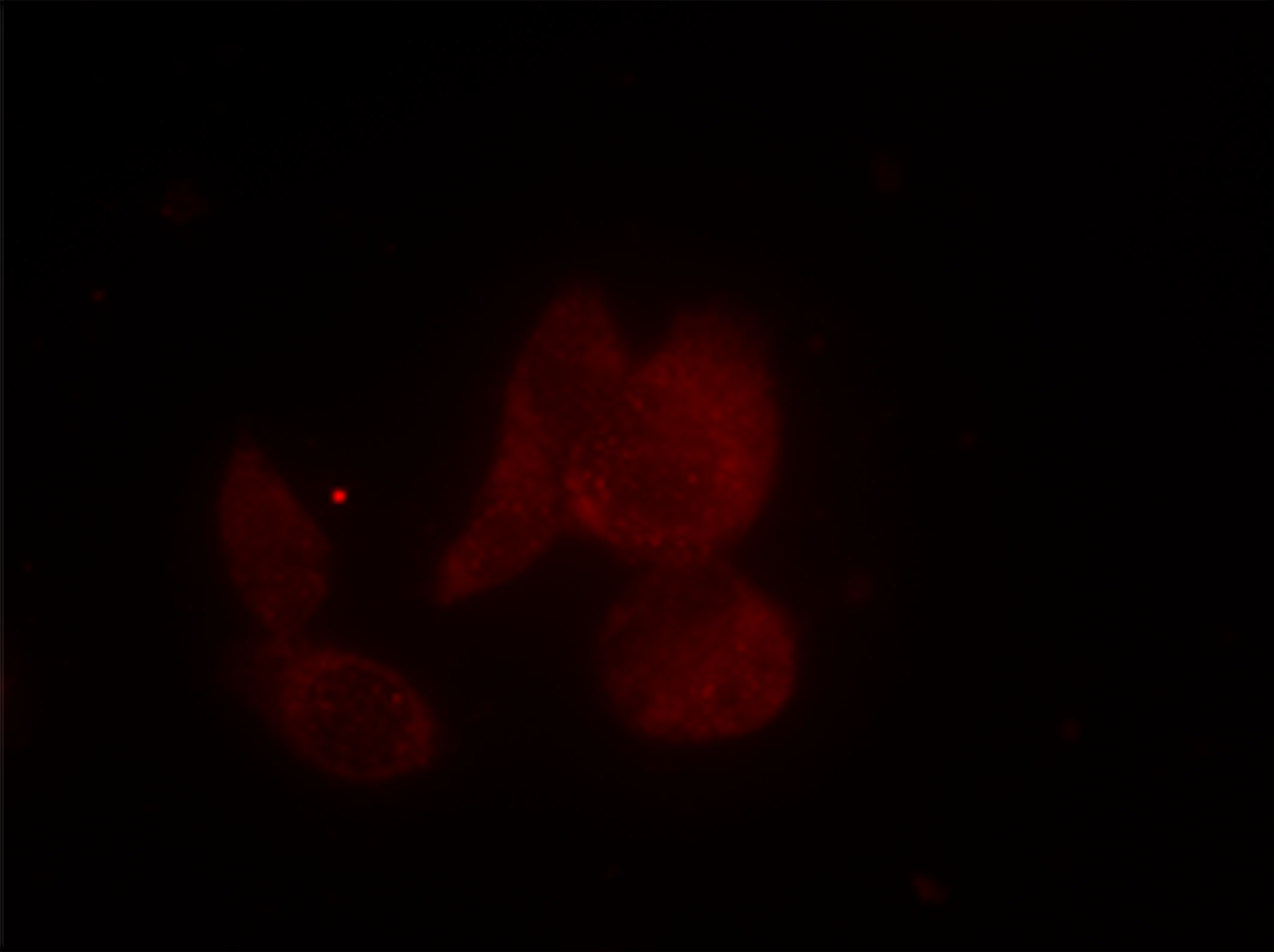

Supplement: S1 Data — (ZIP) [file pgen.1010366.s005.zip › 1G T24 Vector E-cadherin.png]

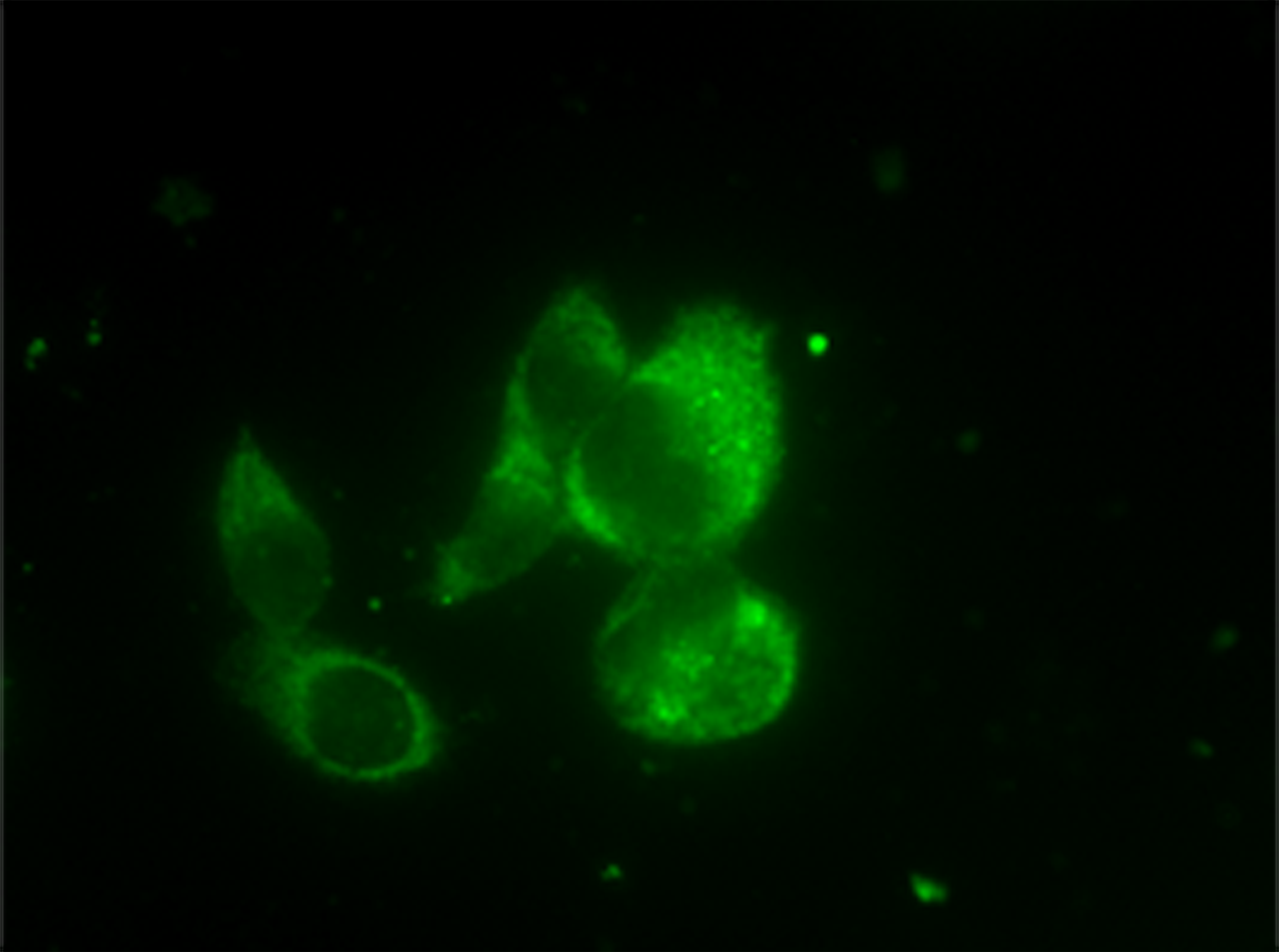

Supplement: S1 Data — (ZIP) [file pgen.1010366.s005.zip › 1G T24 Vector N-cadherin.png]

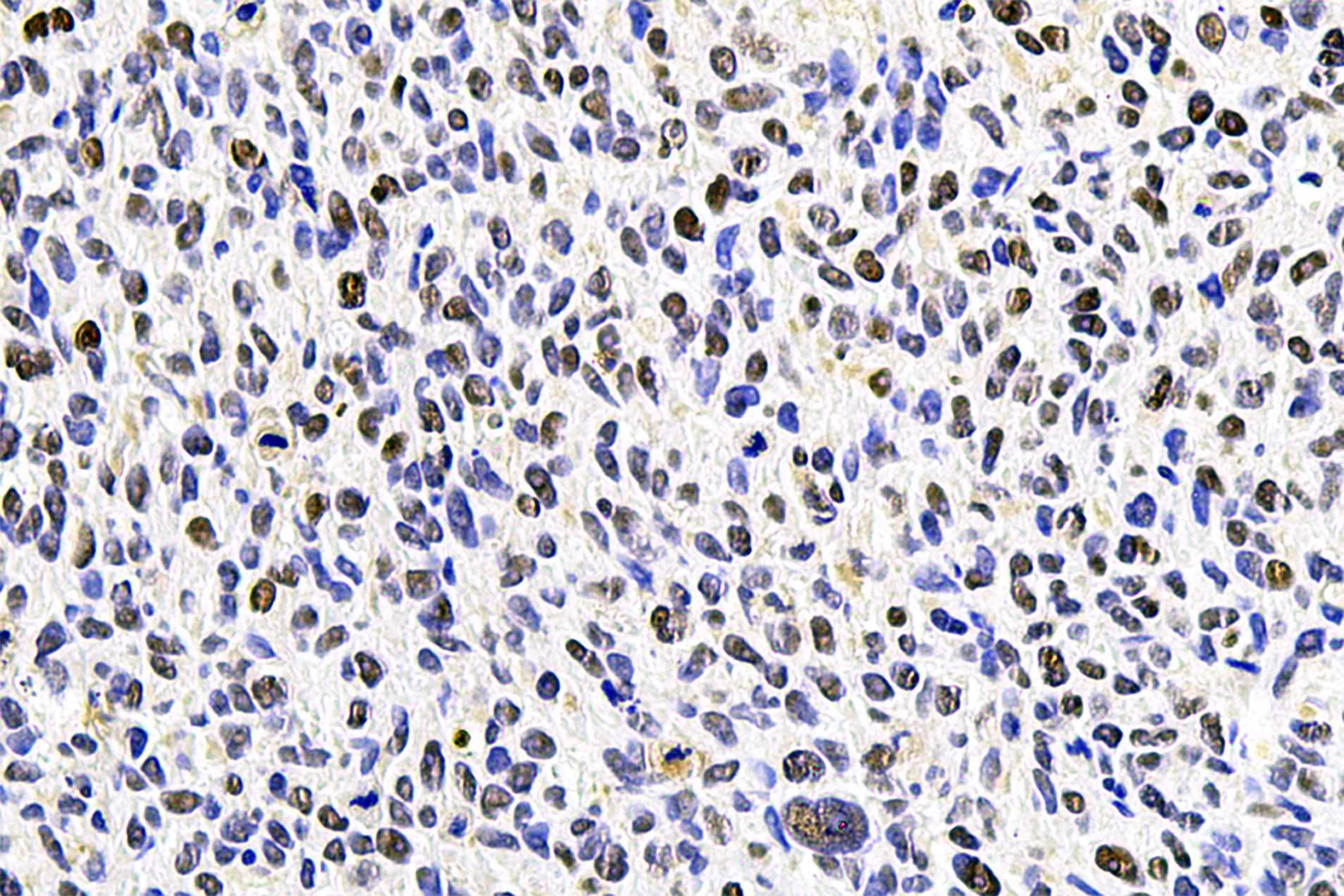

Supplement: S1 Data — (ZIP) [file pgen.1010366.s005.zip › 1J METTL14 METTL14.png]

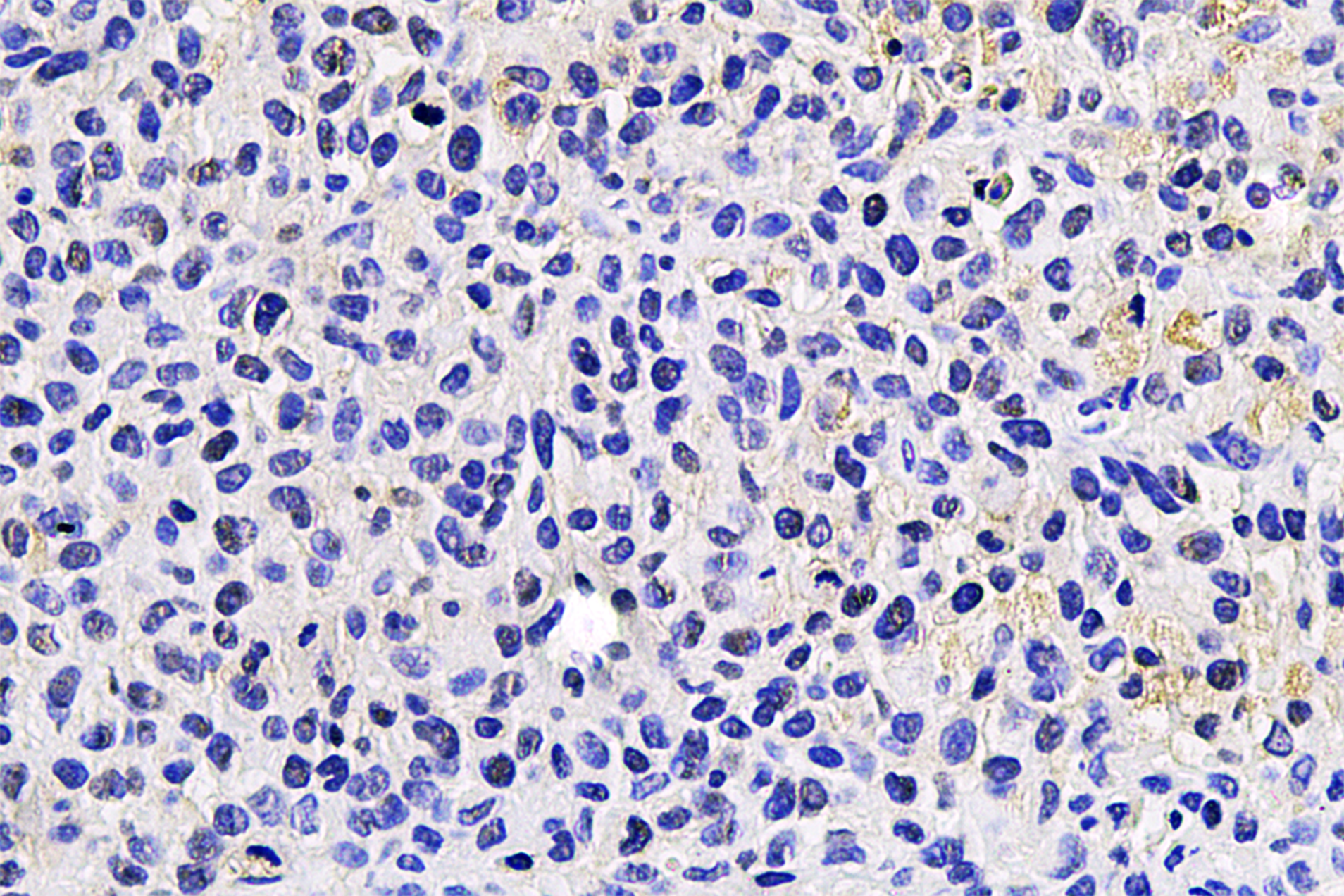

Supplement: S1 Data — (ZIP) [file pgen.1010366.s005.zip › 1J METTL14 Vector.png]

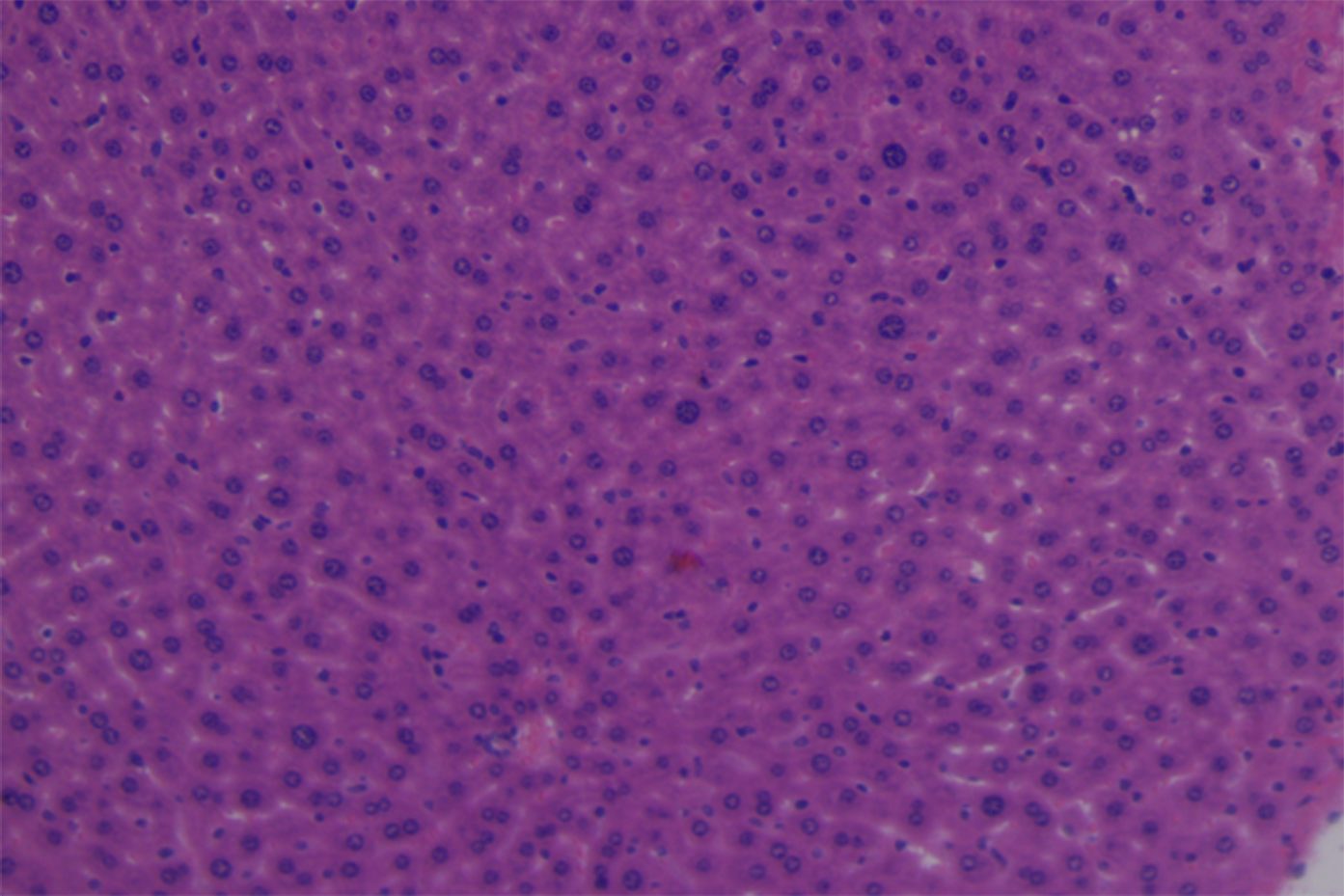

Supplement: S1 Data — (ZIP) [file pgen.1010366.s005.zip › 1K METTL14 Liver.png]

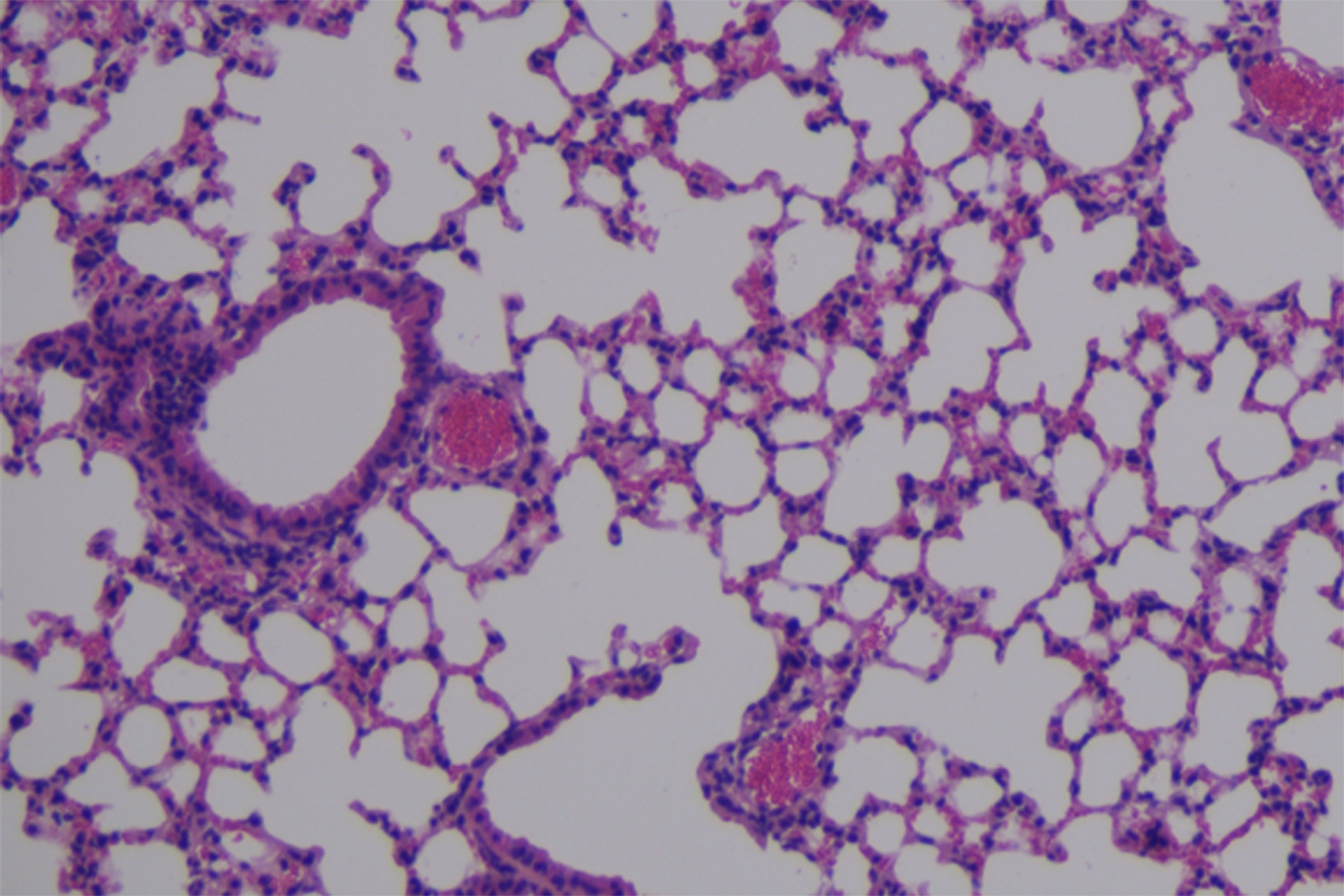

Supplement: S1 Data — (ZIP) [file pgen.1010366.s005.zip › 1K METTL14 Lung.png]

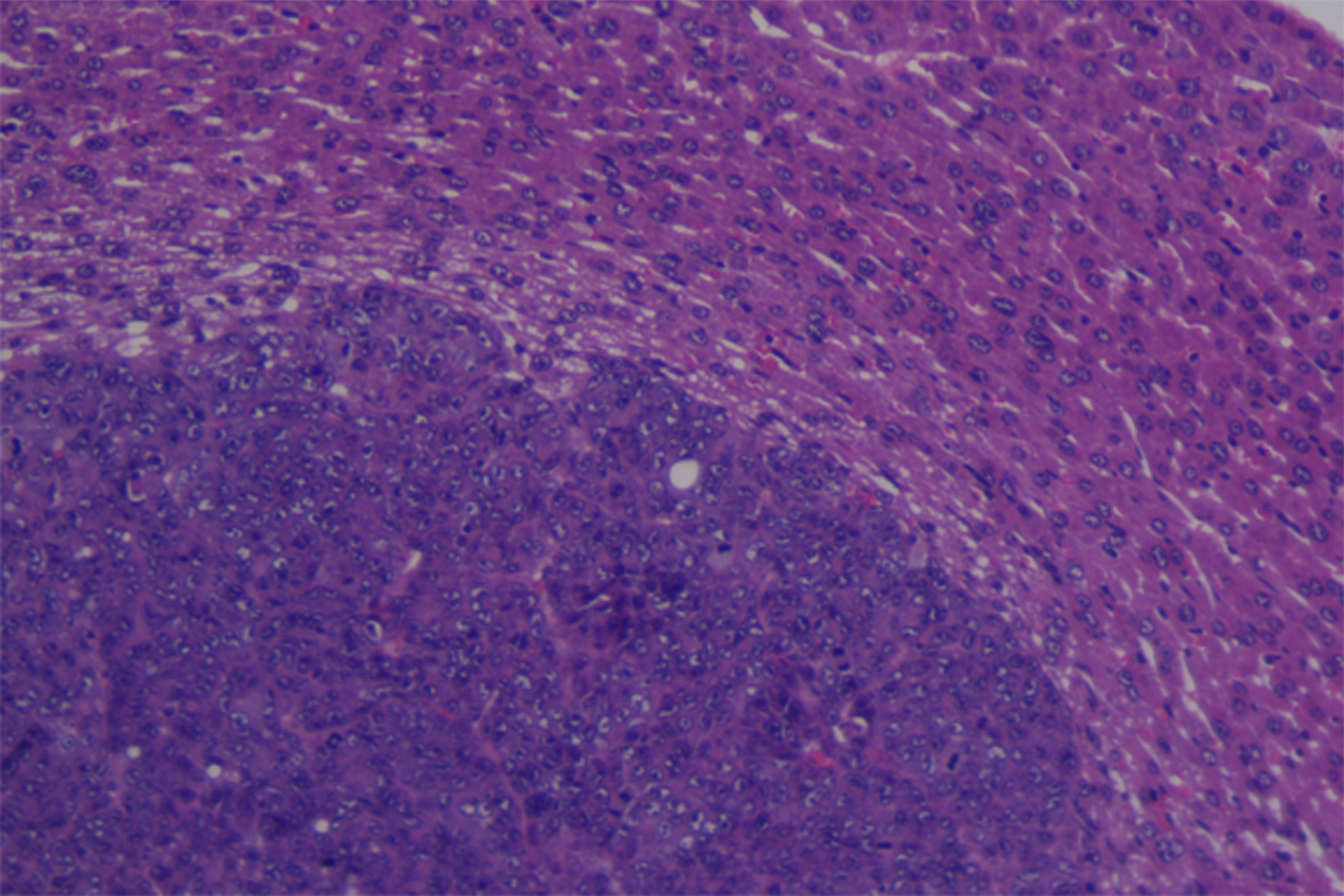

Supplement: S1 Data — (ZIP) [file pgen.1010366.s005.zip › 1K Vector Liver.png]

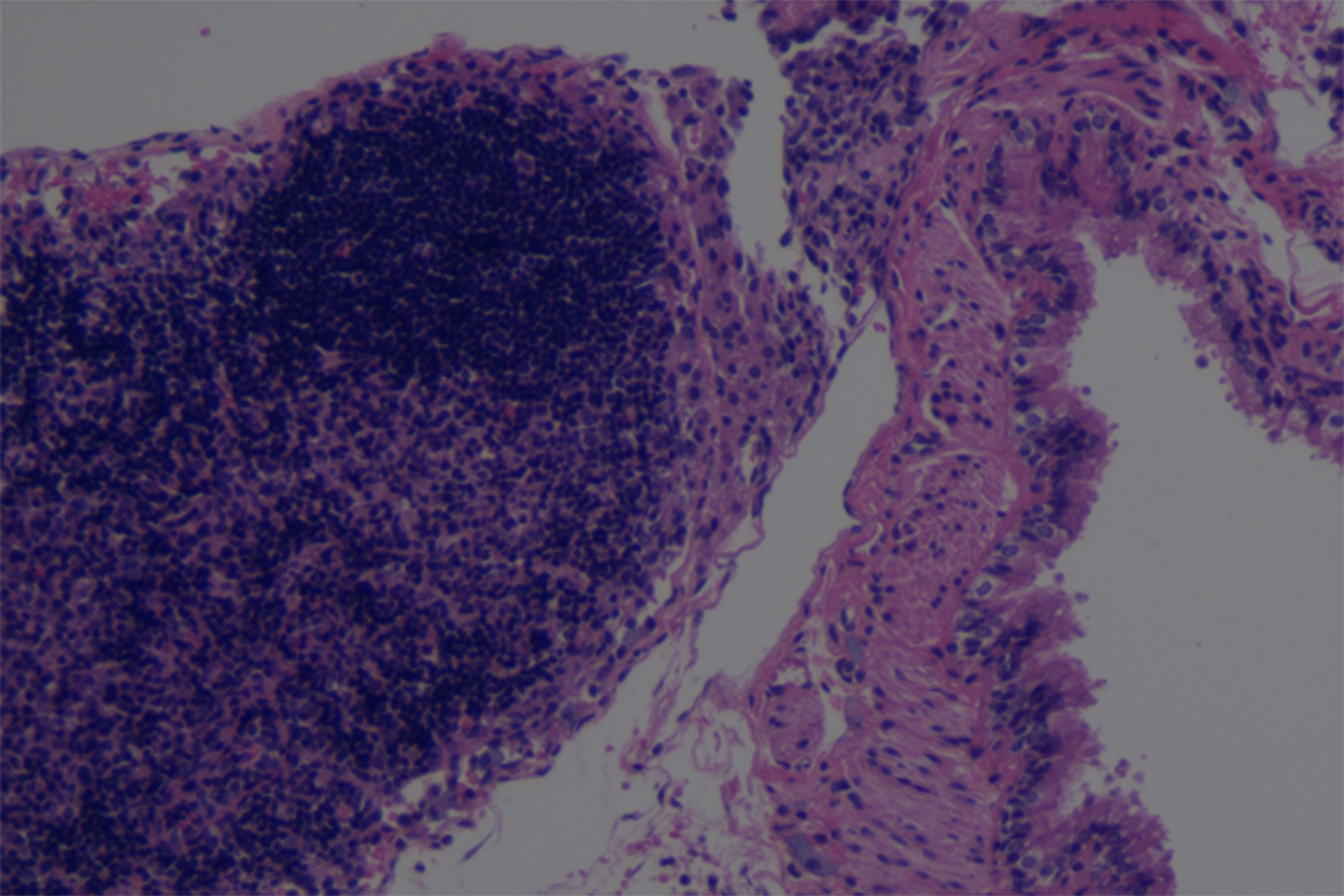

Supplement: S1 Data — (ZIP) [file pgen.1010366.s005.zip › 1K Vector Lung.png]

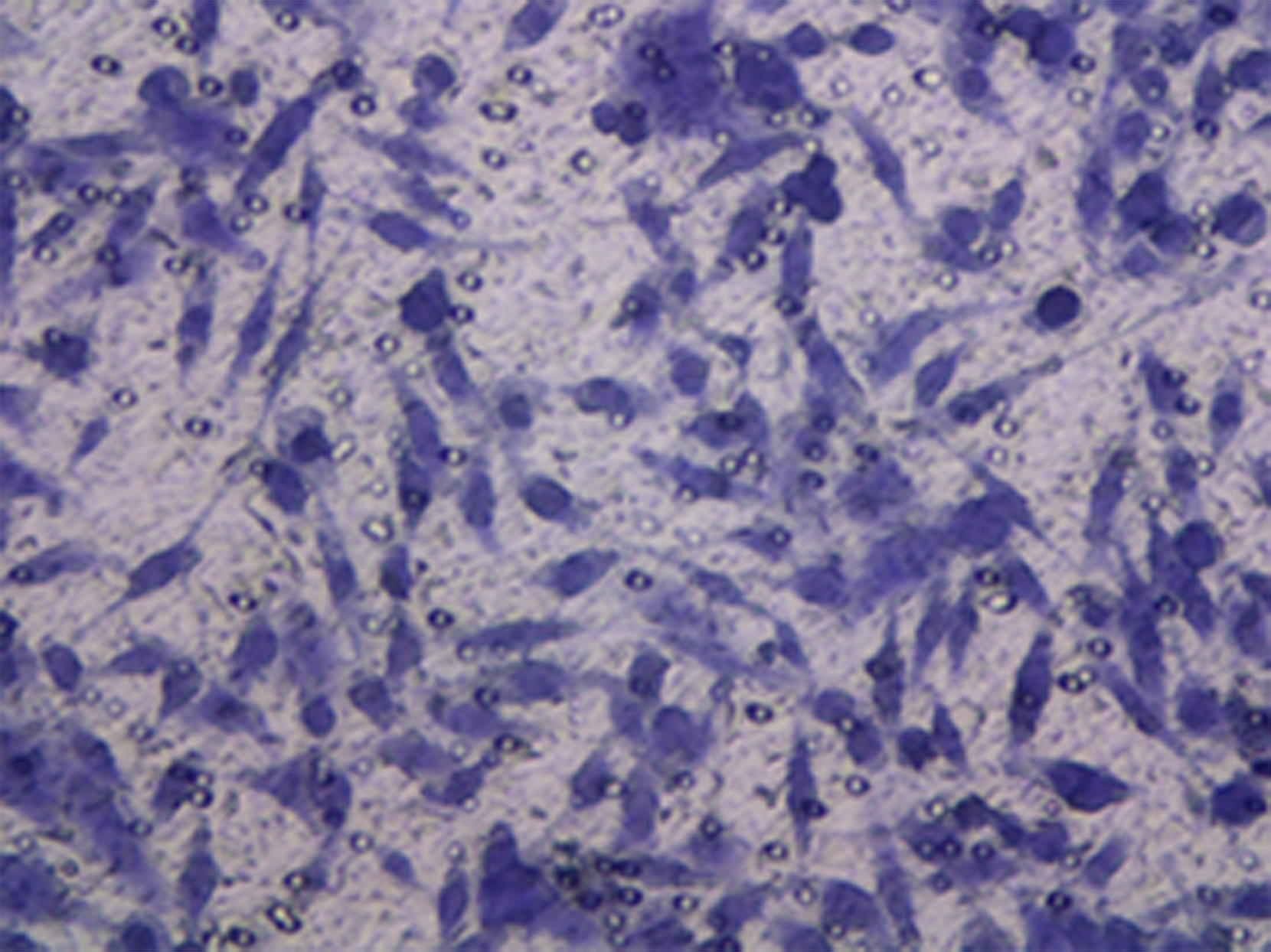

Supplement: S2 Data — (ZIP) [file pgen.1010366.s006.zip › 4C Migration 5637 METTL14 sh-USP38.png]

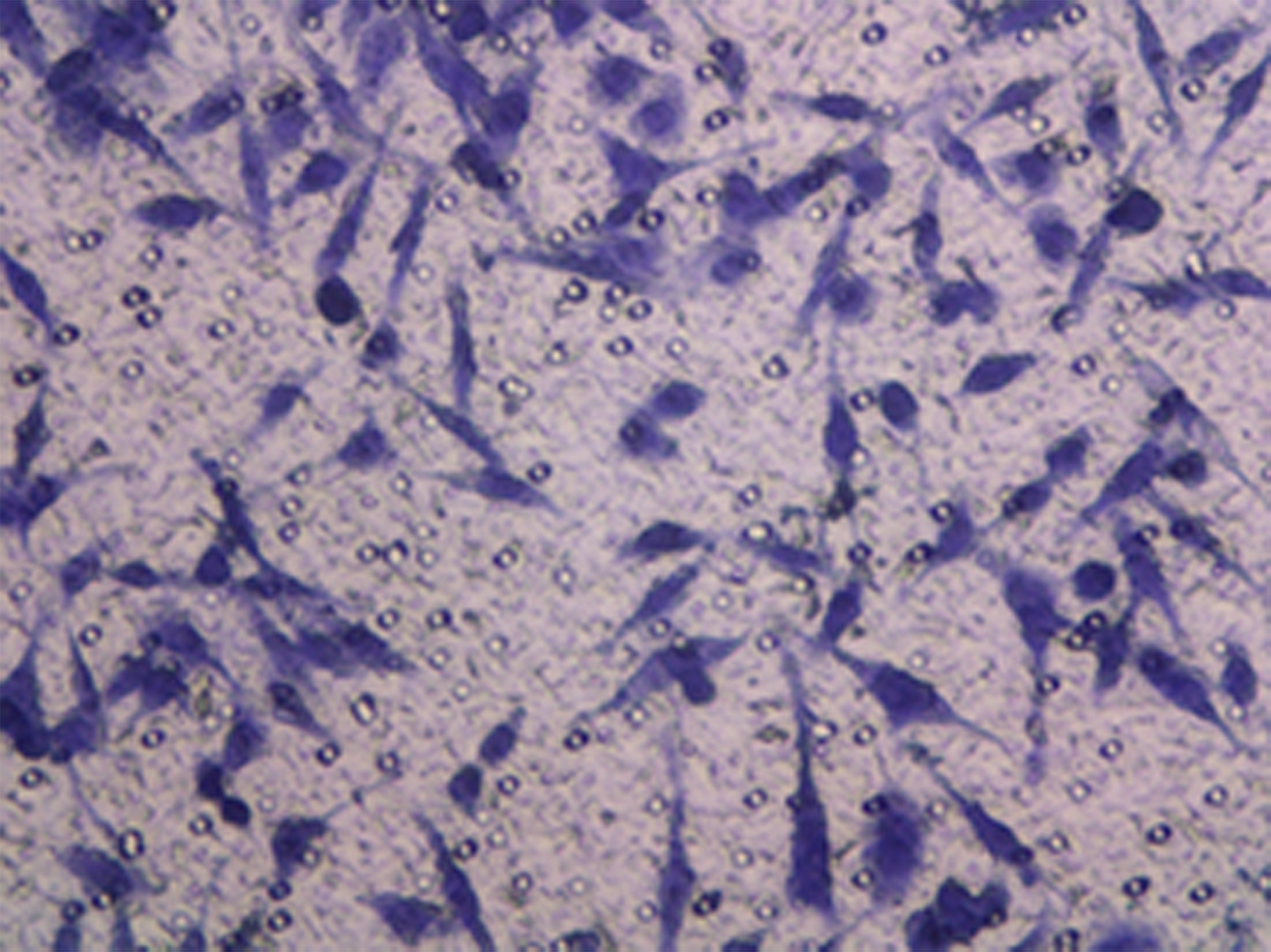

Supplement: S2 Data — (ZIP) [file pgen.1010366.s006.zip › 4C Migration 5637 METTL14.png]

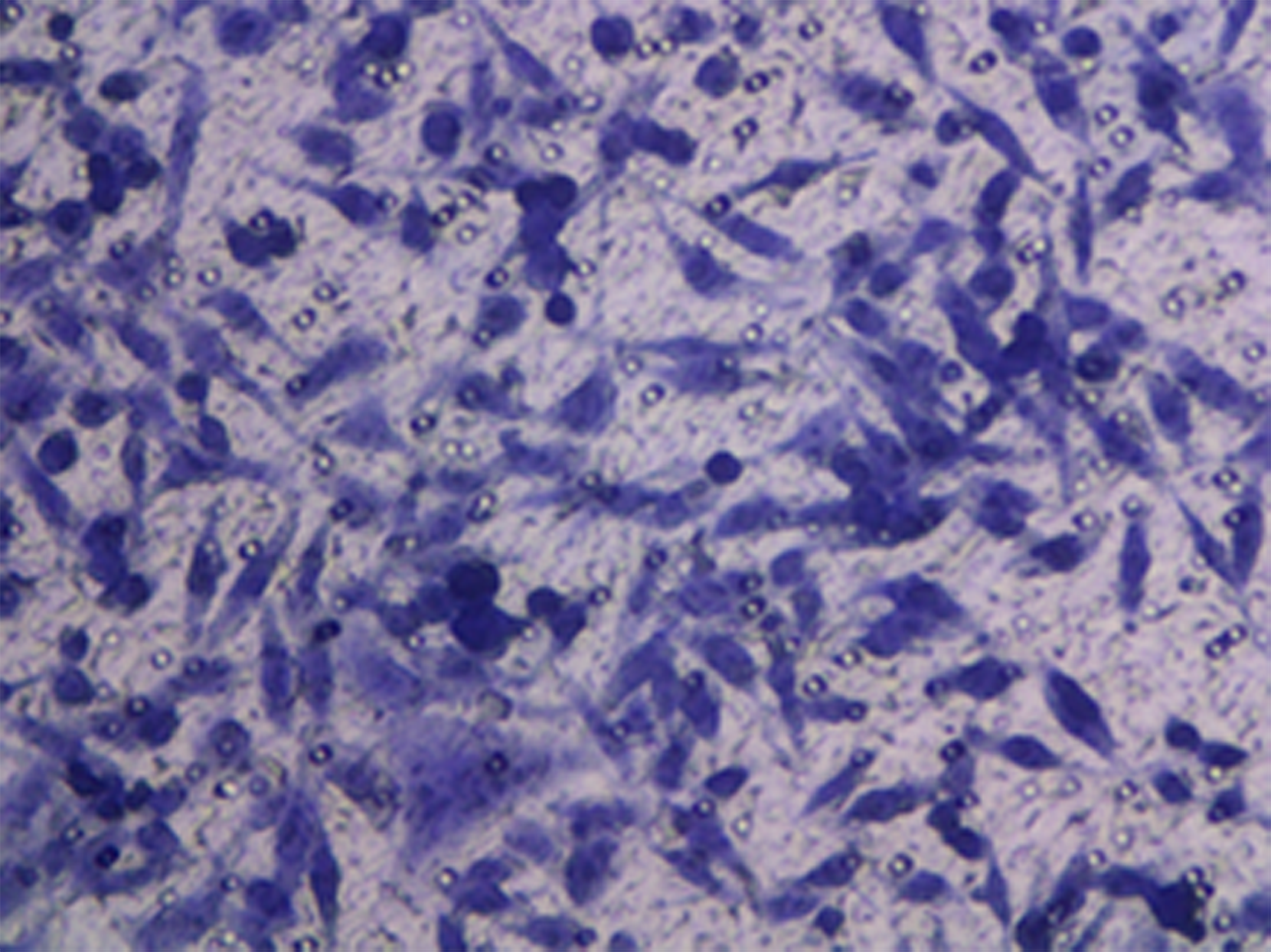

Supplement: S2 Data — (ZIP) [file pgen.1010366.s006.zip › 4C Migration 5637 Vector.png]

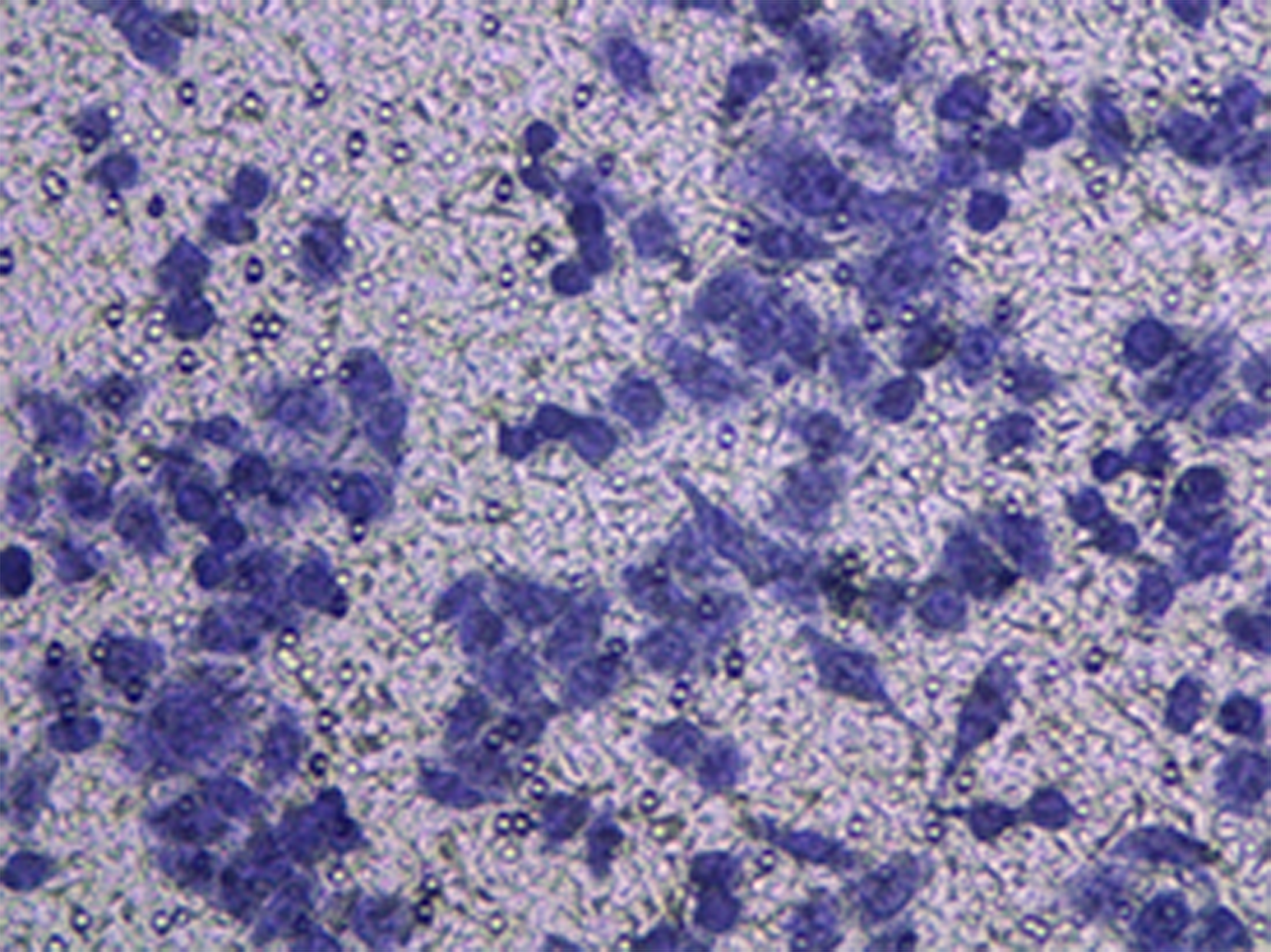

Supplement: S2 Data — (ZIP) [file pgen.1010366.s006.zip › 4C Migration T24 METTL14 sh-USP38.png]

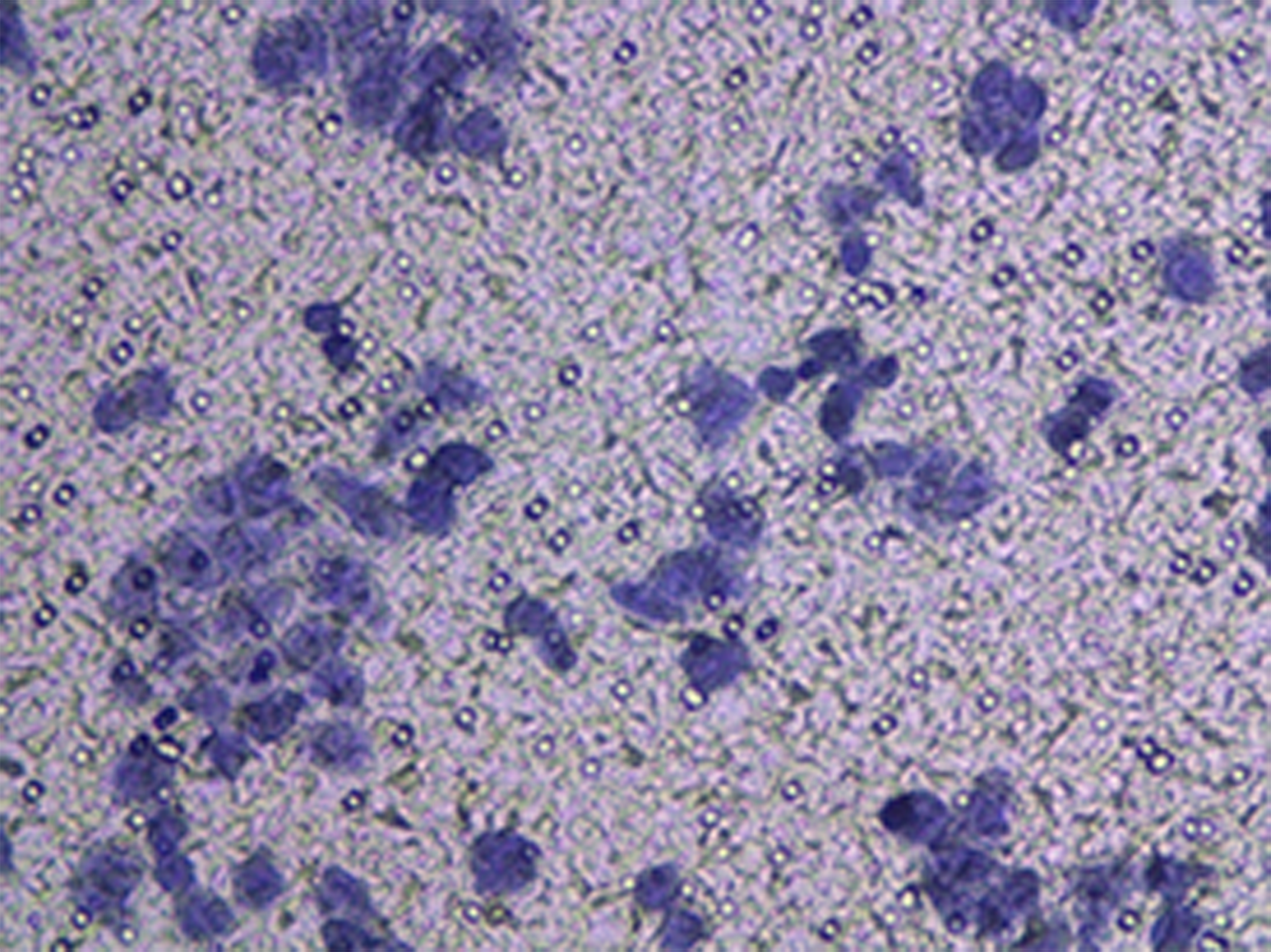

Supplement: S2 Data — (ZIP) [file pgen.1010366.s006.zip › 4C Migration T24 METTL14.png]

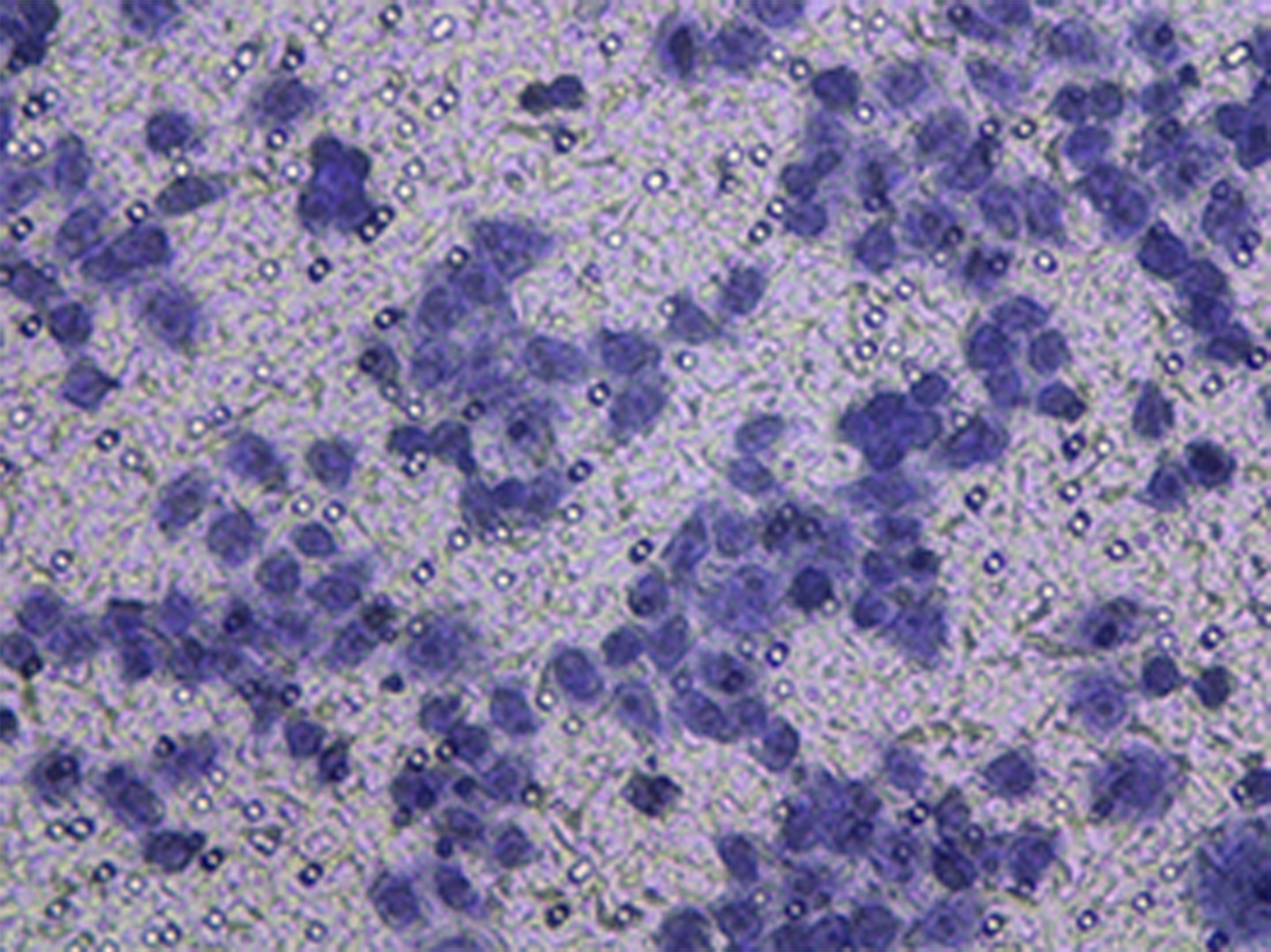

Supplement: S2 Data — (ZIP) [file pgen.1010366.s006.zip › 4C Migration T24 Vector.png]

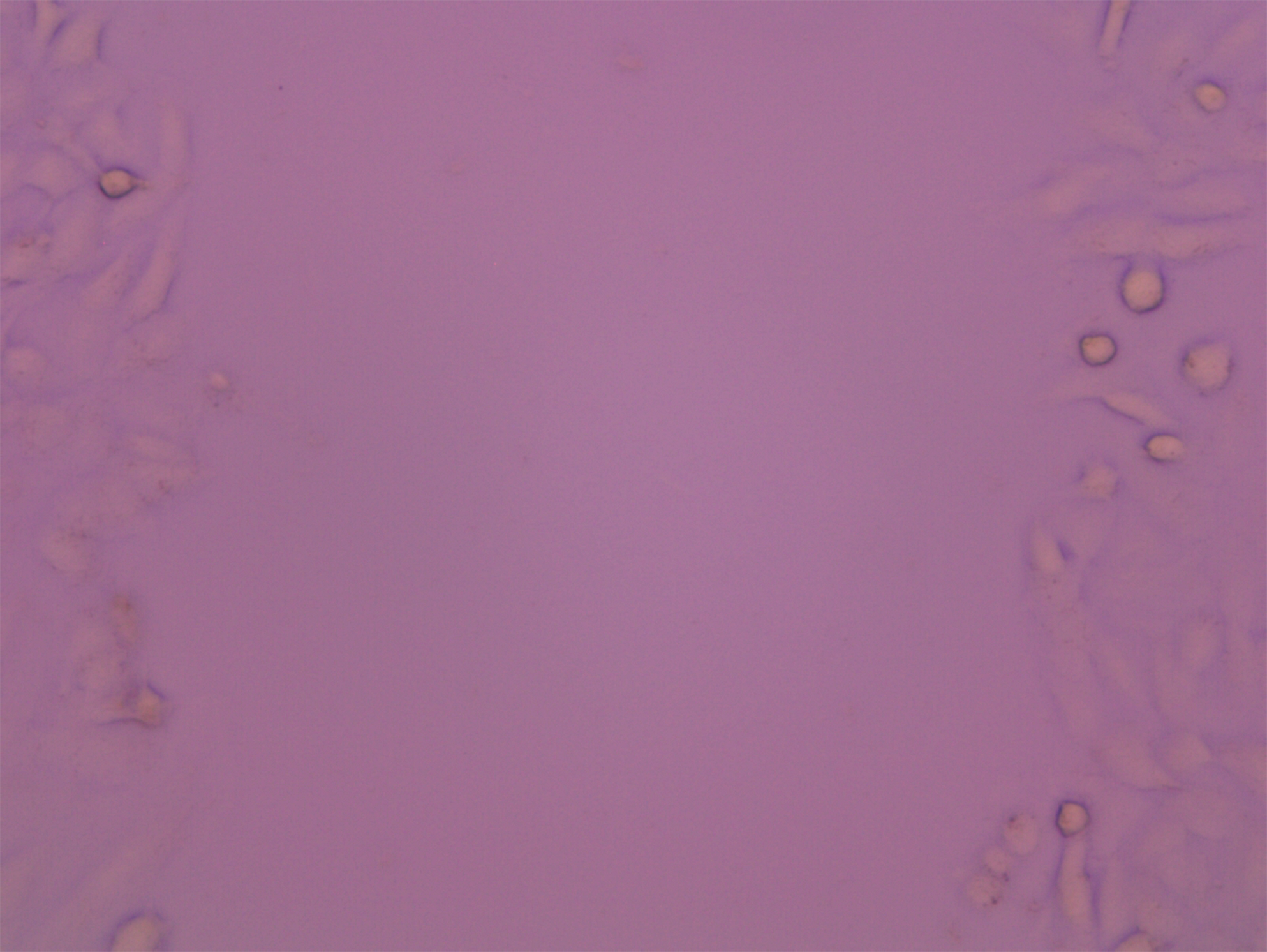

Supplement: S2 Data — (ZIP) [file pgen.1010366.s006.zip › 4D 5637 0h METTL14 sh-USP38.png]

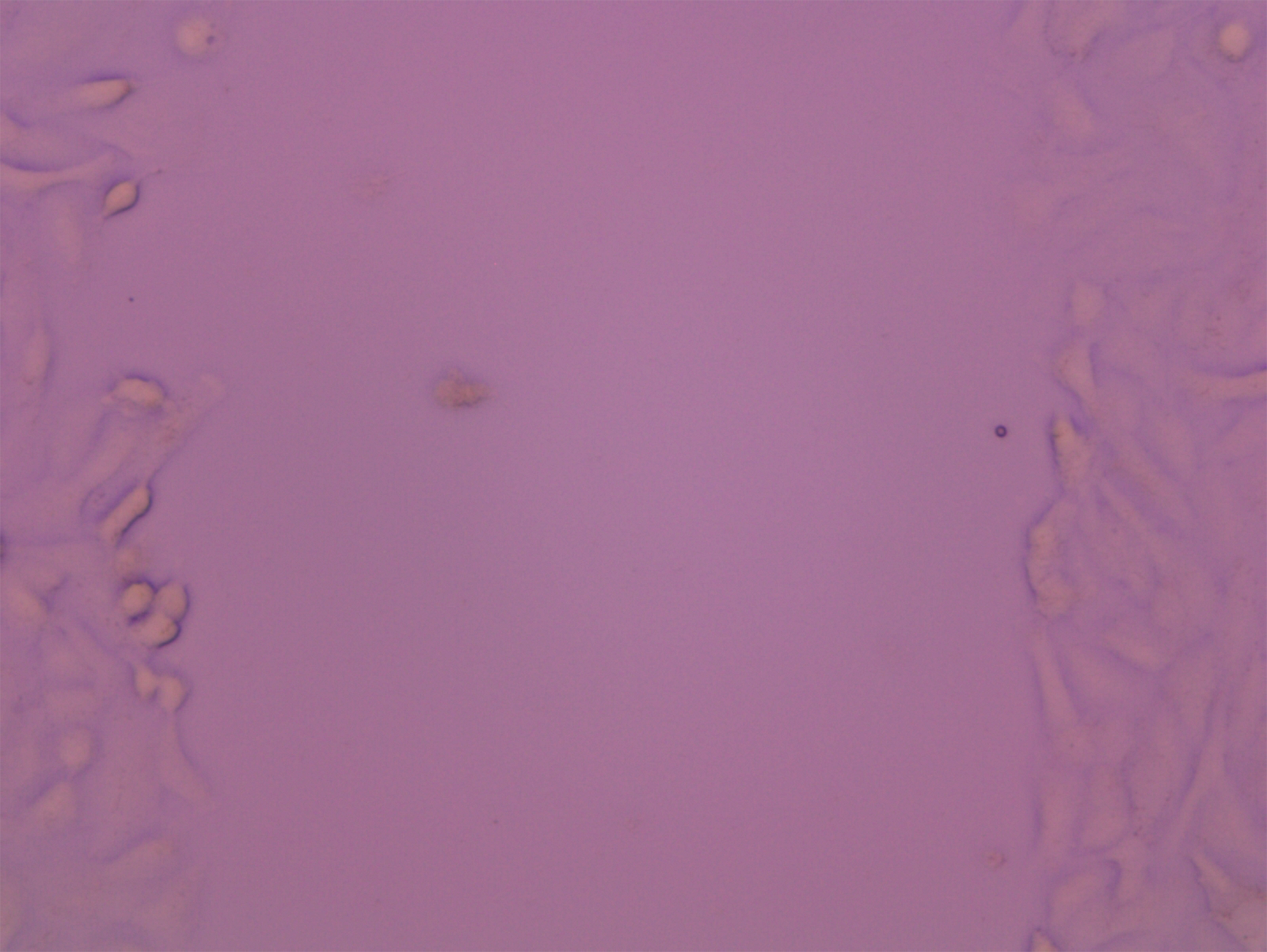

Supplement: S2 Data — (ZIP) [file pgen.1010366.s006.zip › 4D 5637 0h METTL14.png]

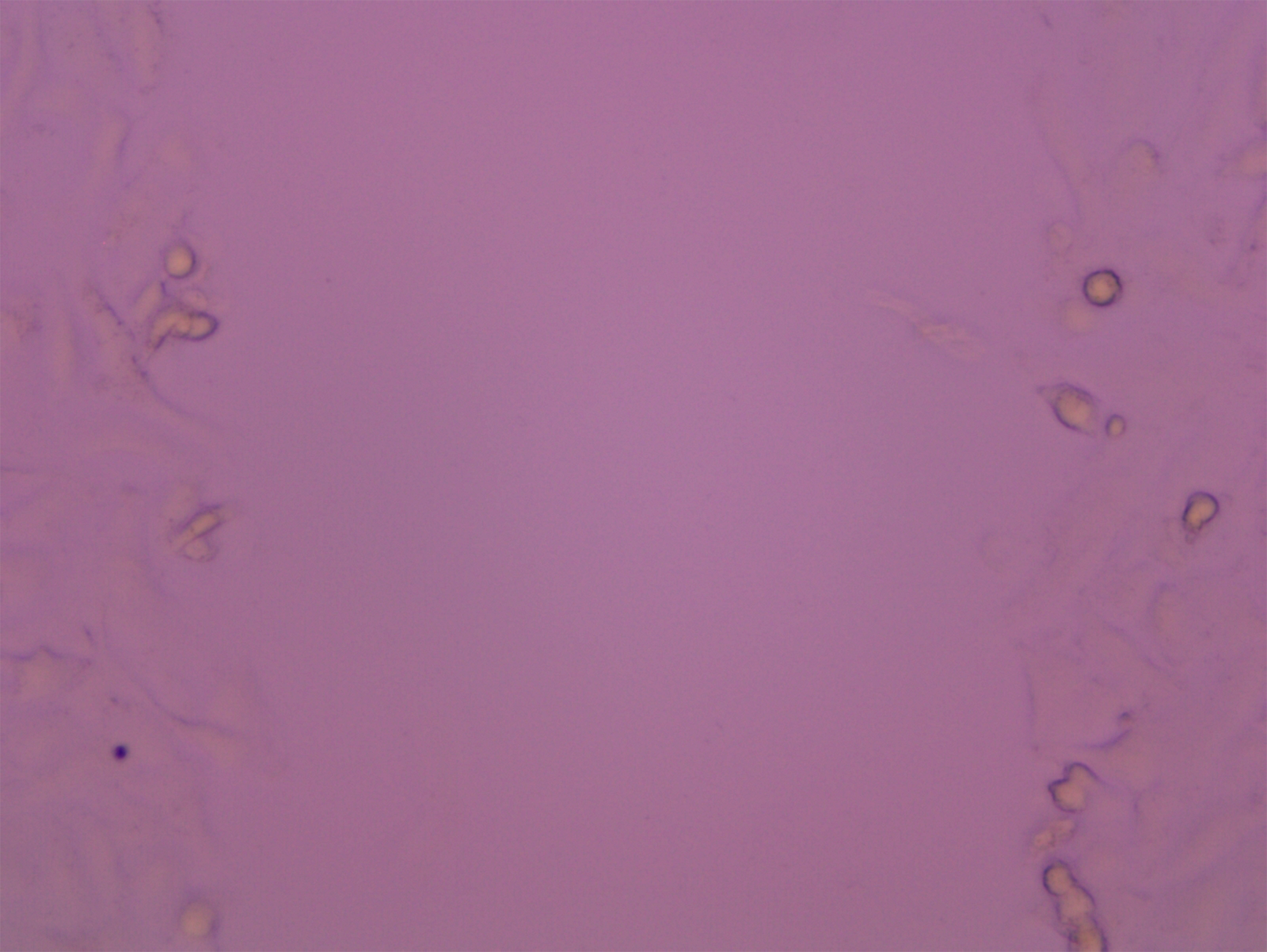

Supplement: S2 Data — (ZIP) [file pgen.1010366.s006.zip › 4D 5637 0h Vector.png]

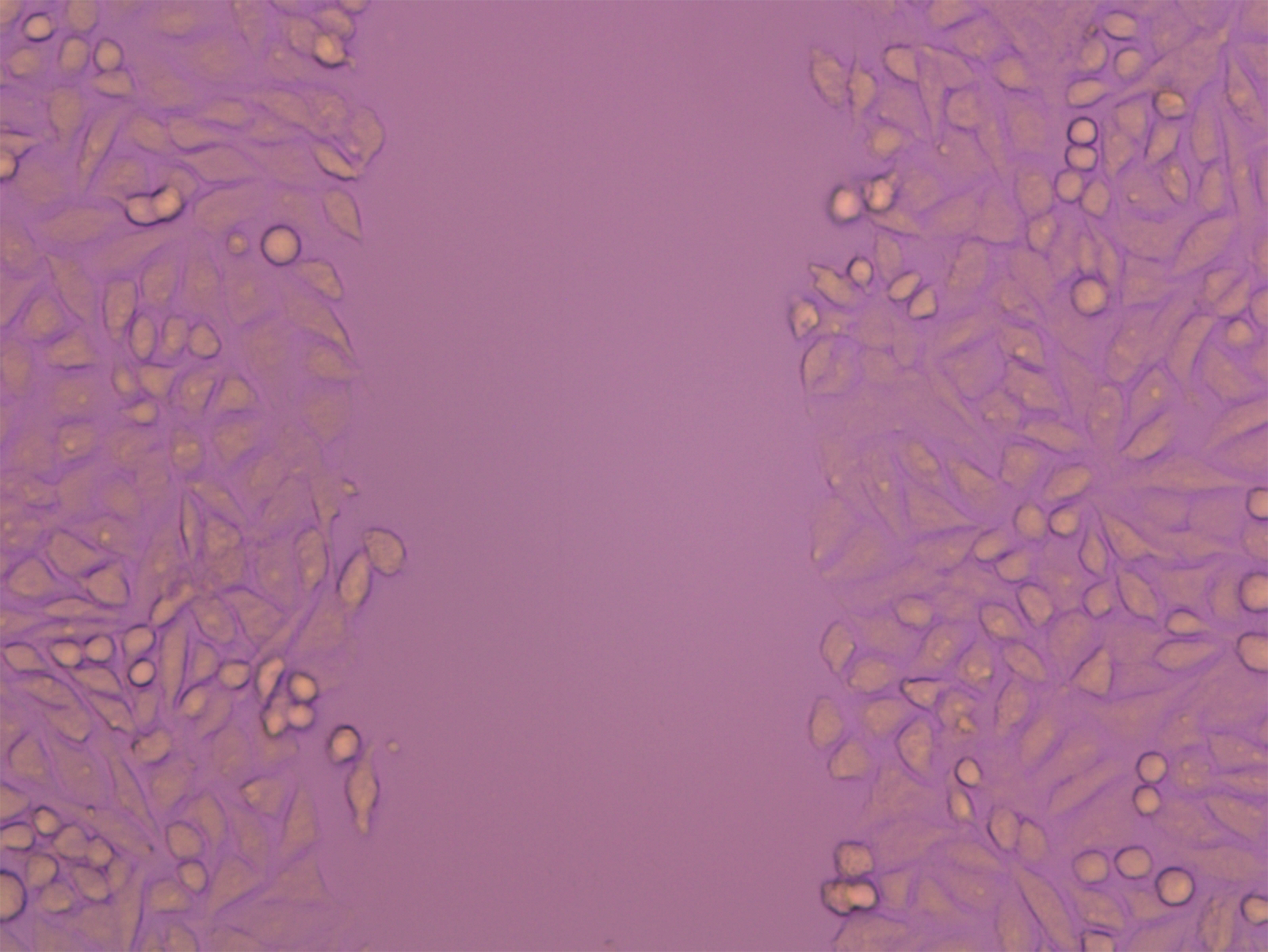

Supplement: S2 Data — (ZIP) [file pgen.1010366.s006.zip › 4D 5637 24h METTL14 sh-USP38.png]

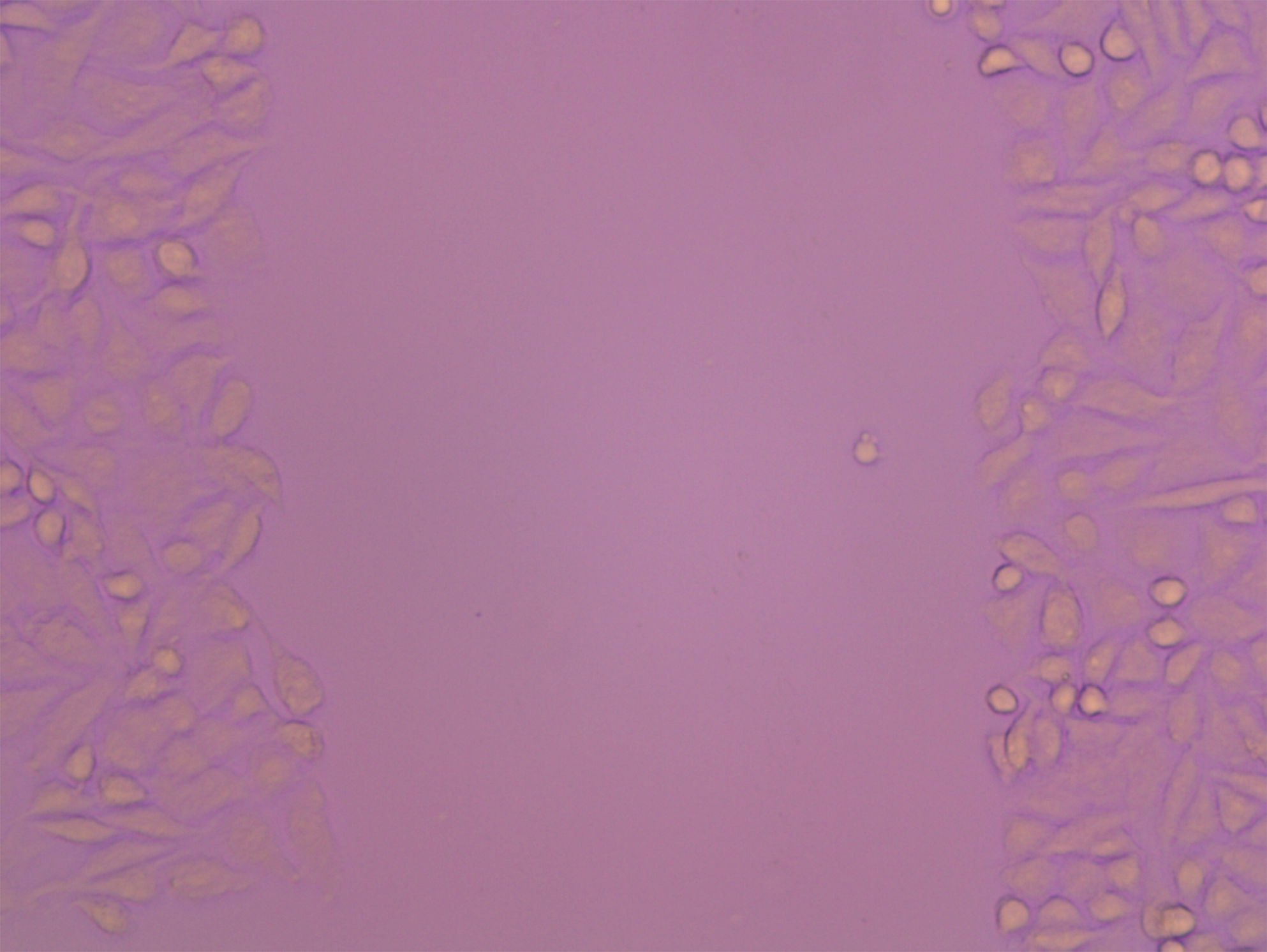

Supplement: S2 Data — (ZIP) [file pgen.1010366.s006.zip › 4D 5637 24h METTL14.png]

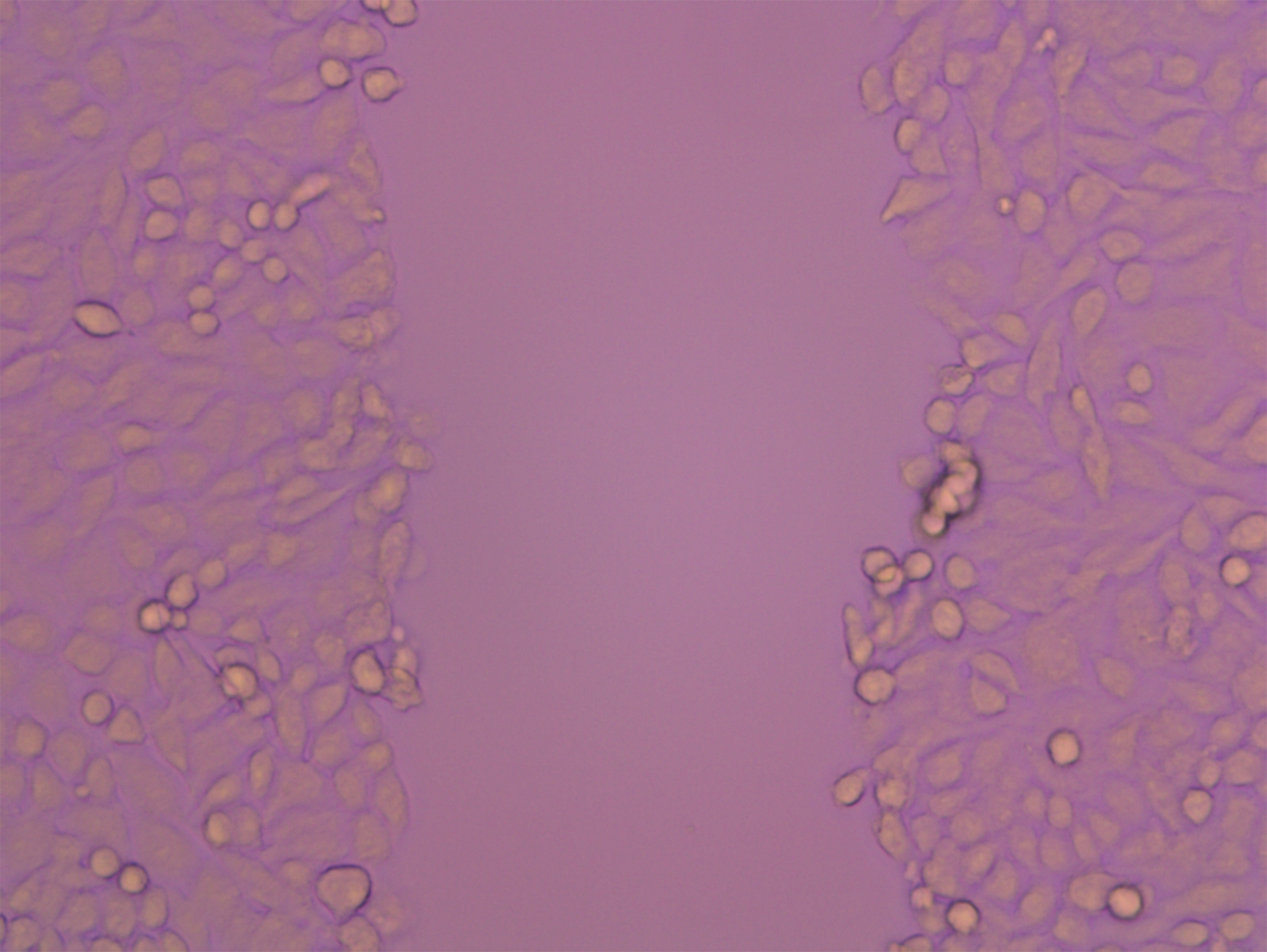

Supplement: S2 Data — (ZIP) [file pgen.1010366.s006.zip › 4D 5637 24h Vector.png]

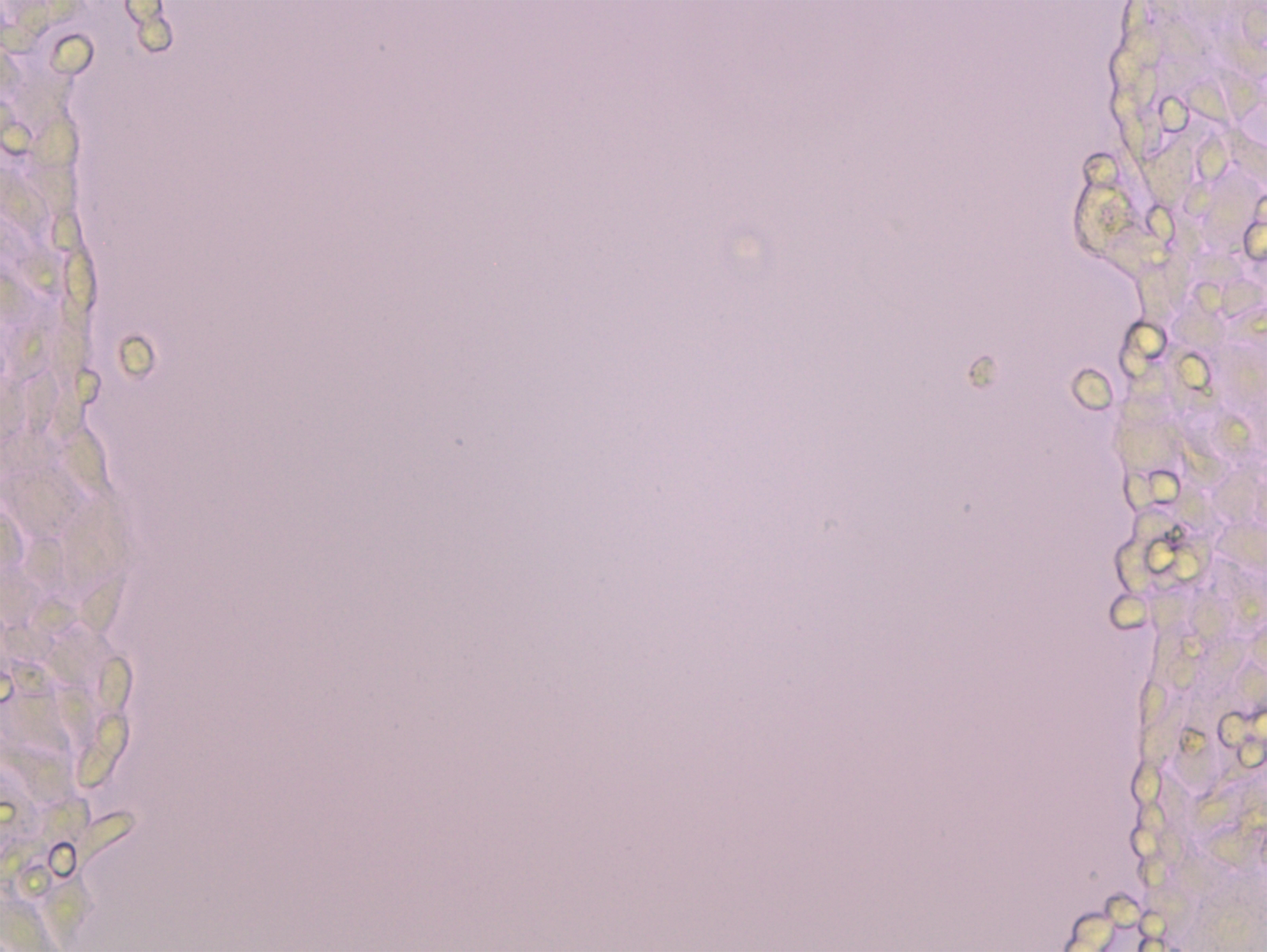

Supplement: S2 Data — (ZIP) [file pgen.1010366.s006.zip › 4D T24 0h METTL14 sh-USP38.png]

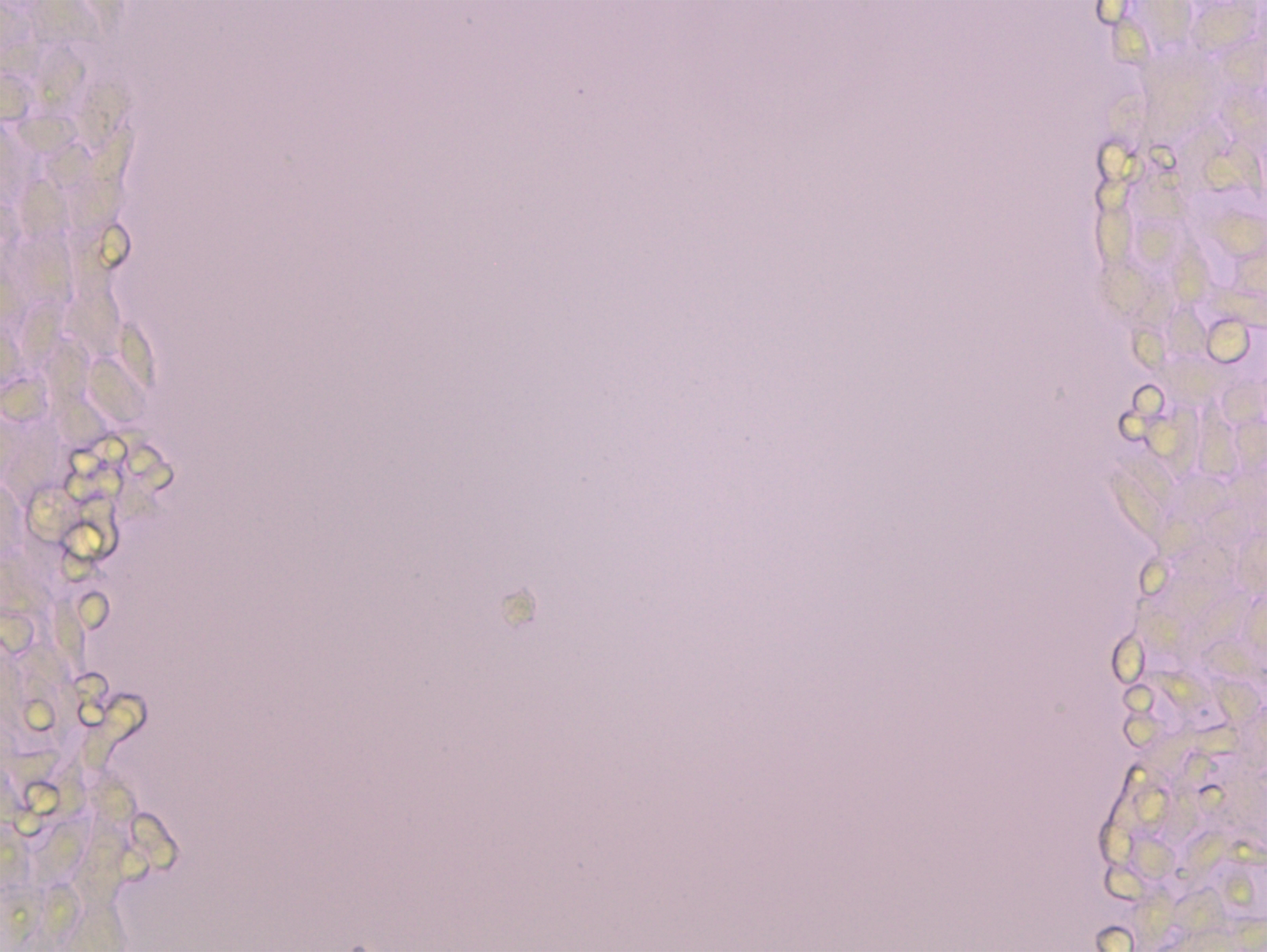

Supplement: S2 Data — (ZIP) [file pgen.1010366.s006.zip › 4D T24 0h METTL14.png]

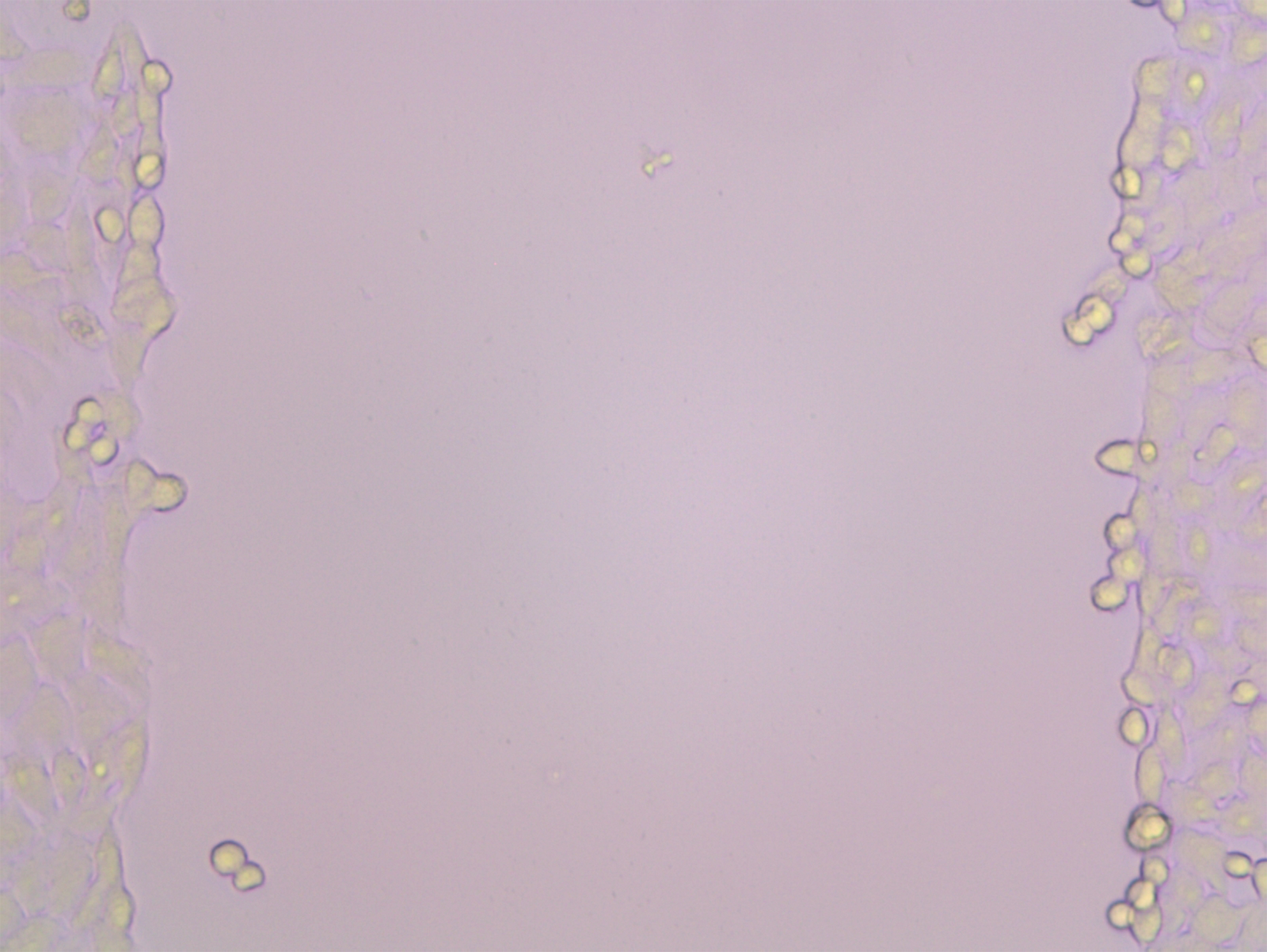

Supplement: S2 Data — (ZIP) [file pgen.1010366.s006.zip › 4D T24 0h Vector.png]

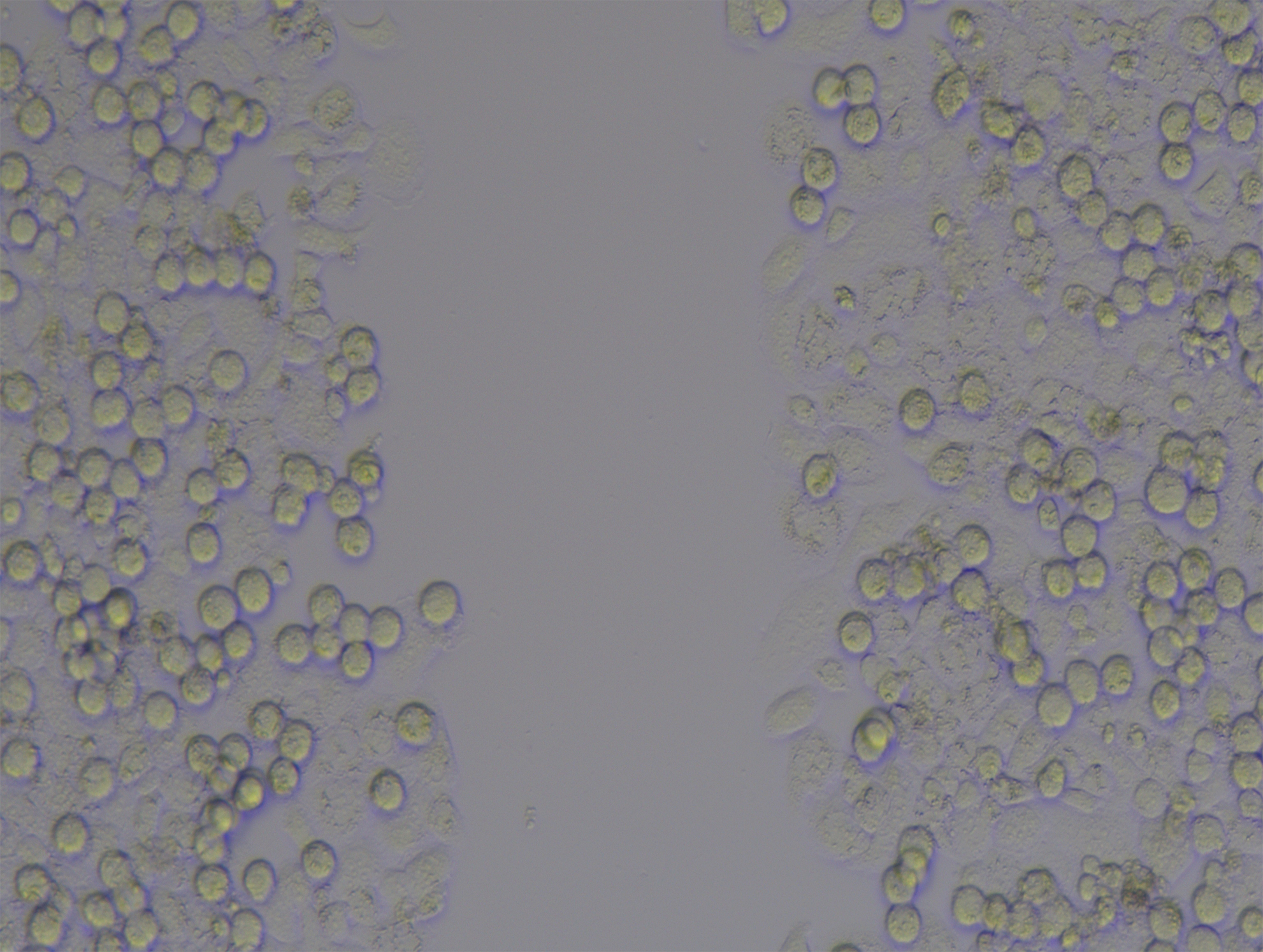

Supplement: S2 Data — (ZIP) [file pgen.1010366.s006.zip › 4D T24 24h METTL14 sh-USP38.png]

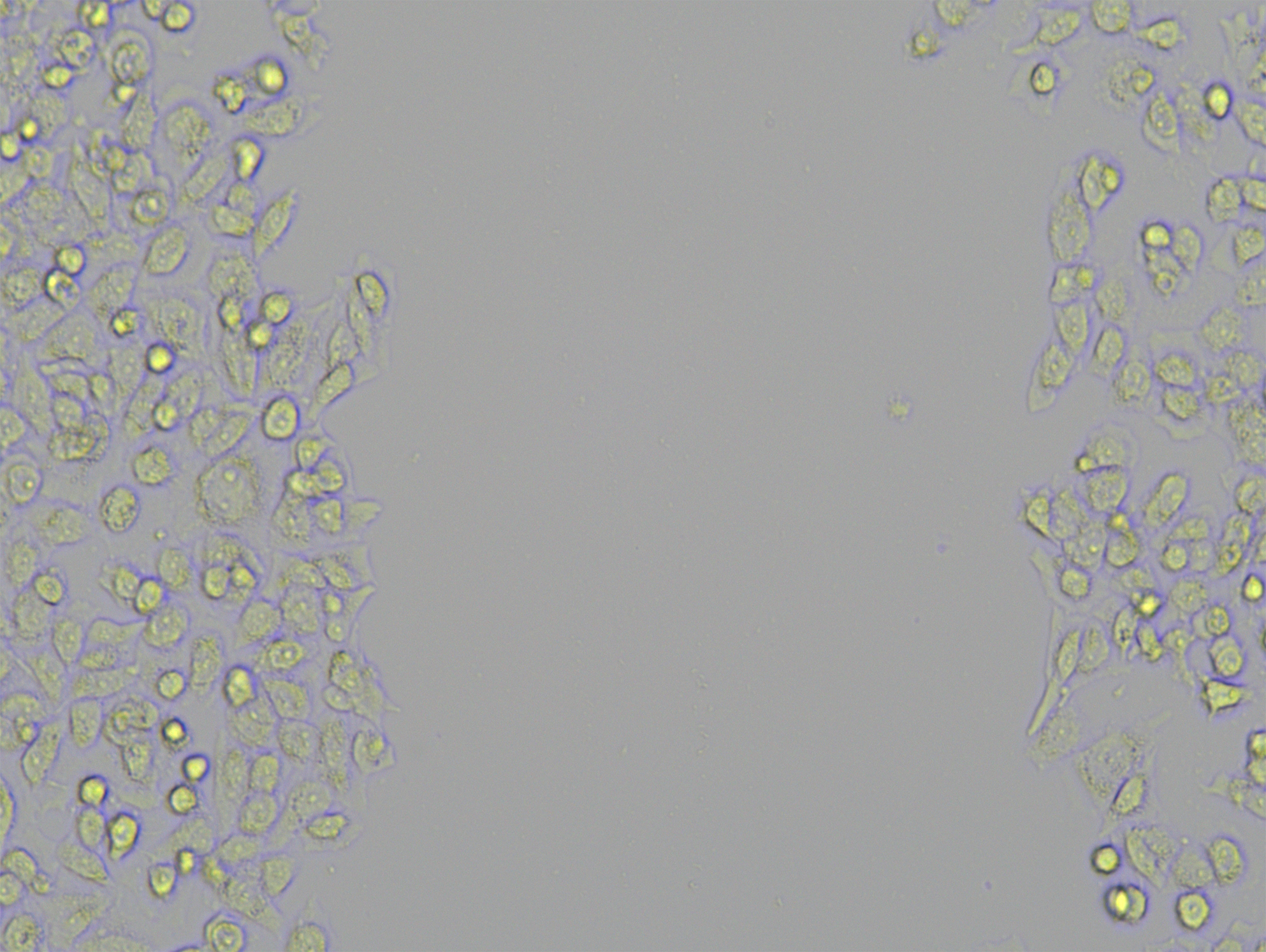

Supplement: S2 Data — (ZIP) [file pgen.1010366.s006.zip › 4D T24 24h METTL14.png]

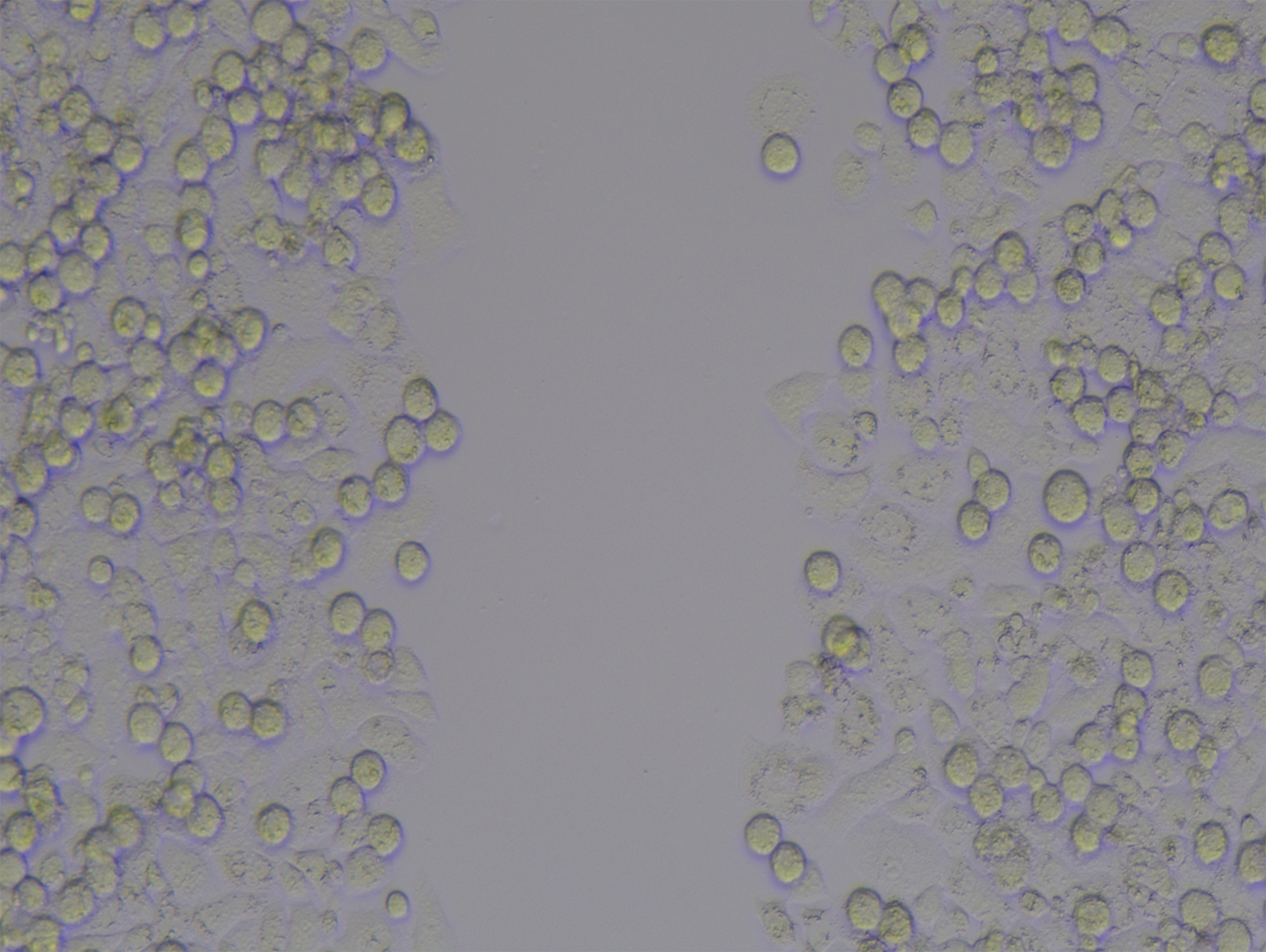

Supplement: S2 Data — (ZIP) [file pgen.1010366.s006.zip › 4D T24 24h Vector.png]

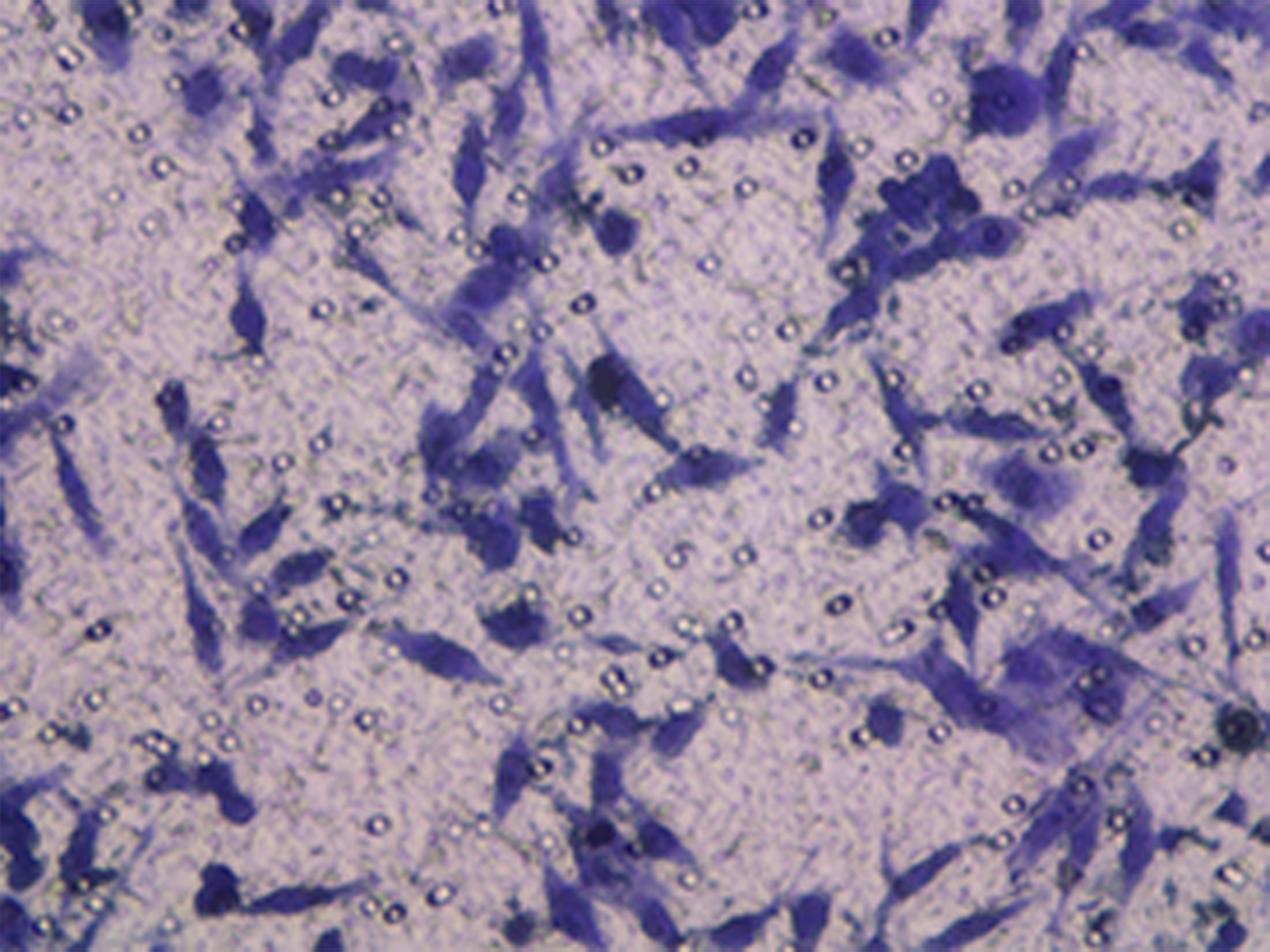

Supplement: S2 Data — (ZIP) [file pgen.1010366.s006.zip › 4E Invasion 5637 METTL14 sh-USP38.png]

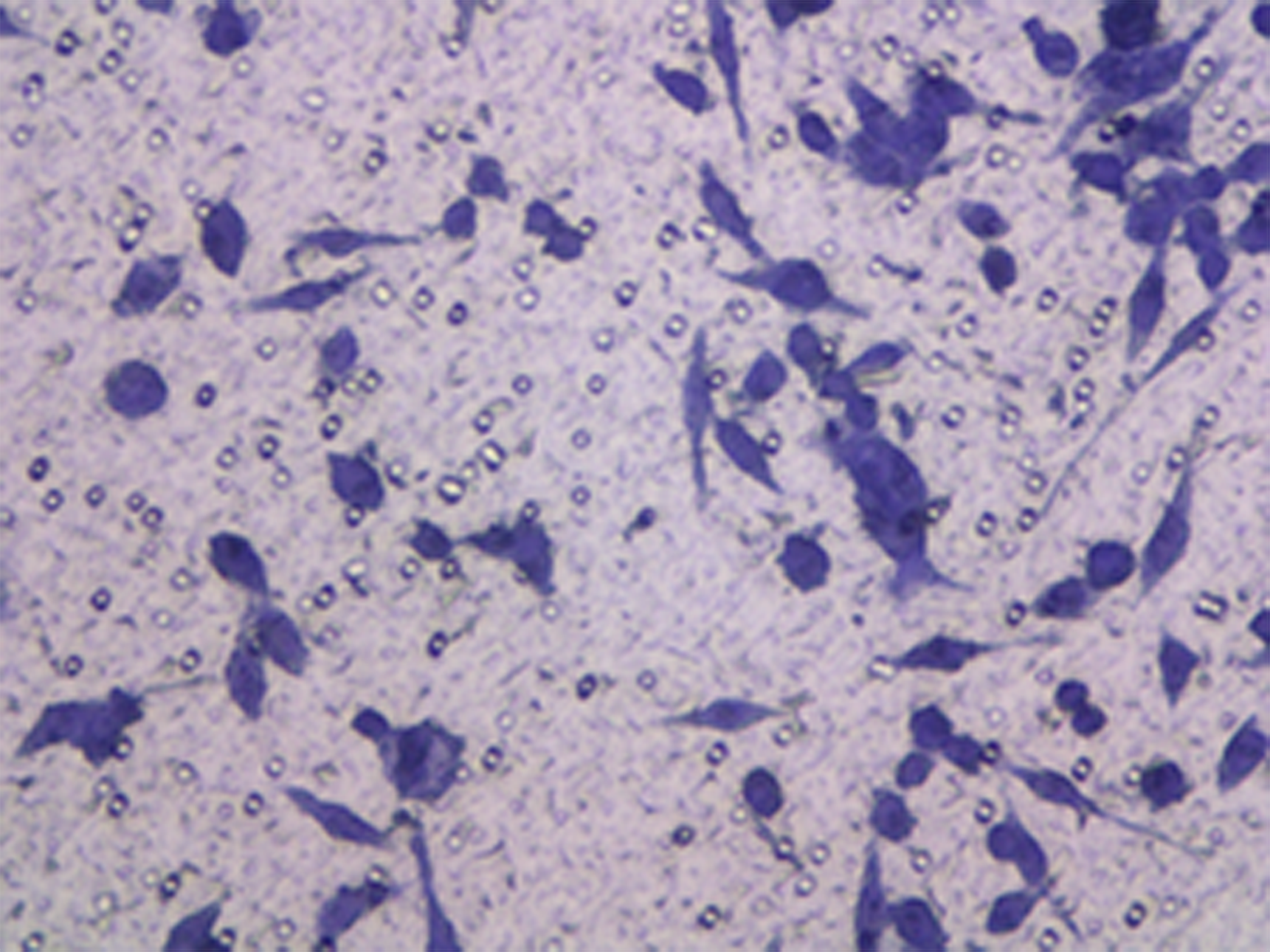

Supplement: S2 Data — (ZIP) [file pgen.1010366.s006.zip › 4E Invasion 5637 METTL14.png]

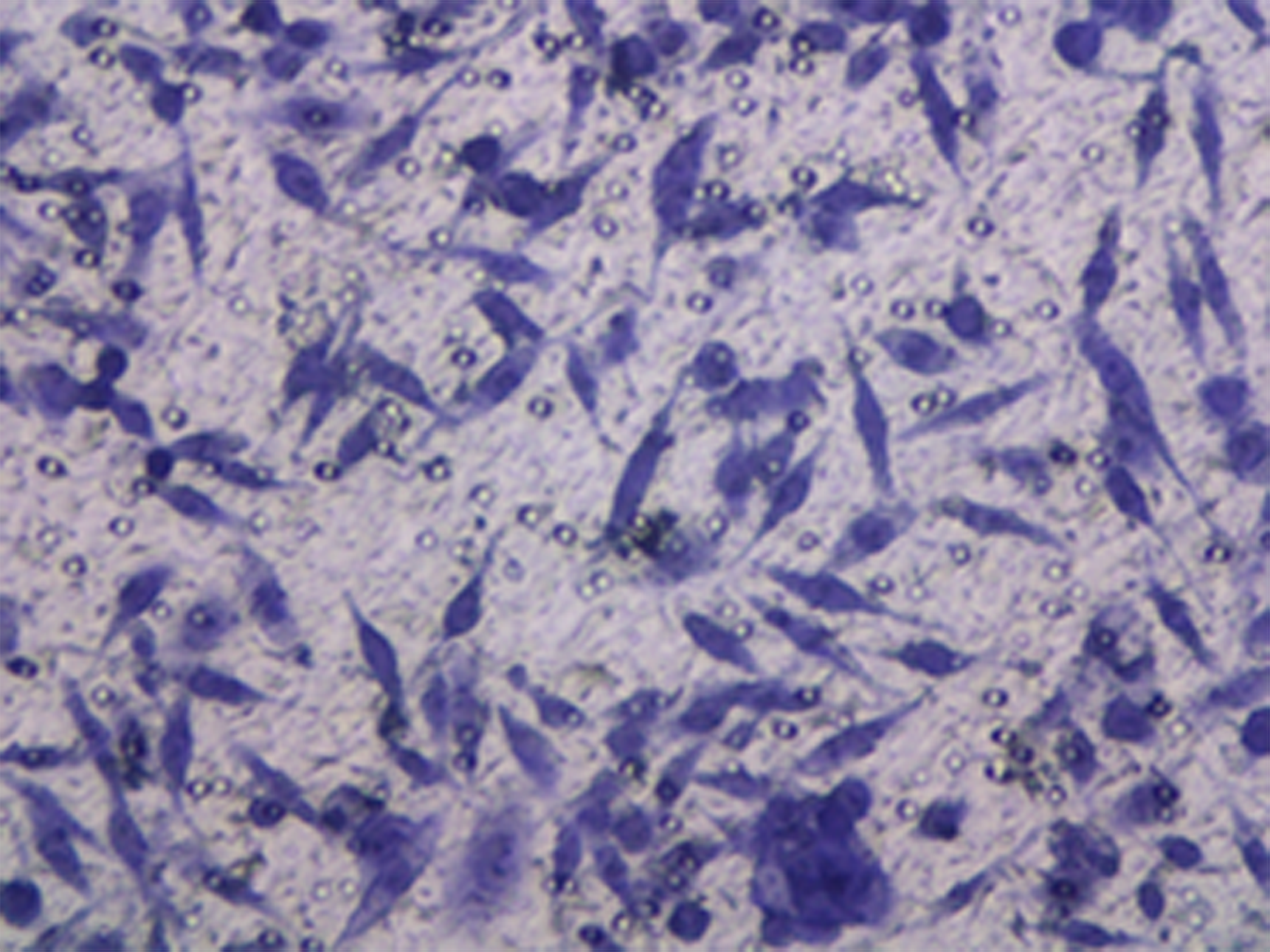

Supplement: S2 Data — (ZIP) [file pgen.1010366.s006.zip › 4E Invasion 5637 Vector.png]

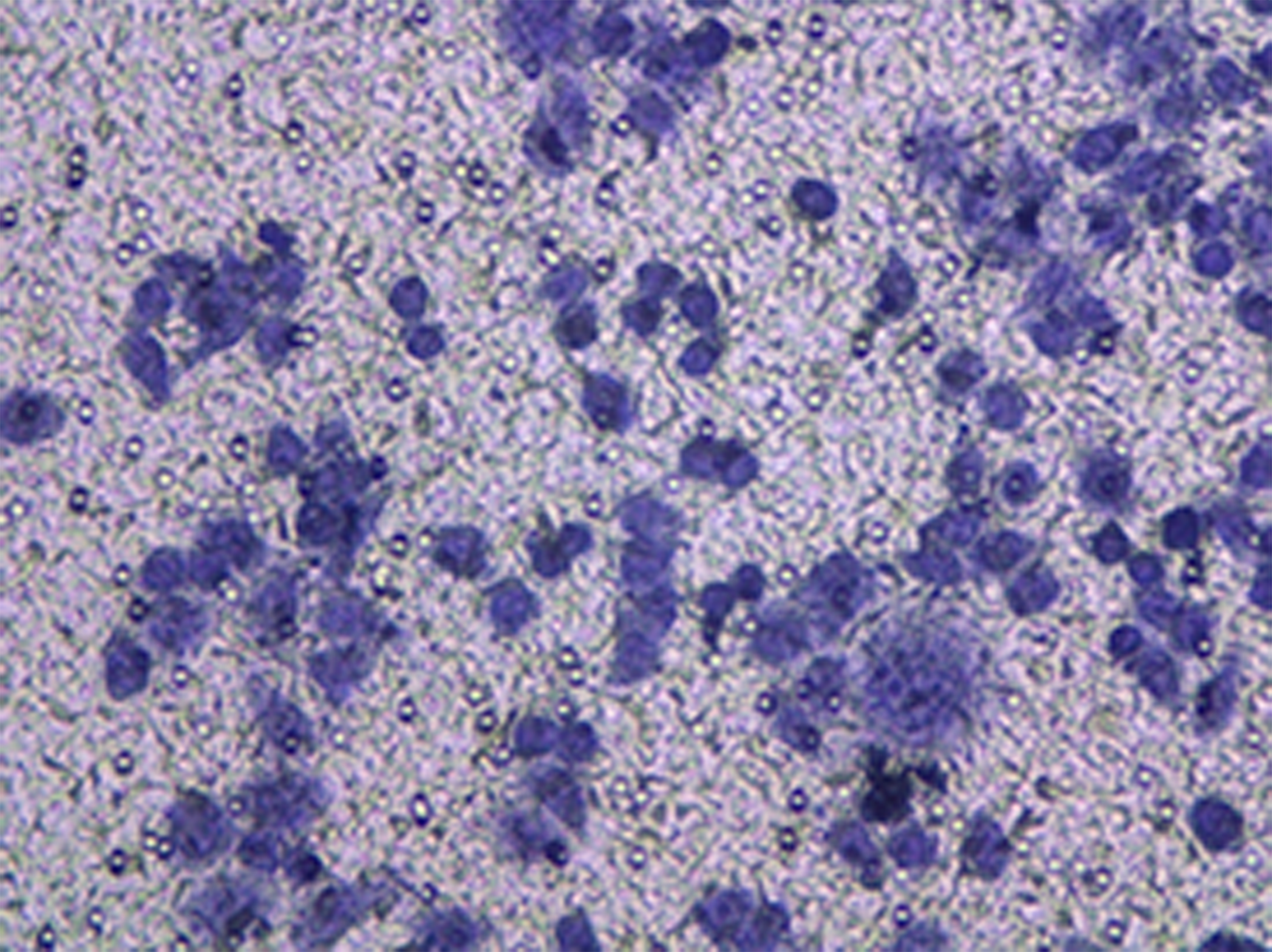

Supplement: S2 Data — (ZIP) [file pgen.1010366.s006.zip › 4E Invasion T24 METTL14 sh-USP38.png]

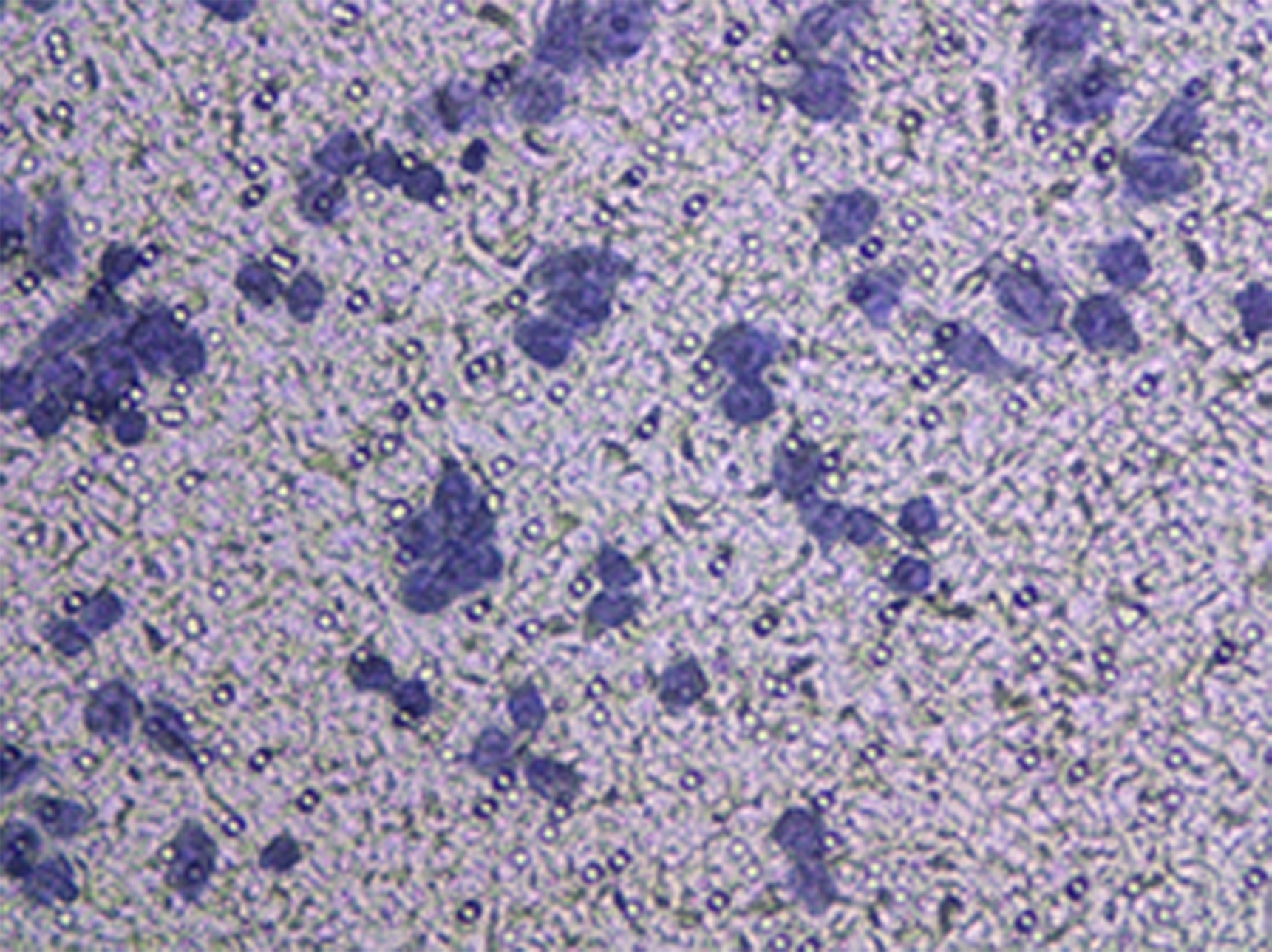

Supplement: S2 Data — (ZIP) [file pgen.1010366.s006.zip › 4E Invasion T24 METTL14.png]

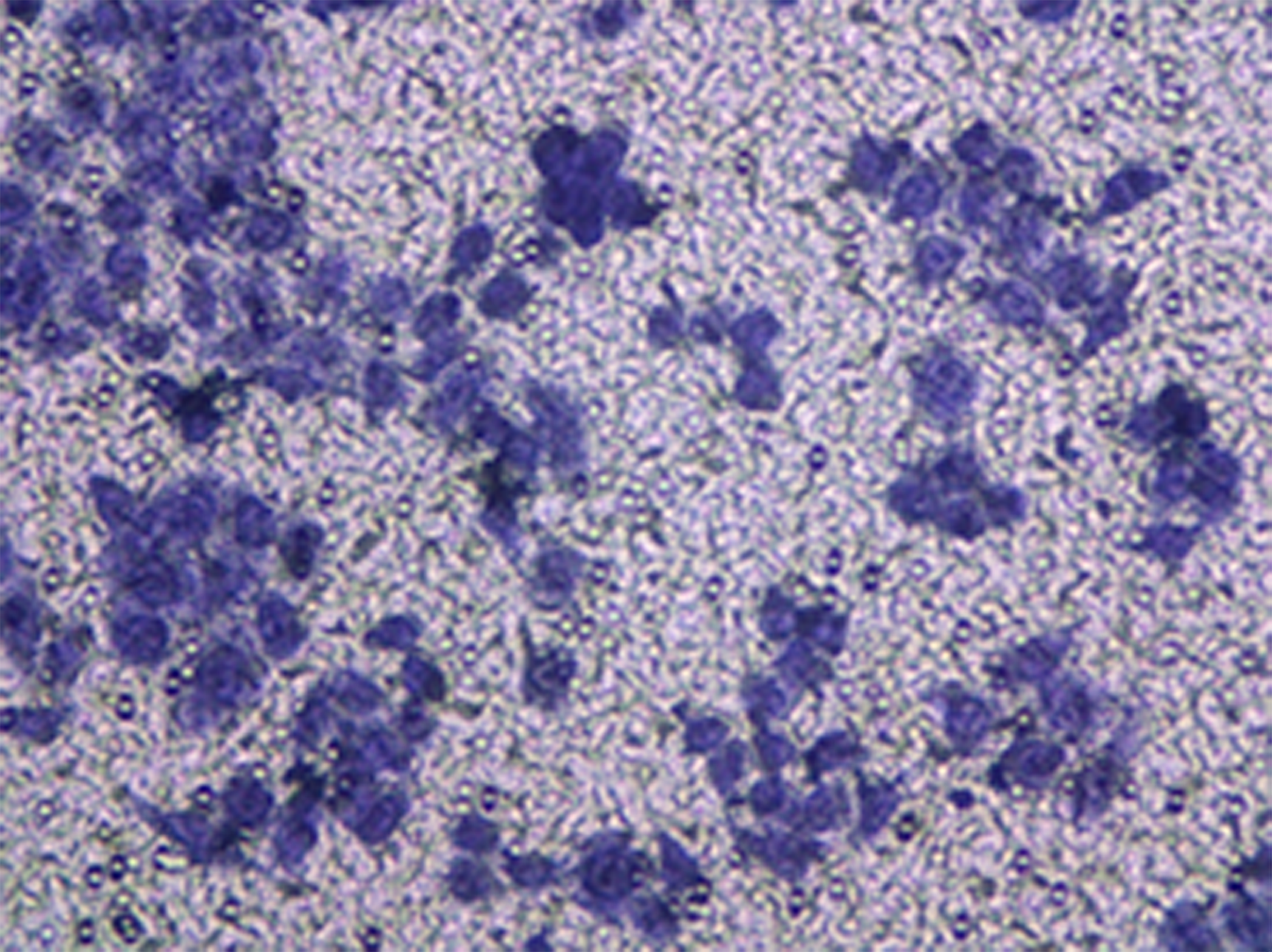

Supplement: S2 Data — (ZIP) [file pgen.1010366.s006.zip › 4E Invasion T24 Vector.png]

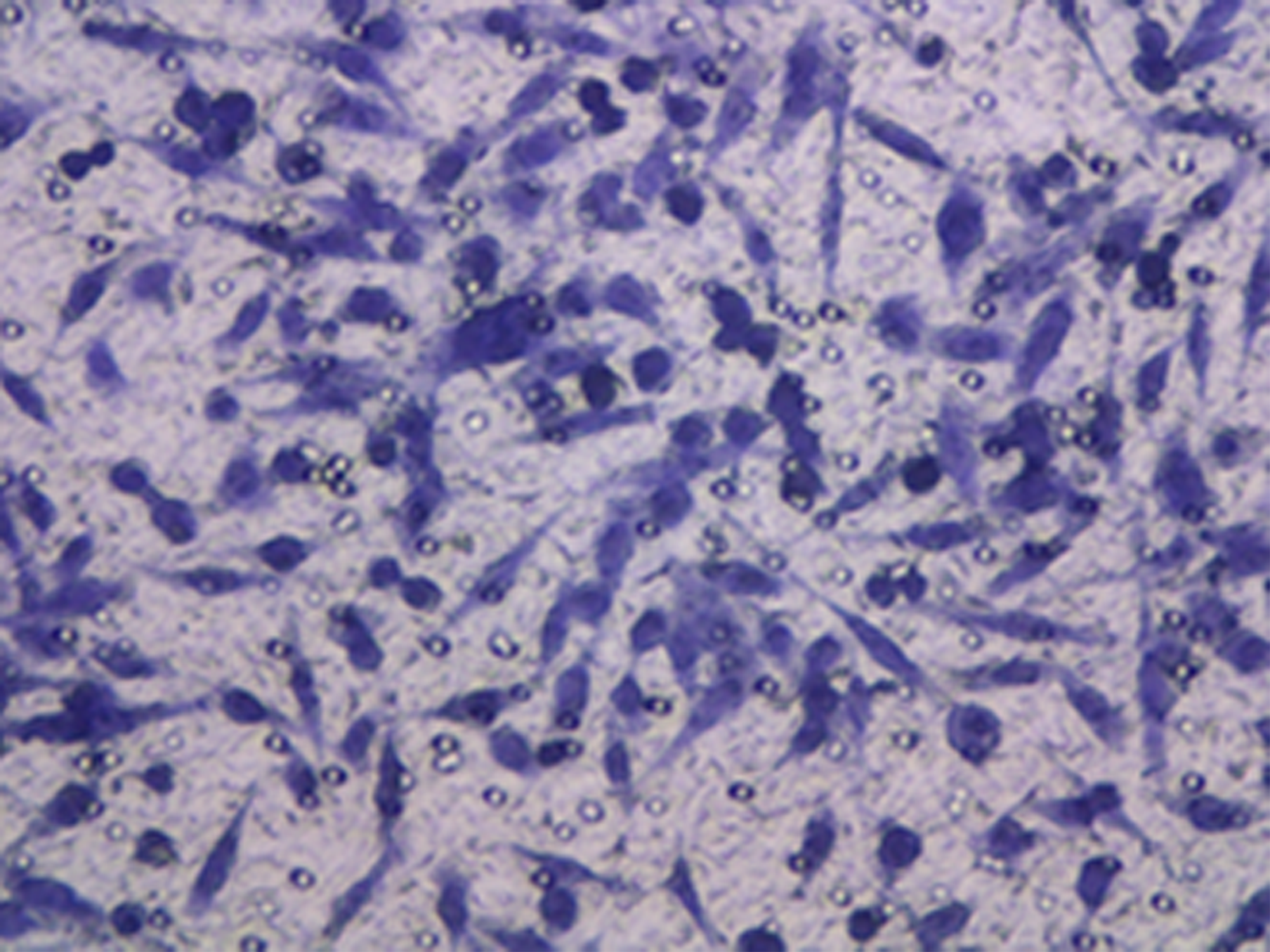

Supplement: S3 Data — (ZIP) [file pgen.1010366.s007.zip › 7B Migration 5637 miR-3165 inhibitor sh-METTL14.png]

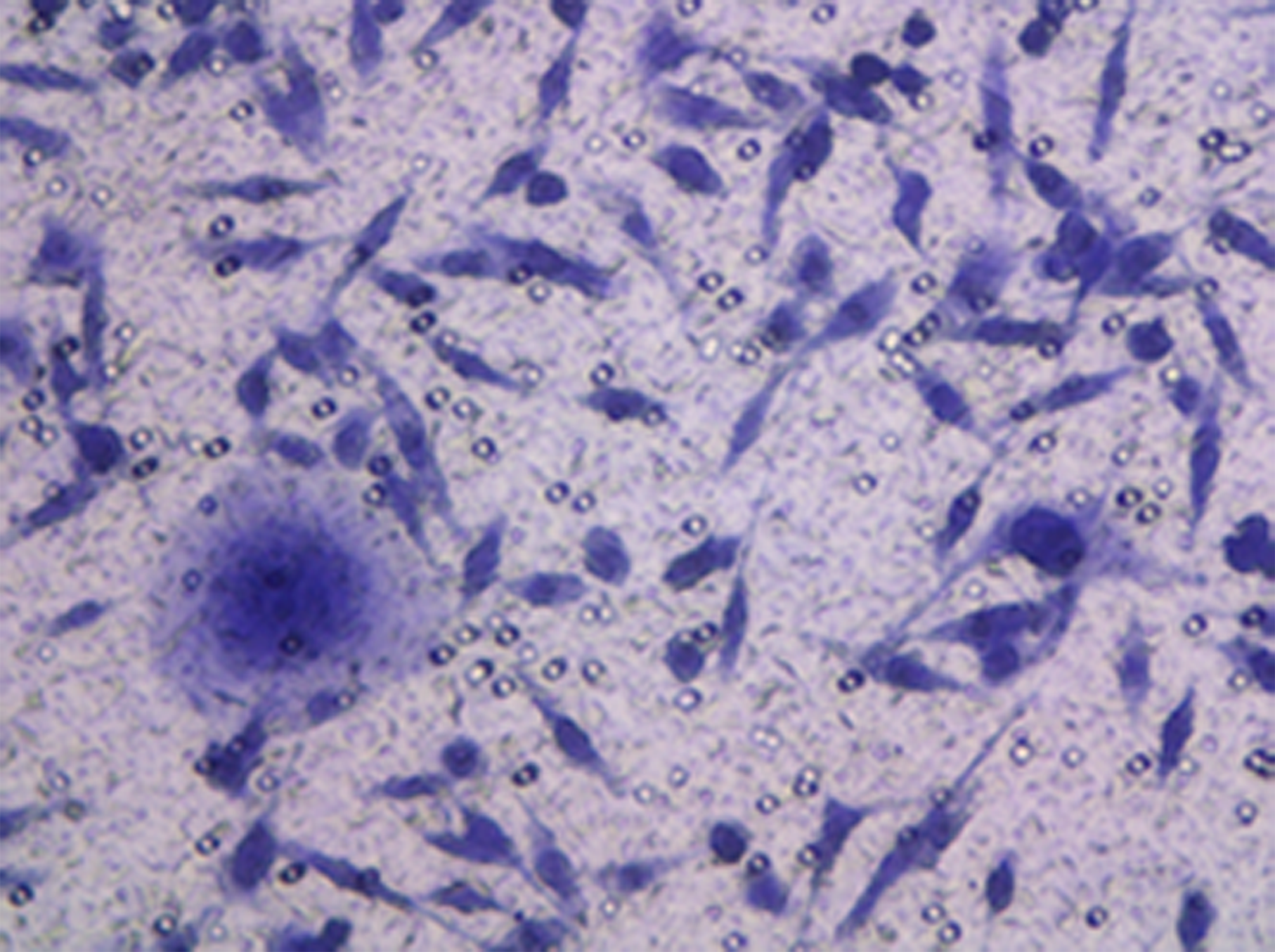

Supplement: S3 Data — (ZIP) [file pgen.1010366.s007.zip › 7B Migration 5637 miR-3165 inhibitor.png]

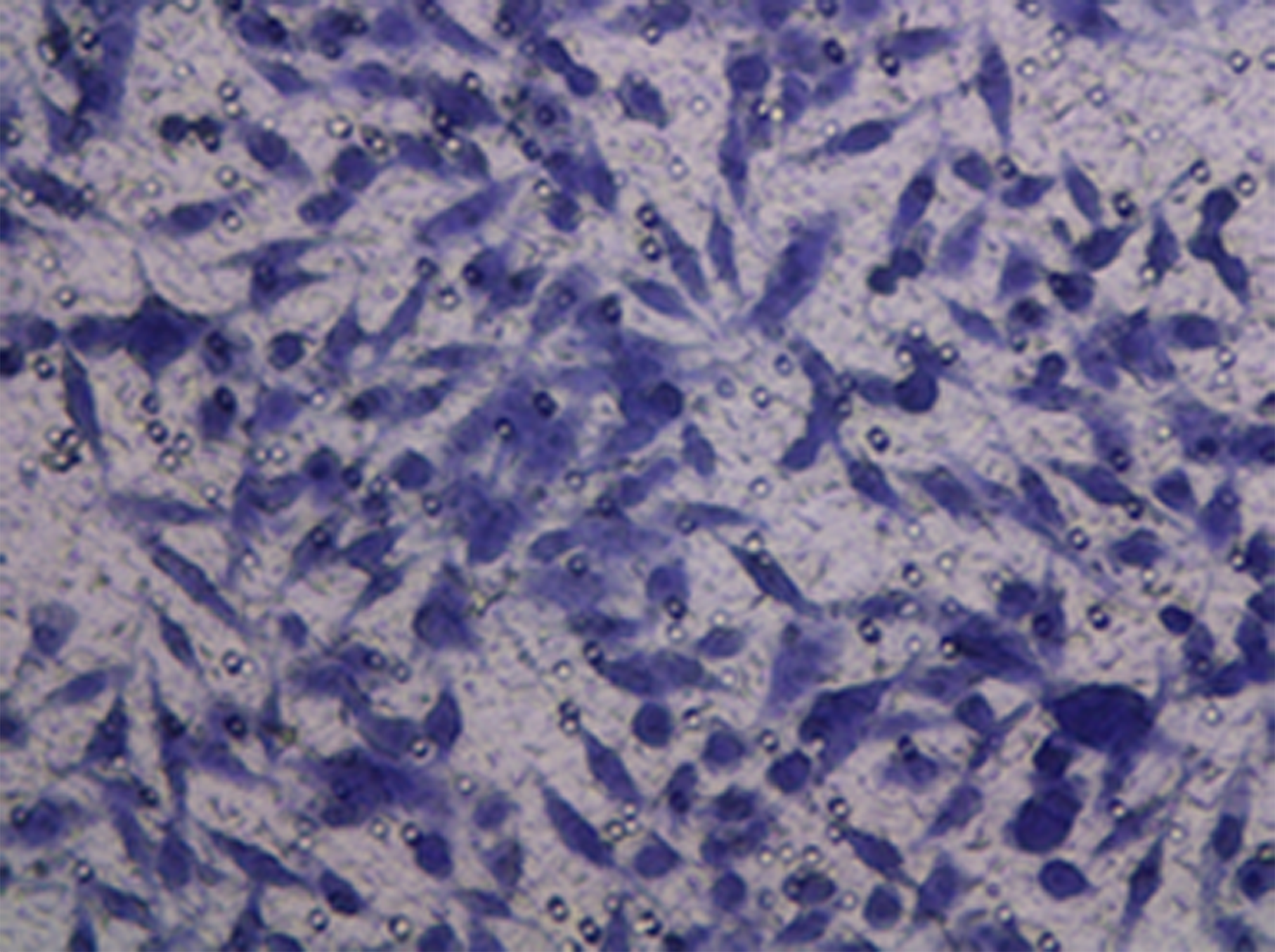

Supplement: S3 Data — (ZIP) [file pgen.1010366.s007.zip › 7B Migration 5637 NC inhibitor.png]

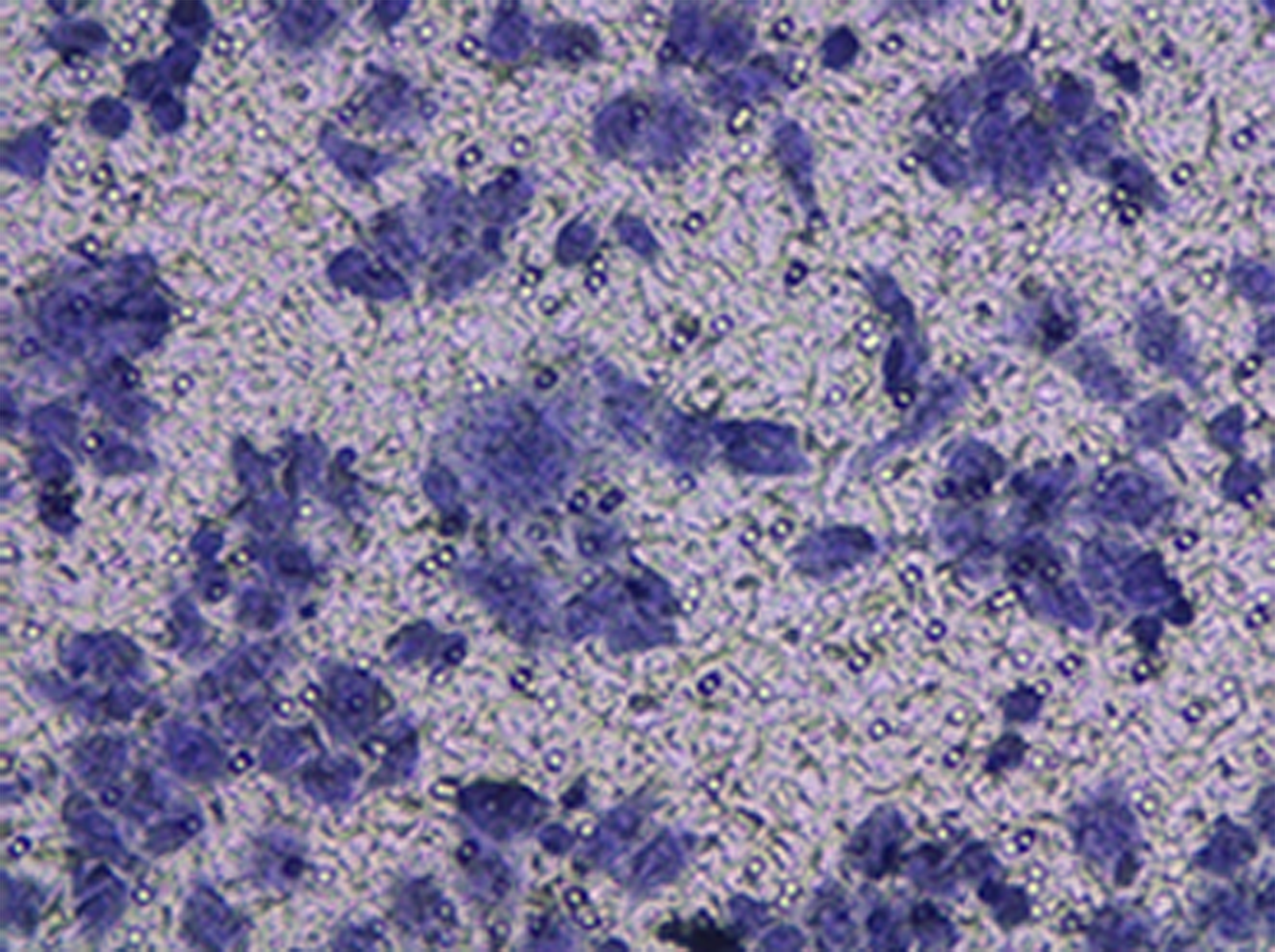

Supplement: S3 Data — (ZIP) [file pgen.1010366.s007.zip › 7B Migration T24 miR-3165 inhibitor sh-METTL14.png]

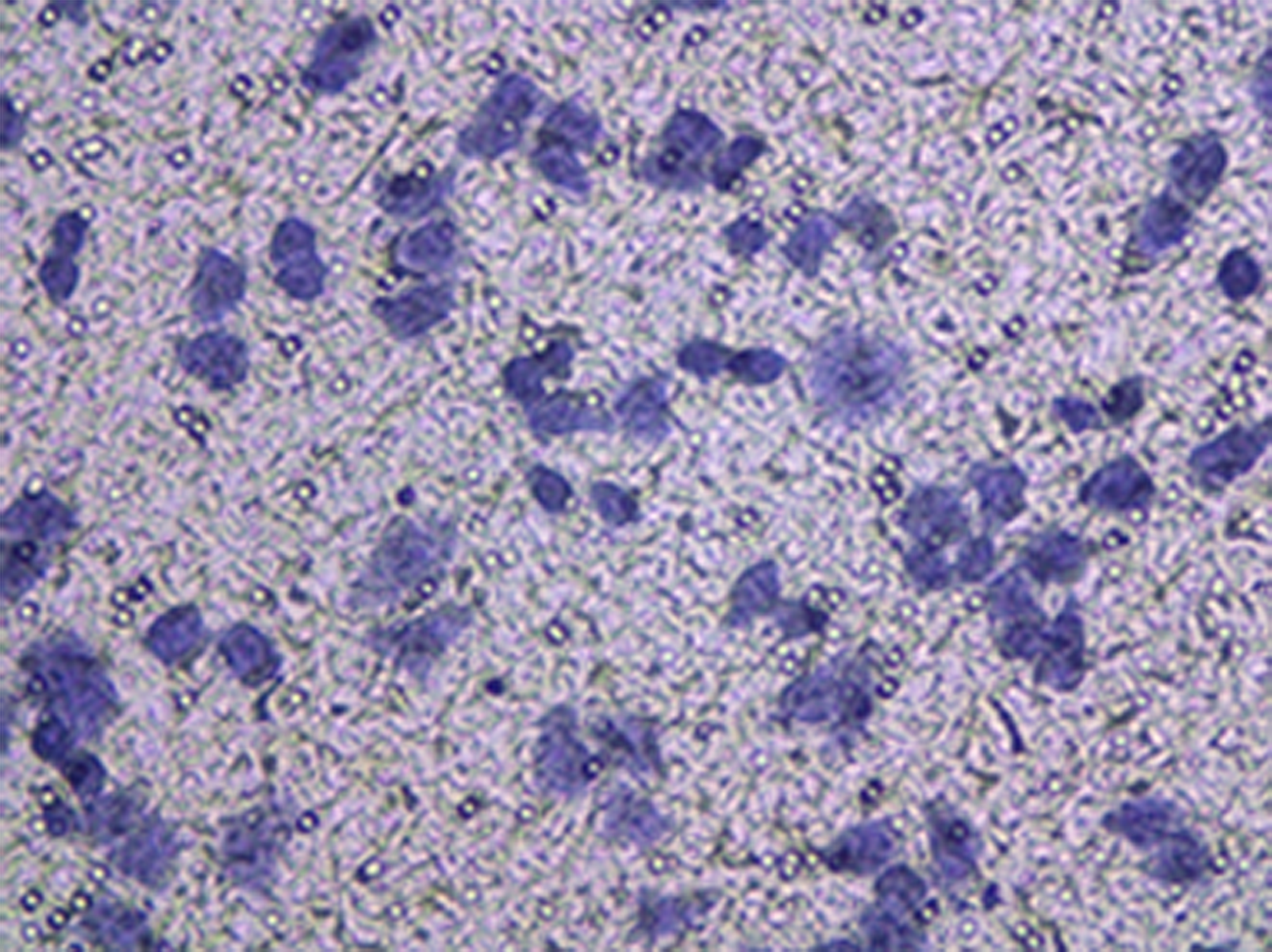

Supplement: S3 Data — (ZIP) [file pgen.1010366.s007.zip › 7B Migration T24 miR-3165 inhibitor.png]

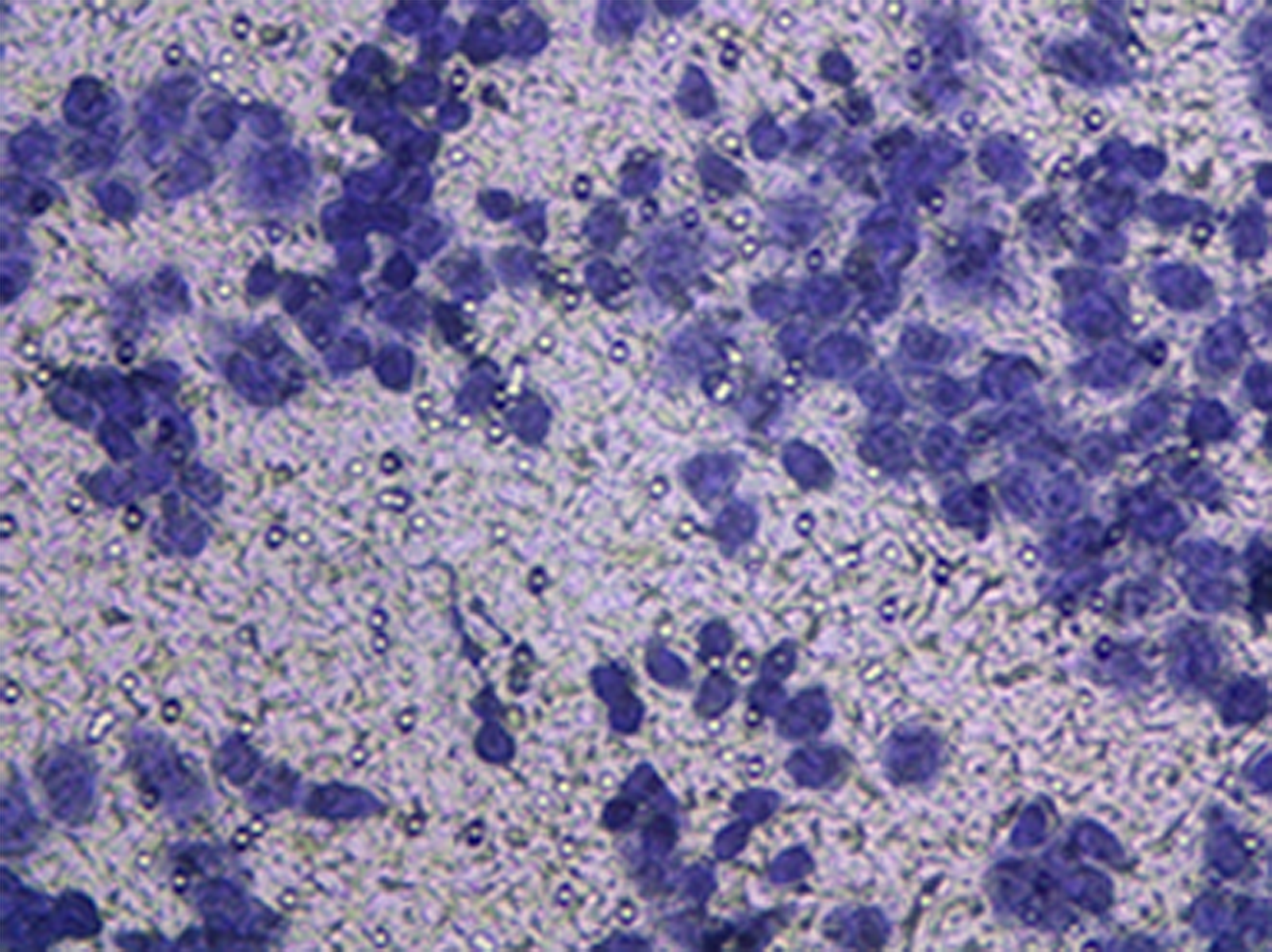

Supplement: S3 Data — (ZIP) [file pgen.1010366.s007.zip › 7B Migration T24 NC inhibitor.png]

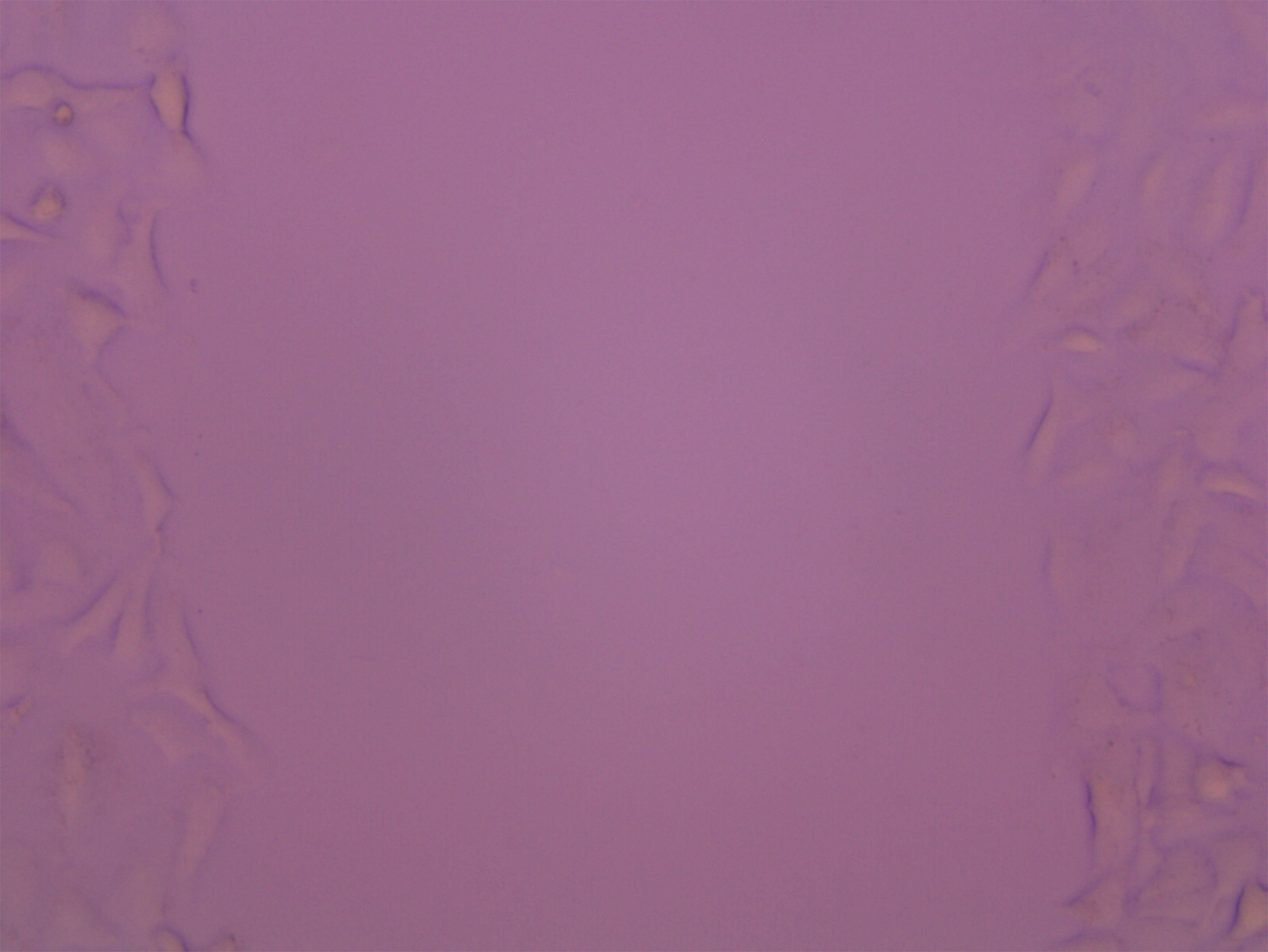

Supplement: S4 Data — (ZIP) [file pgen.1010366.s008.zip › 7C 5637 0h miR-3165 inhibitor sh-METTL14.png]

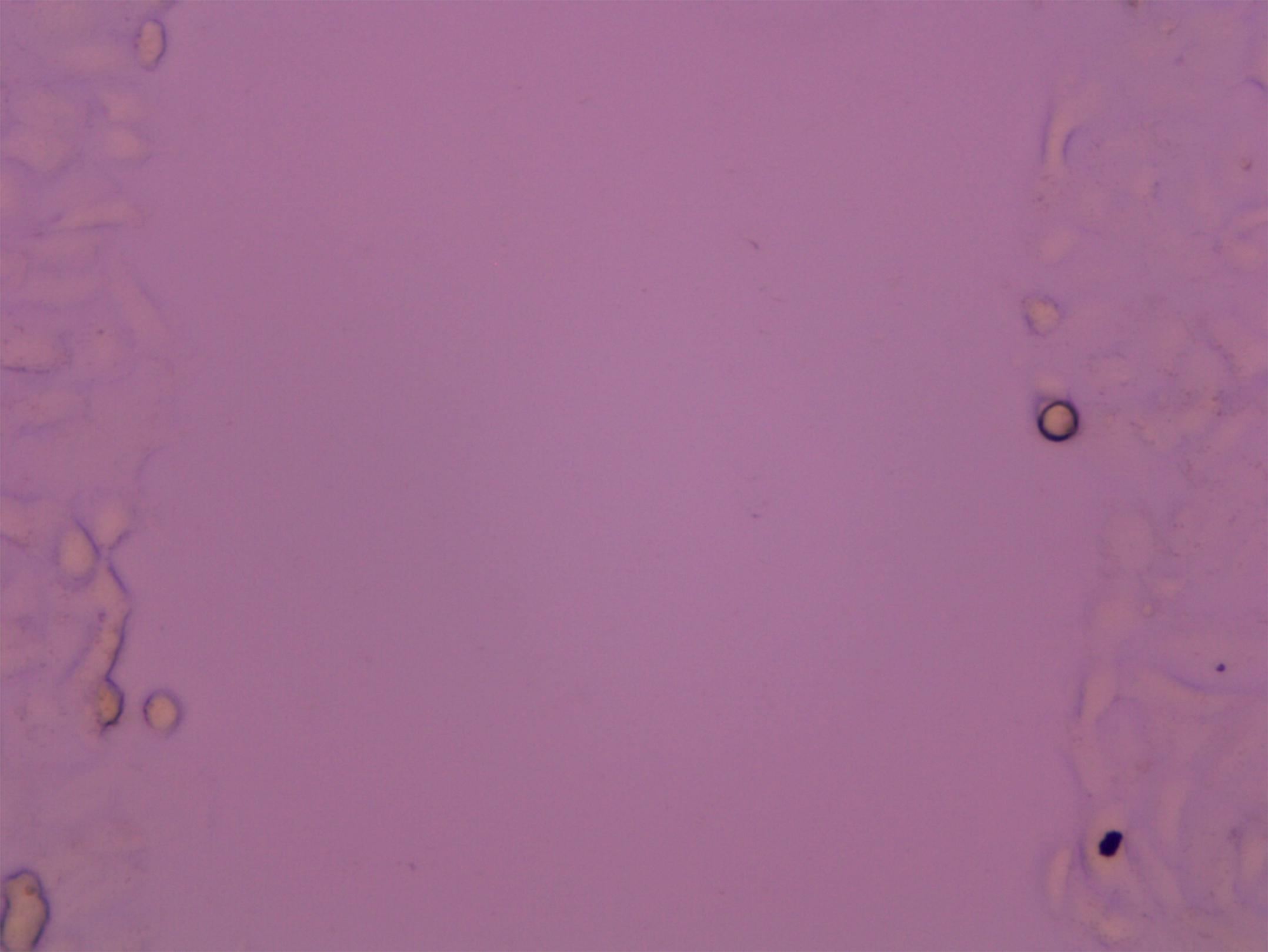

Supplement: S4 Data — (ZIP) [file pgen.1010366.s008.zip › 7C 5637 0h miR-3165 inhibitor.png]

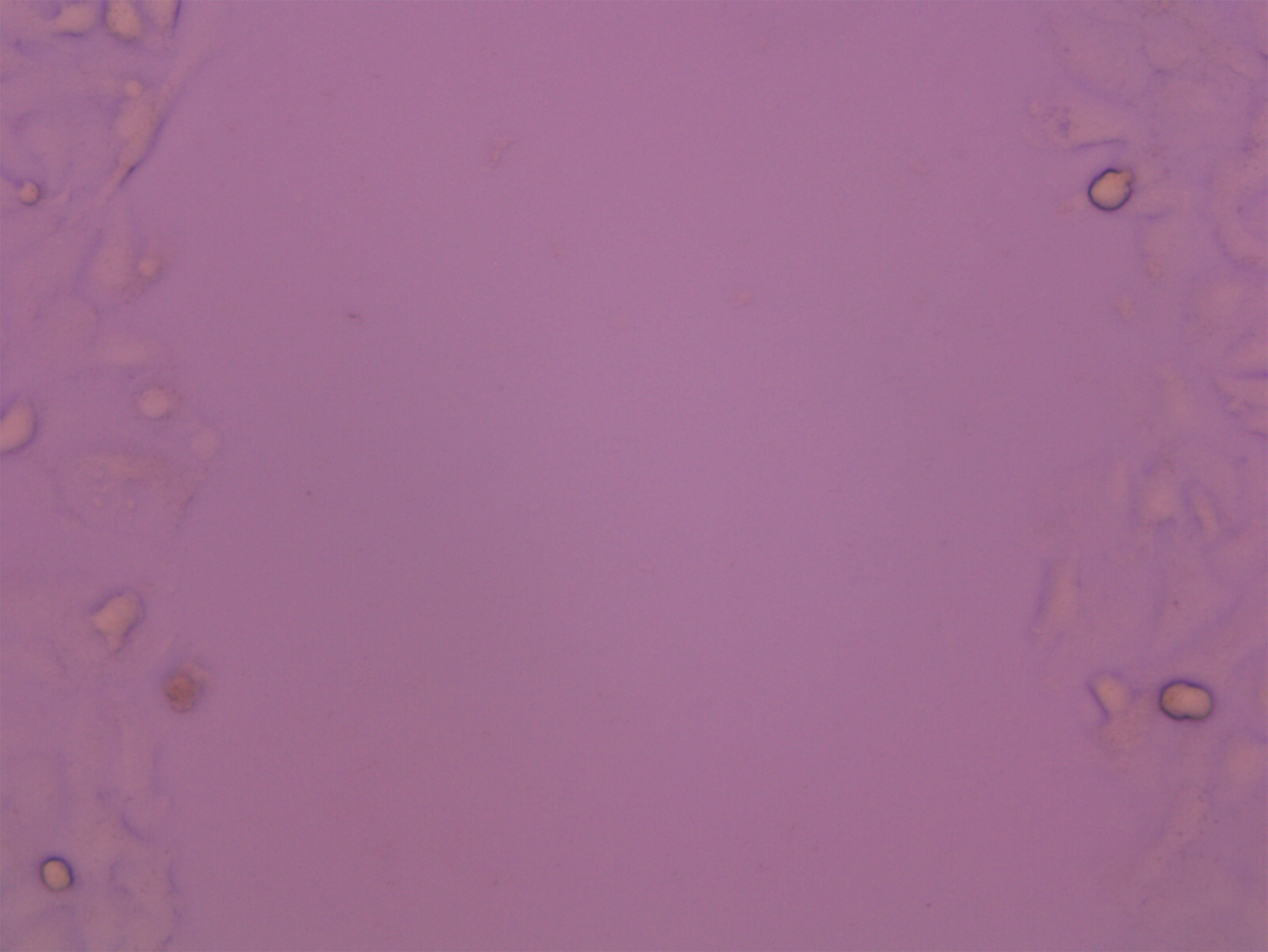

Supplement: S4 Data — (ZIP) [file pgen.1010366.s008.zip › 7C 5637 0h NC inhibitor.png]

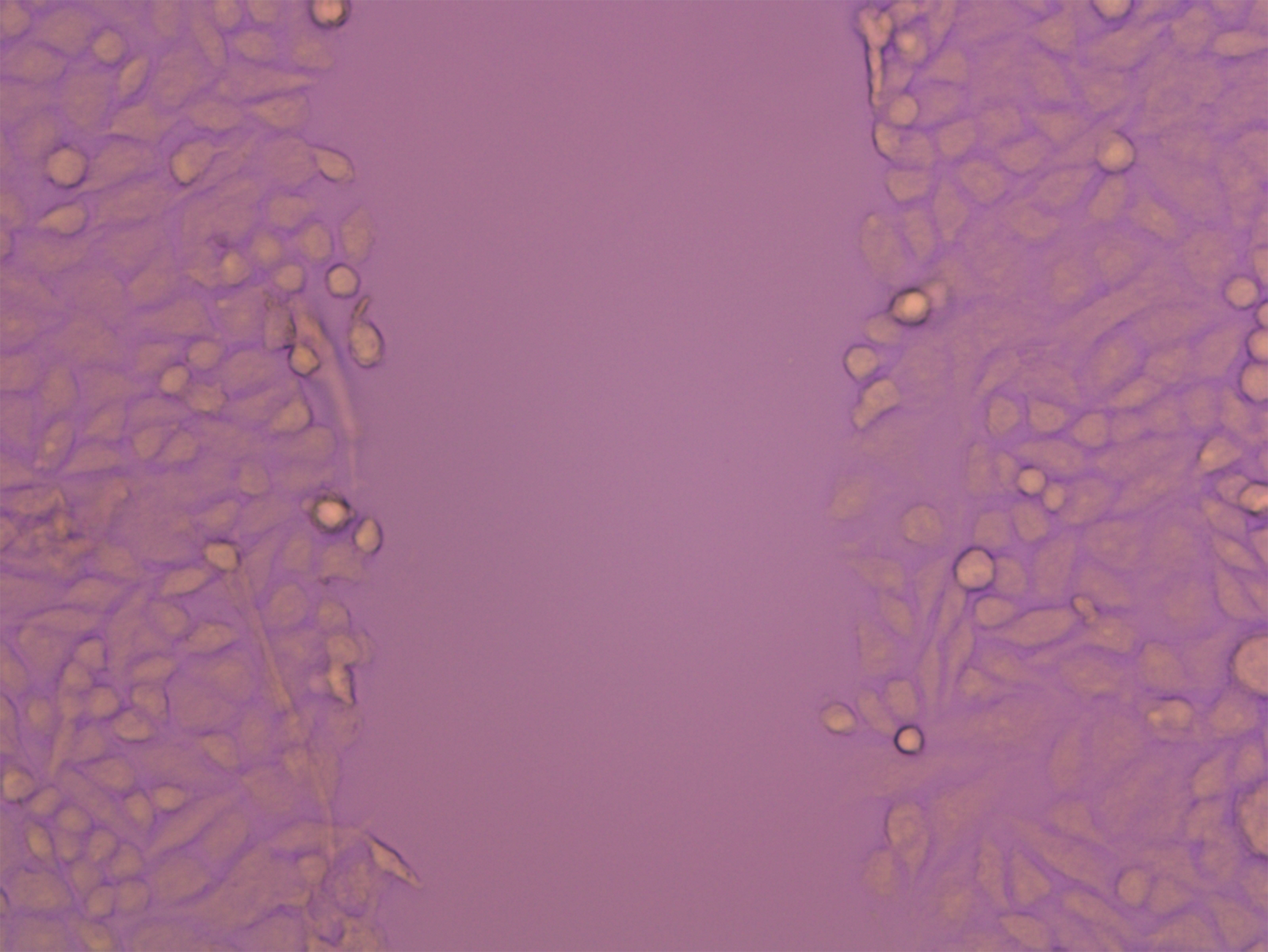

Supplement: S4 Data — (ZIP) [file pgen.1010366.s008.zip › 7C 5637 24h miR-3165 inhibitor sh-METTL14.png]

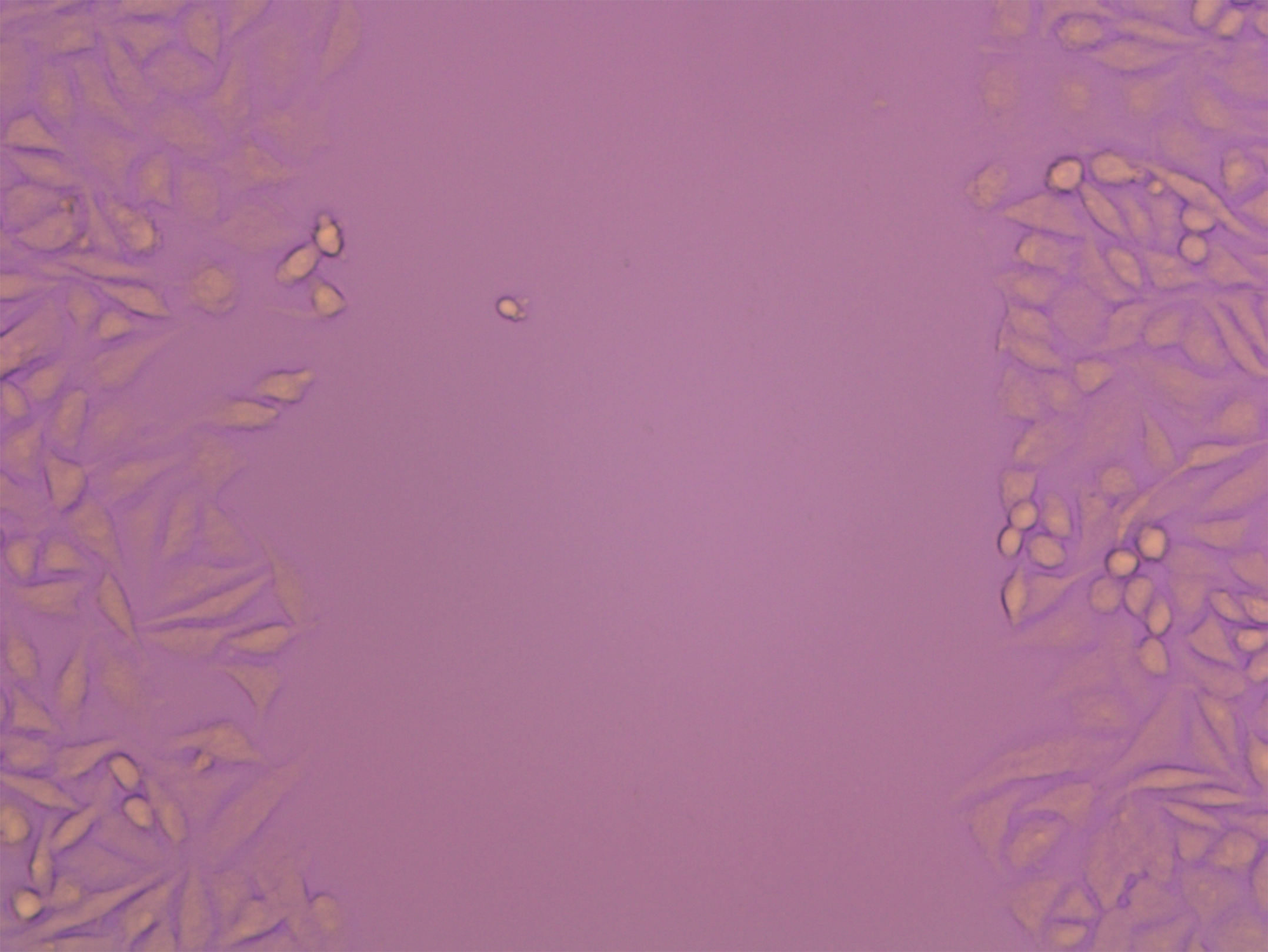

Supplement: S4 Data — (ZIP) [file pgen.1010366.s008.zip › 7C 5637 24h miR-3165 inhibitor.png]

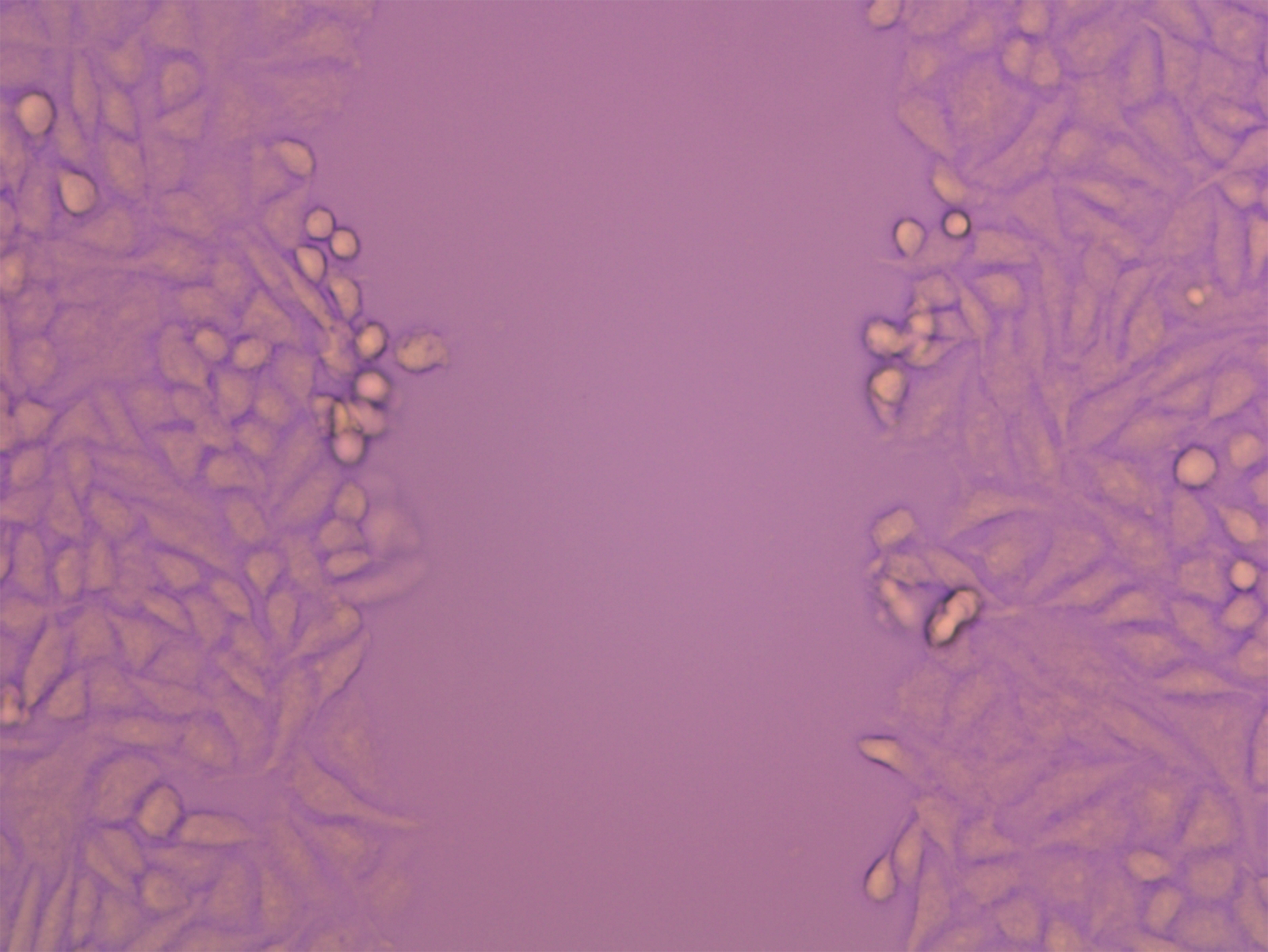

Supplement: S4 Data — (ZIP) [file pgen.1010366.s008.zip › 7C 5637 24h NC inhibitor.png]

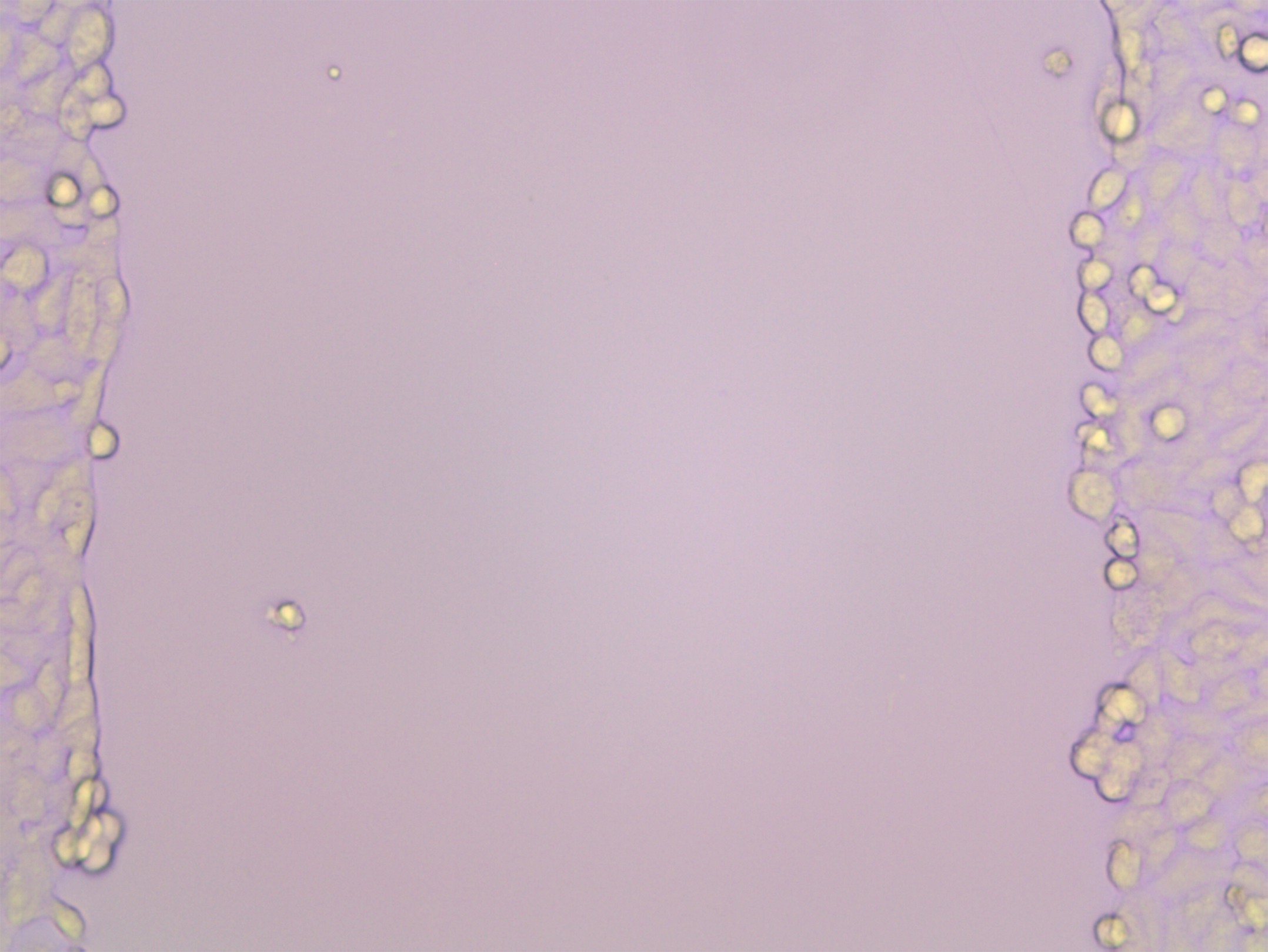

Supplement: S4 Data — (ZIP) [file pgen.1010366.s008.zip › 7C T24 0h miR-3165 inhibitor sh-METTL14.png]

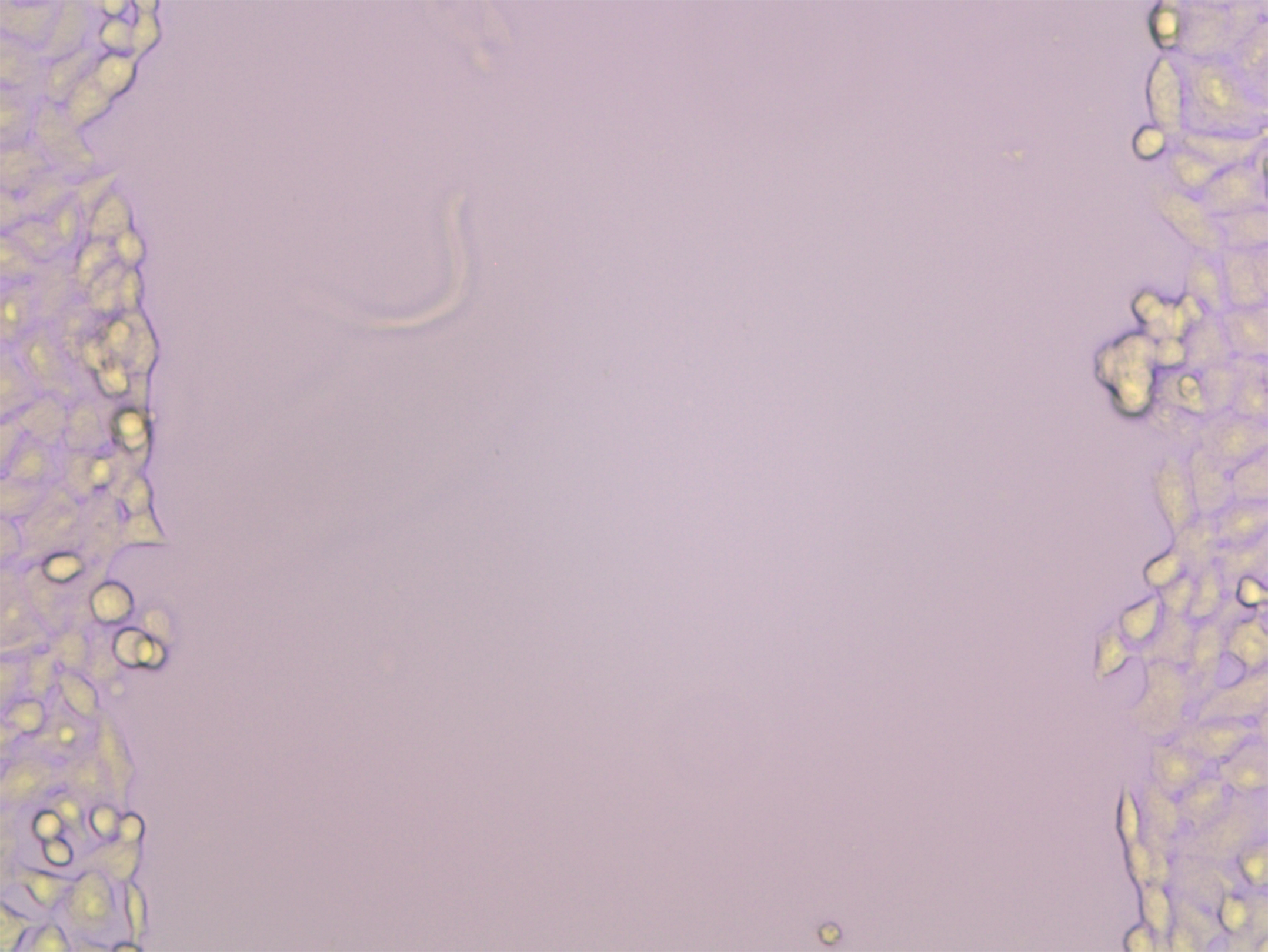

Supplement: S4 Data — (ZIP) [file pgen.1010366.s008.zip › 7C T24 0h miR-3165 inhibitor.png]

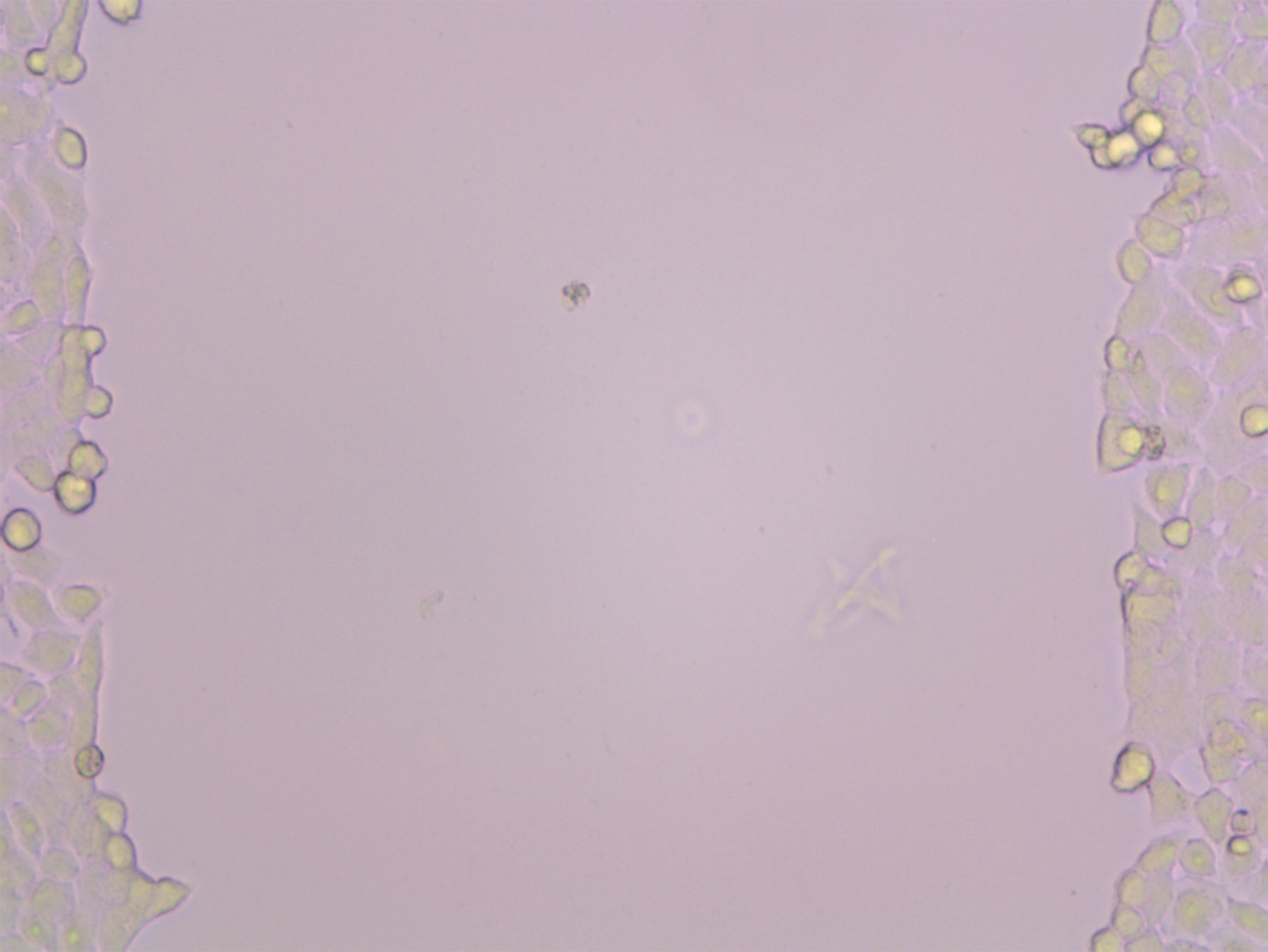

Supplement: S4 Data — (ZIP) [file pgen.1010366.s008.zip › 7C T24 0h NC inhibitor.png]

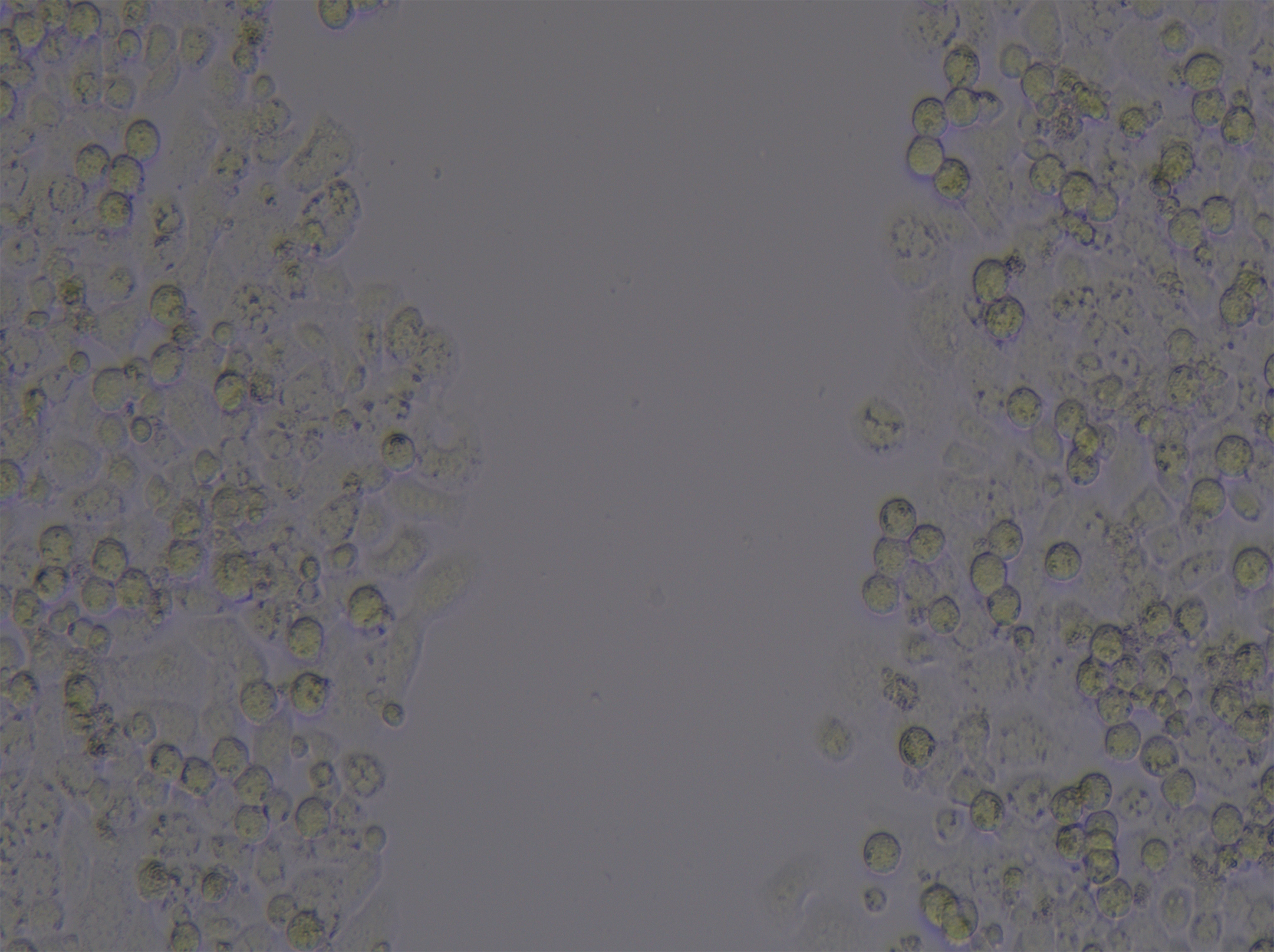

Supplement: S4 Data — (ZIP) [file pgen.1010366.s008.zip › 7C T24 24h miR-3165 inhibitor sh-METTL14.png]

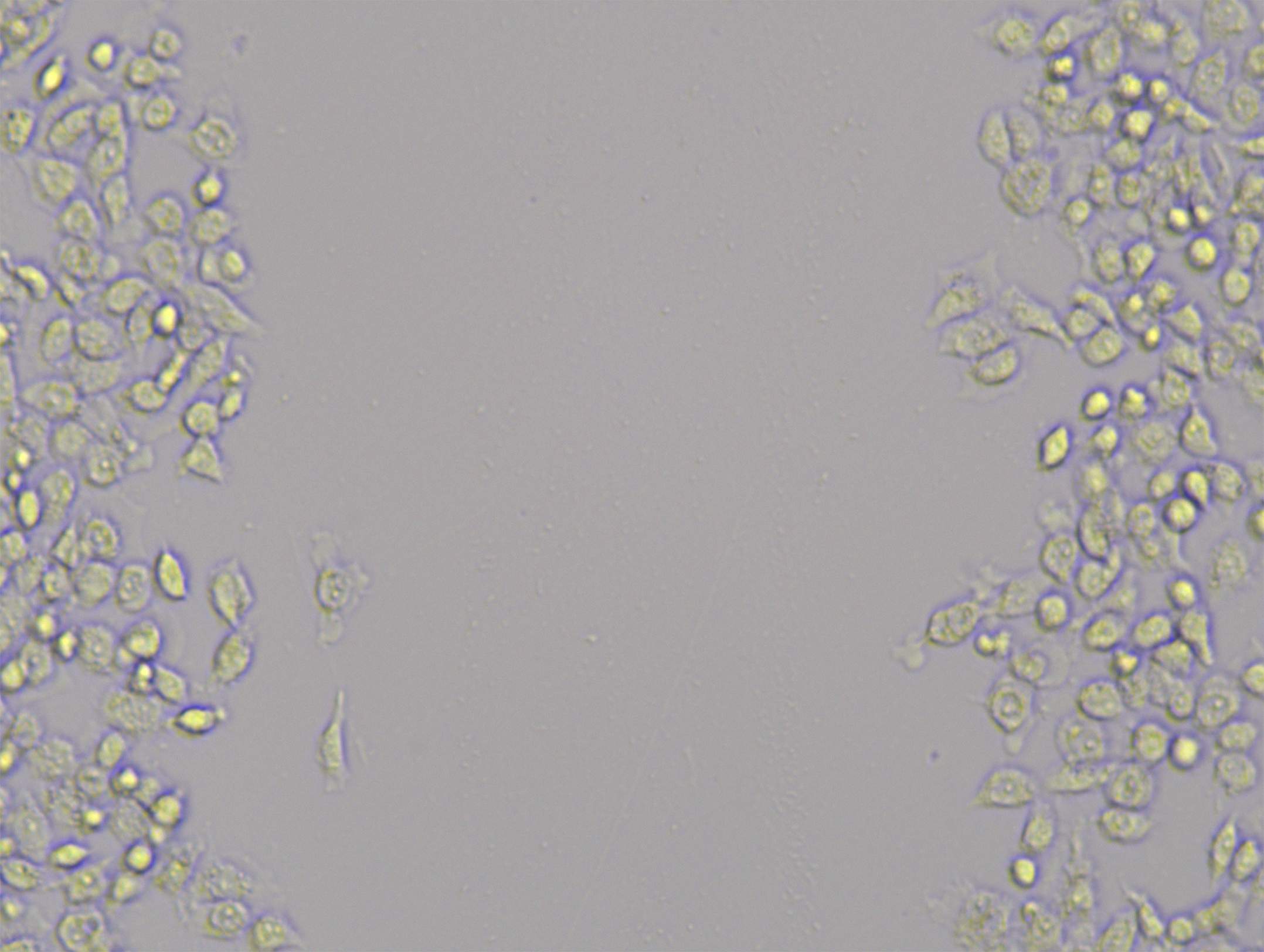

Supplement: S4 Data — (ZIP) [file pgen.1010366.s008.zip › 7C T24 24h miR-3165 inhibitor.png]

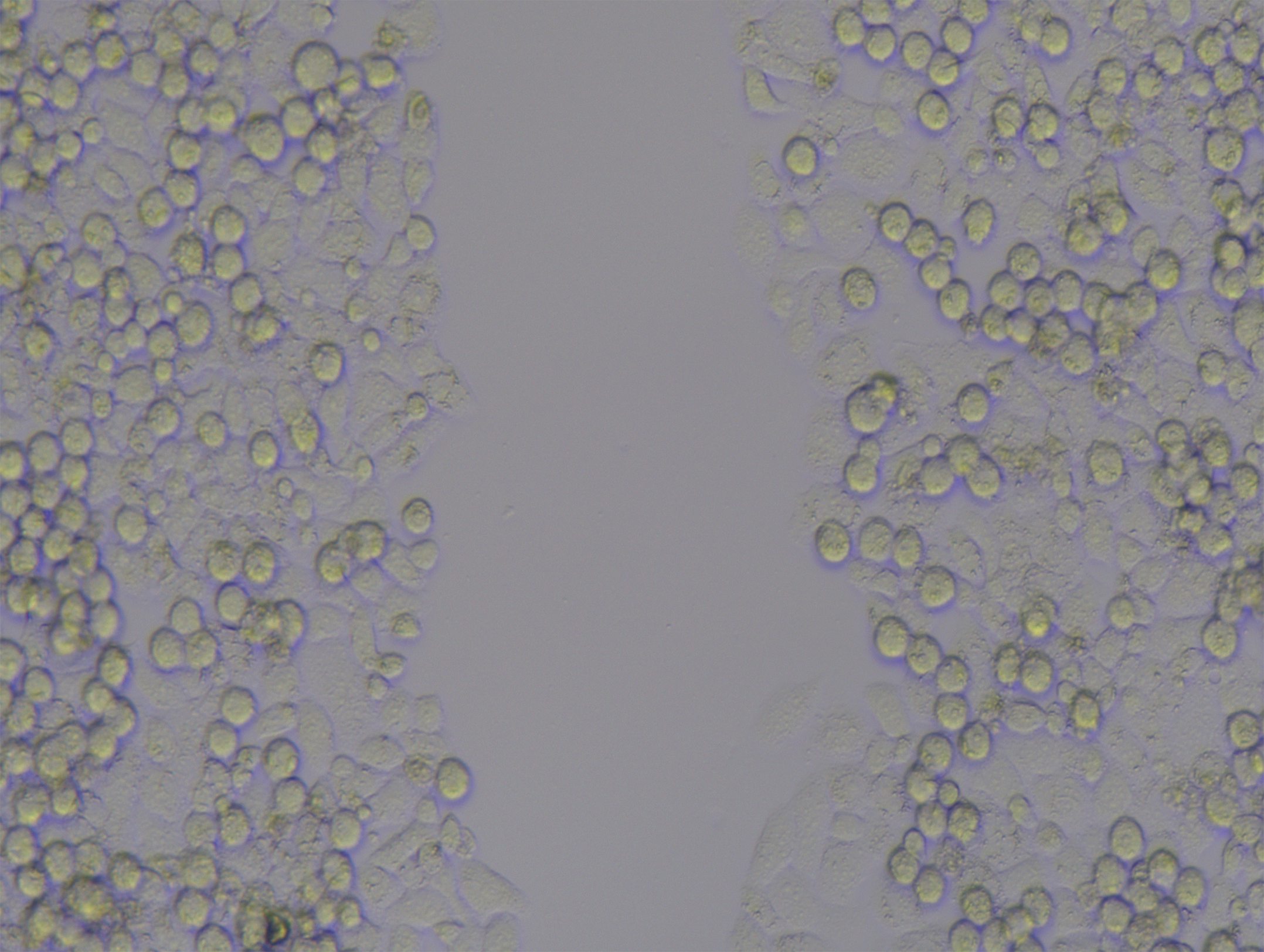

Supplement: S4 Data — (ZIP) [file pgen.1010366.s008.zip › 7C T24 24h NC inhibitor.png]

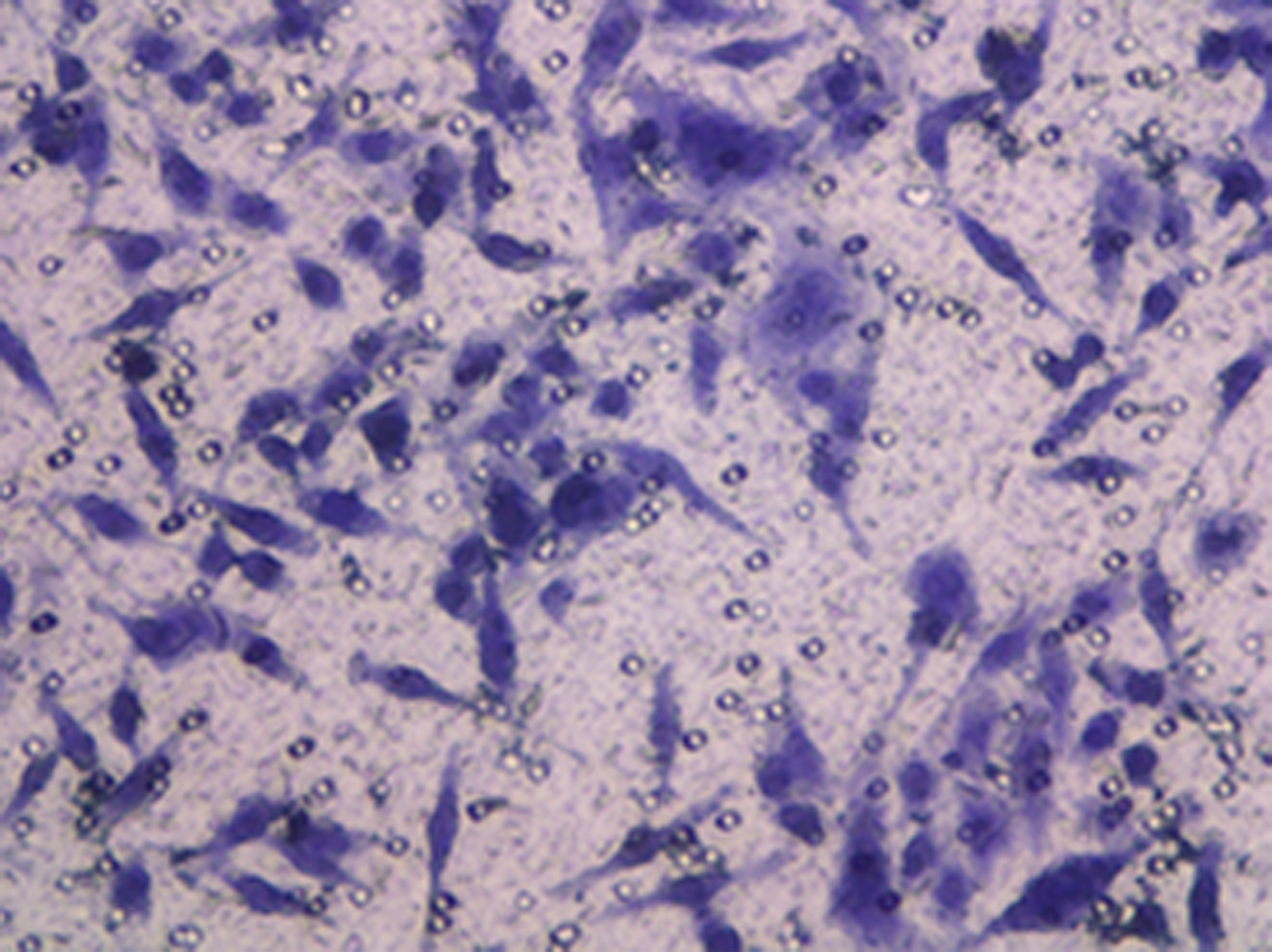

Supplement: S5 Data — (ZIP) [file pgen.1010366.s009.zip › 7D Invasion 5637 miR-3165 inhibitor sh-METTL14.png]

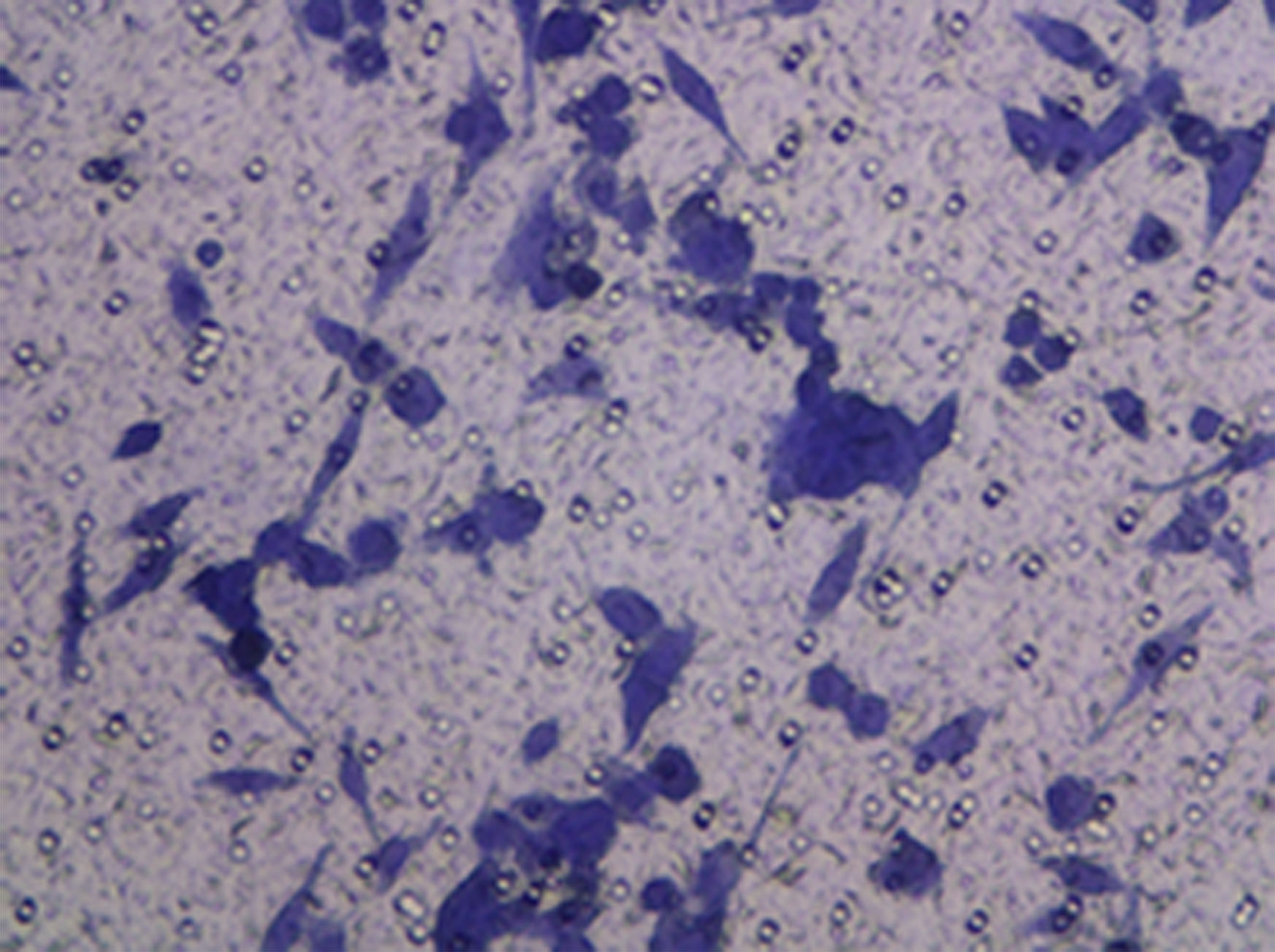

Supplement: S5 Data — (ZIP) [file pgen.1010366.s009.zip › 7D Invasion 5637 miR-3165 inhibitor.png]

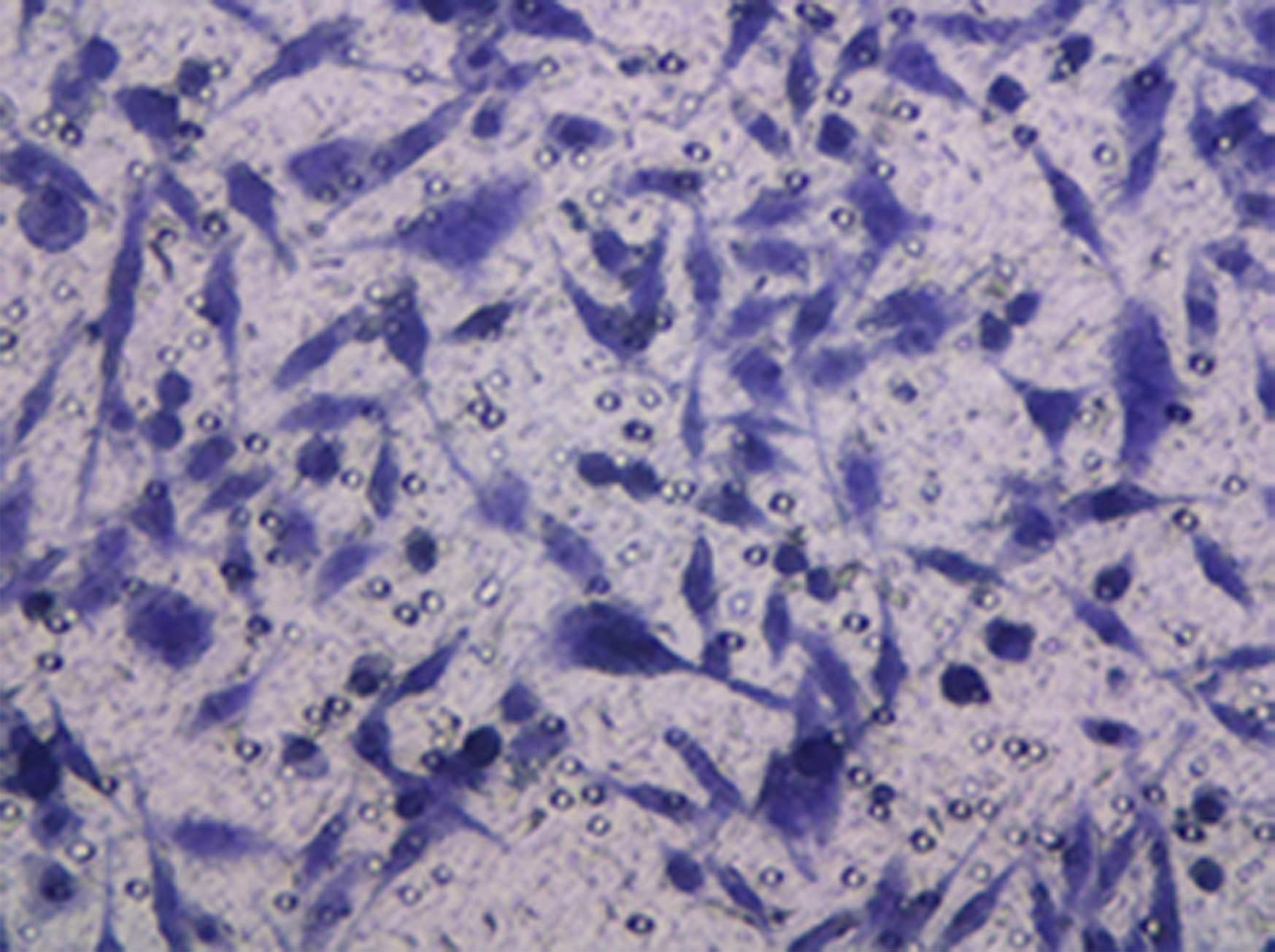

Supplement: S5 Data — (ZIP) [file pgen.1010366.s009.zip › 7D Invasion 5637 NC inhibitor.png]

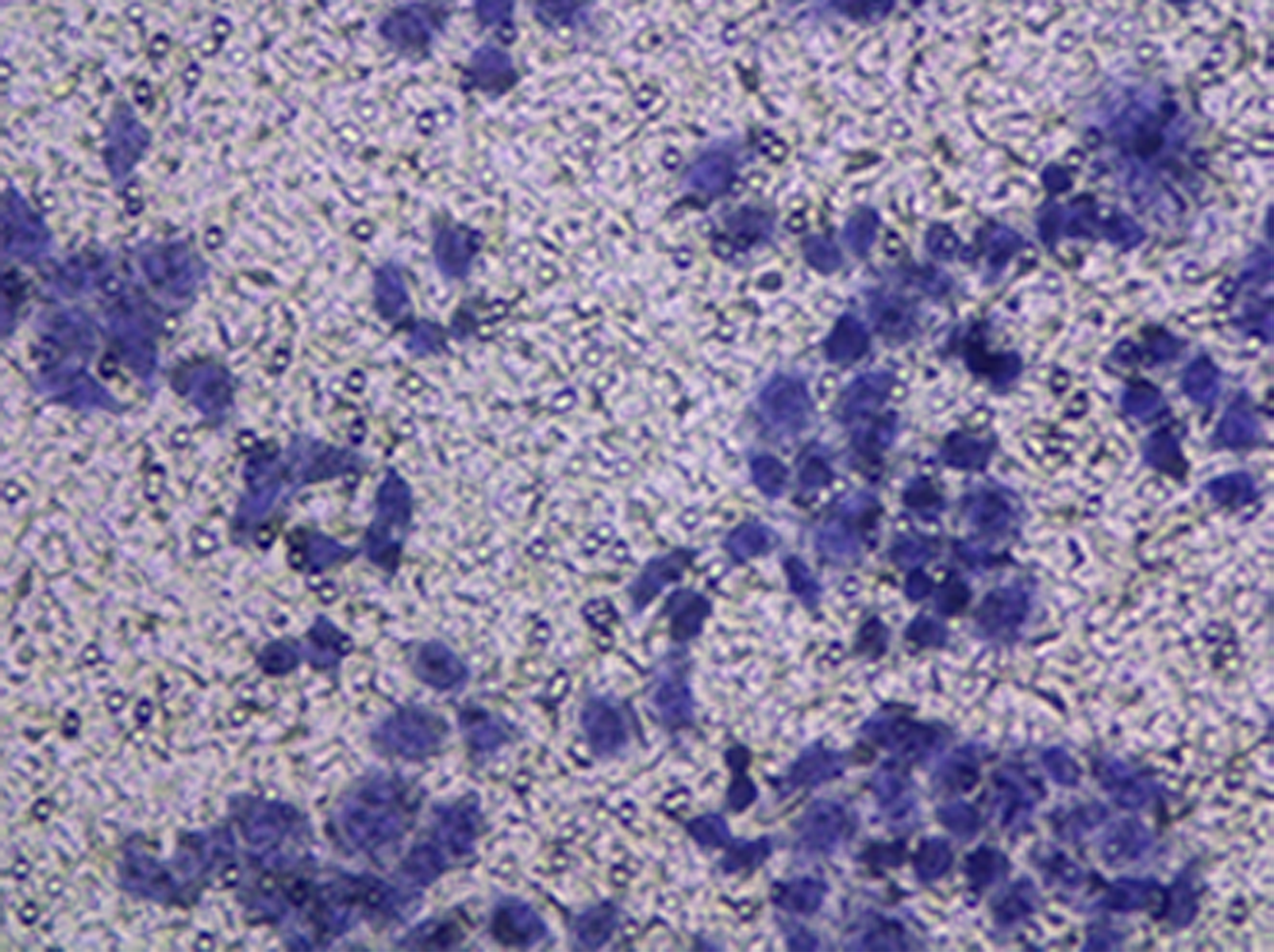

Supplement: S5 Data — (ZIP) [file pgen.1010366.s009.zip › 7D Invasion T24 miR-3165 inhibitor sh-METTL14.png]

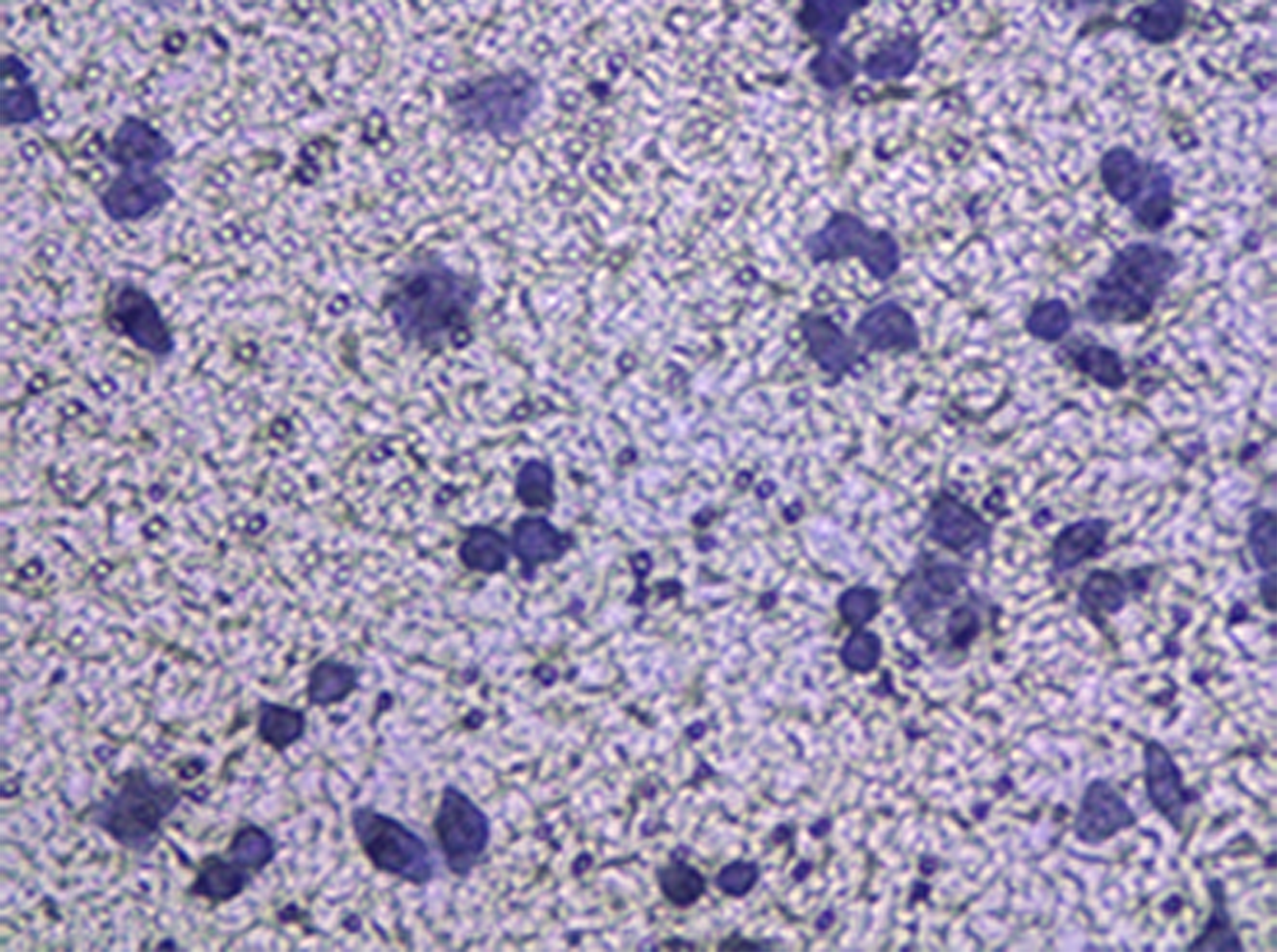

Supplement: S5 Data — (ZIP) [file pgen.1010366.s009.zip › 7D Invasion T24 miR-3165 inhibitor.png]

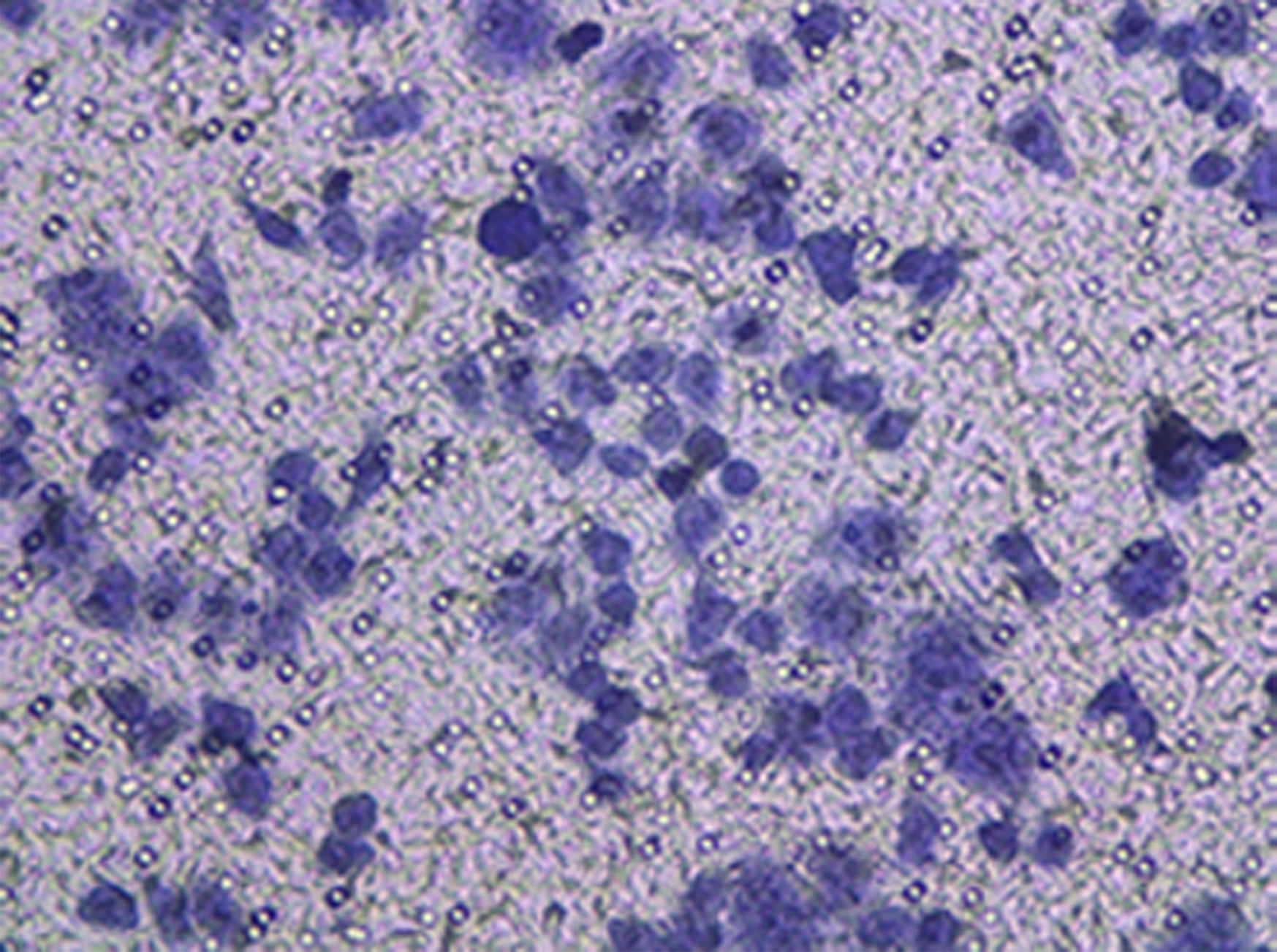

Supplement: S5 Data — (ZIP) [file pgen.1010366.s009.zip › 7D Invasion T24 NC inhibitor.png]

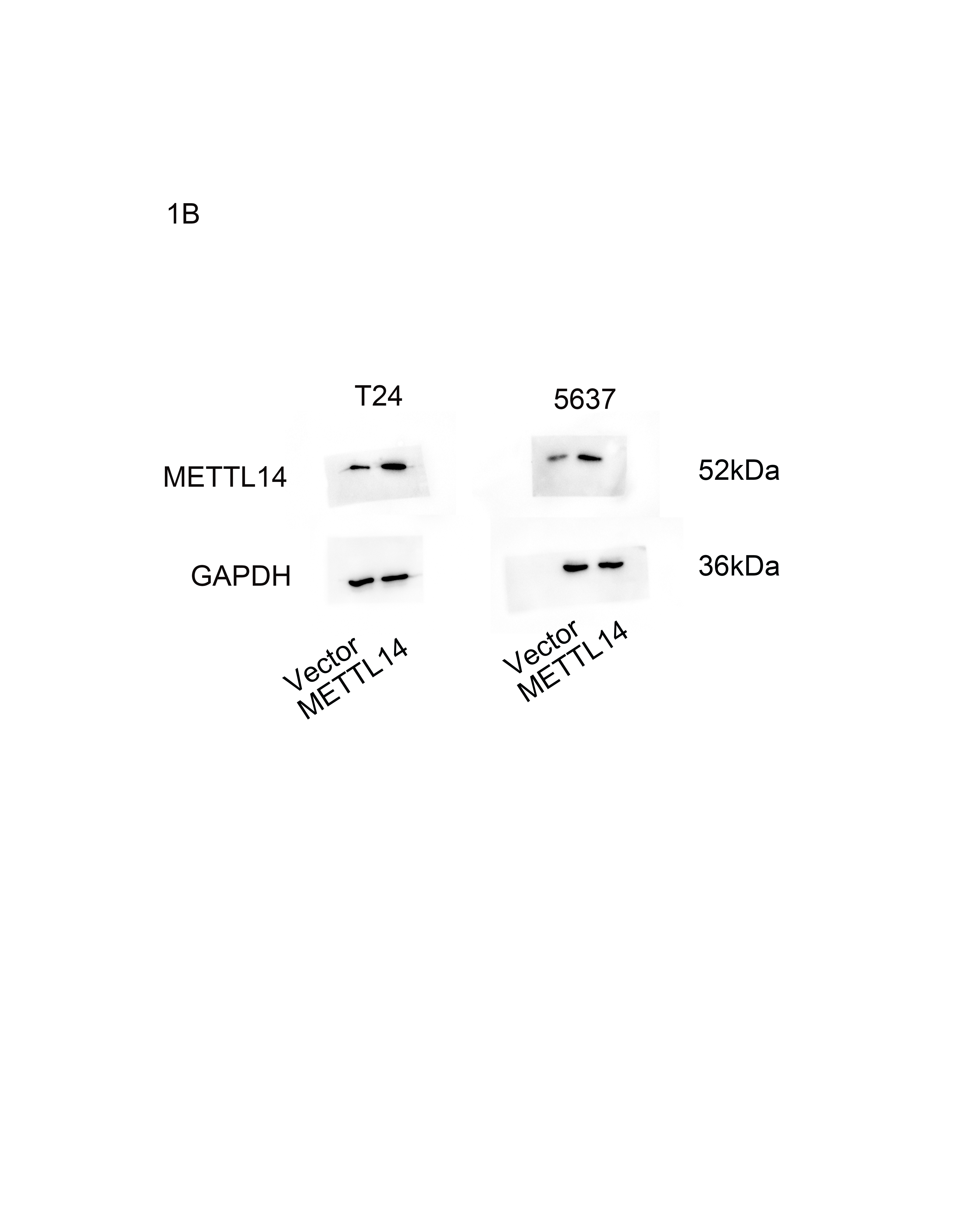

Supplement: S7 Data — (ZIP) [file pgen.1010366.s011.zip › Figure 1B.tif]

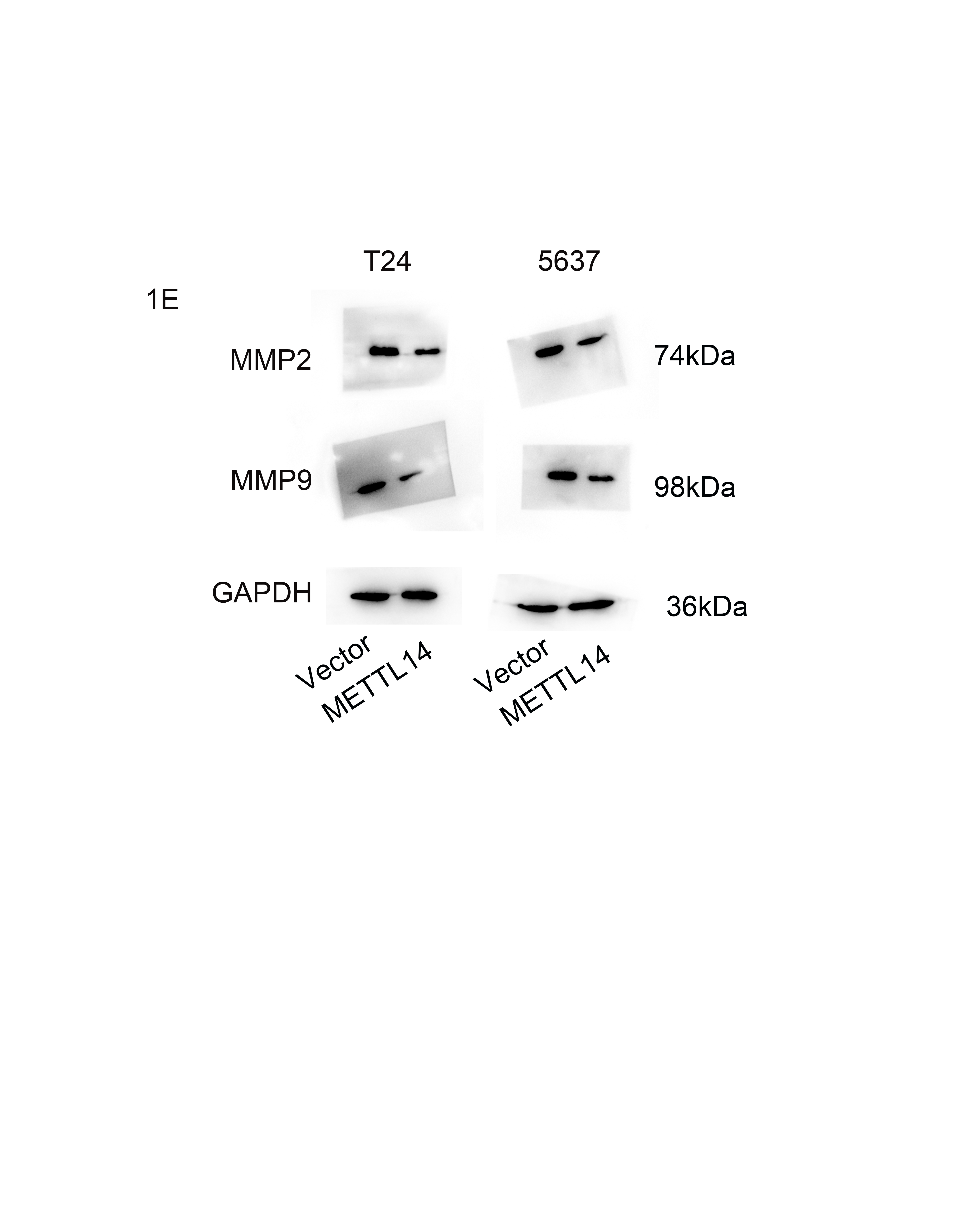

Supplement: S7 Data — (ZIP) [file pgen.1010366.s011.zip › Figure 1E.tif]

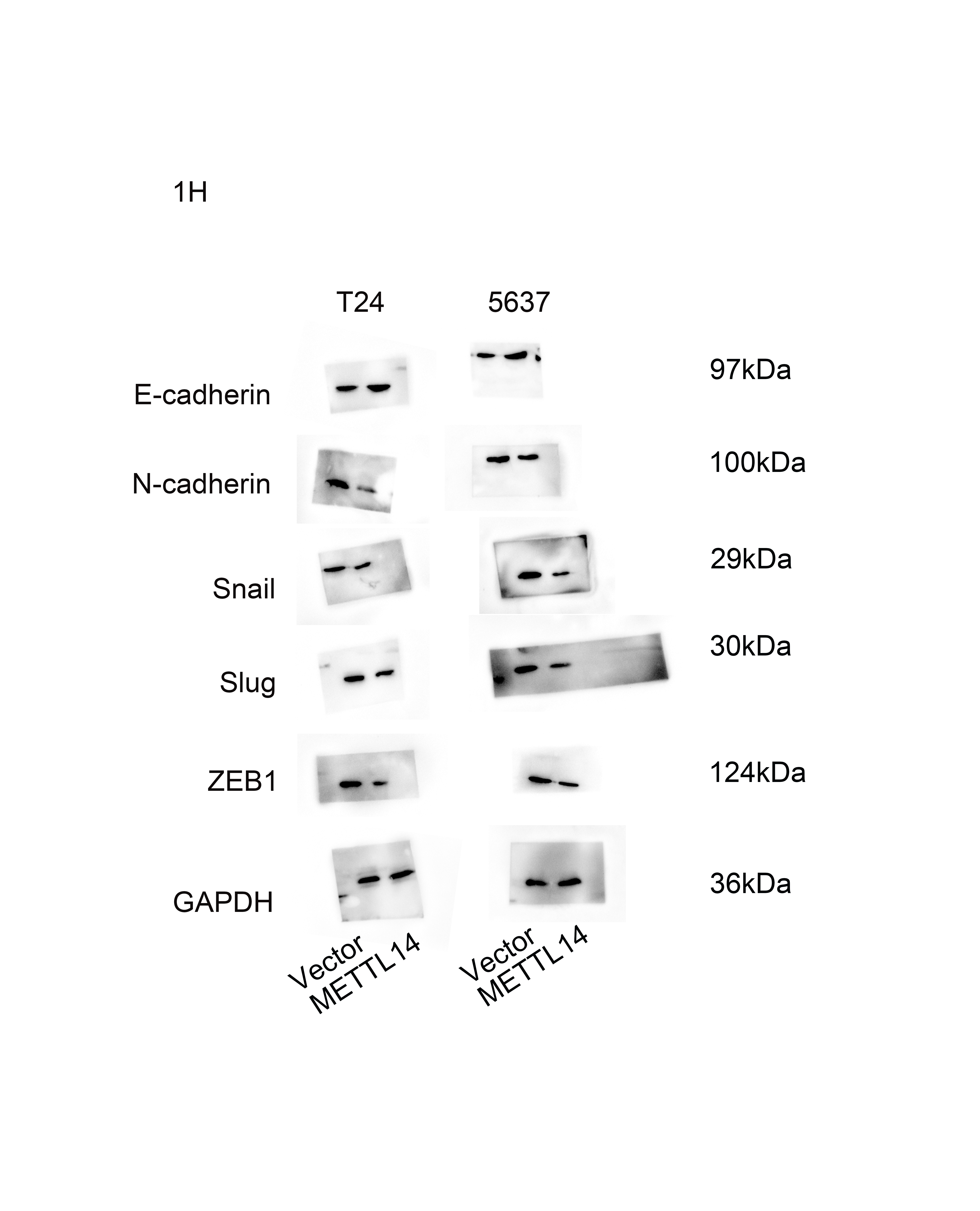

Supplement: S7 Data — (ZIP) [file pgen.1010366.s011.zip › Figure 1H.tif]

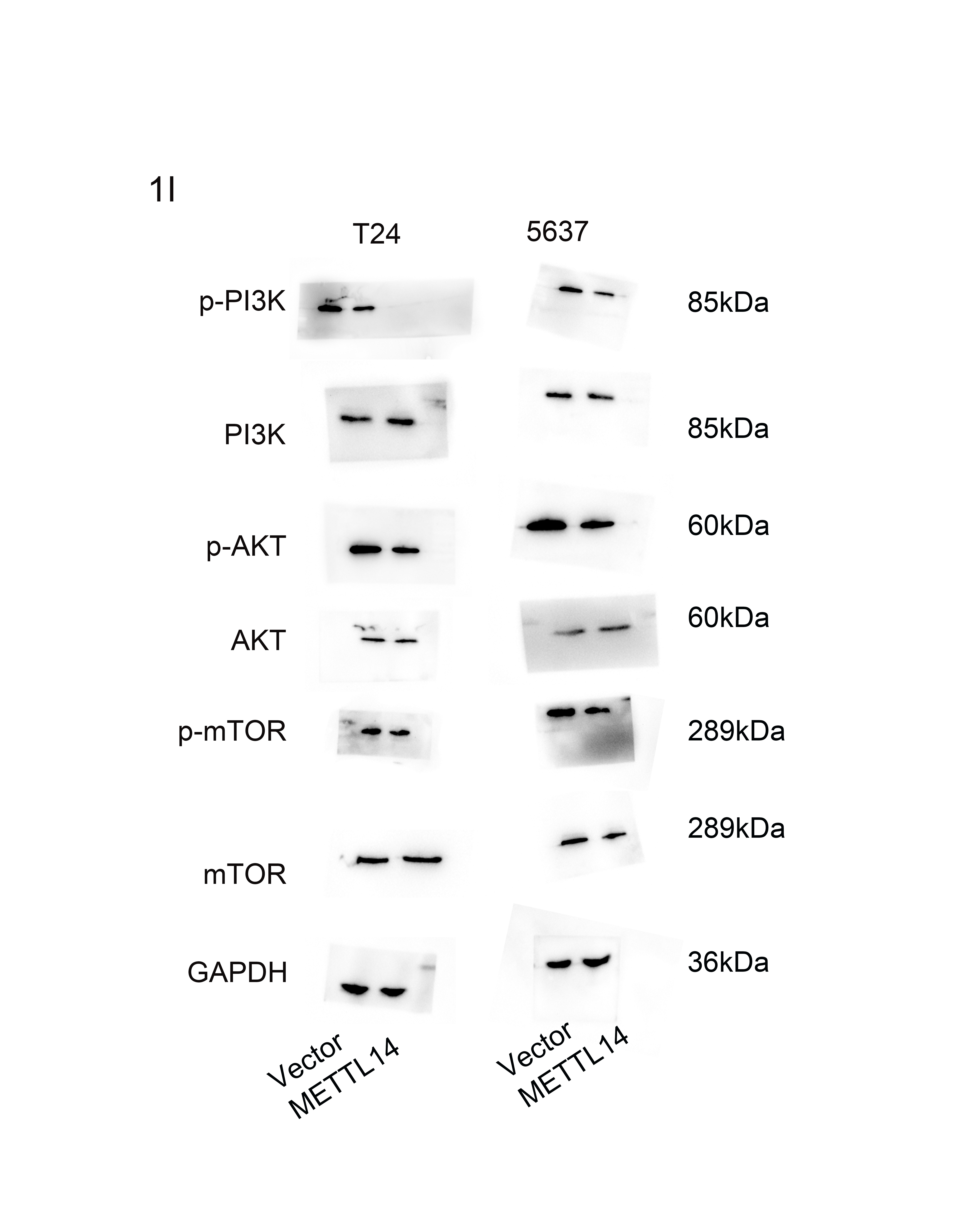

Supplement: S7 Data — (ZIP) [file pgen.1010366.s011.zip › Figure 1I.tif]

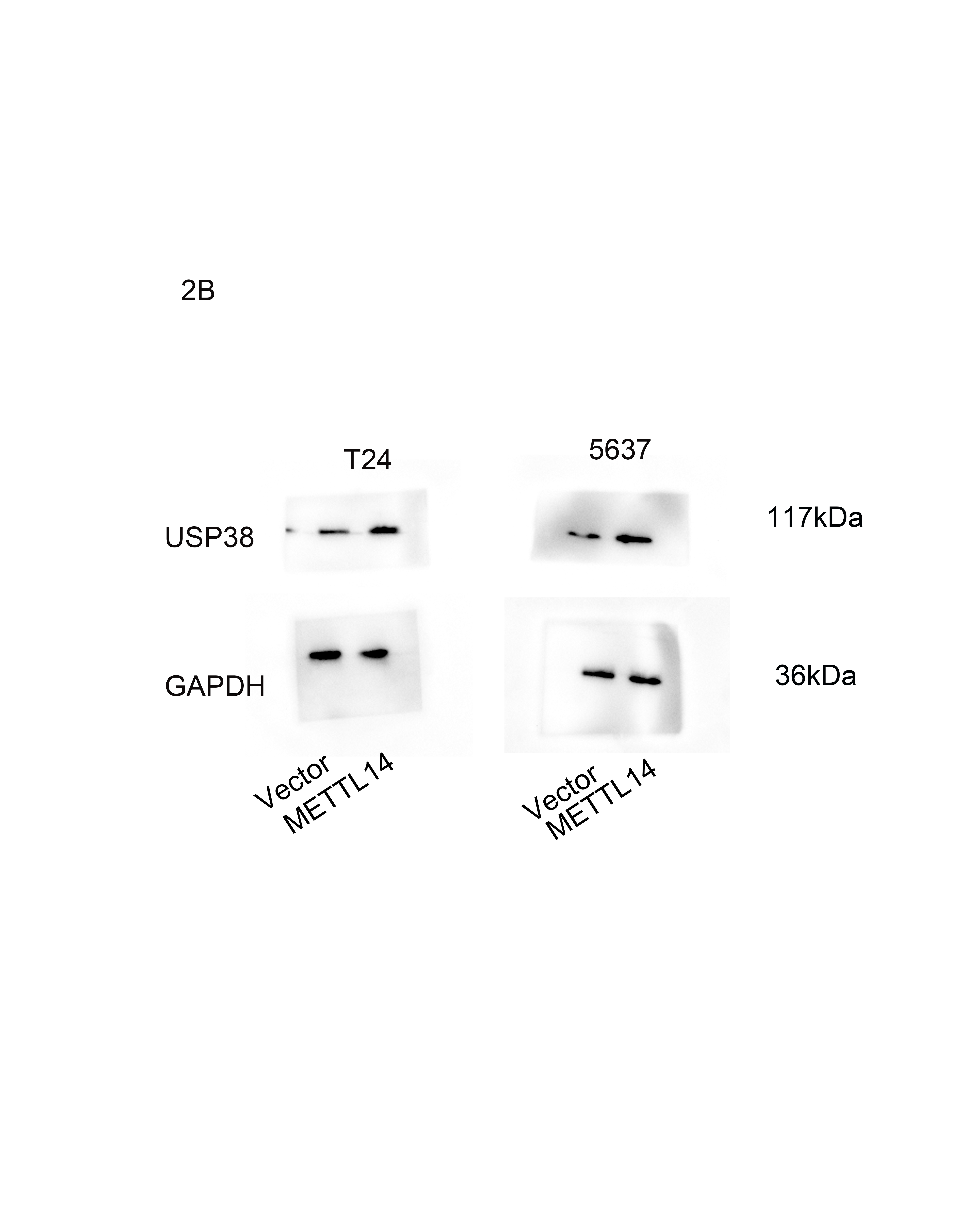

Supplement: S7 Data — (ZIP) [file pgen.1010366.s011.zip › Figure 2B.tif]

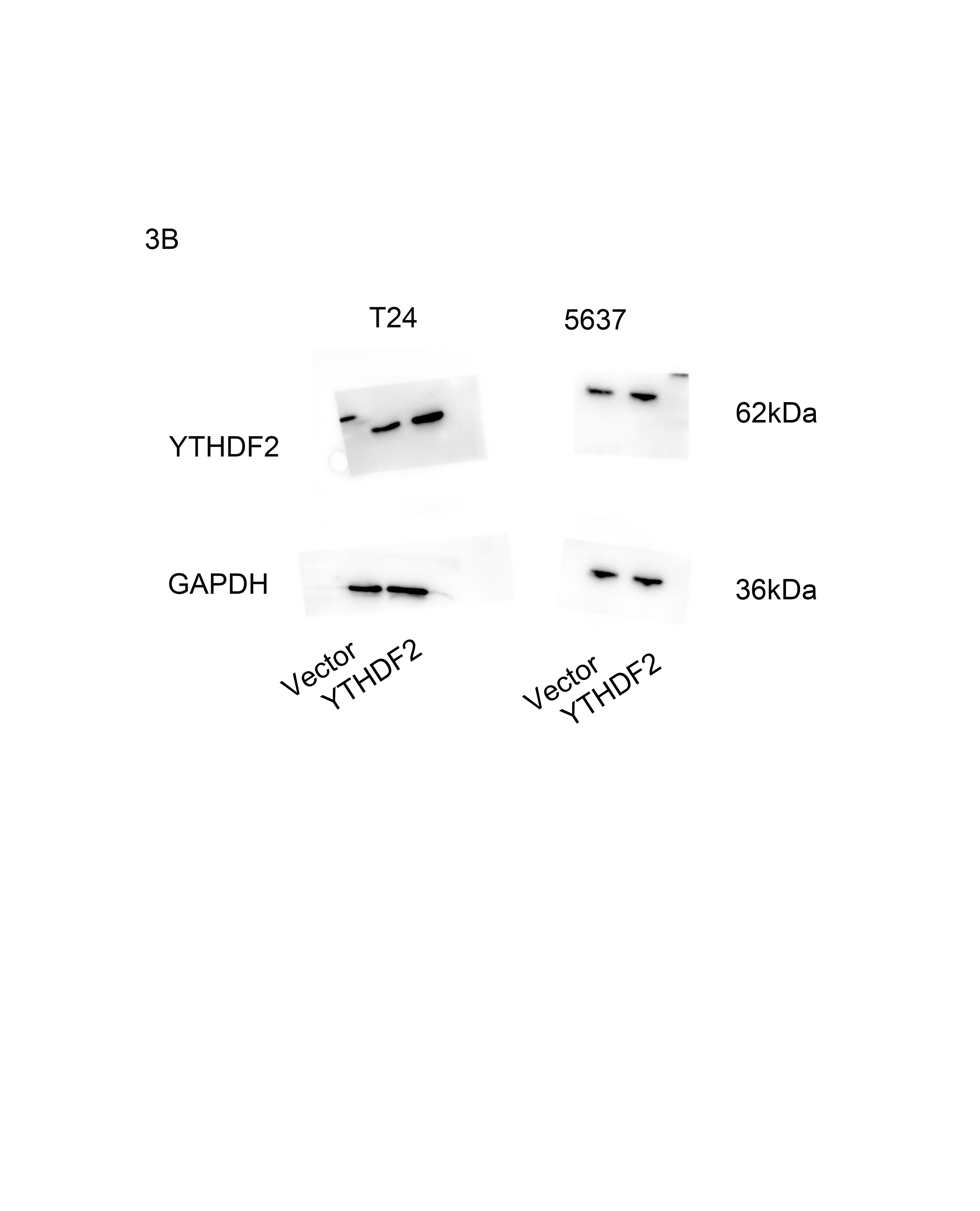

Supplement: S7 Data — (ZIP) [file pgen.1010366.s011.zip › Figure 3B.tif]

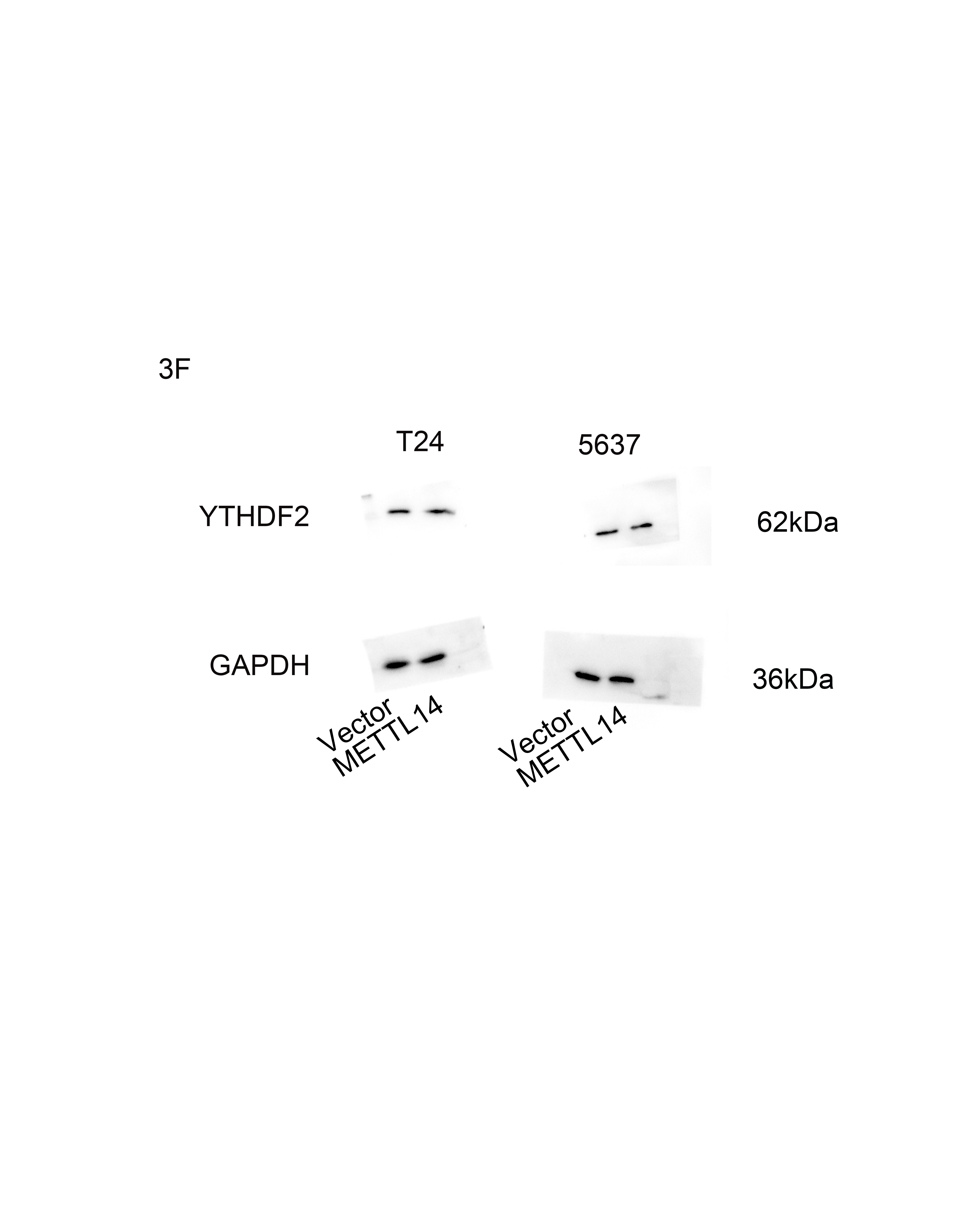

Supplement: S7 Data — (ZIP) [file pgen.1010366.s011.zip › Figure 3F.tif]

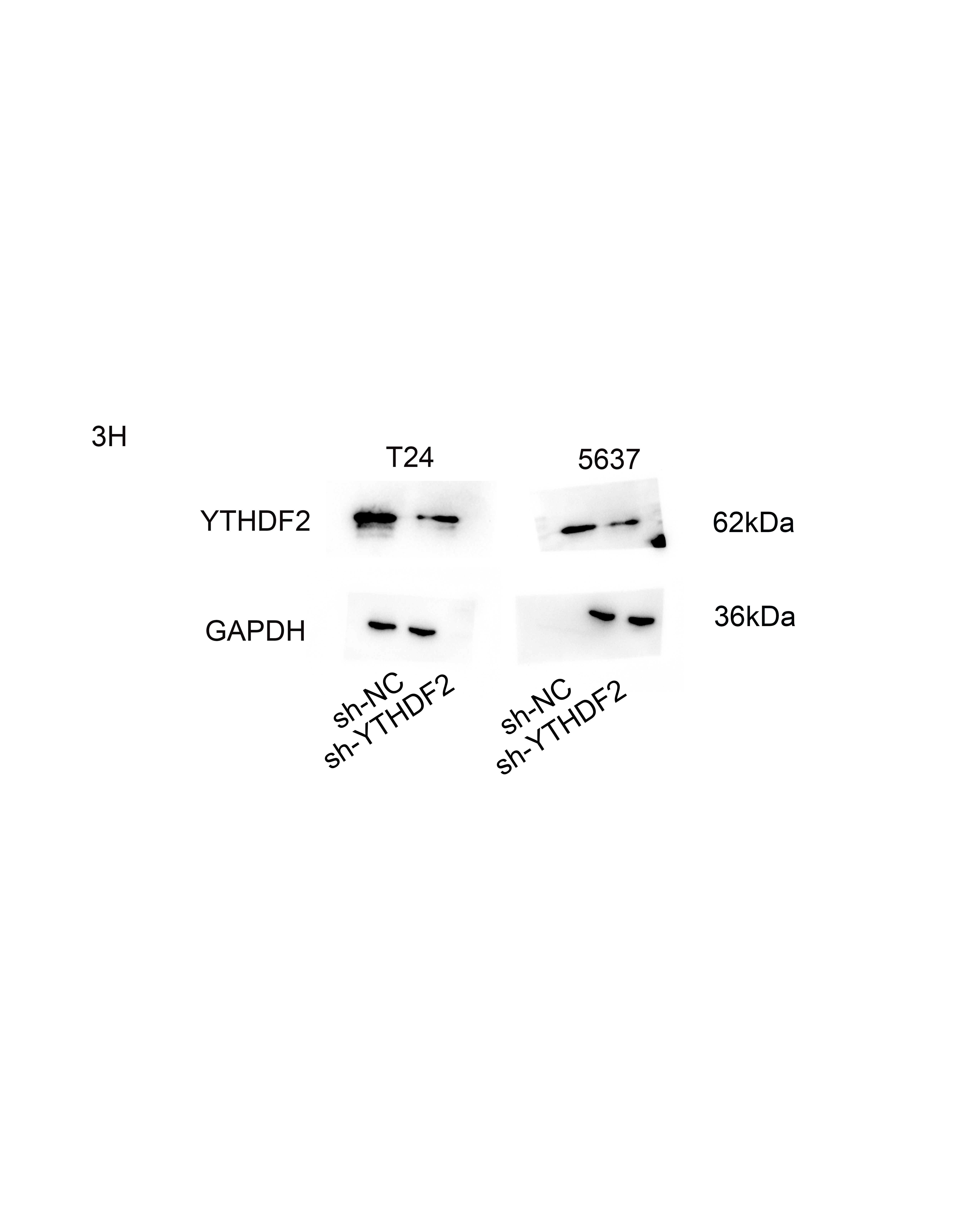

Supplement: S7 Data — (ZIP) [file pgen.1010366.s011.zip › Figure 3H.tif]

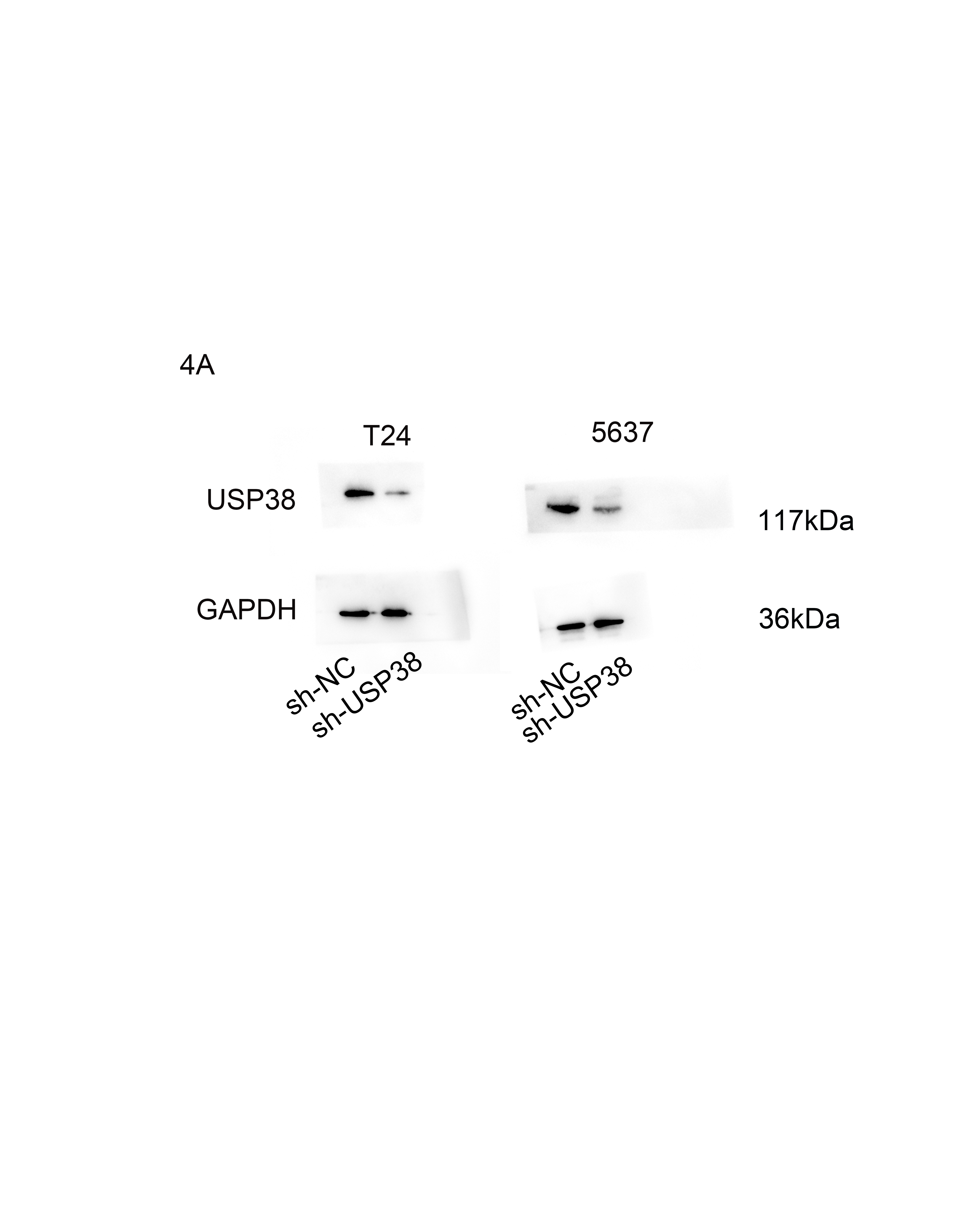

Supplement: S7 Data — (ZIP) [file pgen.1010366.s011.zip › Figure 4A.tif]

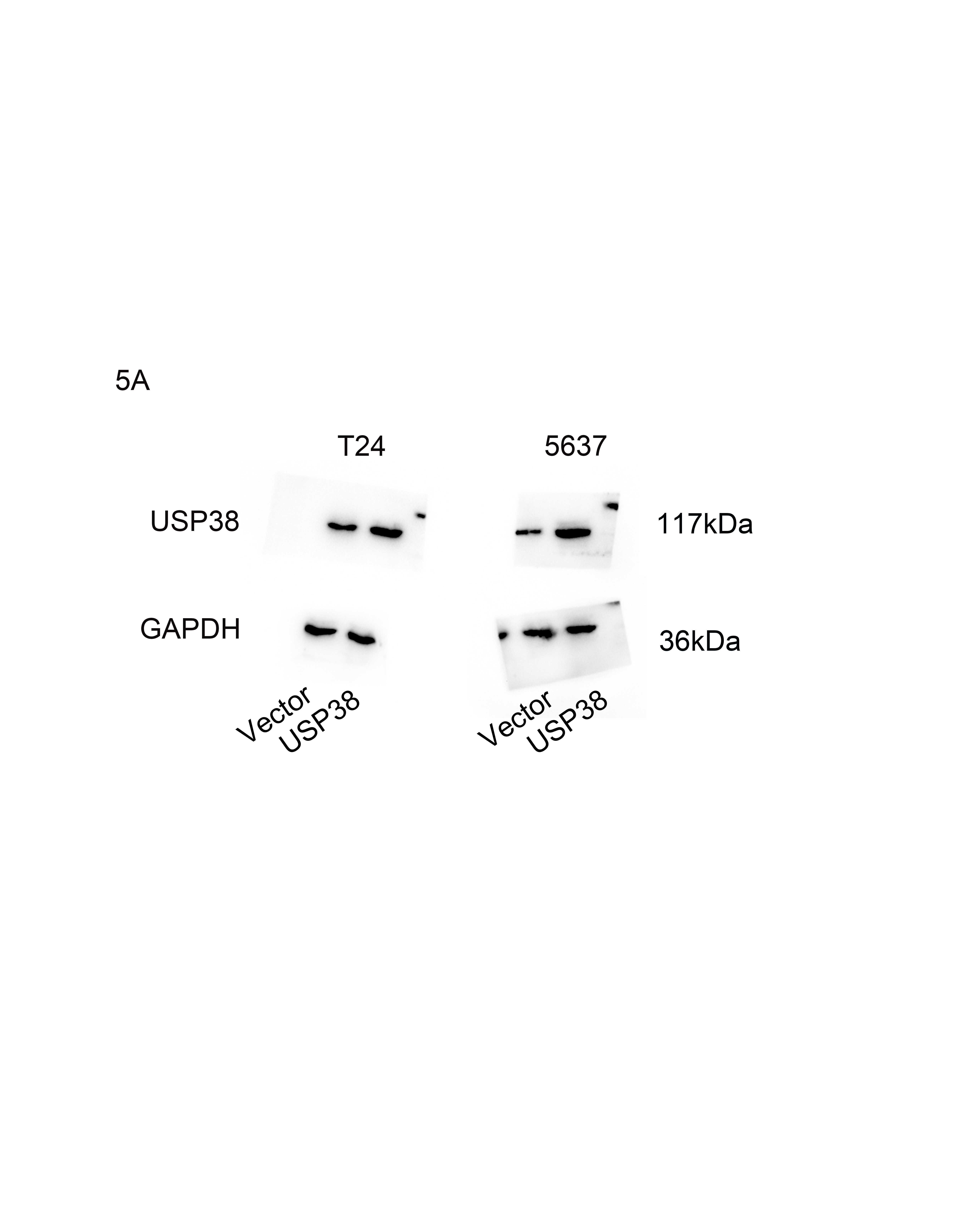

Supplement: S7 Data — (ZIP) [file pgen.1010366.s011.zip › Figure 5A.tif]

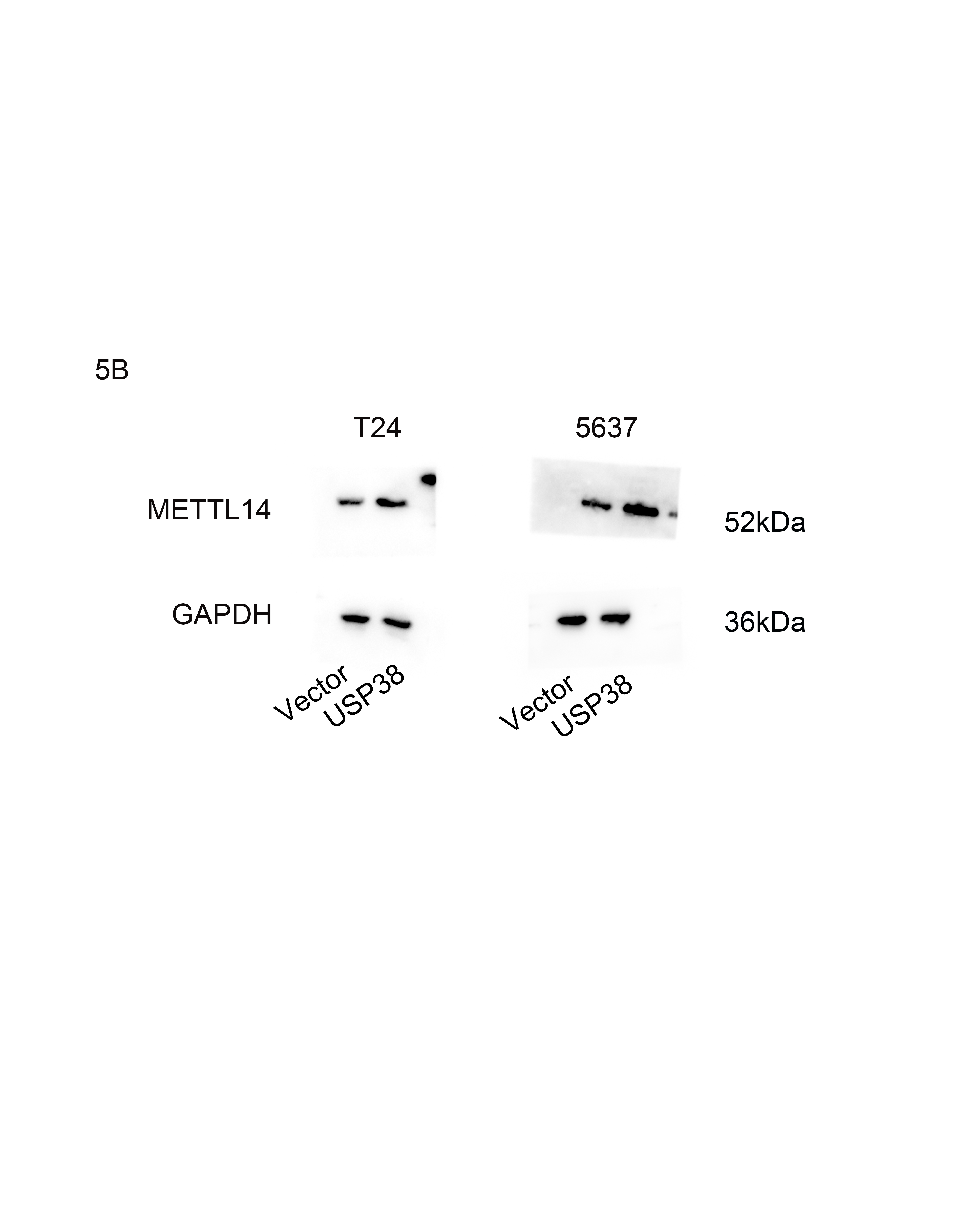

Supplement: S7 Data — (ZIP) [file pgen.1010366.s011.zip › Figure 5B.tif]

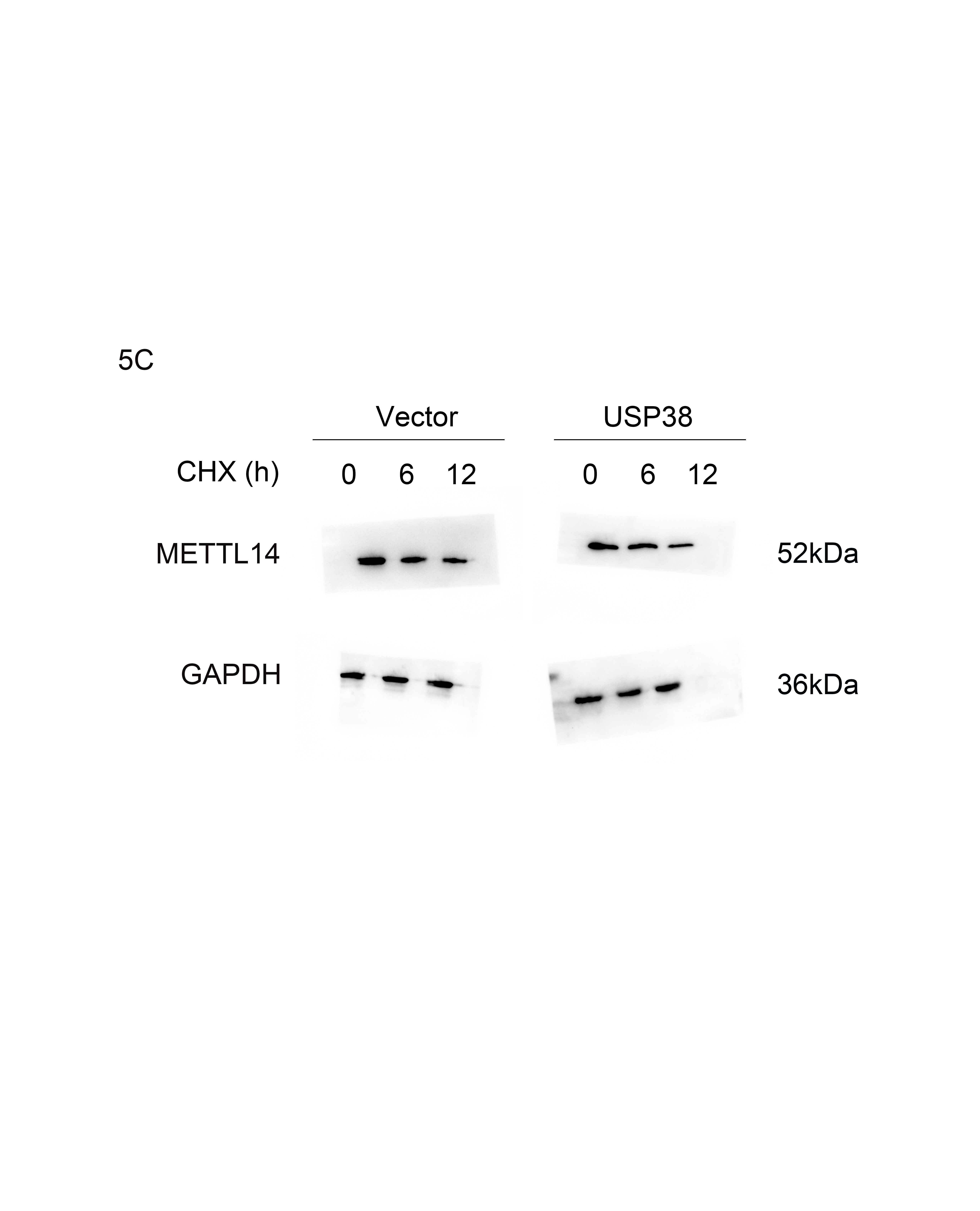

Supplement: S7 Data — (ZIP) [file pgen.1010366.s011.zip › Figure 5C.tif]

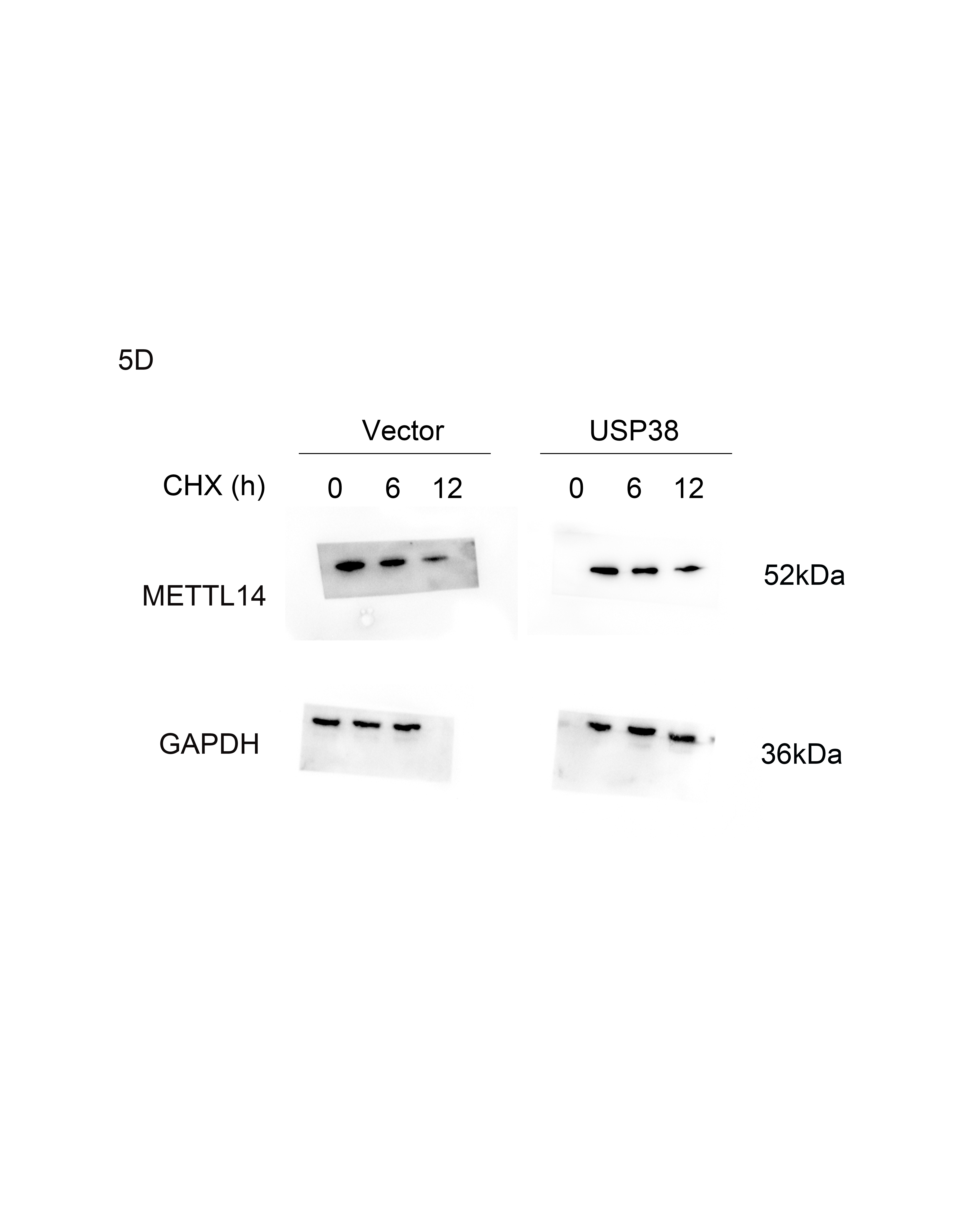

Supplement: S7 Data — (ZIP) [file pgen.1010366.s011.zip › Figure 5D.tif]

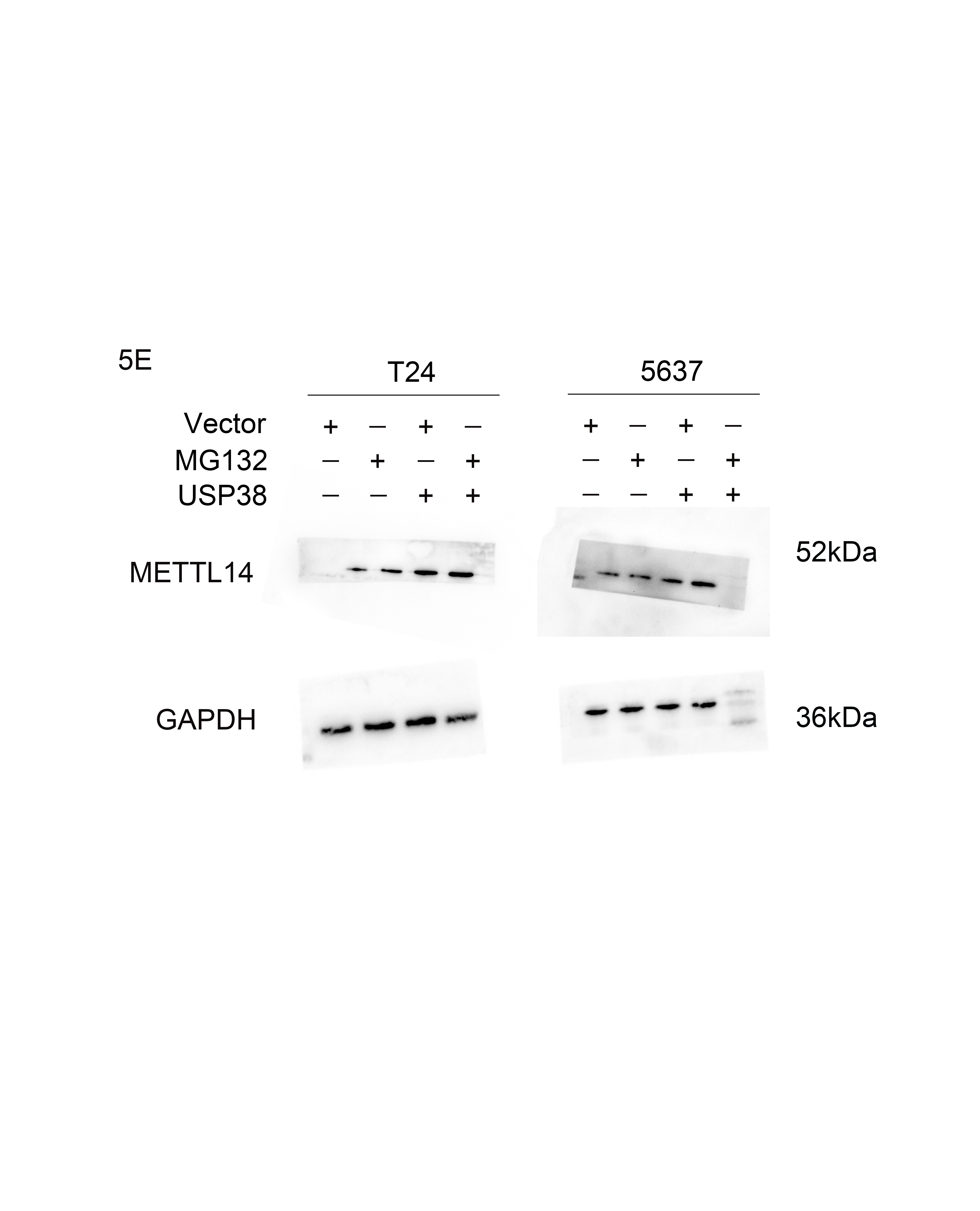

Supplement: S7 Data — (ZIP) [file pgen.1010366.s011.zip › Figure 5E.tif]
