# Supplementary figures and images for: The feedback loop of METTL14 and USP38 regulates cell migration, invasion and EMT as well as metastasis in bladder cancer (part 2 of 2)
Source: PLoS Genet. 2022 Oct 26;18(10):e1010366. doi: 10.1371/journal.pgen.1010366 (PMC9605029; doi:10.1371/journal.pgen.1010366)

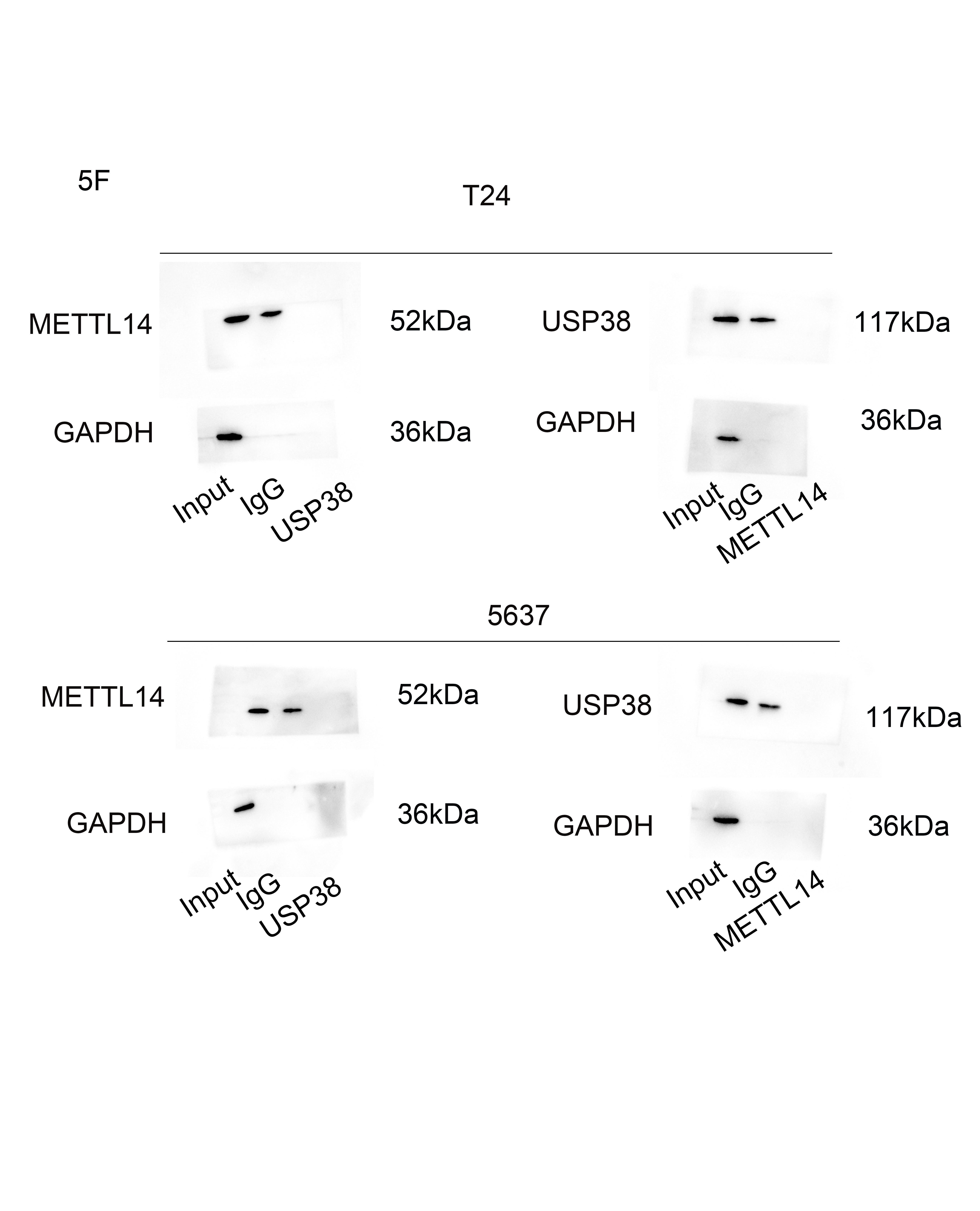

Supplement: S7 Data — (ZIP) [file pgen.1010366.s011.zip › Figure 5F.tif]

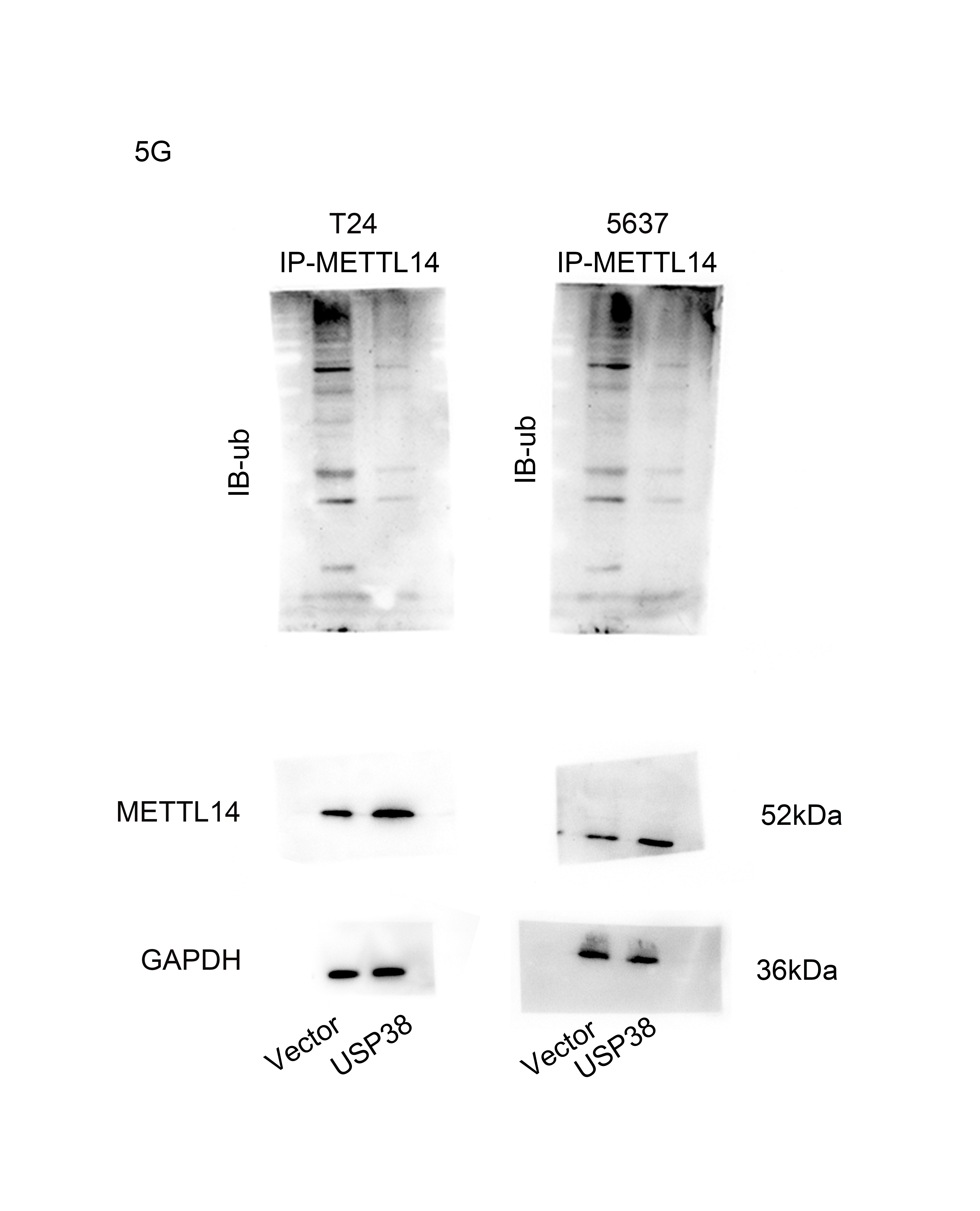

Supplement: S7 Data — (ZIP) [file pgen.1010366.s011.zip › Figure 5G.tif]

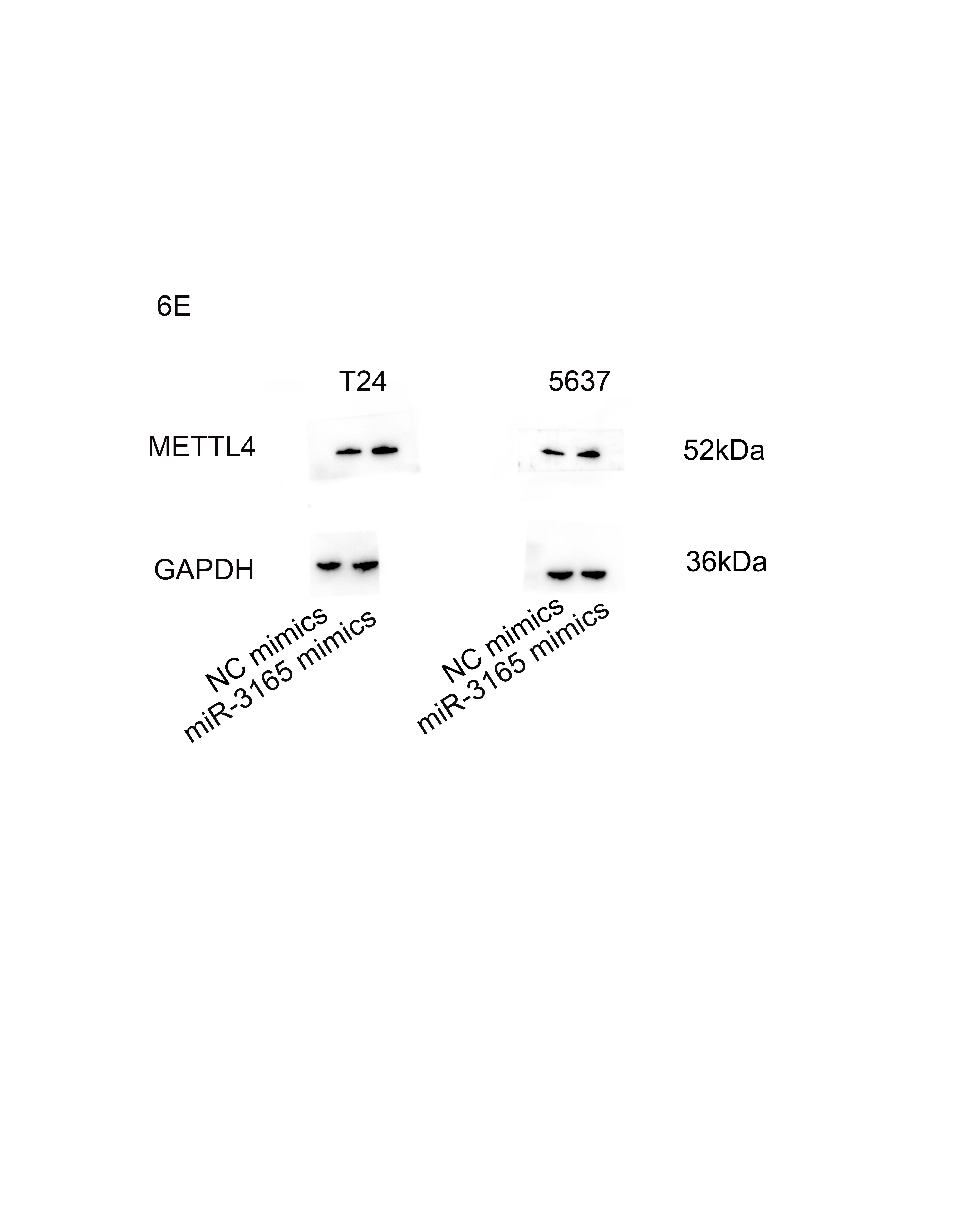

Supplement: S7 Data — (ZIP) [file pgen.1010366.s011.zip › Figure 6E.tif]

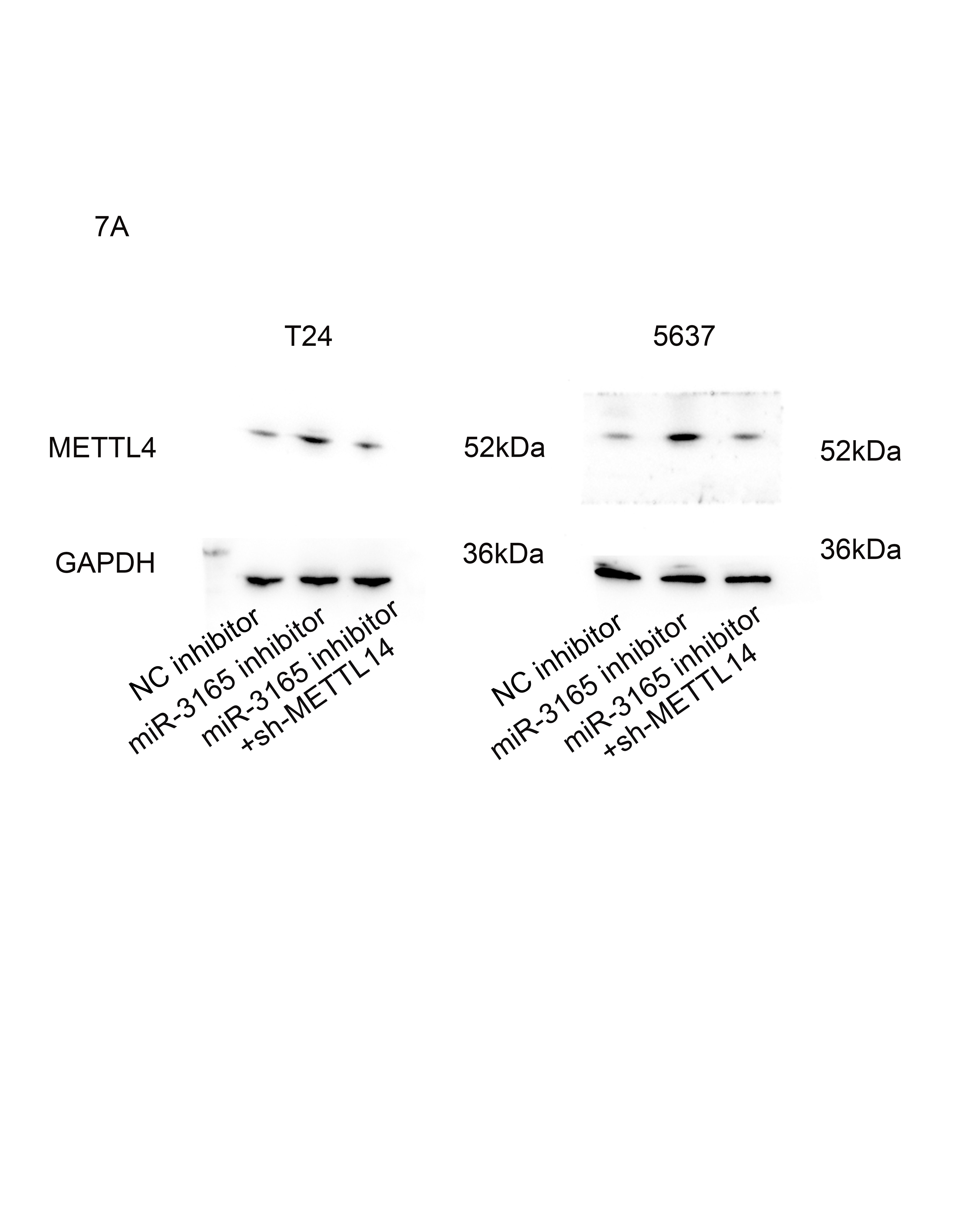

Supplement: S7 Data — (ZIP) [file pgen.1010366.s011.zip › Figure 7A.tif]

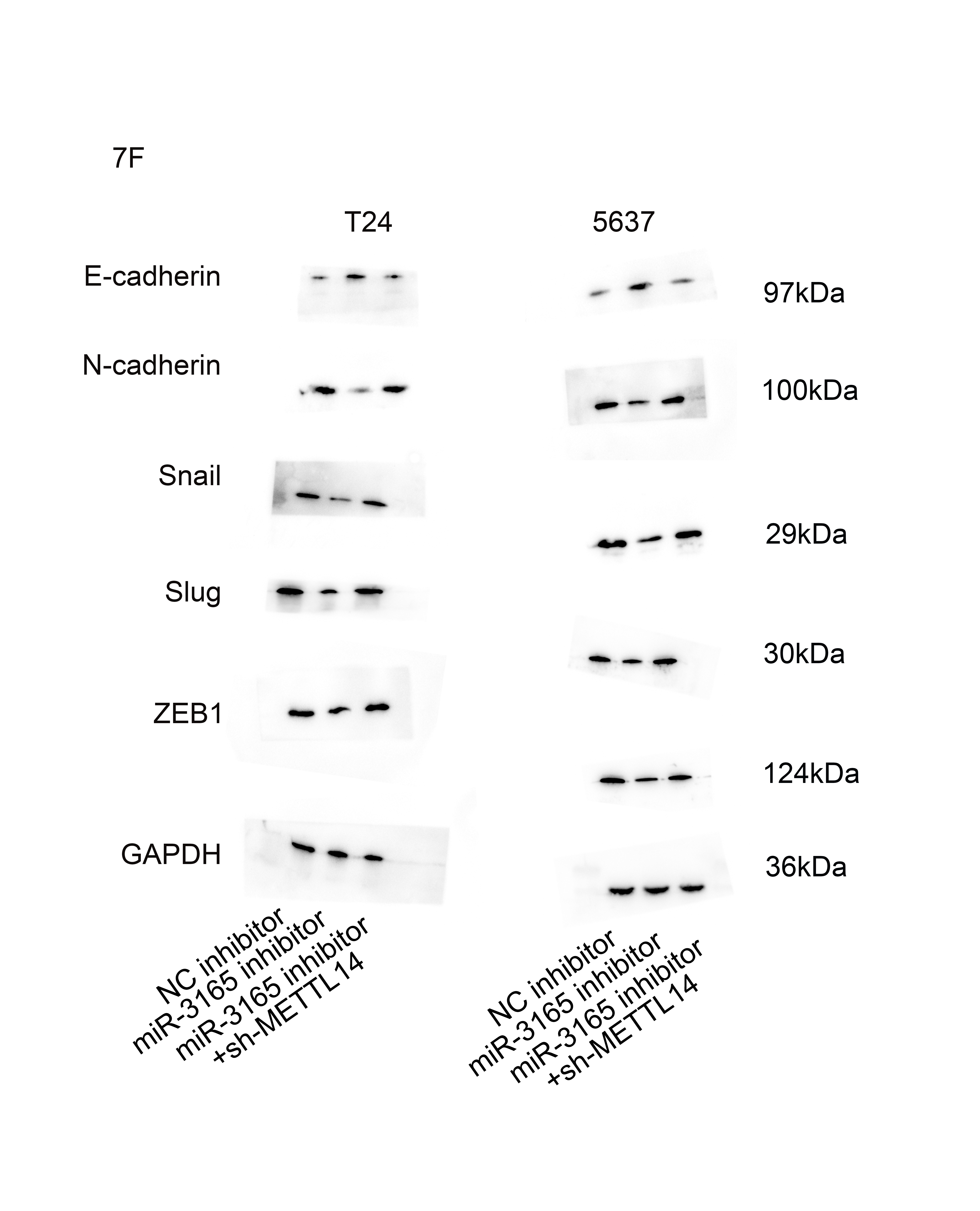

Supplement: S7 Data — (ZIP) [file pgen.1010366.s011.zip › Figure 7F.tif]

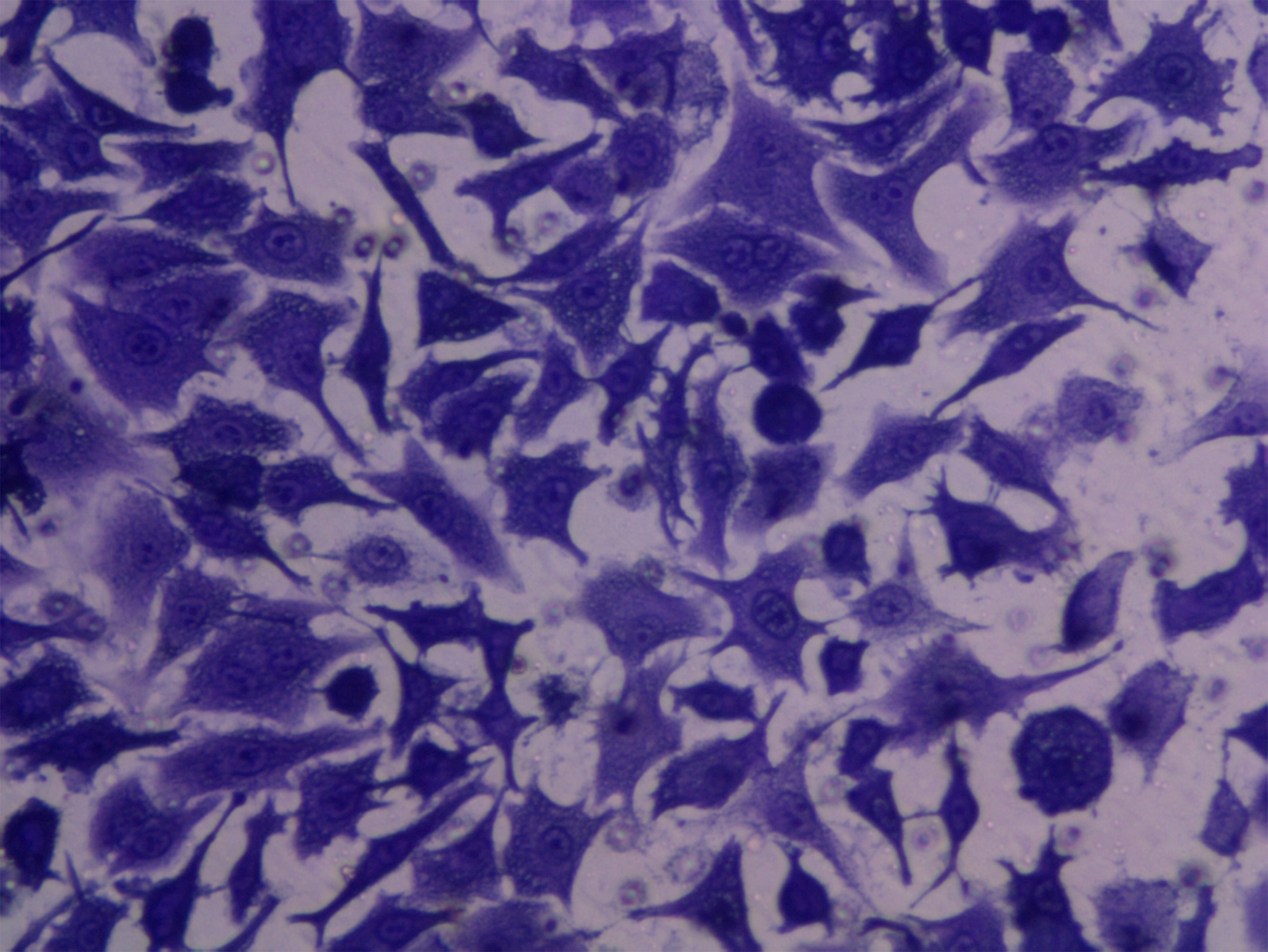

Supplement: S8 Data — (ZIP) [file pgen.1010366.s012.zip › S1A SV-HUC-1 Migration sh-METTL14.png]

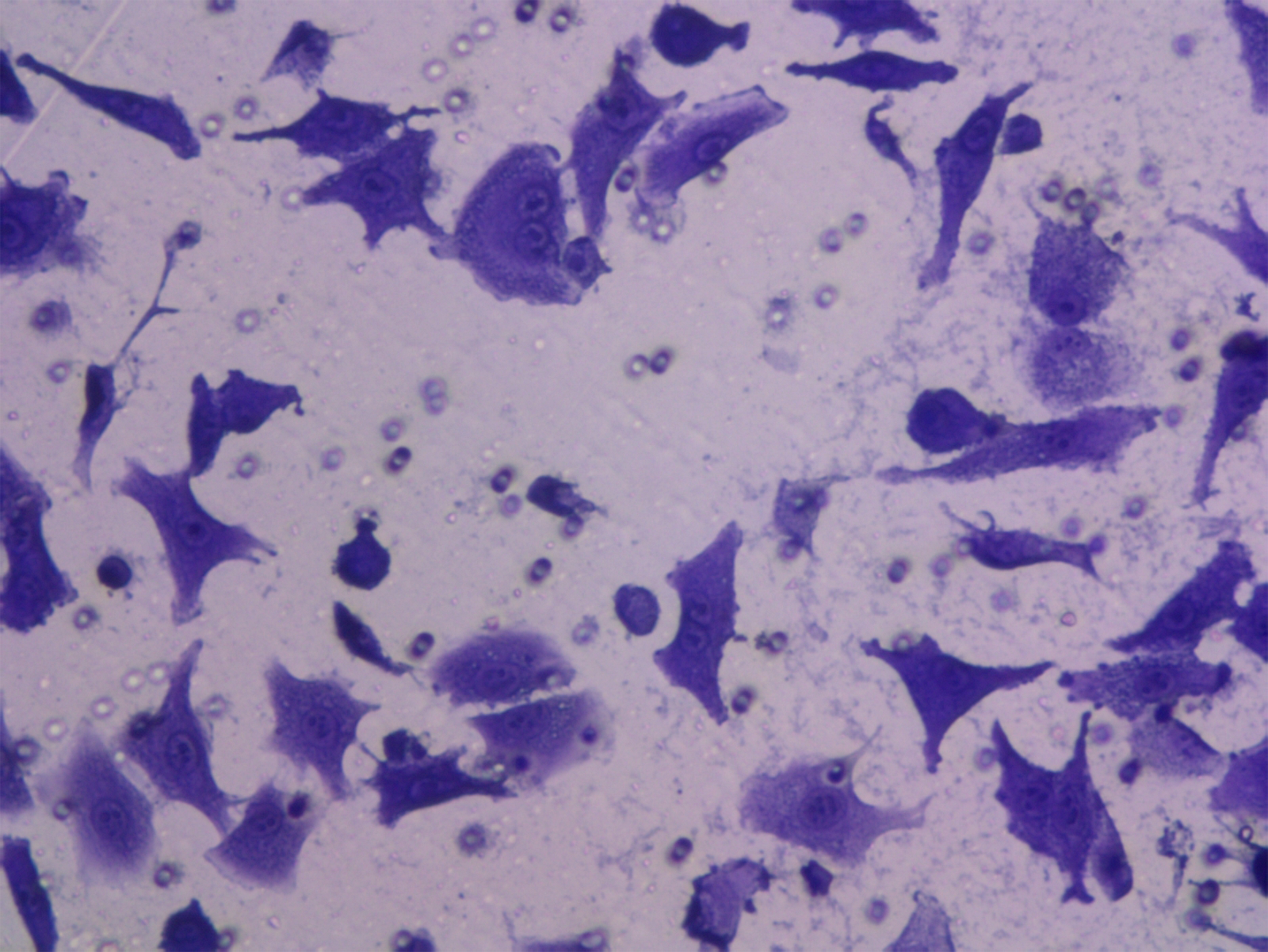

Supplement: S8 Data — (ZIP) [file pgen.1010366.s012.zip › S1A SV-HUC-1 Migration sh-NC.png]

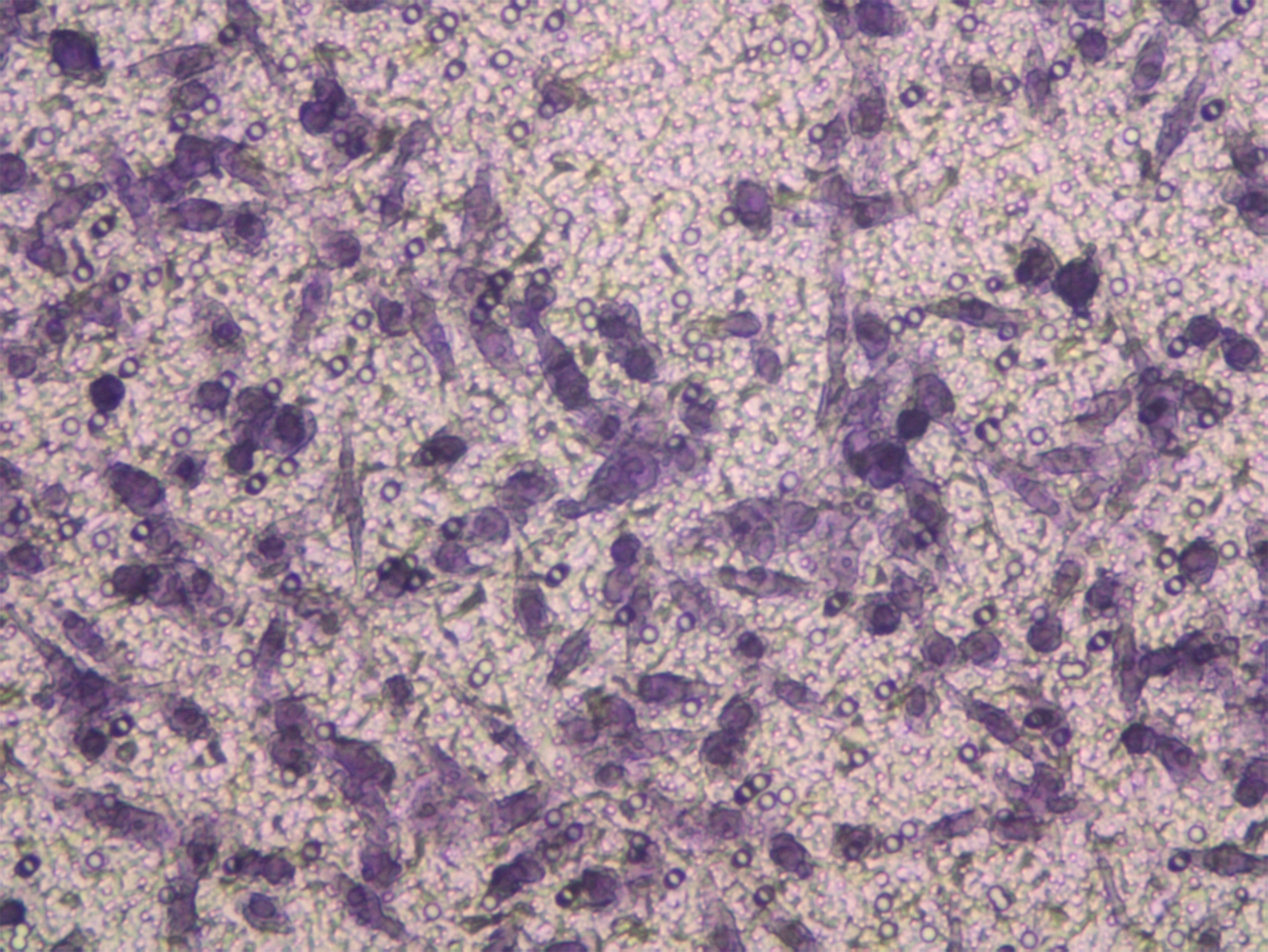

Supplement: S8 Data — (ZIP) [file pgen.1010366.s012.zip › S1A SW780 Migration sh-METTL14.png]

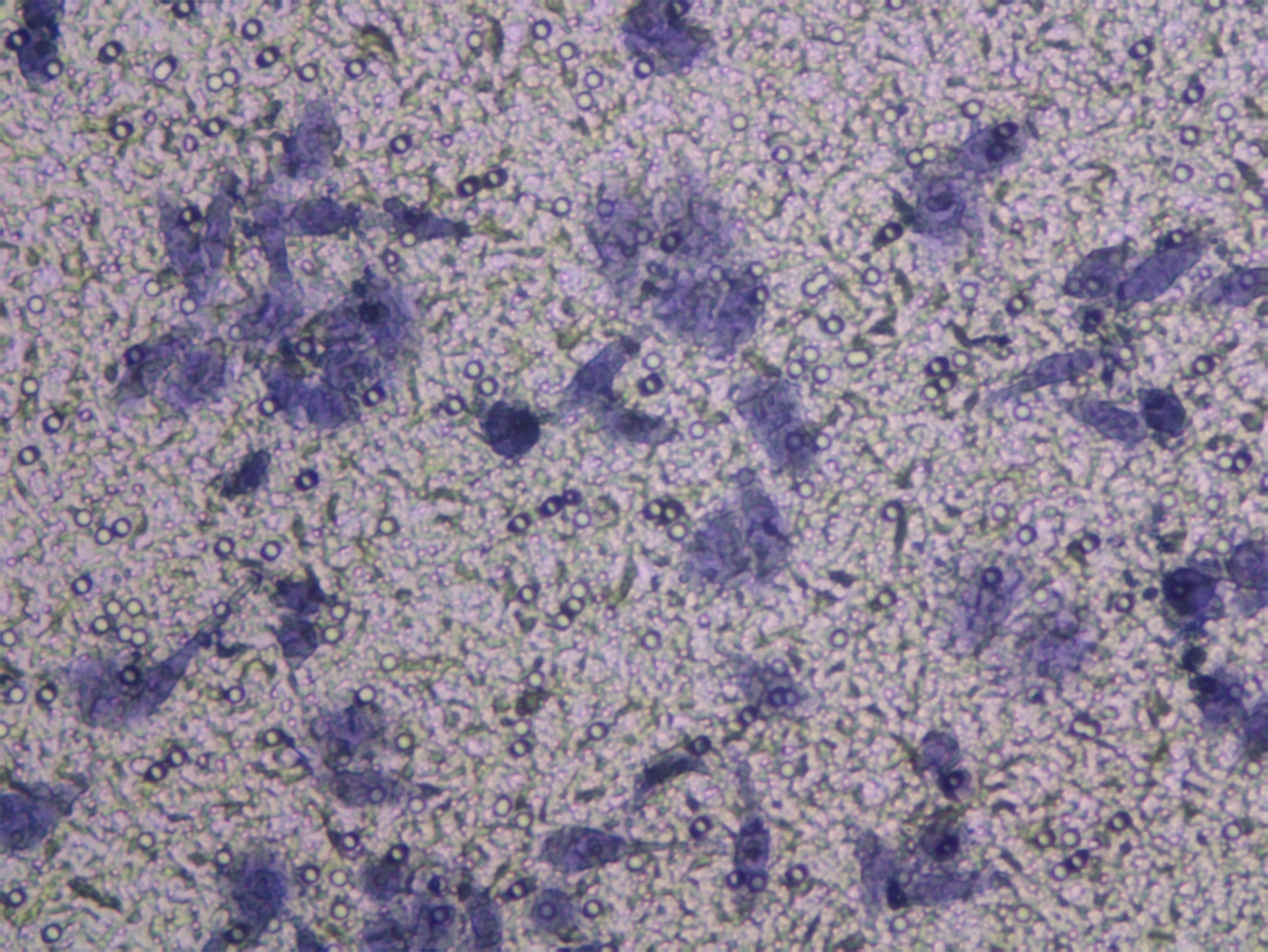

Supplement: S8 Data — (ZIP) [file pgen.1010366.s012.zip › S1A SW780 Migration sh-NC.png]

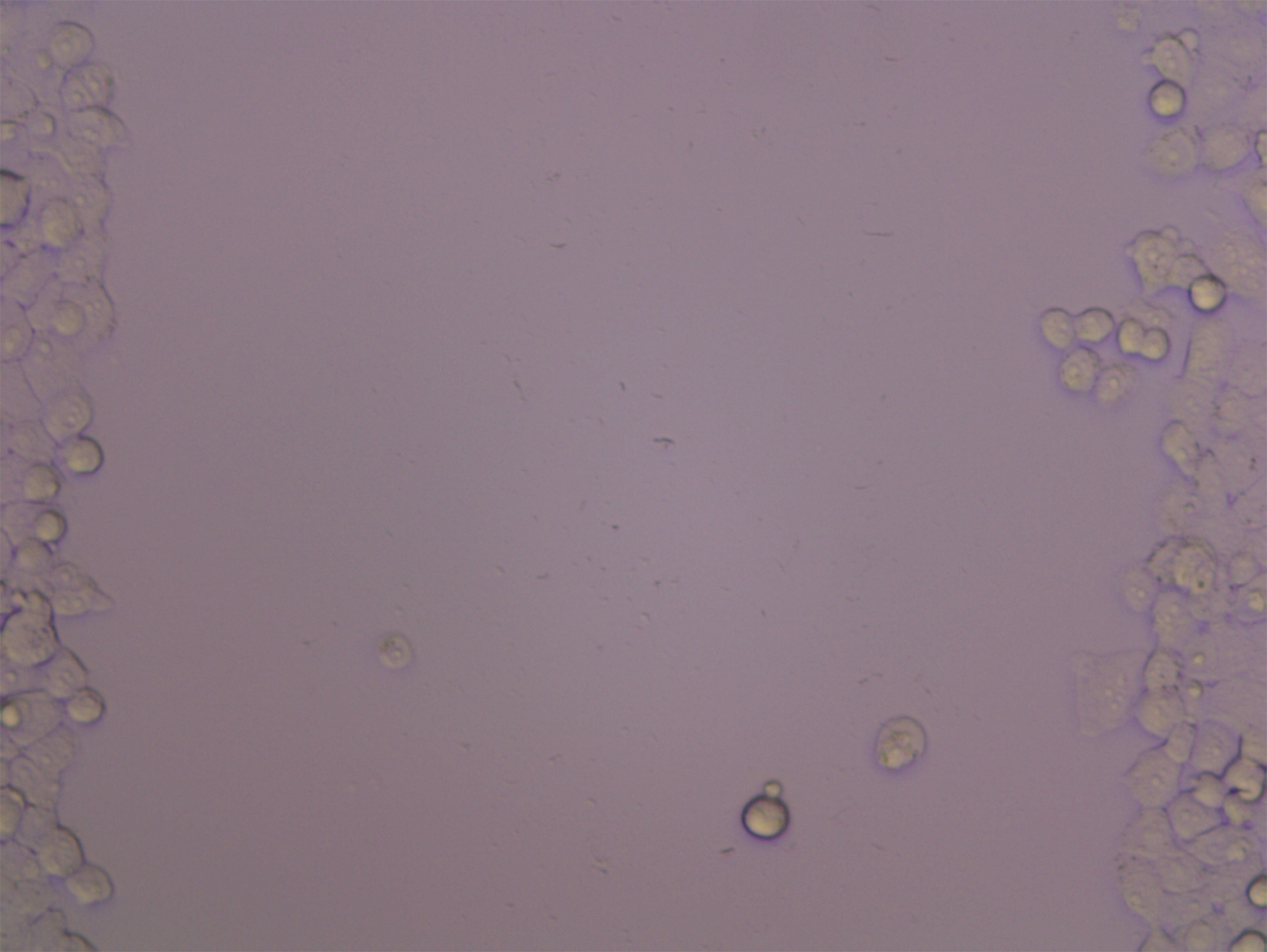

Supplement: S8 Data — (ZIP) [file pgen.1010366.s012.zip › S1B SV-HUC-1 0h sh-METTL14.png]

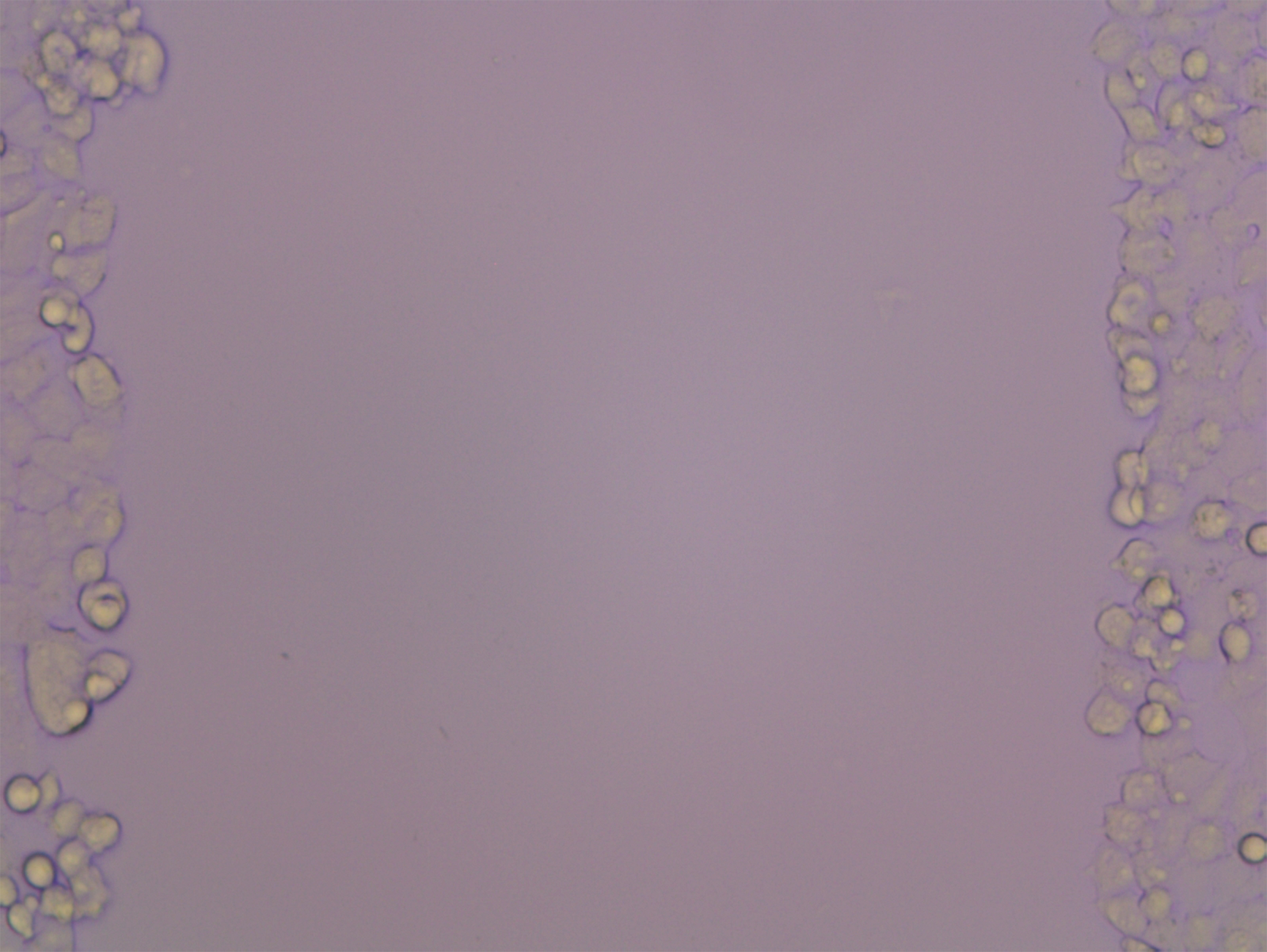

Supplement: S8 Data — (ZIP) [file pgen.1010366.s012.zip › S1B SV-HUC-1 0h sh-NC.png]

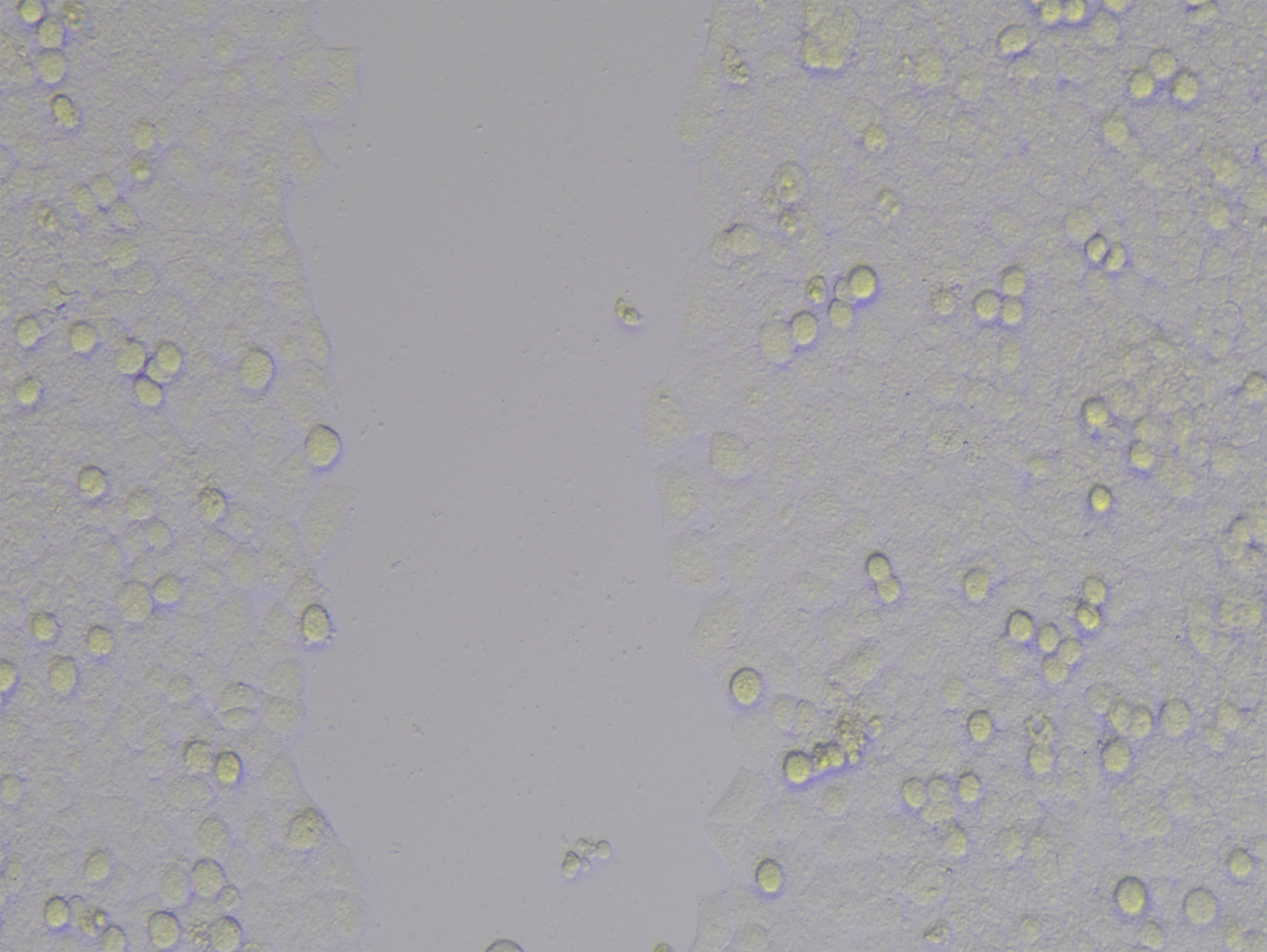

Supplement: S8 Data — (ZIP) [file pgen.1010366.s012.zip › S1B SV-HUC-1 24h sh-METTL14.png]

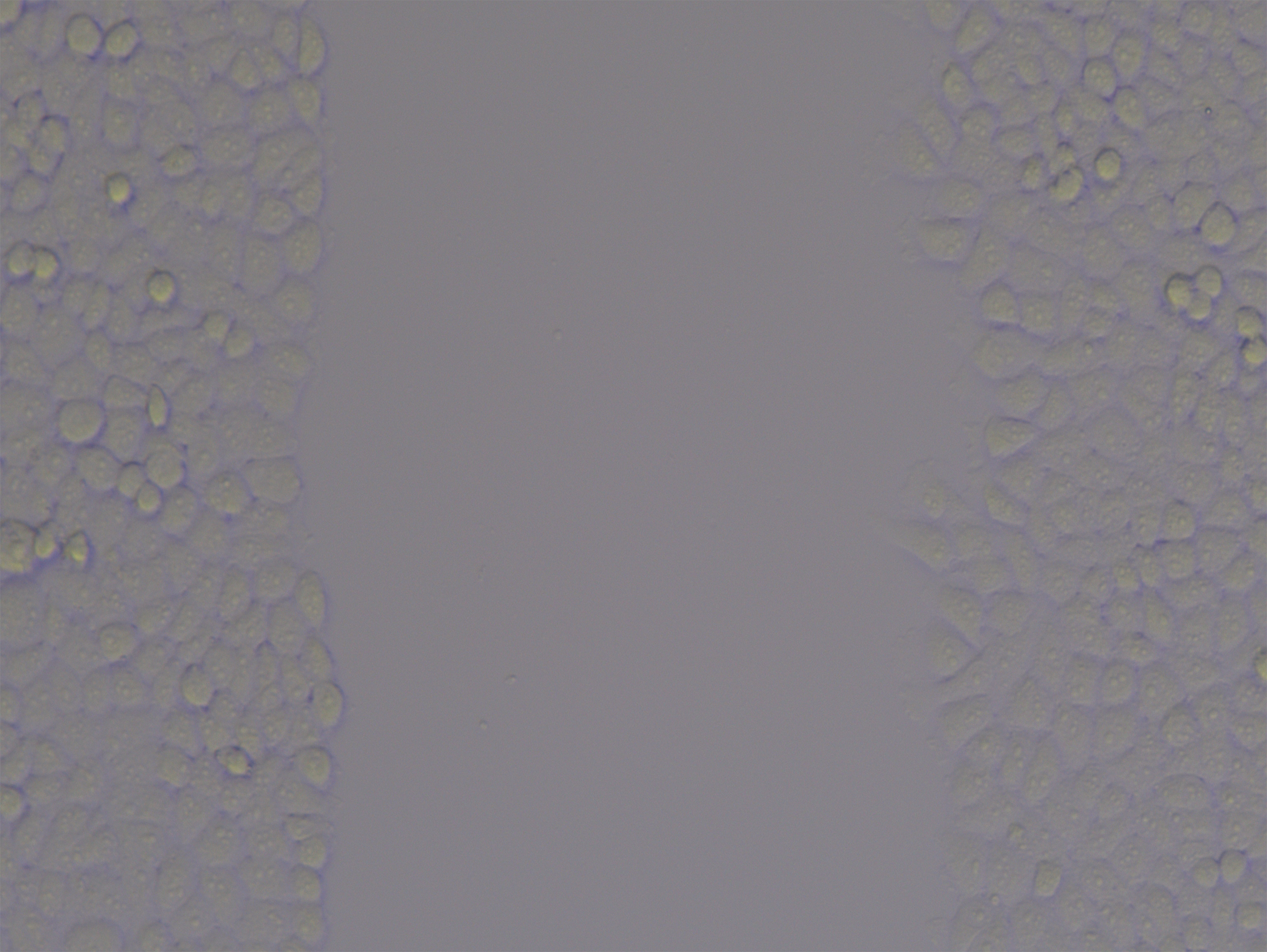

Supplement: S8 Data — (ZIP) [file pgen.1010366.s012.zip › S1B SV-HUC-1 24h sh-NC.png]

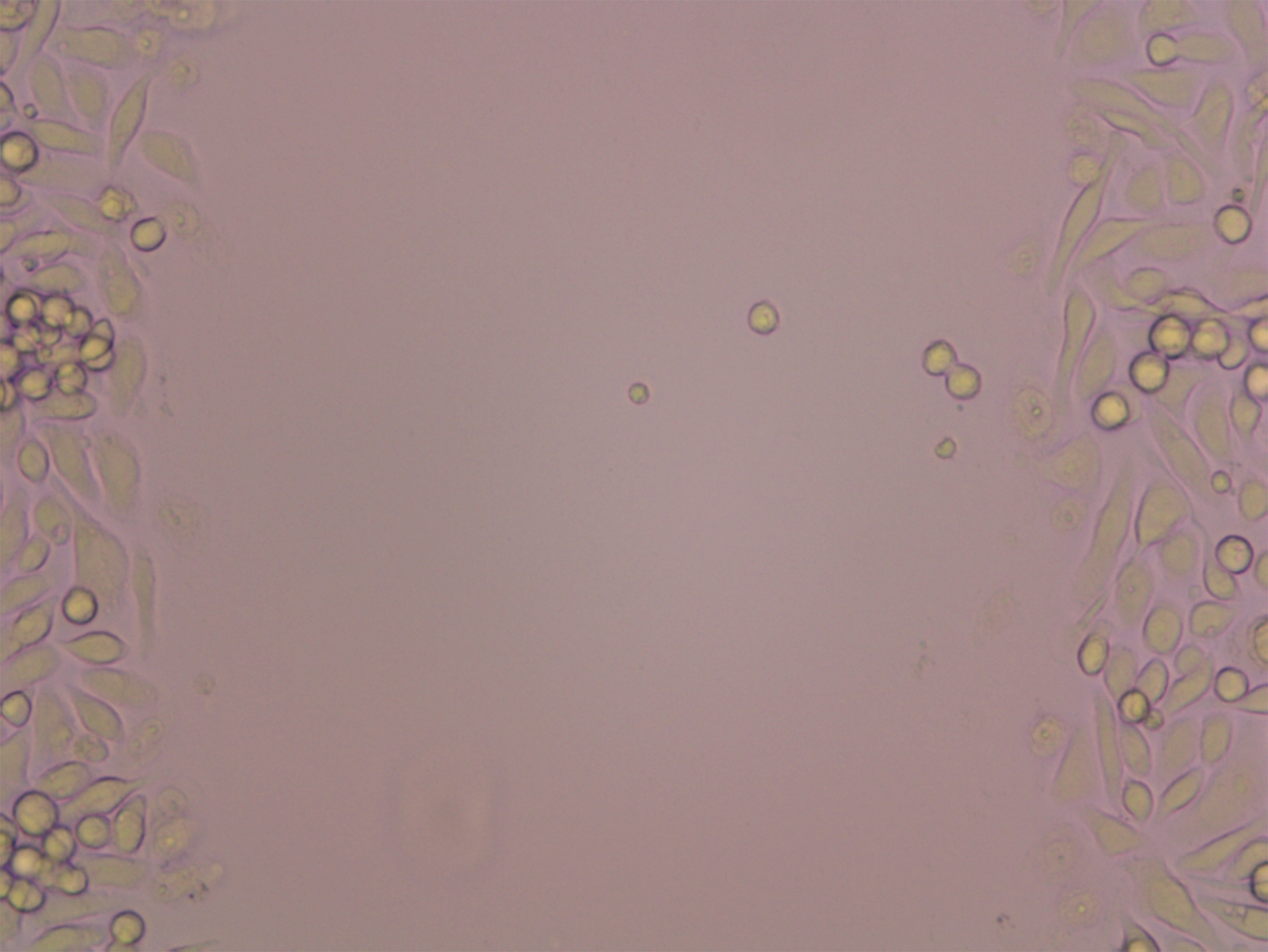

Supplement: S8 Data — (ZIP) [file pgen.1010366.s012.zip › S1B SW780 0h sh-METTL14.png]

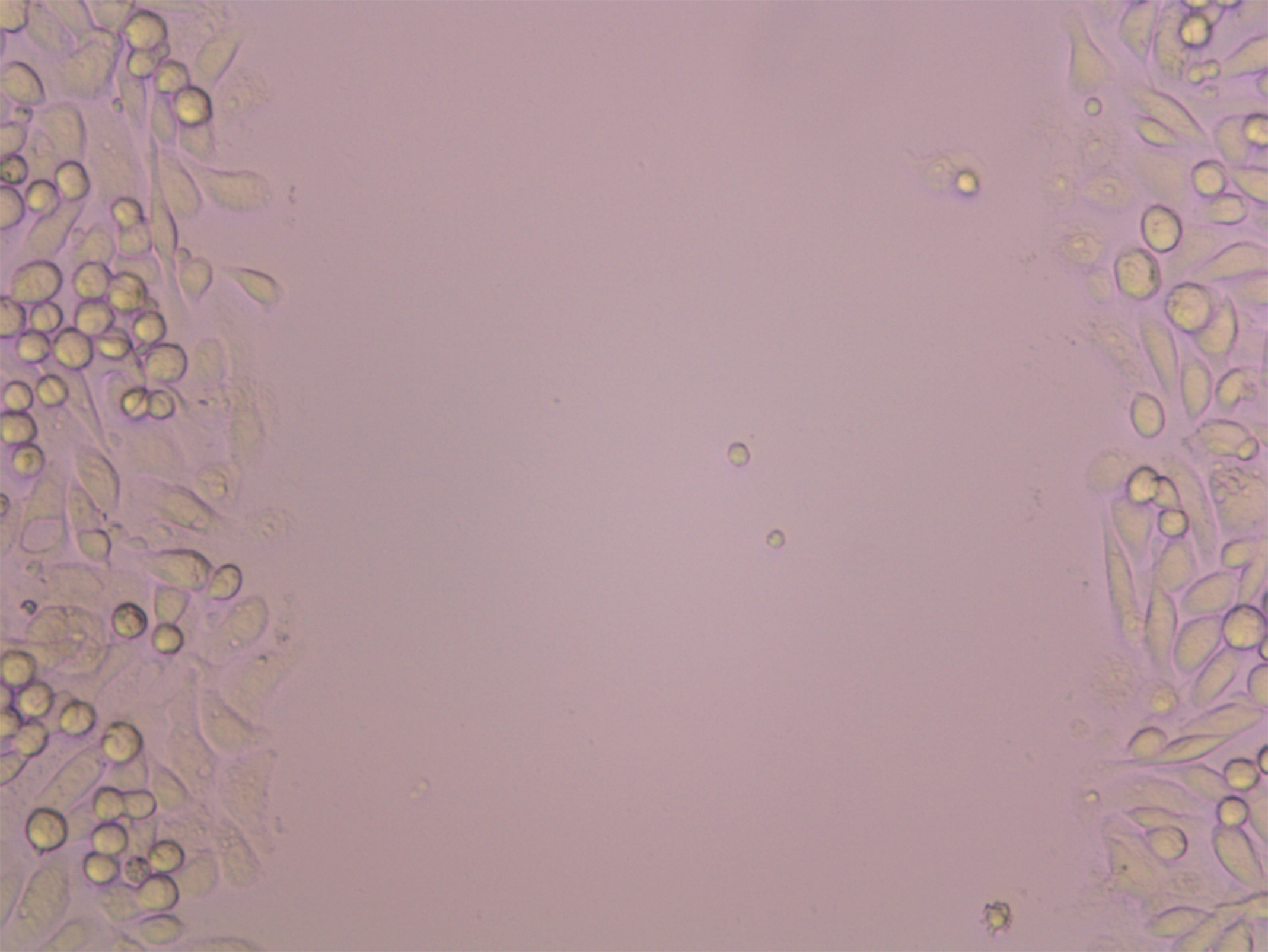

Supplement: S8 Data — (ZIP) [file pgen.1010366.s012.zip › S1B SW780 0h sh-NC.png]

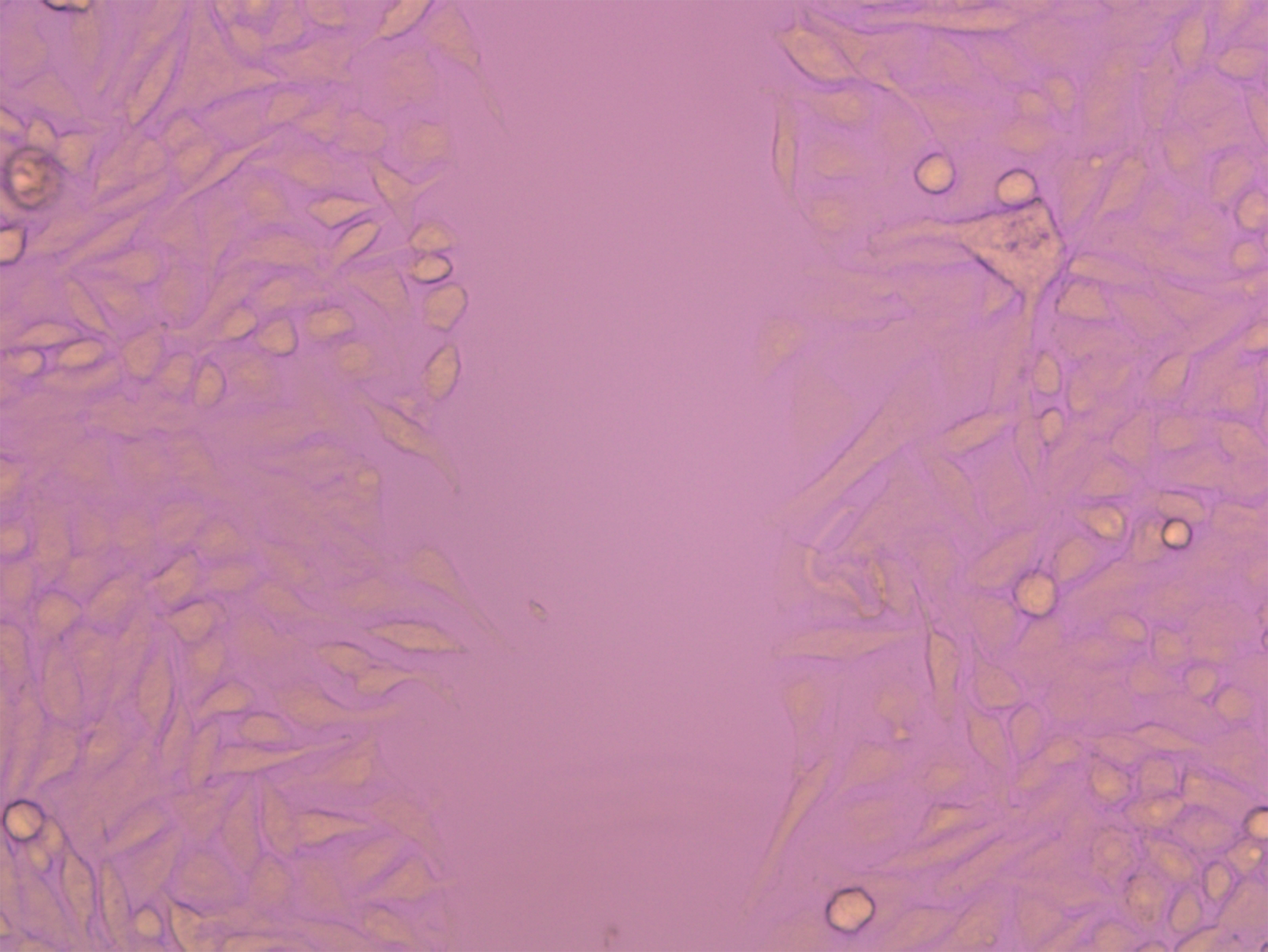

Supplement: S8 Data — (ZIP) [file pgen.1010366.s012.zip › S1B SW780 24h sh-METTL14.png]

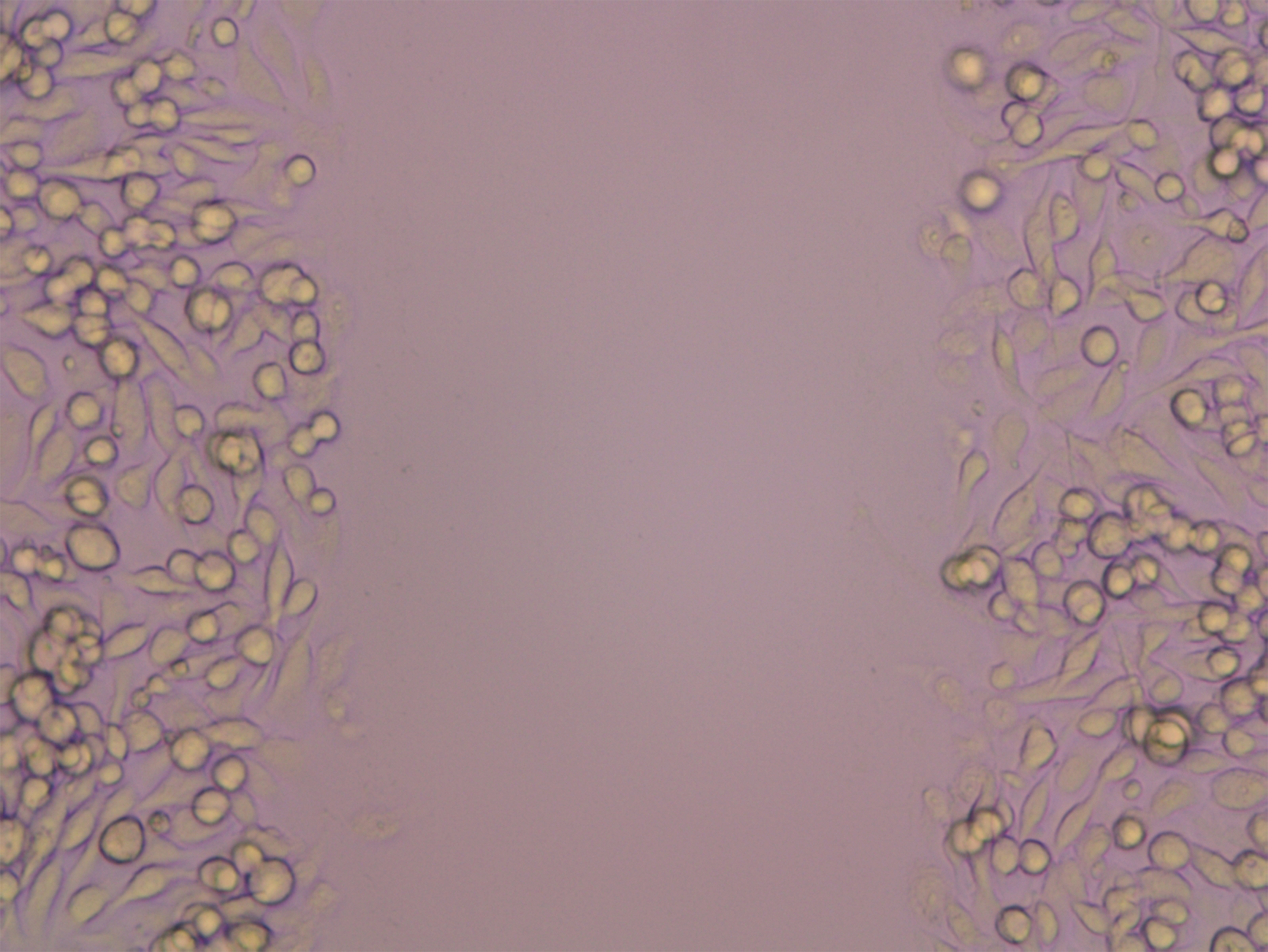

Supplement: S8 Data — (ZIP) [file pgen.1010366.s012.zip › S1B SW780 24h sh-NC.png]

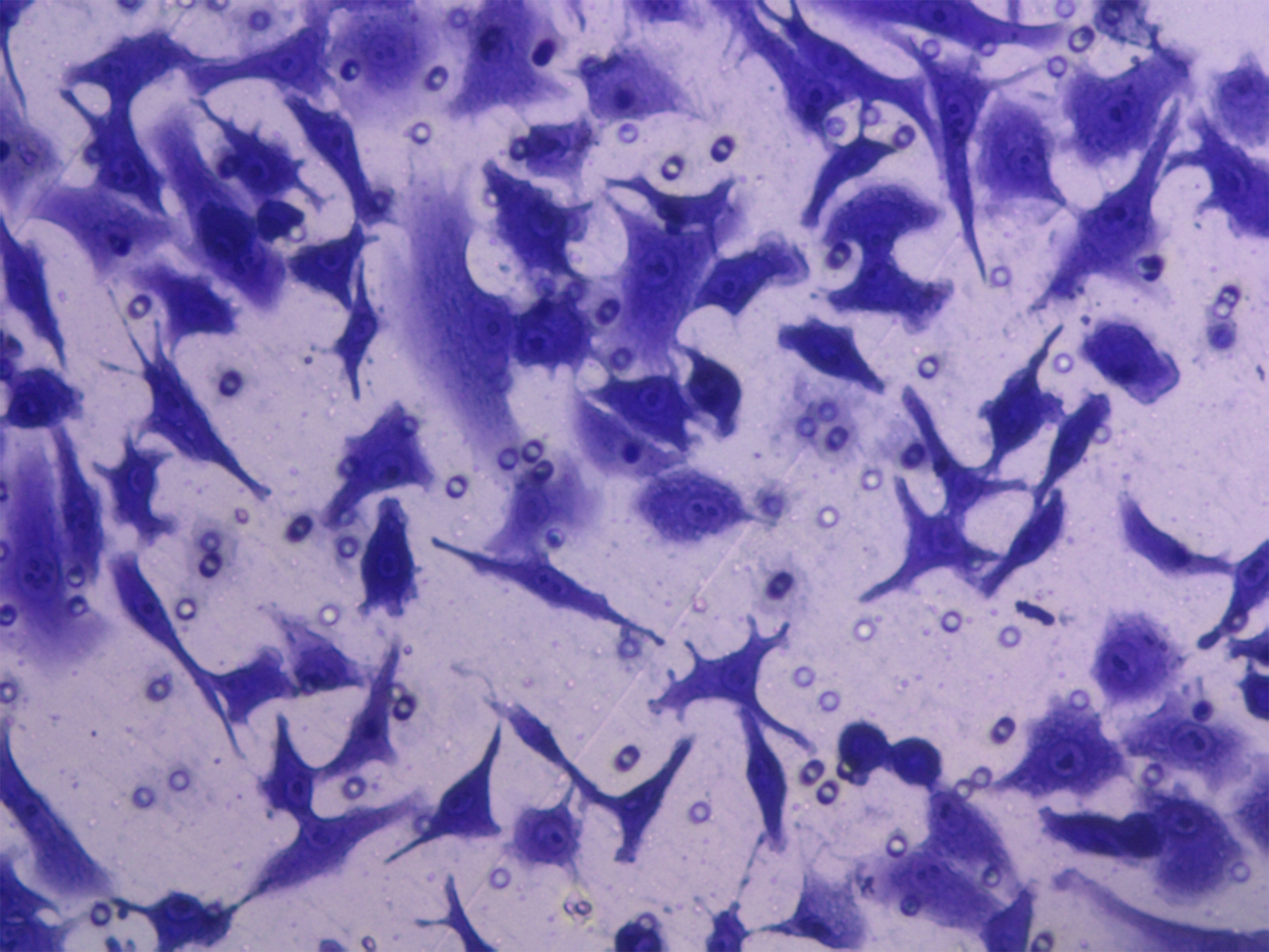

Supplement: S8 Data — (ZIP) [file pgen.1010366.s012.zip › S1C SV-HUC-1 Invasion sh-METTL14.png]

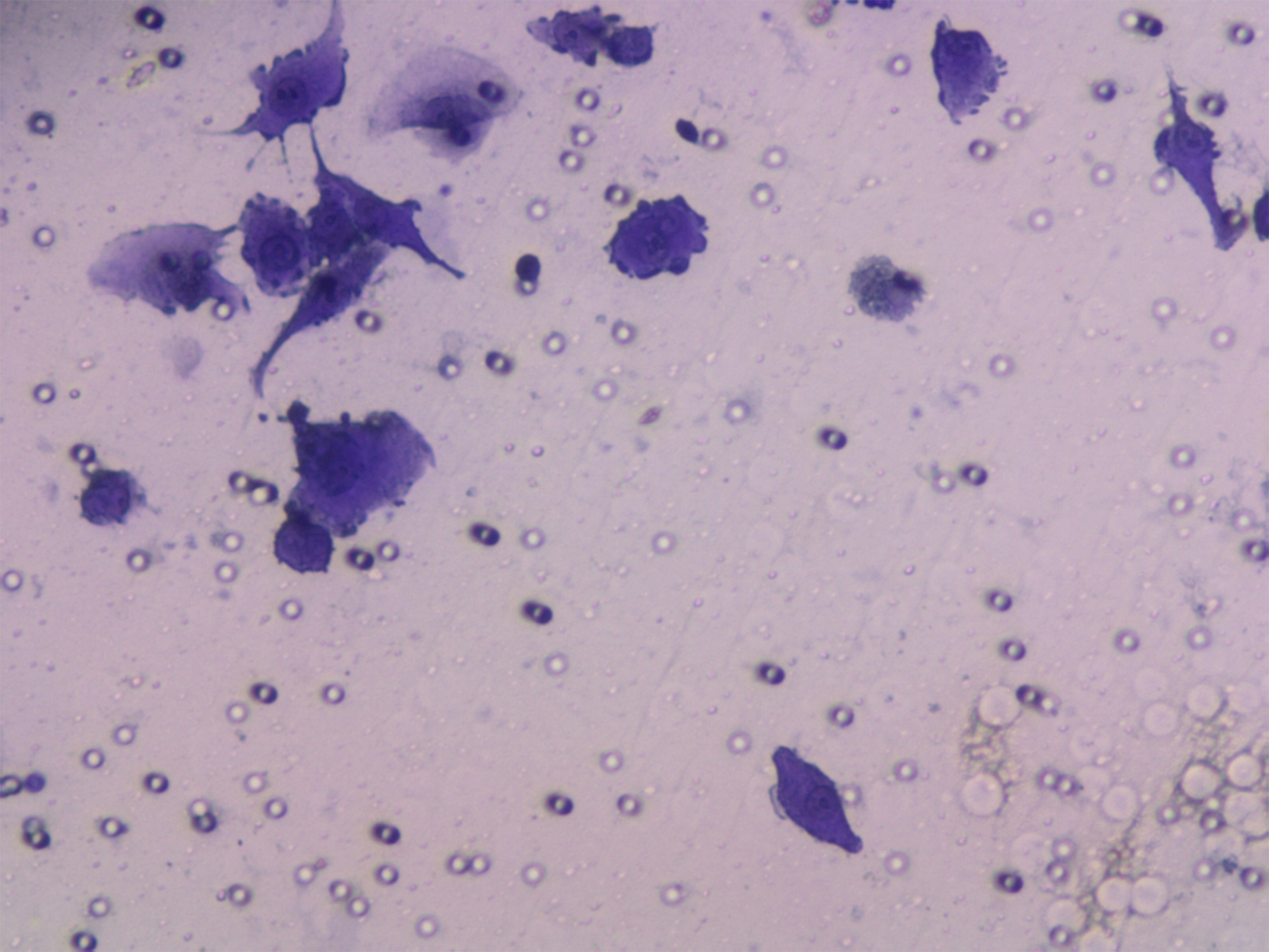

Supplement: S8 Data — (ZIP) [file pgen.1010366.s012.zip › S1C SV-HUC-1 Invasion sh-NC.png]

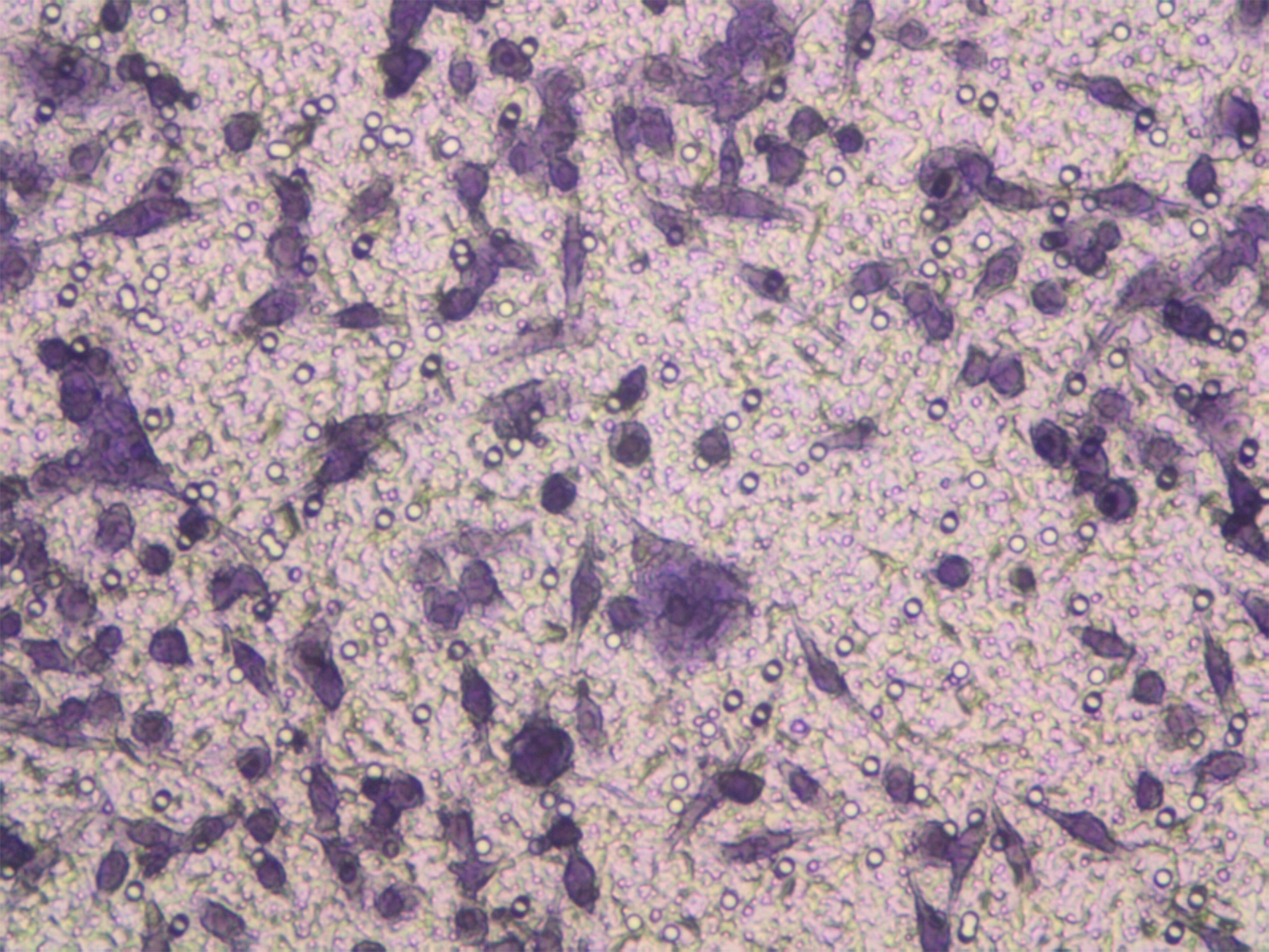

Supplement: S8 Data — (ZIP) [file pgen.1010366.s012.zip › S1C SW780 Invasion sh-METTL14.png]

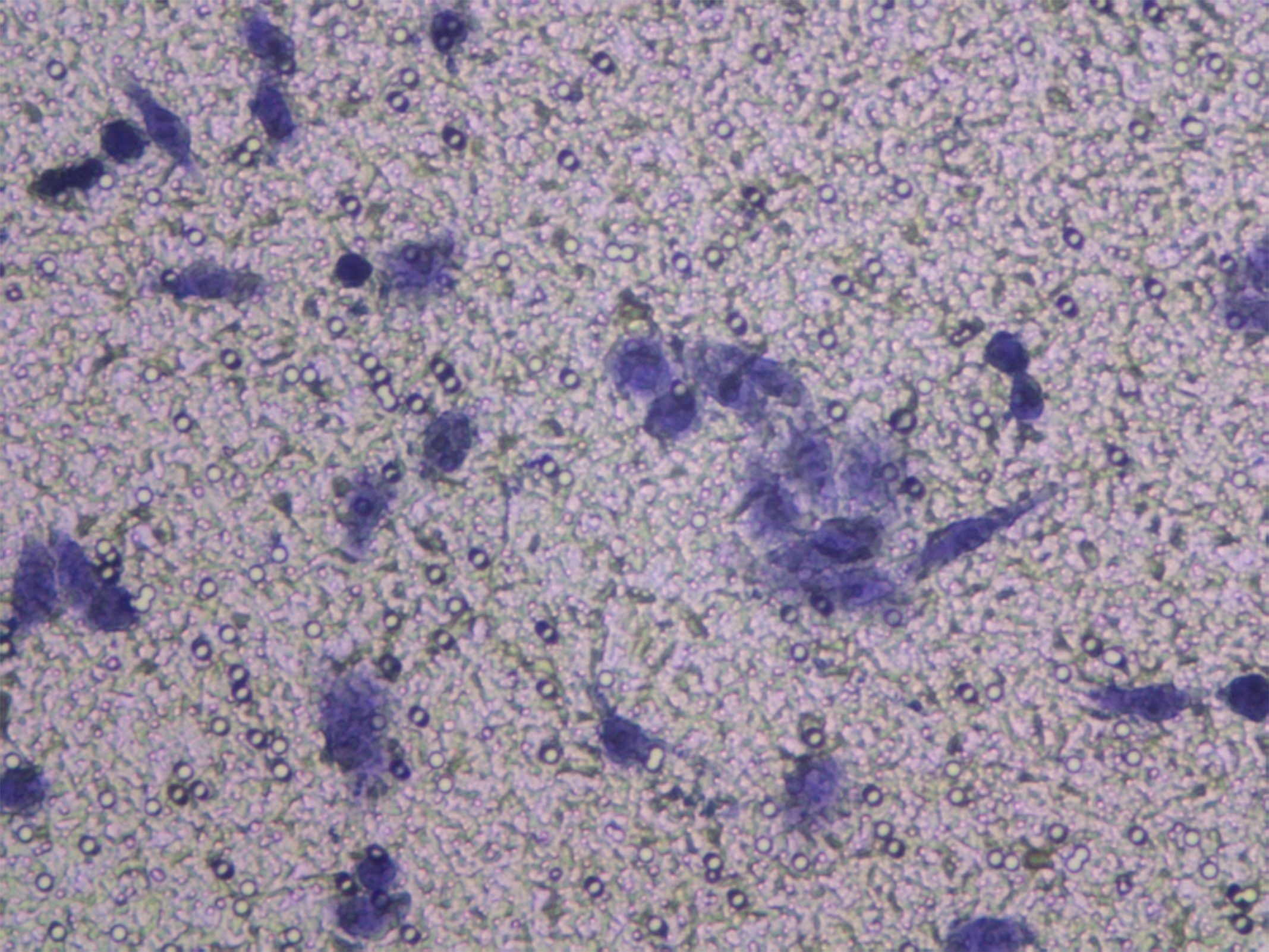

Supplement: S8 Data — (ZIP) [file pgen.1010366.s012.zip › S1C SW780 Invasion sh-NC.png]

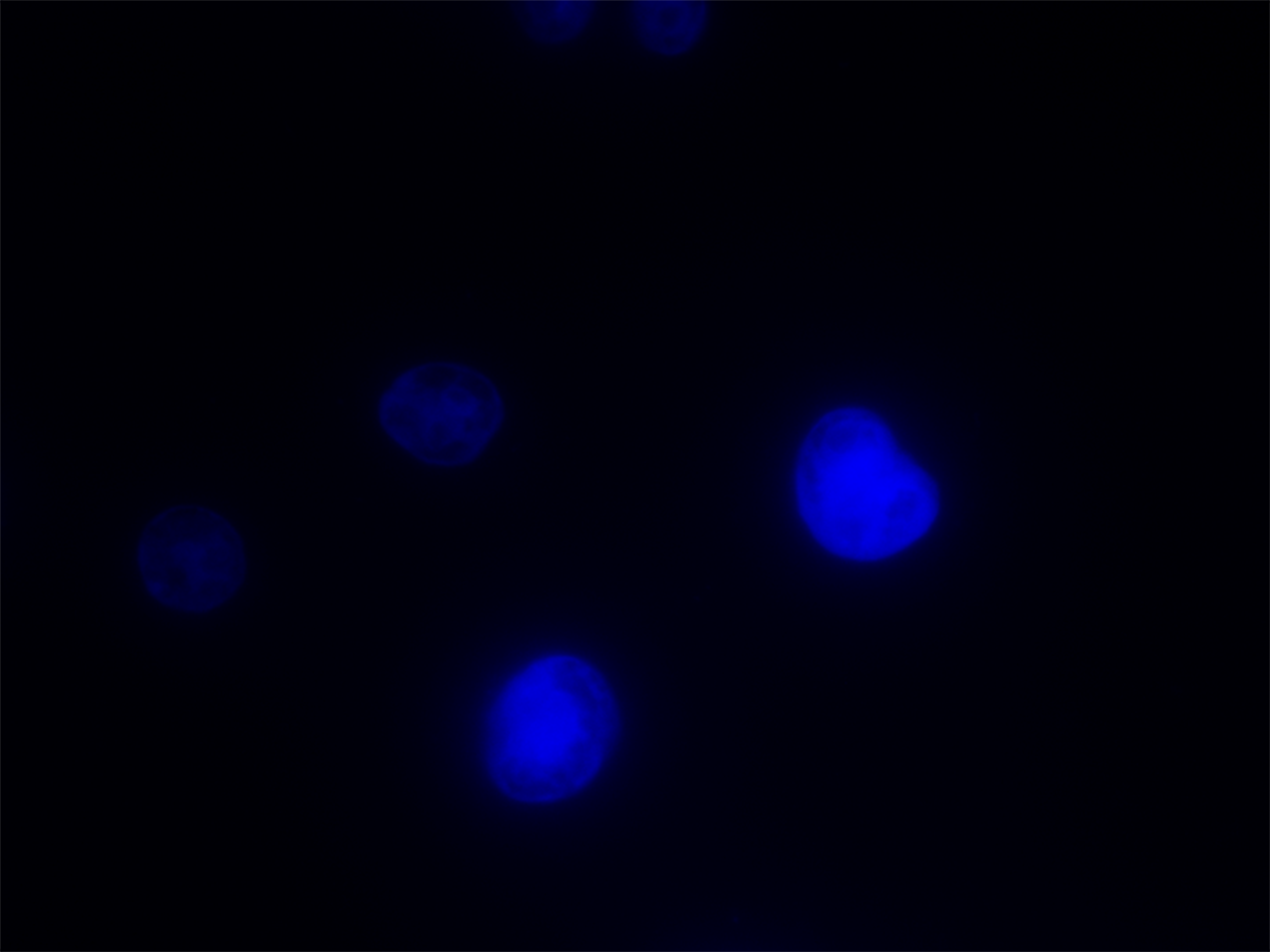

Supplement: S8 Data — (ZIP) [file pgen.1010366.s012.zip › S1D SV-HUC-1 sh-METTL14 DAPI.png]

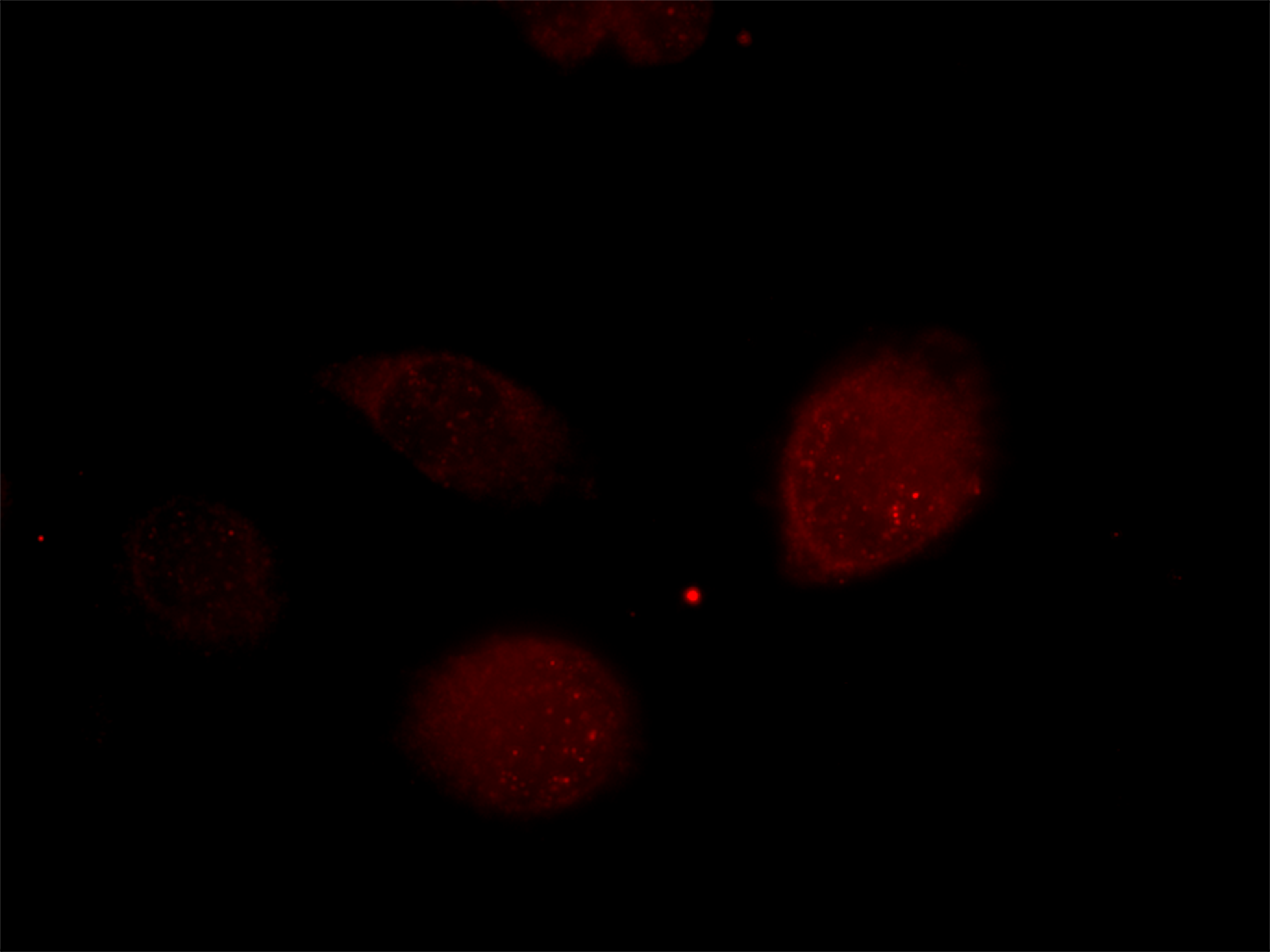

Supplement: S8 Data — (ZIP) [file pgen.1010366.s012.zip › S1D SV-HUC-1 sh-METTL14 E-cadherin.png]

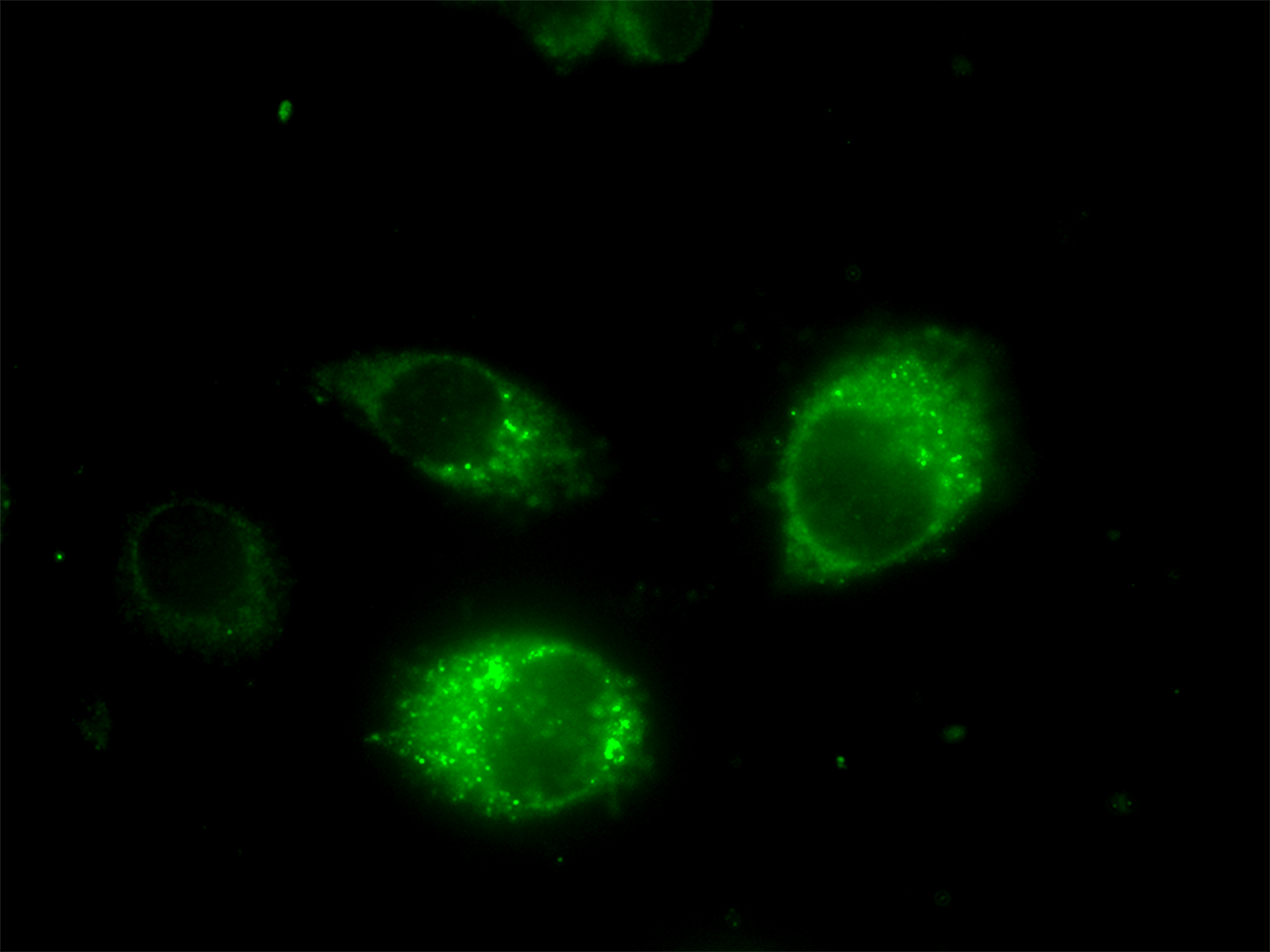

Supplement: S8 Data — (ZIP) [file pgen.1010366.s012.zip › S1D SV-HUC-1 sh-METTL14 N-cadherin.png]

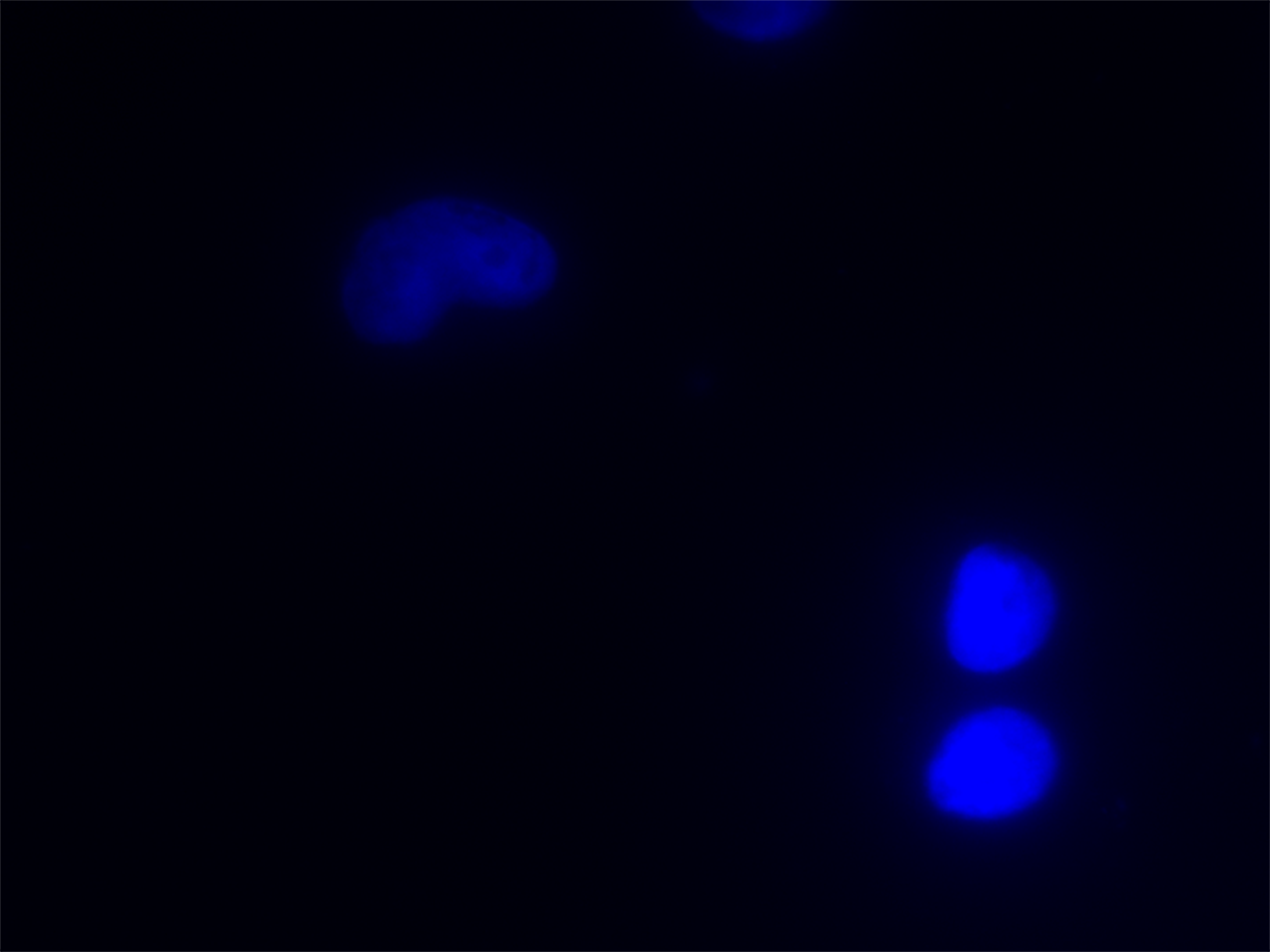

Supplement: S8 Data — (ZIP) [file pgen.1010366.s012.zip › S1D SV-HUC-1 sh-NC DAPI.png]

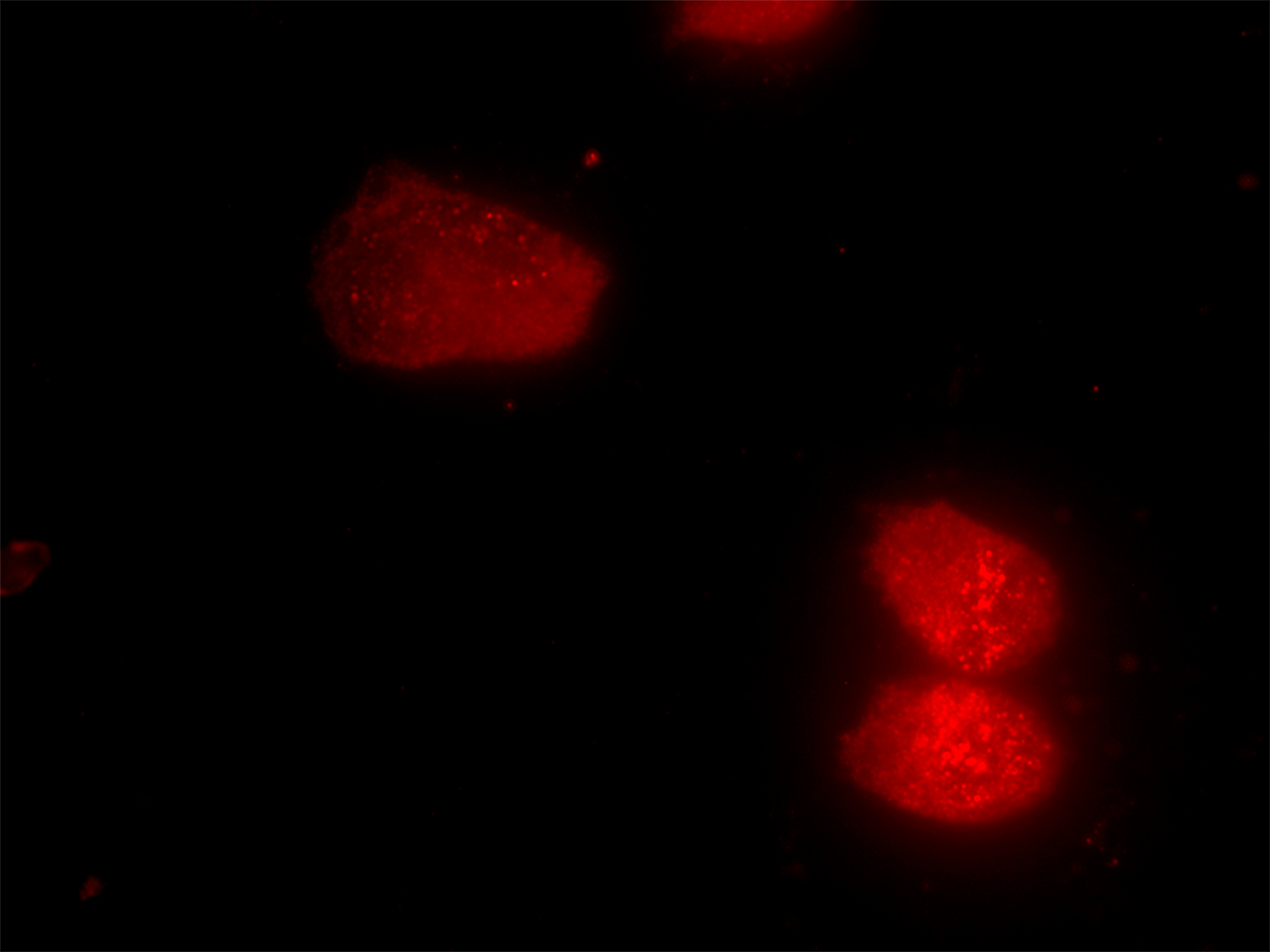

Supplement: S8 Data — (ZIP) [file pgen.1010366.s012.zip › S1D SV-HUC-1 sh-NC E-cadherin.png]

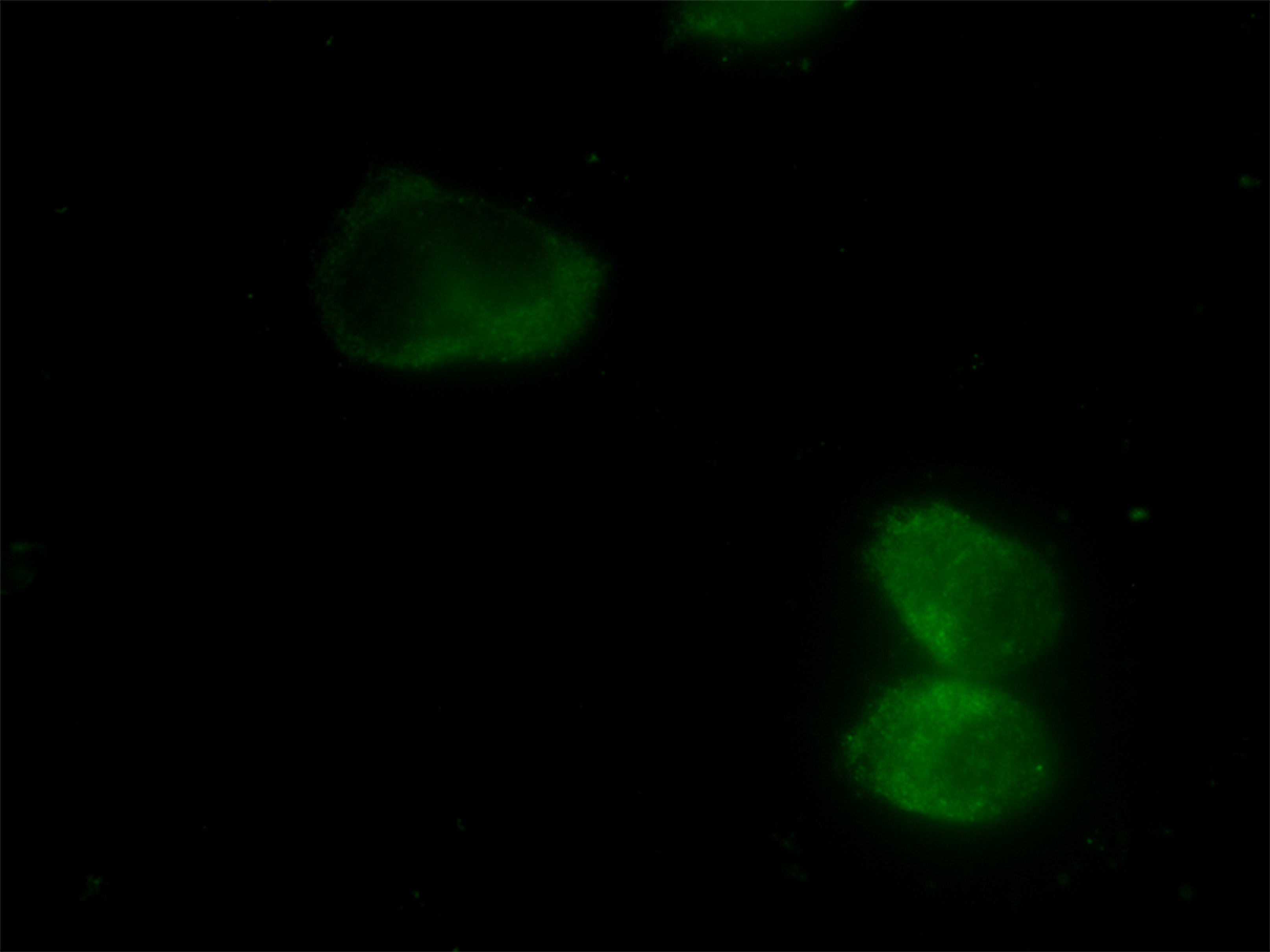

Supplement: S8 Data — (ZIP) [file pgen.1010366.s012.zip › S1D SV-HUC-1 sh-NC N-cadherin.png]

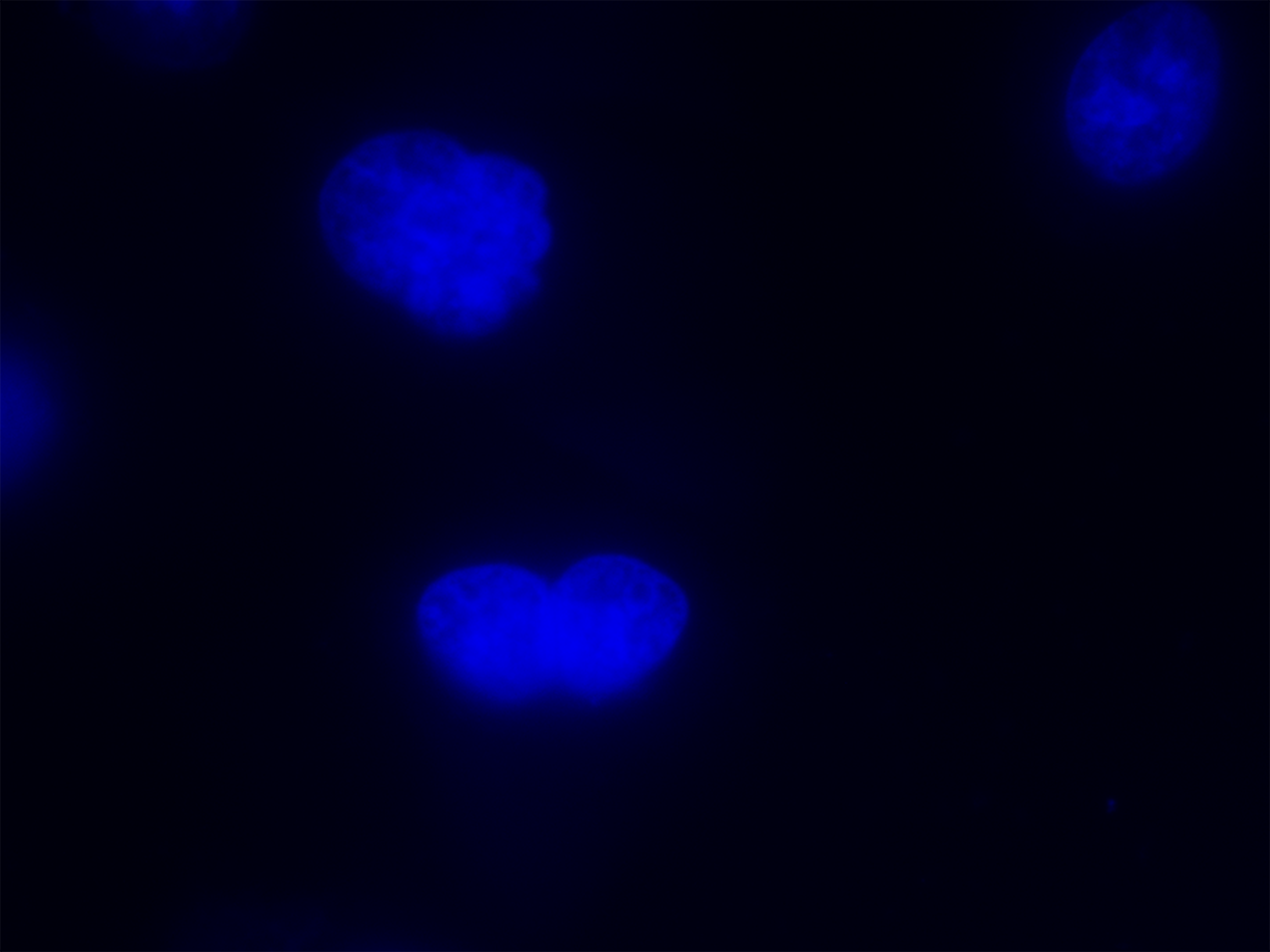

Supplement: S8 Data — (ZIP) [file pgen.1010366.s012.zip › S1D SW780 sh-METTL14 DAPI.png]

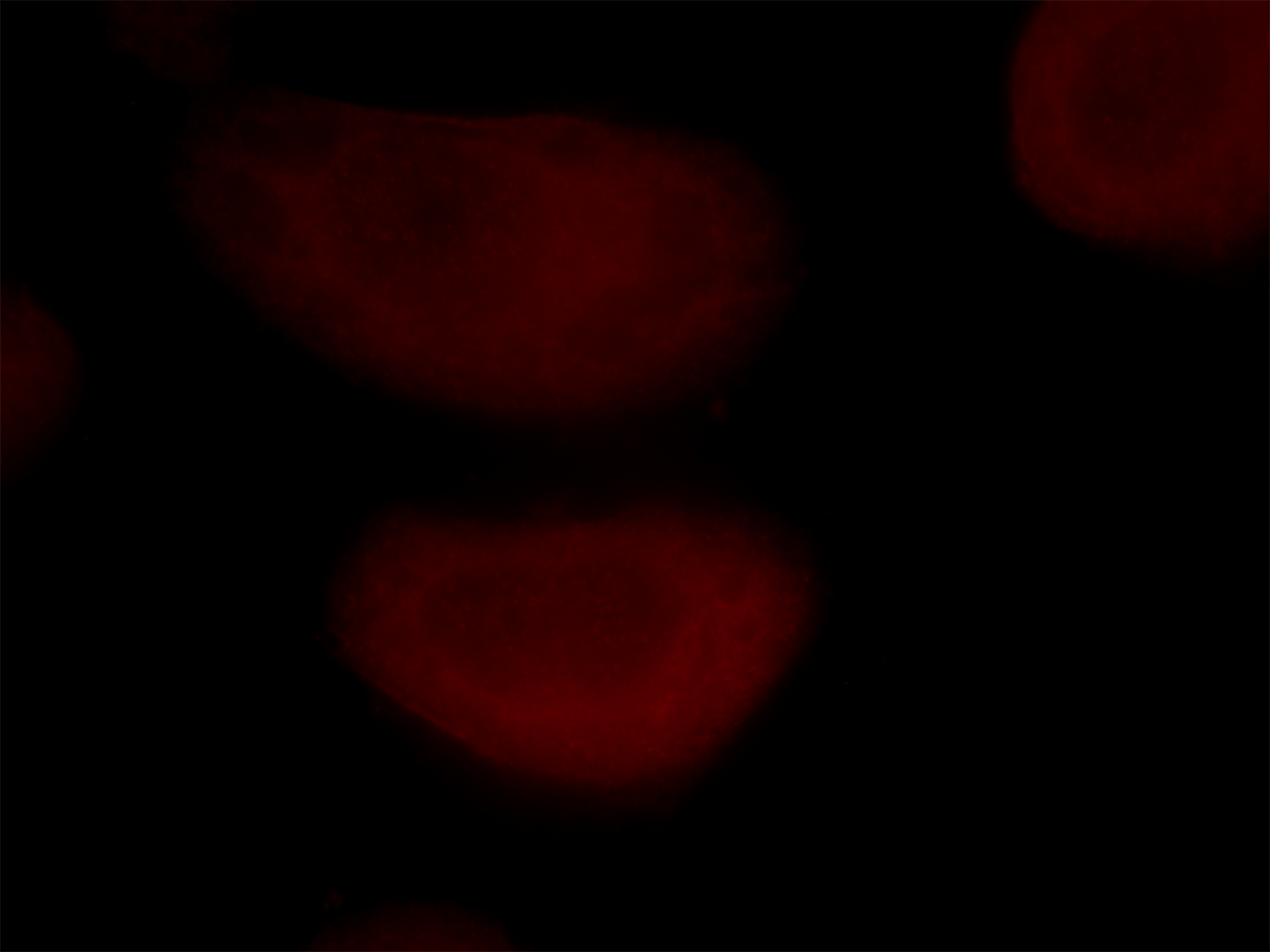

Supplement: S8 Data — (ZIP) [file pgen.1010366.s012.zip › S1D SW780 sh-METTL14 E-cadherin.png]

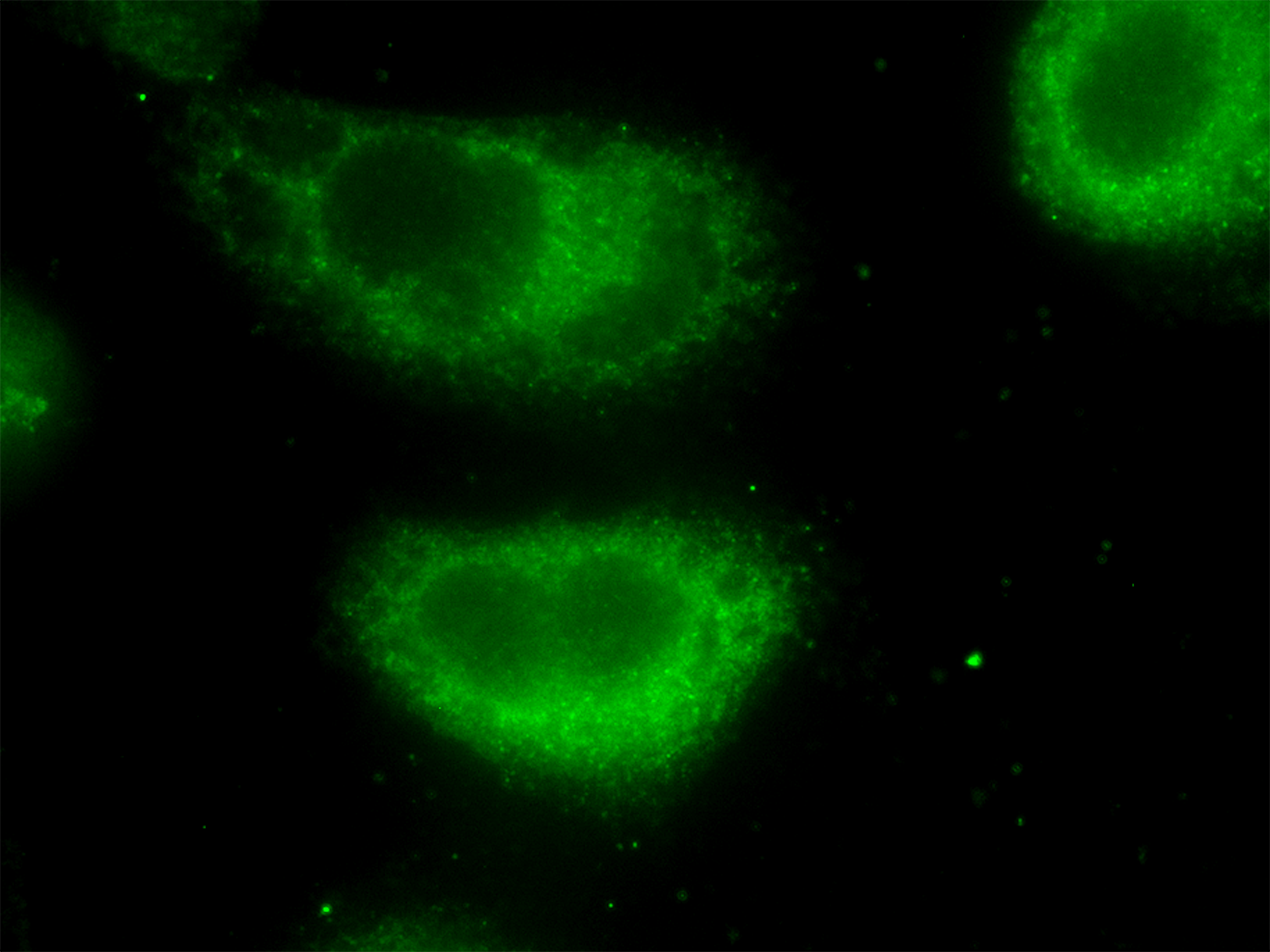

Supplement: S8 Data — (ZIP) [file pgen.1010366.s012.zip › S1D SW780 sh-METTL14 N-cadherin.png]

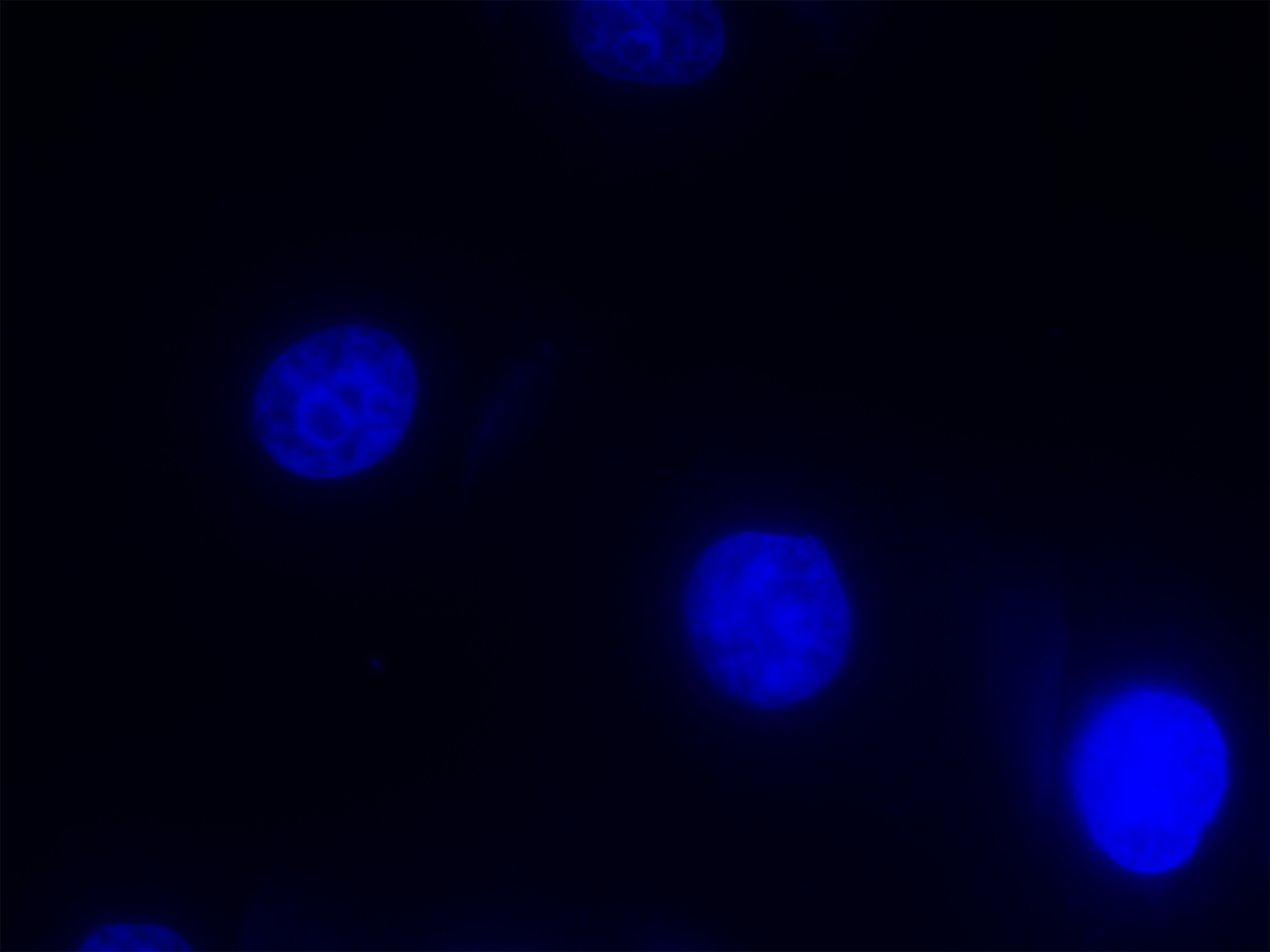

Supplement: S8 Data — (ZIP) [file pgen.1010366.s012.zip › S1D SW780 sh-NC DAPI.png]

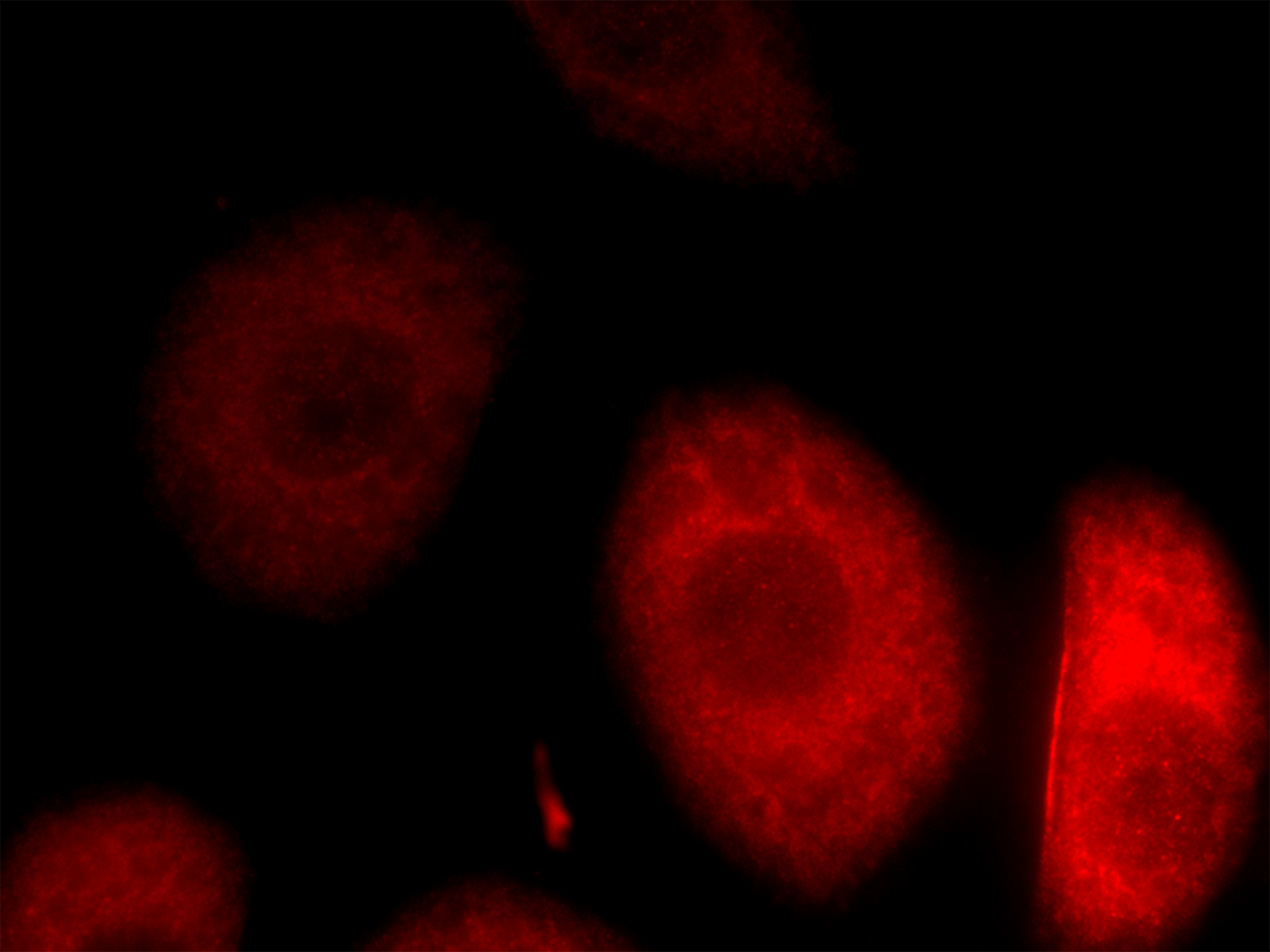

Supplement: S8 Data — (ZIP) [file pgen.1010366.s012.zip › S1D SW780 sh-NC E-cadherin.png]

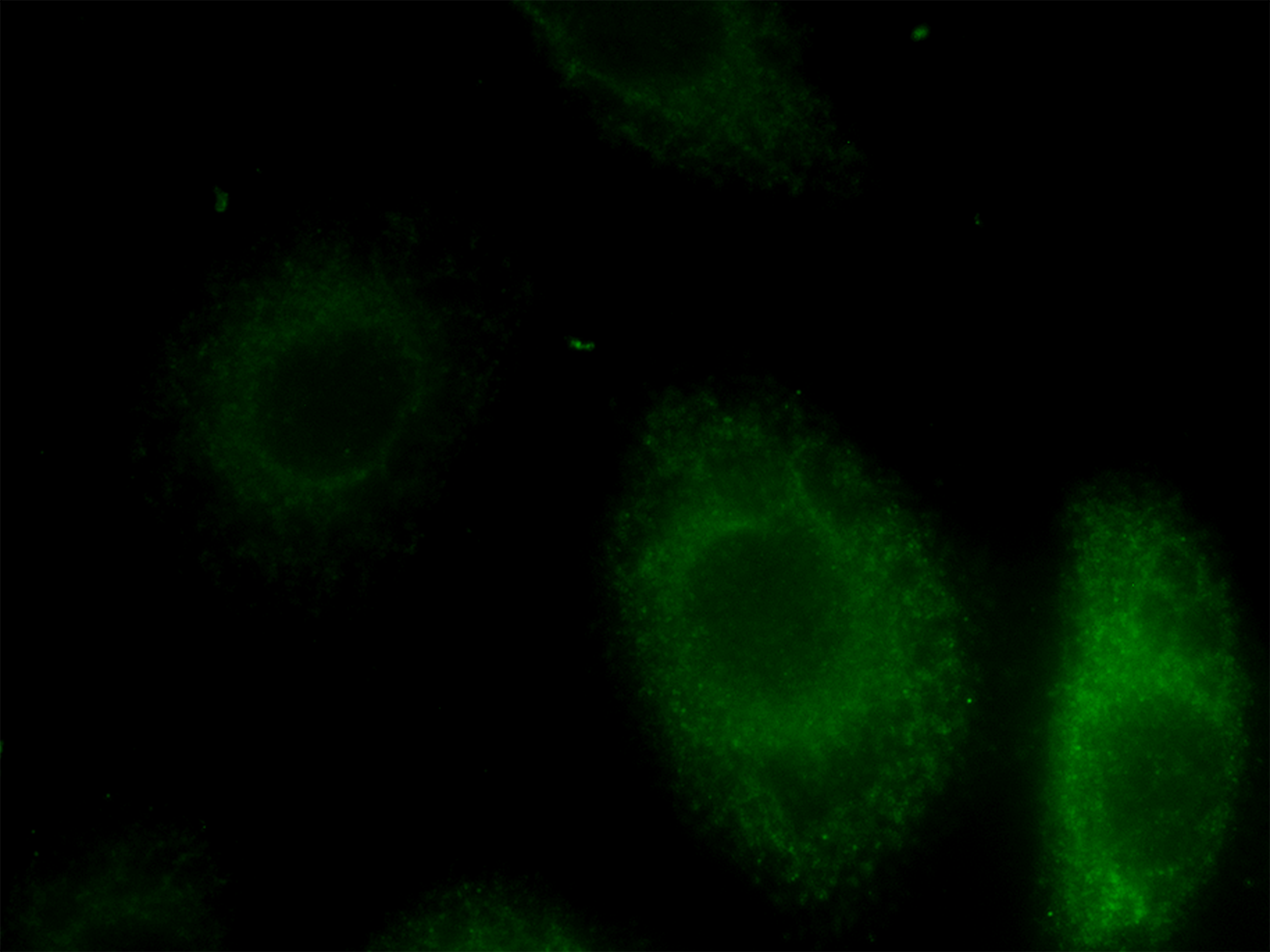

Supplement: S8 Data — (ZIP) [file pgen.1010366.s012.zip › S1D SW780 sh-NC N-cadherin.png]
