# Supplementary material for: The feedback loop of METTL14 and USP38 regulates cell migration, invasion and EMT as well as metastasis in bladder cancer
Source: PLoS Genet. 2022 Oct 26;18(10):e1010366. doi: 10.1371/journal.pgen.1010366 (PMC9605029; doi:10.1371/journal.pgen.1010366)
Supplement: S1 Text — (DOCX) [file pgen.1010366.s013.docx]

**Abbreviations**

Actinomycin D ActD

Analysis of variance ANOVA

Bladder cancer BCa

Bladder urothelial carcinoma BLCA

Co-immunoprecipitation Co-IP

Cycloheximide CHX

Deubiquitinating enzymes DUBs

Enhanced chemiluminescence ECL

Epithelial–mesenchymal transition EMT

Hematoxylin and eosin H&E

Immunofluorescence IF

JAMM/MPN domain-associated metallopeptidases JAMMs

Machado–Joseph disease protein domain proteases MJDs

Messenger RNA mRNA

Methylated RNA immunoprecipitation Me-RIP

Methyltransferase-like 14 METTL14

Monocyte chemotactic protein-induced protein MCPIP

N6-methyladenosine m6A

Ovarian-tumor proteases OTUs

Quantitative real-time PCR RT-qPCR

RNA-binding protein immunoprecipitation RIP

RNA-inducing silenced complexes RISCs

Short hairpin RNAs shRNAs

Ubiquitin carboxy-terminal hydrolases UCHs

Ubiquitin-specific proteases USPs

Ubiquitin specific peptidase 38 USP38
